# Intrapleural administration with traditional Chinese medicine injections (*Sophorae flavescentis* preparations) in controlling malignant pleural effusion: a clustered systematic review and meta-analysis

**DOI:** 10.3389/fphar.2025.1519794

**Published:** 2025-04-24

**Authors:** Yan Zhang, Zheng Xiao, Hui Liu, Da-Chun Cai, Yao-Qin Luo, Jiao Xu, Feng Luo, Jun Huang, Yan-Yan Jin, Teng-Yang Fan, Jun Zhang, Xue Xiao, Ji-Hong Feng

**Affiliations:** ^1^ Evidence-Based Medicine Center, MOE Virtual Research Center of Evidence-based Medicine at Zunyi Medical University, Affiliated Hospital of Zunyi Medical University, Zunyi, Guizhou, China; ^2^ Department of General Practice, Affiliated Hospital of Zunyi Medical University, Zunyi, Guizhou, China; ^3^ Geriatric Medicine Department, Affiliated Hospital (GuiAn) of Guizhou Medical University, Guiyang, Guizhou, China; ^4^ Department of Oncology, Tongren People’s Hospital, Tongren, Guizhou, China; ^5^ Department of Pharmacy, Affiliated Hospital of Zunyi Medical University, Zunyi, Guizhou, China; ^6^ Internal Medicine Department, 96603 Hospital, Huaihua, Hunan, China; ^7^ Department of Oncology, Lishui People’s Hospital, Sixth Affiliated Hospital of Wenzhou Medical University, Lishui, Zhejiang, China

**Keywords:** malignant pleural effusions, Radix Sophorae Flavescentis, compound kushen injection, matrine injection, Kangai injection, clustered systematic review Bibby, A.C., Dorn

## Abstract

**Introduction:**

*Sophorae flavescentis* (*kushen*) preparations are widely used to control malignant pleural effusion (MPE) through intrapleural perfusion.

**Objectives:**

This analysis aims to verify the therapeutic values of perfusion with *kushen* preparations for controlling MPE, reveal the optimal treatment plan, suitable population, and usage, and to demonstrate their clinical effectiveness and safety.

**Methods:**

We performed and reported this systematic review/meta-analysis (PROSPERO: CRD42023430139) following the Preferred Reporting Items for Systematic Reviews and Meta-Analyses (PRISMA) guidelines. All randomized controlled trials (RCTs) concerning perfusion with *kushen* preparation for MPE were collected from Chinese and English databases. We clustered all eligible studies into multiple homogeneous treatment units, assessed their methodological quality using a RoB 2, pooled the data from each unit, and summarized the quality of the evidence.

**Results:**

We included 83 RCTs reporting three types of *kushen* preparation: compound *kushen* injection (CKI), *kang’ai* injection, and matrine injection. All trials were clustered into perfusion with CKI alone or with the addition of sclerosants, *kang’ai*, or matrine-plus platinum for controlling MPE. Compared with cisplatin alone, perfusion with CKI alone displayed a similar complete response, pleurodesis failure, and pleural progression (odds ratios =1.10, 95% CI 0.76 to 1.60; 0.80, 0.56 to 1.14; 0.63, 0.33 to 1.21). Of 14 homogeneous treatment plans, perfusion with CKI and cisplatin significantly improved the complete response (2.71, 2.30 to 3.19) and showed low pleurodesis failure (0.26, 0.22 to 0.32), pleural progression (0.22, 0.14 to 0.36), myelosuppression (0.34, 0.24 to 0.47), neutropenia (0.35, 0.26 to 0.46), gastrointestinal reaction (0.36, 0.29 to 0.44), hepatorenal toxicity (0.42, 0.28 to 0.63 and 0.32, 0.24 to 0.44), and fever (0.50, 0.30 to 0.82). These results were moderate quality (⊕⊕⊕Ο) supported by firm or conclusive information. Additionally, perfusion with *kang’ai* or matrine and cisplatin also improved the complete response (3.04, 1.76 to 5.26 and 1.87, 1.26 to 2.78) and displayed low pleurodesis failure (0.23, 0.14 to 0.41 and 0.27, 0.17 to 0.44). The results were moderate to low quality (⊕⊕⊕Ο to ⊕⊕ΟΟ).

**Conclusion:**

Current moderate evidence demonstrates that CKI may be an effective palliative intervention for MPE which, combined with cisplatin, may be an optimal treatment plan. *Kang’ai* or matrine may be other potential choices.

**Systematic Review Registration:**

https://www.crd.york.ac.uk/PROSPERO/view/CRD42023430139

## 1 Introduction

The dried root of the shrub *Sophora flavescens* Aiton (Chinese name: *kushen*) is an important herbal medicine in China, Japan, Korea, India, and in some of Europe (He et al., 2015;Liang et al., 2019). It contains active components such as matrine, oxymatrine, sophoridine, flavonoids, alkylxanthones, quinones, triterpene glycosides, fatty acids, and essential oils (Cao and He, 2020; Chen et al., 2021; Chen et al., 2022). Its matrine and oxymatrine show significant anti-tumor activities by inhibiting tumor cell proliferation, inducing apoptosis, regulating the tumor microenvironment, and down-regulating cancer-related inflammation (Guo et al., 2015; Ma et al., 2016; Cao and He, 2020; Chen et al., 2021; Chen et al., 2022; Liu et al., 2023). In China, three traditional Chinese medicine injections (TCMIs)—compound *kushen* injection (CKI), *kang’ai*, and matrine injection—were developed, with *S. flavescens* extracts including matrine and oxymatrine as the core components (Supplementary Material S1 and Supplementary Table S1). In this analysis, we defined three types of injection as *S. flavescens* (*kushen*) preparations. CKI mainly contains ethanol and water extracts such as matrine, oxymatrine, and sophoridine, which are extracted from *S. flavescens* Aiton (*kushen*) and *Heterosmilax yunnanensis* Gagnep (*baituling*) (Guo et al., 2015; Ma et al., 2016; Liu et al., 2023). *Kang’ai* injection contains multiple ingredients including *Astragalus* polysaccharides, astragalosides, ginsenosides, ginseng polysaccharides, and oxymatrine, which are extracted from *kushen*, ginseng (*Panax ginseng* C.A. Mey), and *Astragalus membranaceus* (Fisch.) Bunge (Fabaceae) (Wan et al., 2018; Sun et al., 2021). Matrine injection is a chemical drug derived from *kushen*. Clinically, three types of *kushen* preparations have been approved by the China Food and Drug Administration for adjuvant therapy of solid tumors (Ma et al., 2016; Wang et al., 2016; Li H. et al., 2019; Liu et al., 2022; Liu et al., 2023).

Malignant pleural effusion (MPE), a frequent complication often secondary to metastases to the pleura, originates from intra- or extra-thoracic malignant tumors (Hassan et al., 2021; Gayen, 2022). Patients with MPE often experience progressive breathlessness, tumor progression, and poor survival. Currently, effective control of pleural effusion, improvement of clinical symptoms, and quality of life (QOL) have become the main treatment goals for symptomatic MPE and suspected expandable lung patients (Bibby et al., 2018; Feller-Kopman et al., 2018). Excluding malignant tumors, CKI, *kang’ai*, and matrine injections are commonly used to control MPE through intrapleural perfusion (Yang et al., 2016; Wu et al., 2018; Li B. et al., 2019; Xu et al., 2022). According to the Cochrane systematic evaluation, five systematic reviews/meta-analyses (SRs/meta-analyses) (Tang et al., 2014; Biaoxue et al., 2015; Xu et al., 2015; Yang et al., 2016; Wu et al., 2018) reported that *kushen* preparations might increase clinical response rate and improve QOL with a low adverse drug reactions (ADRs) in MPE. But these SRs/meta-analyses (Tang et al., 2014; Biaoxue et al., 2015; Xu et al., 2015; Yang et al., 2016; Wu et al., 2018) exhibited significant clinical heterogeneity, conducted inappropriate data analysis, and involved 16 ineligible studies (Supplementary Tables S3, S4). They also lacked rigorous and reasonable methodologies such as prior planning and systematic retrieval. These deficiencies undermine the credibility of their conclusions, which easily mislead clinical decision-making.

At present, no evaluation has revealed their clinical value for perfusion with *kushen* preparation alone for MPE. No evidence has confirmed its optimal treatment plan, indications, usage, and how to reasonably apply *kushen* preparation to achieve expected clinical efficacy and safety. Since the publication of the latest SR/meta-analysis in 2018, (Wu et al., 2018), 23 trials (Supplementary Material S3) have been published (Huang, 2021; Feng and Shi, 2023; Lin et al., 2023; Wang R. et al., 2023). We further performed a registered SR/meta-analysis to verify the therapeutic value of *kushen* preparations for controlling MPE, reveal their optimal treatment plan, suitable population and usage, and demonstrate their clinical effectiveness and safety. A new evidence framework will be developed for clinical decision-making about the reasonable application of *kushen* preparations to control MPE and further new research projects.

## 2 Materials and methods

**FIGURE 1:**
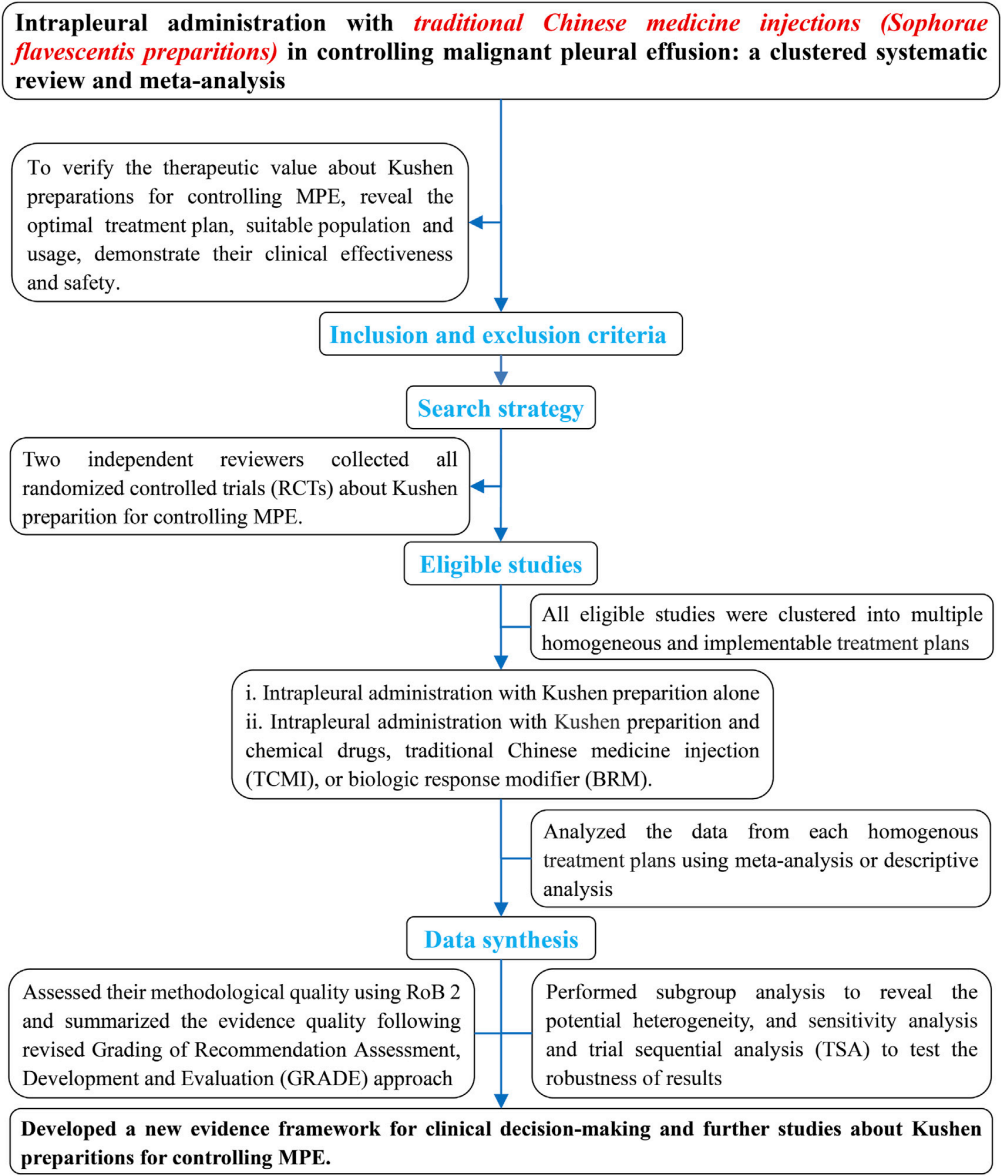
Implementation framework.

*Kushen* preparations mainly include CKI, *kang’ai,* and matrine. To verify their therapeutic value for controlling MPE, we systematically and comprehensively collected all eligible studies about *kushen* preparations for controlling MPE (Figure 1). These were clustered into multiple homogeneous and implementable treatment units such as CKI alone, and CKI, *kang’ai*, or matrine and cisplatin, nedaplatin, or carboplatin. We then further evaluated their methodological quality and pooled the data from each treatment unit and finally summarized and developed an evidence framework for rational drug use decision-making and future research projects. We registered this analysis on PROSPERO (CRD42023430139) and reported all findings according to the Preferred Reporting Items for Systematic Reviews and Meta-Analyses guidelines (PRISMA 2020 Checklist) (Page et al., 2021). During the retrieval, selection, evaluation of methodological quality, data collection, statistical analysis, and summary of evidence, any disagreements were resolved through discussion with each other or with Zheng Xiao. Ethical approval was not required as the materials were published studies.

### 2.1 Inclusion and exclusion criteria

According to the PICOS model, we established the following criteria for all eligible studies to meet.

(i). Only optimum trials as randomized controlled trials (RCTs) without restrictions on follow-up, institutions, language, and publication time.
(ii). All patients presented with MPE and dyspnea which was diagnosed by thorax imaging, pleural fluid analysis, cytology, or pleural biopsy. All patients had normal liver, kidney, and heart function, and no limitations on tumor type and pleural fluid volume.
(iii). The interventions were *kushen* preparations such as CKI, *kang’ai*, and matrine injection through intrapleural perfusion. Both groups did not receive any intrapleural perfusion 1 month before treatment. The experimental groups received *kushen* preparation alone or in combination with other sclerosants, and the controls received sclerosants alone such as chemical drugs, biological response modifiers (BRMs), or TCMI.
(iv). The main outcomes are clinical response and survival, and secondary outcomes are QOL and adverse events.

All ineligible studies must meet the following criteria: studies about patients with ascites or pericardial effusion; all patients receiving systemic chemotherapy, local hyperthermia or oral traditional Chinese medicine (TCM); both groups receiving *kushen* preparation; studies with unclear objectives; without any data about clinical responses, survivals, QOL, or adverse events.

### 2.2 Outcomes definition

The primary outcomes are clinical response and survival. Referring to previous studies (Paladine et al., 1976; Kessinger and Wigton, 1987; Keeratichananont et al., 2015; Jie Wang et al., 2018; Dipper et al., 2020; Xiao et al., 2020a), we integrated both Millar and Ostrowskimj criteria to measure the clinical responses as: (i) complete response (CR) is the disappearance of pleural effusion for more than 30 days, or the lack of accumulation of fluid; (ii) partial response (PR) is less than 50% reduction of pleural effusion for more than 30 days; (iii) no response (NR)/stable disease (SD) is less than 50% reduction of pleural effusion or less than 25% increase or the recurrence of fluid accumulation without further therapy; (iv) pleural progression (PP) is more than 25% increase of pleural effusion or symptomatic fluid accumulation again requiring further therapy. We set the pleurodesis failure as no response or stable disease plus pleural progression and assessed the clinical responses using complete response, pleurodesis failure, and pleural progression (Supplementary Material S2). Long-term survival was assessed by using overall survival (OS), progression-free survival (PFS), OS, and PFS rates. According to the Karnofsky performance status (KPS) scale, when a KPS score increased ≥10 after treatment, QOL was improved.

Adverse events (AEs) were assessed by using ADRs and thoracentesis-related adverse events (TRAEs). According to World Health Organization (WHO) or Common Terminology Criteria for Adverse Events (CTCAEs) criteria (Miller et al., 1981; Trotti et al., 2003), ADRs were measured by using the indicators myelosuppression, neutropenia, thrombocytopenia, anemia, hepatorenal toxicity, gastrointestinal reactions, thoracodynia, and fever. TRAEs were measured by using indicators including treatment-related death, respiratory failure, dyspnea, pneumothorax, chest infection, drainage tube detachment, tumor metastasis along the indwelling duct, catheter-related infection, or subcutaneous emphysema.

### 2.3 Retrieval and selection strategies

Adhering to a retrieval logic of patient plus intervention, we customized the retrieval strategies for each database using MeSH and free words (Supplementary Material S3). Yan Zhang and Hui Liu independently searched all related studies about “*Kushen* preparations in controlling MPE” from Chinese and English electronic databases (to February 2025) including the Guizhou Digital Library, SinoMed, China National Knowledge Infrastructure Database, WanFang Database, Chinese Scientific Journals Full-text Database, PubMed, Embase, Web of Science, and Cochrane Central Register of Controlled Trials (Issue 2, February 2025). We collected ongoing trials from the Chinese Clinical Trial Registry (http://www.chictr.org.cn), WHO International Clinical Trials Registry Platform (http://apps.who.int/trialsearch/), and US clinical trials (https://clinicaltrials.gov). Finally, we also identified eligible studies from the references of relevant SRs or network meta-analysis. Hui Liu and Yan Zhang independently selected eligibles and excluded ineligible studies following a predesigned inclusion and exclusion criteria.

### 2.4 Assessment of methodological quality

For clinical responses, survivals, QOL, or adverse events, Da-chun Cai and Jiao Xu independently applied a revised Cochrane tool (RoB 2) to assess methodological quality arising from five domains: randomization process (D1), intended interventions (D2), missing outcome data (D3), outcomes measurement (D4), and selective reporting of results (D5) (Sterne et al., 2019; Higgins et al., 2021). We judged each quality based on the domain algorithm and made an overall judgment.

### 2.5 Data collection

Yao-Qin Luo and Da-chun Cai independently collected all data using a predesigned data extraction form. The data were first author, time of publication, methodological features, demographic characteristics and cases; characteristics of patients as tumor types, pleural fluid volume, anticipated survival time (AST), KPS score, treatment history, and recurrence; drainage methods as indwelling pleural catheters (IPCs) or thoracentesis; *kushen* preparations, treatment dose, frequency and times, and sclerosants and uses; follow-up protocol, research institutions, criterion and time of evaluation. The outcomes were: complete response, pleurodesis failure, pleural progression, PFS, OS, QOL, ADRs, and TRAEs. Additionally, the authors of papers were contacted about available survival data. If they were unavailable, the Kaplan–Meier survival curves were transformed into data using Engauge Digitizer 4.1 (Guyot et al., 2012; Xiao et al., 2018).

### 2.6 Statistical analysis

All eligible studies were clustered into multiple homogeneous treatment units, and we further analyzed their clinical effectiveness and safety. The odds ratios (ORs) and their 95% confidence interval (CI) were applied to measure the complete response, pleurodesis failure, pleural progression, OS rate, QOL, ADRs, and TRAEs, with *p* < 0.05 being identified as statistically significant. Cochran’s χ^2^ test and *I* ^2^ statistic were performed to identify statistical heterogeneity among each unit. If the results showed significant heterogeneity and inconsistent directions or involved a single trial, we used forest plots to describe the result. When *p* ≥ 0.1 and *I* ^2^ ≤ 50%, a fixed-effects model (FEM) was applied to pool the OR and their 95% CI. When *p* < 0.1, *I* ^2^ > 50%, and the results had consistent direction, a random-effects model (REM) was applied. Yan Zhang and Feng Luo independently applied Review Manager 5.4 to pool the data from each unit. If the outcomes involved more than ten trials, a funnel plot and Egger’s test (STATA V.15.0 software, 401506209499) were applied to identify potential publication bias.

Referring to previous experience (Xiao et al., 2020b; Wang et al., 2021; Wang et al., 2022; Wang C. Q. et al., 2023), a subgroup analysis was implemented to reveal the potential clinical heterogeneity among the main treatment plans with enough trials to analyze the effects of patient related factors, interventions, and evaluation criteria on clinical responses and to further identify the suitable population and optimum usage. We further implemented univariate random effects meta-regression analysis to reveal the correlation between each factor and clinical responses and post hoc multiple regression analysis to identify it.

Following underestimation of effectiveness/safety, we implemented sensitivity analysis to identify robustness (Xiao et al., 2020b; Wang et al., 2021; Wang et al., 2022; Wang C. Q. et al., 2023). The consistency of results before and after excluding both trials with high risk and overestimation were analyzed. If consistency was good, the result was robust; otherwise, it was poor. To identify the required information size (RIS) for the results of main treatment units (Thorlund et al., 2016), we further applied Trial Sequential Analysis (TSA) software (version 0.9.5.10 Beta) to implement the analysis. In the light of previous experience, we set the risk of type I error as 5% with a power of 80%, relative risk reduction (RRR) as 25% for clinical responses and QOL, and 20% for adverse events (AEs) (Wetterslev et al., 2008; Thorlund et al., 2009). We used control event rates from this analysis for these calculation, and adjusted the information size for diversity (Wetterslev et al., 2009).

### 2.7 Summary of evidence quality

We integrated the results of sensitivity analysis into the GRADE approach (Guyatt et al., 2008; Xiao et al., 2020b; Wang et al., 2021; Wang et al., 2022; Wang C. Q. et al., 2023) and developed a revised approach to summarize the evidence. Quality was identified as “high”, moderate”, “low”, and “very low” following five domains: risk-of-bias of results, heterogeneity, indirectness, imprecision, and publication bias (Supplementary Material S2). Jun Huang and Yan-Yan Jin independently applied the GRADE profiler to summarize the evidence quality and generated the absolute estimates of effect for outcomes.

## 3 Results

### 3.1 Search results

**FIGURE 2:**
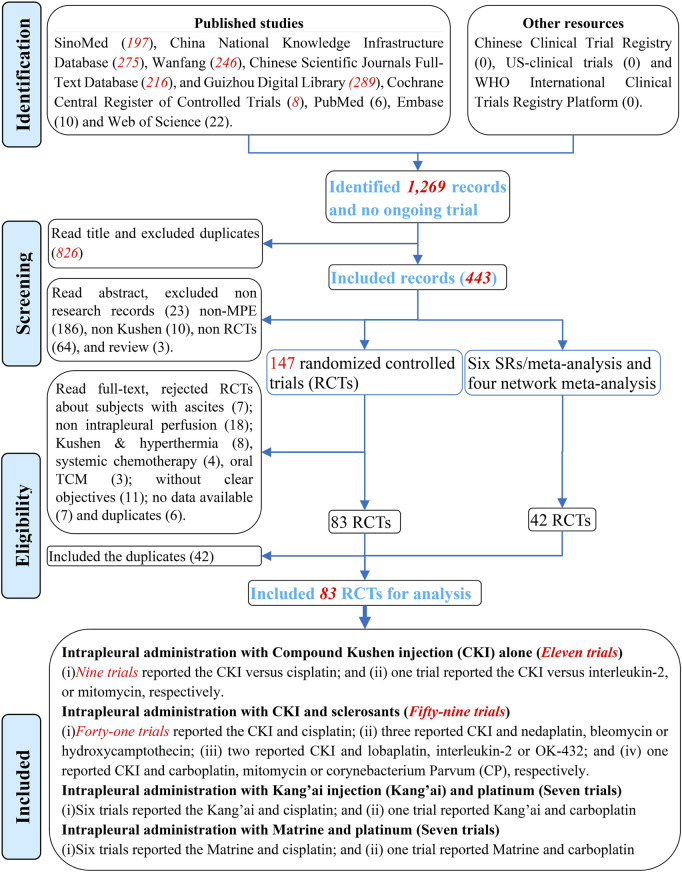
PRISMA 2020 flow diagram for selecting eligible studies.

After retrieval, 1,269 records were identified. Two reviewers read the titles, excluded duplicates, and identified 443 records. After screening abstracts and excluding irrelevant and non RCTs, 147 RCTs, six SRs/meta-analyses (Tian et al., 2010; Tang et al., 2014; Biaoxue et al., 2015; Xu et al., 2015; Yang et al., 2016; Wu et al., 2018) and four network meta-analyses (Yang et al., 2017; Li B. et al., 2019; Li, 2022; Xu et al., 2022) were selected. Further evaluating full-texts and excluding 64 ineligible studies (Supplementary Material S3), 83 were considered eligible. Additionally, 42 studies were selected from previous studies. Finally excluding duplicates, 83 eligible studies were selected for this analysis (Figure 2).

### 3.2 Characteristics of included studies

We clustered the 83 eligible studies from 2001 to 2023 into four themes: intrapleural perfusion with CKI alone, CKI and sclerosants, *kang’ai* or matrine, and platinum for controlling MPE. Eleven trials reported CKI alone (Table 1a). CKI and sclerosant developed three comparisons as CKI-versus-cisplatin (Yuan, 2007; Hu Q. et al., 2008; Chen, 2010; Liang et al., 2011; Chen, 2013; Xing, 2013; Wang and Zhou, 2016; Yan et al., 2016; Wang R. et al., 2023), mitomycin (Zhang, 2011), or interleukin-2 (Huang, 2013). All trials recruited 796 inpatients—426 male and 244 female patients aged 20–82 years. Receiving CKI were 396 patients, while another 400 received sclerosants alone. Perfusion with CKI and sclerosants was reported in 59 trials (Table 1b). The CKI and chemical drug or BRM developed ten treatment plans: perfusion with CKI and cisplatin, nedaplatin (Li, 2014; Zhang S. et al., 2015; Li et al., 2017), bleomycin (Chen and He, 2003; Liu and Wan, 2011; Sun, 2012), hydroxycamptothecin (He et al., 2009; Wu et al., 2014; Cai and Wang, 2019), lobaplatin (Liu and Xu, 2016; Huang, 2021), carboplatin (He and Xie, 2010), mitomycin (Zhang et al., 2013), interleukin-2 (Hao and Liang, 2007; Zhou et al., 2010), OK-432 (Wei et al., 2014; Zhong et al., 2015), and *Corynebacterium parvum* (Huang et al., 2012). There were 41 trials which evaluated perfusion with CKI and cisplatin, recruiting 2,823 inpatients aged 15–91, with 1,346 male and 909 female patients. Some 1,424 patients received perfusion with CKI and cisplatin, while another 1,399 received cisplatin alone. CKI was administrated 10–60 mL/time, once to thrice per week, lasting one to twelve times; the cisplatin was administrated with 20–80 mg/time. *Kang’ai* or matrine and platinum developed four plans. Six trials involving 334 inpatients aged 36–84 years (Zhang, 2006; Hu J. et al., 2008; Xu and Xiong, 2008; He, 2011; Qu et al., 2012; Wang, 2016) evaluated perfusion with *kang’ai* and cisplatin (Table 1c). Received *kang’ai* and cisplatin were 168 patients, while another 166 received only cisplatin. *Kang’ai* was administrated 40–60 mL/time, once or twice per week, lasting one to four times. Six trials recruiting 319 inpatients aged 30–85 (Du et al., 2009; Li and Yang, 2009; He, 2010; Wang et al., 2010; Ji, 2011; Ji et al., 2012) evaluated perfusion with matrine and cisplatin (Table 1d). A total of 167 patients received matrine and cisplatin, while another 152 only received cisplatin. Matrine was administrated 150–800 mg/time, once a week, lasting 2 to 6 weeks.

**TABLE 1:** Characteristics of included studies.

First author, year	Malignant pleural effusions								Interventions			Evaluation times	Criteria	Outcomes
	Tumor	Volume	KPS	TH	AST	E/C	M/F	Years	DM	*Kushen*, dose, times (dose*)	Sclerosants			
a. Intrapleural administration with compound *kushen* injection (CKI) alone														
CKI versus cisplatin (nine trials)														
Yuan (2007)	MTs	Un	≥40	PT	Un	26/26	32/20	36–87	IPC	20 mL, 2–3 times/w, 4–6 times	40 mg/m^2^	6–7 weeks	Millar, Un	O1-3
Hu et al. (2008b)	LC	Small to large	≥50	Un	>3	20/20	25/15	62–67	IPC	20 mL, 2 times/w, 4 times	30 mg	9 weeks	Millar, Un	O1-3
Chen (2010)	MTs	Large	Un	Un	Un	28/30	31/27	59–77	IPC	20 mL, 1 time/w, 3 times	30 mg/m^2^	2 years	Millar, WHO	O1,3,4
Liang et al. (2011)	MTs	Un	>50	Un	Un	56/54	Un	35–83	IPC	20 mL, 1 time/w, 3–4 times	40 mg/m^2^	7–8 weeks	Ostrowskimj, WHO	O1-3
Chen (2013)	MTs	Moderate to large	≥70	Un	Un	40/40	46/34	20–82	IPC	40 mL, 1 time/w, 4 times	40 mg/m^2^	8 weeks	Millar, Un	O1-3
Xing (2013)	LC	Moderate to large	>50	Un	≥3	45/42	52/35	43–79	IPC	20 mL, 1–2 time/w, 4 times	40–60 mg	8 weeks	Millar, Un	O1-3
Yan et al. (2016)	MTs	Un	>60	Un	>3	20/30	Un	45–81	IPC	20 mL, 1 time/w, 4 times	40 mg	8 weeks	Ostrowskimj, Un	O1-3
Wang and Zhou (2016)	MTs	Un	Un	PT	Un	30/30	51/39	36–81	IPC	40 mL, 2 times/w, 7 times	40 mg	8 weeks	Ostrowskimj, WHO	O1,3
Wang et al. (2023b)	BC	Small to large	Un	Un	>3	16/15	0/31	57–75	IPC	50 mL, 2 times/48 h,i2 times	40 mg	6 weeks	Millar, Un	O1,3
CKI versus Interleukin-2 (one trial)														
Huang (2013)	HCC	Un	Un	Un	Un	65/63	117/11	45.3 ± 3.2/44.8 ± 2.9	Un	20 mL, 1 time/day, 5 times	1 MU	7 weeks	Ostrowskimj, Un	O1
CKI versus mitomycin (one trial)														
Zhang (2011)	MTs	Un	>60	Un	>3	50/50	68/32	38–76	IPC	40 mL, 1 time/w, 3 times	10 mg	7 weeks	Ostrowskimj, Un	O1-3
b. Intrapleural administration with CKI and sclerosants														
CKI and cisplatin versus cisplatin (41Trials)														
Huang (2007)	LC	Large	Un	Un	>1	20/18	28/10	35–70	Thora*	20 mL, 1 time/w, 1-2 times	40 mg	5–6 weeks	Ostrowskimj, Un	O1,3
Lin et al. (2007)	MTs	Moderate to large	Un	Un	Un	33/33	40/26	36–75	Thora*	20 mL, 1 time/w, Un	60 mg	Un	Millar, Un	O1,3
Pan et al. (2007)	MTs	Un	≥60	Un	Un	36/34	43/27	60 ± 21	IPC	30 mL, 1 time/w, 2–4 times	40 mg	6–8 weeks	Ostrowskimj, WHO	O1-3
Zhang et al. (2008)	MTs	Un	>60	PT	Un	28/23	27/24	31–80	IPC	20 mL, 1 time/w, 4 times	20 mg	8 weeks	Ostrowskimj, Un	O1
Ding et al. (2009)	MTs	Un	≥60	Un	≥3	31/30	41/20	38–76	IPC	20 mL, 1 time/w, 3 times	30 mg	7 weeks	Ostrowskimj, WHO	O1-3
Li et al. (2009)	LC	Un	Un	Un	>1	30/30	49/11	35–70	Thora*	20 mL, 1 time/w, Un	40 mg	8 weeks	Ostrowskimj, Un	O1,3
He et al. (2010)	LC	Moderate to large	≥50	RT	>3	24/20	25/19	39–75	IPC	40 mL, 1 time/w, 3 times	40 mg	7 weeks	Ostrowskimj, WHO	O1,3
Wang (2010)	MTs	Un	>60	Un	>3	24/24	Un	55–82	IPC	20 mL, 2 times/w, 8 times	40 mg	8 weeks	Ostrowskimj, Un	O1,3
Chen et al. (2011)	MTs	Un	≥60	Un	Un	84/84	Un	38–85	IPC	60 mL, 1 time/w,3-5 times	40–60 mg	3 years	Ostrowskimj, Un	O1,3,4
Wei and Sun (2011)	MTs	Un	Un	Un	Un	35/35	40/30	21–75	IPC	20 mL, 1 time/w, 4 times	40 mg	8 weeks	Ostrowskimj, Un	O1
Chen and Liao (2012)	MTs	Moderate to large	≥60	Un	Un	43/43	60/26	35–68	IPC	30 mL, 1 time/w, 4 times	60 mg/m^2^	8 weeks	Millar, WHO	O1,3
Han et al. (2012)	LC	Un	>50	PT	>3	28/28	35/21	41–91	IPC	12–20 mL, 1time/w, 2-4 times	20–40 mg	8 weeks	Millar, WHO	O1-3
Yang (2012)	MTs	Moderate to large	≥60	Un	≥3	39/39	43/35	33–76	IPC	25 mL, 1 time/w, 4 times	40–60 mg	8 weeks	Ostrowskimj, CTEC3.0	O1-3
Zhuang et al. (2012)	HM	Un	Un	Un	Un	24/22	23/23	15–81	Thora*	10 mL, 1 time/w, 3–6 times	20 mg/m^2^	7–10 weeks	Millar, WHO	O1,3
Guo et al. (2013)	MTs	Moderate to large	≥50	PT	Un	31/31	Un	18–72	IPC	20 mL, 1 time/w, 4 times	40 mg	7–10 weeks	Ostrowskimj, WHO	O1-3
Han (2013)	MTs	Un	>60	Un	>3	90/90	93/87	34–82	IPC	40 mL, 1 time/w, 3 times	20 mg/m^2^	7 weeks	Millar, WHO	O1-4
Zheng and Jia (2013)	MTs	Un	>50	Un	>3	31/31	Un	51–78	IPC	30 mL, 1–2 times/w, Un	30 mg/m^2^	Un	Ostrowskimj, WHO	O1,3
Zhu et al. (2013)	MTs	Moderate to large	≥70	Un	Un	28/28	30/26	35–82	IPC	20 mL, 1 time/w, 4–6 times	60 mg	8–10 weeks	Millar, Un	O1,3
Chen et al. (2014)	MTs	Moderate to large	Un	PT	Un	30/30	38/22	60–83	IPC	20 mL, 1 time/w, 4 times	40 mg	8 weeks	Ostrowskimj, Un	O1-3
Jiang (2014)	MTs	Un	Un	Un	Un	34/34	37/31	34–81	IPC	20 mL, 1 time/w, 6 times	60 mg	10 weeks	Ostrowskimj, Un	O1-3
Xu (2014a)	MTs	Un	≥60	Un	Un	30/30	36/24	32–79	Thora*	60 mL, 1 time/w, 2–4 times	80 mg	6–8 weeks	Millar, Un	O1,3
Xu (2014b)	MTs	Un	Un	Un	Un	32/32	34/30	39–82	Thora*	40 mL, 1 time/w, 6 times	40 mg	10 weeks	Ostrowskimj, Un	O1-2
Liu and Li (2015)	LC	Un	>60	PT	>3	46/42	48/40	60.2 ± 8.2	IPC	20 mL, 1 time/w, 3 times	40–60 mg	8 weeks	Millar, Un	O1-3
Song and Jia (2015)	MTs	Un	≥70	Un	Un	59/59	64/54	42–73	IPC	25 mL, 1 time/w, 4 times	50 mg	8 weeks	Ostrowskimj, Un	O1,3
Yan et al. (2016)	MTs	Un	>60	Un	>3	35/30	Un	45–81	IPC	20 mL, 1time/w, 4 times	40 mg	8 weeks	Ostrowskimj, Un	O1-3
Qin and Fan (2016)	MTs	Un	Un	Un	Un	32/32	Un	38–76	IPC	20 mL, 1time/w, Un	60 mg	Un	Ostrowskimj, Un	O1,3
Huang et al. (2017)	MTs	Un	>60	Un	>3	30/30	43/17	62.8 ± 7.7; 3.3 ± 8.1	IPC	20 mL, 1 time/w, 4 times	40 mg	8 weeks	Ostrowskimj, Un	O1,3
Liu et al. (2017)	LC	Moderate to large	≥50	PT	Un	30/30	Un	32–76	IPC	20 mL, 1 time/w, 4 times	40 mg	8 weeks	Ostrowskimj, WHO	O1-3
Shi (2017)	LC	Large	≥50	PT	Un	30/30	39/21	34–78	IPC	20 mL, 1 time/w, 4 times	40 mg	8 weeks	Ostrowskimj, WHO	O1-3
Tang et al. (2018)	LC	Large	≥50	PT	Un	30/30	Un	33–77	IPC	60 mL, 2 times/w, 6 times	40 mg	7 weeks	Ostrowskimj, WHO	O1-3
Wu et al. (2019)	LC	Un	Un	Un	Un	25/25	30/20	39–68	IPC	40–60 mL, 1–2 times/w, 3–6 times	40–60 mg	8–9 weeks	Millar, WHO	O1-3
Wang et al. (2019)	LC	Un	Un	Un	Un	45/45	49/41	58–75	IPC	20 mL, 1 time/w, 3 times	40–60 mg	7 weeks	Millar, Un	O1-3
Peng (2020)	LC	Un	Un	Un	Un	25/25	29/22	41–70	IPC	40 mL, 3 times/w, 12 times	30 mg	8 weeks	Millar, Un	O1,3
Feng and Shi (2023)	LC	Moderate	>60	RT	>3	34/34	39/29	43–79	IPC	30 mL, 1 times/w, 3 times	40 mg	7 weeks	Millar, WHO	O1-3
Ning et al. (2001)	MTs	Un	Un	Un	Un	30/30	46/14	25–65	Thora*	20 mL, 1 time/w, 4 times (40 mg)	60 mg	8 weeks	Ostrowskimj, WHO	O1,3
Deng et al. (2008)	MTs	Un	Un	Un	Un	40/40	46/34	29–69	Thora*	20 mL, 1 time/w, Un (30 mg)	60 mg	Un	Ostrowskimj, WHO	O1,3
Li (2008)	MTs	Un	Un	Un	Un	32/32	51/13	29–73	IPC	20 mL, 1 time/w, 4 times (40 mg)	60 mg	8 weeks	Ostrowskimj, WHO	O1,3
Li and Tian (2011)	MTs	Moderate to large	Un	Un	Un	30/30	34/26	40–80	IPC	20 mL, 1 time/w, 2–3 times (40 mg)	60 mg	6–7 weeks	Ostrowskimj, Un	O1,3
Ran and Zang (2011)	MTs	Un	>60	Un	>3	30/30	33/27	35–79	IPC	25 mL, 1 time/w, 2–4 times (40 mg)	60 mg	6–8 weeks	Ostrowskimj, WHO	O1-3
Jiang and Li (2020)	MTs	Un	Un	Un	Un	30/30	44/16	43–71	IPC	20mL, un (20 mg)	40 mg	Un	Millar, Un	Q1,3
Lin et al. (2023)	MTs	Small to large	≥60	PT	>3	26/26	29/23	34–76	IPC	30 mL, 1time/w, 3 times	40 mg	7 weeks	Ostrowskimj, WHO	O1-3
CKI and nedaplatin versus Nedaplatin (Three trials)														
Li (2014)	MTs	Moderate to large	≥50	PT	>3	37/37	Un	36–78	IPC	25 mL, 2 times/w, 4 times	40–60 mg	6 weeks	Ostrowskimj, WHO	O1-3
Zhang et al. (2015a)	LC	Un	>60	Un	>3	56/56	68/44	35–78	IPC	30 mL, 1 time/w, 4 times	60 mg	8 weeks	Ostrowskimj, Un	O1-4
Li et al. (2017)	MTs	Un	>60	Un	>3	36/36	38/34	40–79	IPC	30 mL, 1 time/w, 4 times	60 mg	8 weeks	Ostrowskimj, Un	O1-3
CKI and carboplatin versus carboplatin (One trial)														
He and Xie (2010)	MTs	Moderate to large	Un	Un	>3	21/20	22/19	Un	IPC	40 mL, 1 time/w, 4 times	400 mg	8 weeks	Millar, WHO	O1,3
CKI and lobaplatin versus lobaplatin (two trials)														
Liu and Xu (2016)	LC	Moderate to large	Un	Un	Un	30/30	62/28	32–76	Thora*	30 mL, 1 time/w, 4 times	30 mg	8 weeks	Ostrowskimj, Un	O1,3
Huang (2021)	LC	Un	Un	Un	Un	25/25	27/23	44–81	IPC	30 mL, 1 time/w, Un	30 mg	Un	Millar, Un	O1
CKI and bleomycin versus bleomycin (three trials)														
Chen and He (2003)	MTs	Large	Un	Un	Un	15/14	18/11	40–75	IPC	20 mL, 1 time/w, 2 times (40 mg)	60 mg	8 weeks	Ostrowskimj, WHO	O1,3
Liu and Wan (2011)	MTs	Un	>60	Un	>3	37/30	37/30	45–76	IPC	20 mL, 1 time/w, 4 times	40 mg	8 weeks	Ostrowskimj, WHO	O1-3
Sun (2012)	MTs	Un	≥40	Un	>3	25/25	31/19	42–81	IPC	20 mL, 1 time/w, 4 times	45 mg	8 weeks	Ostrowskimj, Un	O1-3
CKI and hydroxycamptothecin versus hydroxycamptothecin (Three trials)														
He et al. (2009)	MTs	Un	Un	Un	Un	30/30	45/15	27–64	Thora*	30 mL, 1 time/w, 4 times	10 mg	8 weeks	Ostrowskimj, WHO	O1,3
Wu et al. (2014)	LC	Un	Un	Un	Un	42/40	50/32	60–82	Thora*	30 mL, 1 time/w, 4 times	5 mg	8 weeks	Millar, Un	O1,3
Cai and Wang (2019)	LC	Large	Un	Un	≥3	48/48	59/37	65.3 ± 7.1; 66.0 ± 7.2	IPC	30 mL, 1 time/w, 4 times	5 mg	8 weeks	Ostrowskimj, Un	O1,3
CKI and interleukin-2 versus interleukin-2 (two trials)														
Hao and Liang (2007)	MTs	Un	Un	Un	Un	26/21	33/14	45–83	IPC	20 mL, 1 time/w, 3 times	2MU	7 weeks	Millar, WHO	O1,3
Zhou et al. (2010)	LC	Small to large	>40	PT	>3	30/30	42/18	60–85	IPC	30 mL, 1 time/w, 4 times	2MU	8 weeks	Ostrowskimj, WHO	O1,3
CKI and OK-432 versus OK-432 (two trials)														
Wei et al. (2014)	MTs	Un	Un	Un	Un	40/40	45/35	Un	IPC	20 mL, 3 time/w, 3 times	d1:5 KE, d4,d7:10 KE	Un	Ostrowskimj, Un	O1,3
Zhong et al. (2015)	MTs	Un	>40	Un	Un	44/44	49/39	Un	IPC	20 mL, 3 time/w, 3 times	d1:5 KE, d4,d7:10 KE	5 weeks	Ostrowskimj, Un	O1,3
CKI and mitomycin versus mitomycin (one trial)														
Zhang et al. (2013)	MTs	Moderate to large	>40	Un	>3	60/60	67/53	49–76	IPC	40 mL, 1 time/w, 3 times	10 mg	7 weeks	Ostrowskimj, Un	O1-3
CKI and *Corynebacterium parvum* versus *C. parvum* (one trial)														
Huang et al. (2012)	LC	moderate to large	>50	PT	>3	45/45	47/43	40–77	IPC	30 mL, 1 time/w, 4 times	4 mL (24*10^9^)	8 weeks	Millar, WHO	O1-3
c. Intrapleural administration with *kang’ai* injection (*kang’ai*)														
*Kang’ai* and cisplatin versus cisplatin (six trials)														
Zhang (2006)	MTs	Un	≥60	Un	Un	20/21	19/22	36–72	IPC	60 mL, 1 time/w, 1–3 times	80 mg	7 weeks	Ostrowskimj, WHO	O1,3
Hu et al. (2008a)	LC	Large	>50	Un	>1	36/35	43/28	45–80	IPC	60 mL, 1 time/w, 2–4 times	40 mg/m^2^	6–8 weeks	Ostrowskimj, WHO	O1,3
Xu and Xiong (2008)	MTs	Un	≥50	Un	>3	33/33	44/22	56 ± 4.7	IPC	40 mL, 2 times/w, 4 times	60 mg	6 weeks	Ostrowskimj, Un	O1-3
He (2011)	MTs	Un	≥70	Un	Un	20/20	24/16	45–72	Un	60 mL, 1 time/w, 1–3 times	80 mg	6–10 weeks	Millar, WHO	O1,3,4
Qu et al. (2012)	LC	Moderate to large	>60	PT	>3	24/22	27/19	46–84	IPC	50 mL,1 time/w, 3 times	40–60 mg	8 weeks	Ostrowskimj, Un	O1-3
Wang (2016)	LC	Large	Un	Un	>1	35/35	44/26	64.5 ± 8.7	IPC	60 mL, 1 time/w, Un	40 mg/m^2^	Un	Ostrowskimj, Un	O1
*Kang’ai* and carboplatin versus carboplatin (one trial)														
Chen (2009)	MTs	Un	≥50	Un	Un	25/23	26/22	53–82	IPC	60 mL, 1 time/week, 2–4 times	300 mg	6–8 weeks	Ostrowskimj, WHO	O1,3
d. Intrapleural administration with matrine injection (matrine)														
Matrine and cisplatin versus cisplatin (six trials)														
Du et al. (2009)	MTs	Un	Un	Un	Un	40/36	39/37	39–78	IPC	200 mg, 1 time/w, 3-6 times	20 mg	7–10 weeks	Millar, Un	O1,3
Li and Yang (2009)	MTs	Moderate to large	Un	Un	>2	30/30	Un	47–73	Thora*	500 mg, 1 time/w, 4 times (40 mg)	60 mg	8 weeks	Ostrowskimj, Un	O1,3
He (2010)	MTs	Un	>50	Un	>3	47/36	38/45	30–70	IPC	800 mg, 1 time/w, 3 times	30 mg/m^2^	10 weeks	Ostrowskimj, WHO	O1,3
Wang et al. (2010)	MTs	Un	≥60	Un	>3	20/20	Un	32–76	IPC	150 mg, 1 time/w, 3 times	60 mg	8 weeks	Ostrowskimj, WHO	O1-3
Ji (2011)	MTs	Un	>50	Un	>3	30/30	27/33	33–74	Thora*	200 mg, 1 time/w, 4 times (40 mg)	60 mg	8 weeks	Ostrowskimj, WHO	O1-3
Ji et al. (2012)	MTs	Un	Un	Un	Un	82/70	90/62	35–85	IPC	200 mg, 1 time/w, 2 times	40 mg	8–10 weeks	Millar, Un	O1
Matrine and carboplatin versus carboplatin (one trial)														
Cui et al. (2008)	MTs	Un	Un	Un	Un	40/38	41/37	35–76	Un	200 mg, 1 time/w, 3–6 times	50–100 mg	7–10 weeks	Millar, Un	O1,3,4

Note: MTs, miscellaneous tumors; LC, lung cancer; BC, breast cancer; HM, hematologic malignancies; KPS, Karnofsky performance status score; TH, treatment history; AST, anticipated survival time; PT, primary treatment; RT: retreatment; *Kushen*, radix *Sophorae flavescentis* preparations; E/C, experimental group (*kushen* alone or with sclerosants)/control group (sclerosants alone); M/F, male/female; DM, drainage method; IPC, indwelling pleural catheter; Thora*, thoracentesis; MU, million units; KE, *Klinische Einheit*; O, outcome, O1, clinical responses; O2, quality of life (QOL); O3, adverse events; O4, long-term survival; Un, unclear.

Of 83 eligible studies, 58 (69.88%, 58/83) involved inpatients with miscellaneous tumors, 24 (28.92%, 24/83) with lung cancer, and only one with hematologic malignancies (Huang, 2013) or breast cancer (Wang R. et al., 2023). Most studies described demographic characteristics, but only 16 to 50 (19.28%, 16/83% to 60.24%, 50/83) reported the pleural fluid volume, KPS, AST, and treatment history. All studies reported the drainage methods and characteristics of interventions and assessed the clinical responses 5–10 weeks after treatment began using Ostrowskimj or Millar criteria. Only 36 studies (43.37%, 36/83) reported the QOL, and six reported overall survival (Cui et al., 2008; Chen, 2010; He, 2011; Han, 2013; Zhang S. et al., 2015). Some 79 studies (95.18%, 79/83) reported the AEs, 38 (45.78%, 38/83) assessed ADRs using WHO or CTEC3.0 criteria, and only four assessed TRAEs (Wang et al., 2010; Yang, 2012; Wei et al., 2014; Liu and Li, 2015; Song and Jia, 2015). No study reported conflicts of interest.

### 3.3 Methodological quality

**FIGURE 3:**
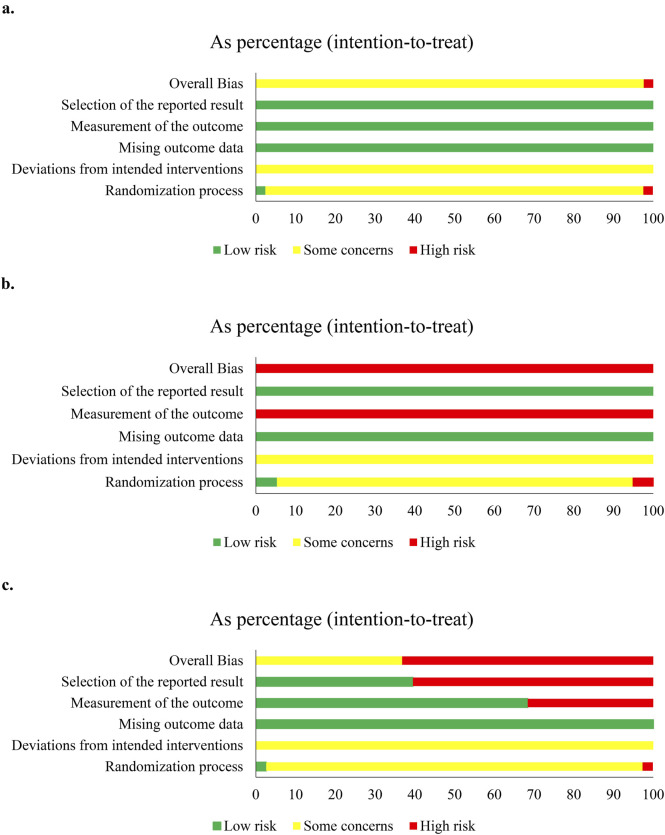
Risk-of-bias of compound *kushen* injection and cisplatin. **(a)** Clinical responses; **(b)** quality of life; **(c)** adverse events.

Of 83 studies, 79 (95.18%, 79/83) expressed concerns at overall bias for clinical responses, and four showed high risk (Qu et al., 2012; Wu et al., 2014; Wang and Zhou, 2016; Lin et al., 2023). At domain-level, only one study had low risk at D1 (Liu and Li, 2015), one showed high risk at D1 (Lin et al., 2023) or D2 (Wang and Zhou, 2016), and others had some concerns. All had low risk at D3 and D4. Two studies showed high risk at D5 (Qu et al., 2012; Wu et al., 2014), and others had low risk (Figure 3A; Supplementary Figures S1, S2). For overall survival, five studies had concerns of overall bias (Cui et al., 2008; Chen, 2010; He, 2011; Han, 2013; Zhang S. et al., 2015). All had some concerns at D1 and D2, and low risk at D3, D4, and D5 (Supplementary Figure S4).

Since studies were limited, we only assessed the methodological quality of QOL and adverse events in CKI versus cisplatin, and perfusion with CKI, *kang’ai*, or matrine and cisplatin. QOL was reported by 29 studies and showed high risk at overall bias. Only one study (Liu and Li, 2015) had low risk, and one (Lin et al., 2023) had high risk at D1. All showed some concern at D2, low risk at D3 and D5, and high risk at D4 (Figure 3B and S3). A total of 57 studies reported AEs, 35 (61.40%, 35/57) showed high risk at overall bias, and 21 had some concerns. There were 55 studies (96.49%, 55/57) with some concerns at D1and D2, two with low risk at D1 (Liu and Li, 2015; Lin et al., 2023), and one with high risk at D2 (Wang and Zhou, 2016). All studies had low risk at D3. High risk was shown by 16 studies (28.07%, 16/57), and 39 had low risk at D4. A total of 34 studies (59.65%, 34/57) showed high risk, and 21 had low risk at D5 (Figure 3C and Figure. S5).

### 3.4 Clinical responses

Nine trials reported clinical responses about CKI versus cisplatin (Table 2a; Supplementary Figures S6–S8). Cochran’s χ^2^ test and *I* ^2^ statistic revealed no heterogeneity (*I* ^2^ = 0%). We pooled the OR using a FEM. The results of meta-analyses revealed that CKI perfusion displayed a complete response (1.10, 95% CI 0.76 to 1.60), pleurodesis failure (0.80, 95% CI 0.56 to 1.14), and pleural progression (0.63, 95% CI 0.33 to 1.21) similar to cisplatin alone. Only single trial reported that CKI achieved clinical response similar to mitomycin and better than interleukin-2.

**TABLE 2:** Meta-analysis results of clinical responses.

Outcomes	Trials	*Kushen* preparations (events/total)	Sclerosants (events/total)	Statistical method	Odds ratios 95% CI	*I* ^*2*^	*P*
a. Compound *kushen* injection (CKI) alone (Supplementary Figures S6–S8)							
CKI versus cisplatin							
Complete response	9	87/281	85/287	Fixed-effects model	1.10 [0.76, 1.60]	0%	p = 0.60
Pleurodesis failure	9	87/281	103/287	Fixed-effects model	0.80 [0.56, 1.14]	0%	p = 0.21
Pleural progression	6	18/175	26/173	Fixed-effects model	0.63 [0.33, 1.21]	0%	p = 0.17
CKI versus interleukin-2							
Complete response	1	45/65	31/63	Not applicable	2.32 [1.13, 4.78]	No	*p* = 0.02
Pleurodesis failure	1	9/65	25/63	Not applicable	0.24 [0.10, 0.58]	No	*p* = 0.001
CKI versus mitomycin							
Complete response	1	16/50	15/50	Not applicable	1.10 [0.47, 2.56]	No	*p* = 0.83
Pleurodesis failure	1	22/50	21/50	Not applicable	1.09 [0.49, 2.40]	No	*p* = 0.84
b. CKI and sclerosants (Figures 4A–C)							
CKI and cisplatin versus cisplatin							
Complete response	41	649/1,424	342/1,399	Fixed-effects model	2.71 [2.30, 3.19]	0%	p < 0.00001
Pleurodesis failure	41	235/1,424	590/1,399	Fixed-effects model	0.26 [0.22, 0.32]	0%	p < 0.00001
Pleural progression	13	25/481	90/475	Fixed-effects model	0.22 [0.14, 0.36]	0%	p < 0.00001
CKI and nedaplatin versus nedaplatin							
Complete response	3	44/129	30/129	Fixed-effects model	1.72 [0.99, 2.98]	0%	*p* = 0.05
Pleurodesis failure	3	28/129	58/129	Fixed-effects model	0.33 [0.19, 0.57]	0%	*p* < 0.0001
CKI and lobaplatin versus lobaplatin							
Complete response	2	26/55	20/55	Fixed-effects model	1.57 [0.73, 3.36]	44%	p = 0.25
Pleurodesis failure	2	9/55	18/55	Fixed-effects model	0.35 [0.13, 0.93]	0%	p = 0.04
Pleural progression	1	1/25	1/25	Not applicable	0.11 [0.01, 0.95]	No	p = 0.04
CKI and bleomycin versus bleomycin							
Complete response	3	33/77	16/69	Fixed-effects model	2.62 [1.23, 5.58]	0%	p = 0.01
Pleurodesis failure	3	12/77	30/69	Fixed-effects model	0.23 [0.11, 0.52]	0%	p = 0.0004
CKI and hydroxycamptothecin versus hydroxycamptothecin							
Complete response	2	41/78	21/78	Fixed-effects model	3.01 [1.54, 5.87]	0%	p = 0.001
Pleurodesis failure	3	15/120	33/118	Fixed-effects model	0.37 [0.19, 0.72]	0%	p = 0.004
CKI and interleukin-2 versus interleukin-2							
Complete response	2	29/56	13/51	Fixed-effects model	3.21 [1.41, 7.34]	0%	p = 0.006
Pleurodesis failure	2	9/56	22/51	Fixed-effects model	0.24 [0.10, 0.60]	0%	p = 0.002
Pleural progression	1	0/26	4/21	Not applicable	0.07 [0.00, 1.45]	No	p = 0.09
CKI and OK-432 versus OK-432							
Complete response	2	24/84	17/84	Fixed-effects model	1.58 [0.77, 3.21]	0%	p = 0.21
Pleurodesis failure	2	24/84	17/84	Fixed-effects model	0.32 [0.16, 0.67]	0%	p = 0.002
CKI and mitomycin versus mitomycin							
Complete response	1	14/60	13/60	Not applicable	1.10 [0.47, 2.59]	No	p = 0.83
Pleurodesis failure	1	11/60	21/60	Not applicable	0.42 [0.18, 0.97]	No	p = 0.04
CKI and carboplatin versus carboplatin							
Complete response	1	11/21	6/20	Not applicable	2.57 [0.71, 9.27]	No	p = 0.15
Pleurodesis failure	1	3/21	9/20	Not applicable	0.20 [0.05, 0.92]	No	p = 0.04
Pleural progression	1	0/21	3/20	Not applicable	0.12 [0.01, 2.41]	No	p = 0.16
CKI and *Corynebacterium parvum* versus *C. parvum*							
Complete response	1	18/45	13/45	Not applicable	1.64 [0.68, 3.95]	No	p = 0.27
Pleurodesis failure	1	4/45	16/45	Not applicable	0.18 [0.05, 0.58]	No	p = 0.004
c. *Kang’ai* injection (Supplementary Figures S9–S11)							
*Kang’ai* and cisplatin versus cisplatin							
Complete response	5	56/144	25/144	Fixed-effects model	3.04 [1.76, 5.26]	0%	*p* < 0.0001
Pleurodesis failure	6	26/168	69/166	Fixed-effects model	0.23 [0.14, 0.41]	0%	P < 0.00001
Pleural progression	1	3/20	8/20	Not applicable	0.26 [0.06, 1.21]	No	p = 0.09
*Kang’ai* and carboplatin versus carboplatin (One trial)							
Complete response	1	9/25	6/23	Not applicable	1.59 [0.46, 5.50]	No	p = 0.46
Pleurodesis failure	1	4/25	8/23	Not applicable	0.36 [0.09, 1.41]	No	p = 0.14
d. Matrine injection (Supplementary Figures S9–S11)							
Matrine and cisplatin versus cisplatin (six trials)							
Complete response	6	106/249	66/222	Fixed-effects model	1.87 [1.26, 2.78]	0%	p = 0.002
Pleurodesis failure	6	32/249	74/222	Fixed-effects model	0.27 [0.17, 0.44]	0%	P < 0.00001
Pleural progression	2	4/122	11/106	Fixed-effects model	0.29 [0.09, 0.95]	0%	p = 0.04
Matrine and carboplatin versus carboplatin							
Complete response	1	23/40	16/38	Not applicable	1.86 [0.76, 4.57]	No	p = 0.18
Pleurodesis failure	1	4/40	12/38	Not applicable	0.24 [0.07, 0.83]	No	p = 0.02
Pleural progression	1	1/40	6/38	Not applicable	0.14 [0.02, 1.20]	No	p = 0.07

Note: CI: confidence interval.

**FIGURE 4:**
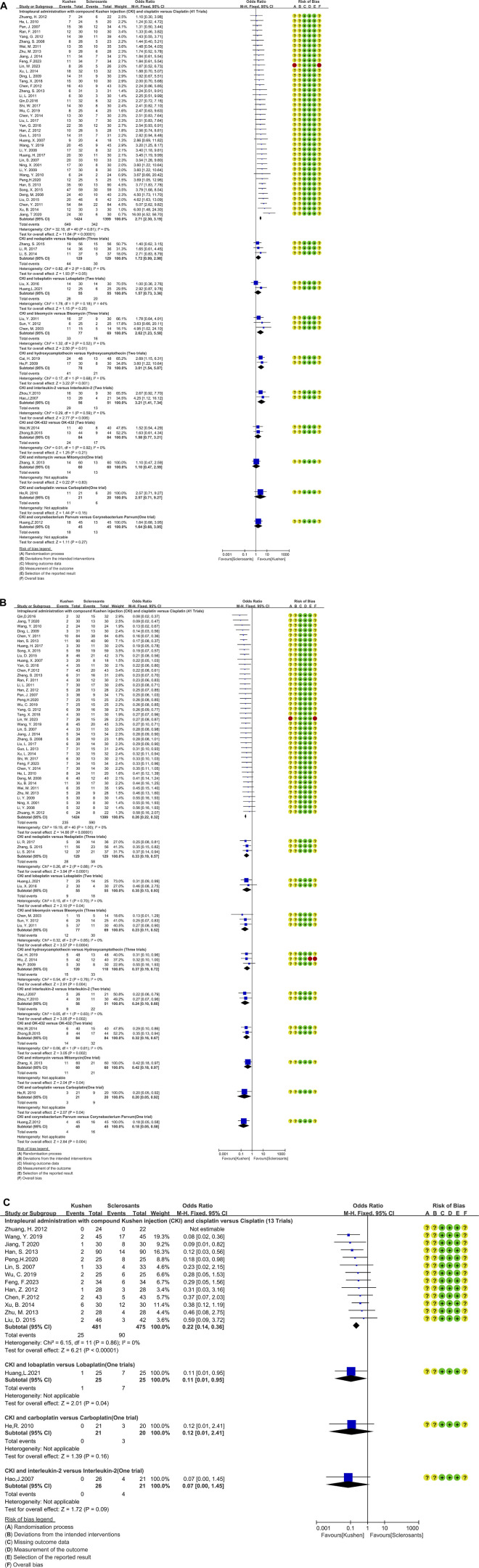
Clinical responses of compound *kushen* injection in MPE. **(A)** Meta-analysis of complete response; **(B)** meta-analysis of pleurodesis failure; **(C)** forest plot of pleural progression.

**FIGURE 5:**
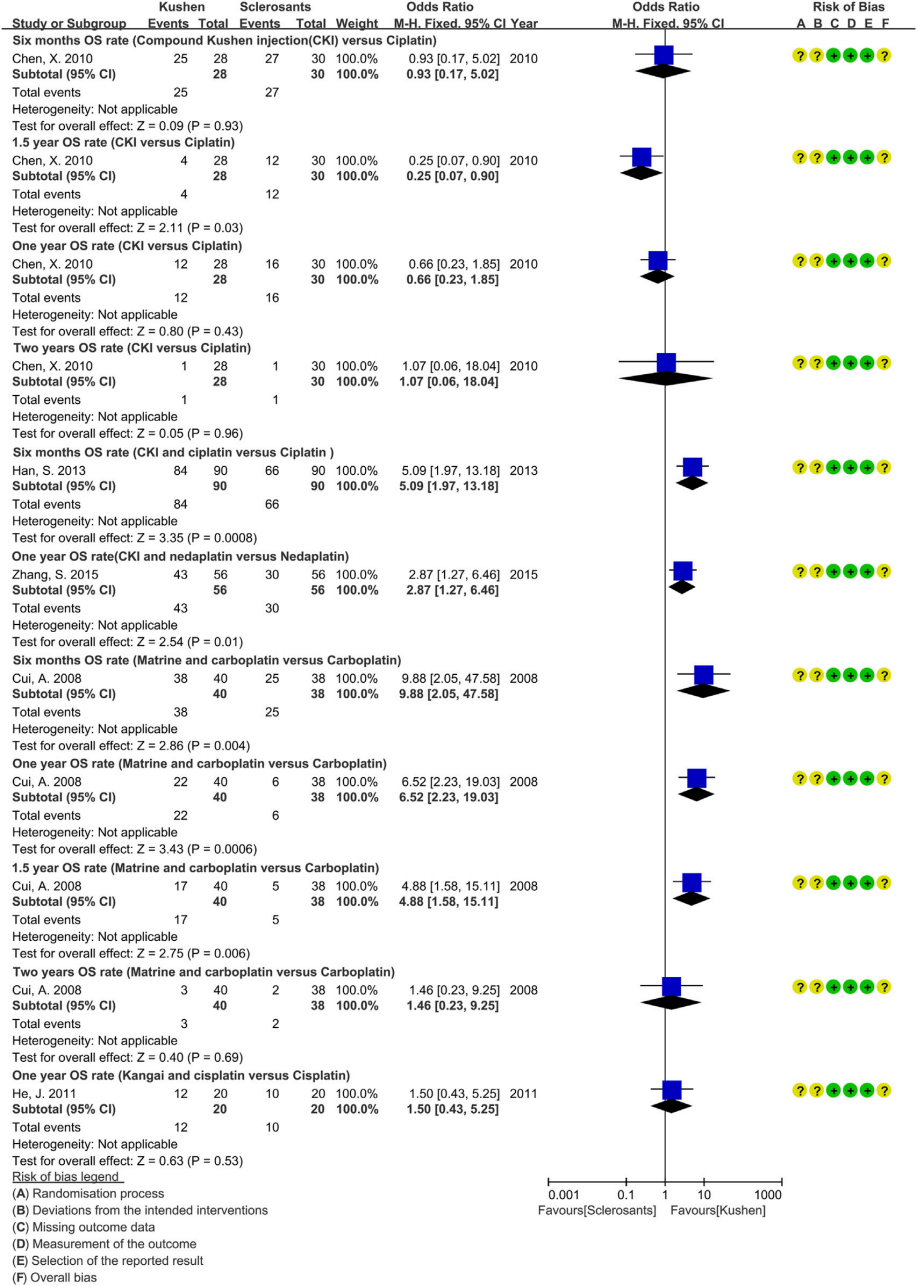
Forest plot of overall survivals.

The CKI and chemical drug or BRM developed ten treatment plans (Table 2b; Figure 4C; Figure 5). Perfusion with CKI and cisplatin was evaluated by 41 trials. With no statistical heterogeneity (*I* ^2^ = 0%), an FEM was used to pool the OR. The results demonstrated it significantly improving the complete response (2.71, 95% CI 2.30 to 3.19) and displaying a low pleurodesis failure (0.26, 95% CI 0.22 to 0.32) and pleural progression (0.22, 95% CI 0.14–0.36) than cisplatin alone. One to three trials reported nine other treatment plans. Compared with sclerosants alone, the results revealed that nine treatment plans achieved a low pleurodesis failure, while only CKI and bleomycin, hydroxycamptothecin, or interleukin-2 significantly improved the complete response.

*Kang’ai* or matrine and platinum developed four treatment plans (Table 2c, 2d; Supplementary Figures S9–S11). With no statistical heterogeneity (*I* ^2^ = 0%), an FEM was used. The results demonstrated that perfusion with *kang’ai* or matrine and cisplatin significantly improved the complete response (3.04, 95% CI 1.76 to 5.26 and 1.87, 95% CI 1.26–2.78) and achieved a low pleurodesis failure (0.23, 95% CI 0.14 to 0.41 and 0.27, 95% CI 0.17–0.44) than cisplatin alone. Additionally, matrine and cisplatin achieved a low pleural progression (0.29, 95% CI 0.09–0.95).

### 3.5 Overall survivals

Of 83 studies, only six (Cui et al., 2008; Chen, 2010; Chen et al., 2011; He, 2011; Han, 2013; Zhang S. et al., 2015) reported the OS of perfusion with CKI alone, CKI and cisplatin or nedaplatin, *kang’ai* and cisplatin, or matrine and carboplatin (Figure 5). Compared with sclerosants alone, only one trial reported that perfusion with CKI and cisplatin might improve the 0.5-year OS rate (Han, 2013), and it might prolong median survival time and PFS (Chen et al., 2011). Perfusion with CKI and nedaplatin might improve the 1-year OS rate (Zhang S. et al., 2015), and matrine and carboplatin might improve the 0.5-year, 1-year, and 1.5-year OS rates (Cui et al., 2008).

### 3.6 Quality of life

Due to limited trials, we only assessed the QOL of perfusion with CKI alone, CKI, *kang’ai*, or matrine and cisplatin (Table 3; Supplementary Figures S12, S13). Six trials reported the QOL about CKI alone (Yuan, 2007; Hu Q. et al., 2008; Liang et al., 2011; Chen, 2013; Xing, 2013; Yan et al., 2016). Statistical heterogeneity (*I* ^2^ = 67%) was found, and an REM was used. Compared with cisplatin alone, CKI perfusion acquired a similar QOL. There were 21 trials reporting QOL about perfusion with CKI, *kang’ai*, or matrine and cisplatin. No heterogeneity was found (*I* ^2^ = 0%), and an FEM was used to pool the OR. Compared with cisplatin alone, the results demonstrated that perfusion with CKI, *kang’ai* or matrine and cisplatin significantly improved QOL (3.60, 95% CI 2.84 to 4.56; 3.95, 95% CI 1.78 to 8.74 and 2.95, 95% CI 1.25–6.97).

**TABLE 3:** Meta-analysis results of quality of life and adverse events (Supplementary Figures S12–S24).

Outcomes	Trials	*Kushen* preparations with sclerosants (events/total)	Sclerosants (events/total)	Statistical method	Odds ratios 95% CI	*I* ^*2*^	*P*
a. Compound kushen injection (CKI) versus cisplatin							
Quality of life (Supplementary Figure S12)	6	144/207	127/212	Random-effects model	1.52 [0.69, 3.35]	67%	*p* = 0.30
Myelosuppression (Supplementary Figure S14)	6	3/193	109/197	Random-effects model	0.02 [0.00, 0.15]	69%	*p < 0.0001*
Leukopenia (Supplementary Figure S15)	3	2/88	22/90	Fixed-effects model	0.10 [0.03, 0.35]	0%	*p* = 0.0003
Gastrointestinal reaction (Supplementary Figure S18)	9	10/281	167/287	Random-effects model	0.03 [0.01, 0.12]	67%	*p < 0.00001*
Hepatotoxicity (Supplementary Figure S19)	6	1/201	22/197	Fixed-effects model	0.09 [0.02, 0.33]	0%	*p* = 0.0003
Nephrotoxicity (Supplementary Figure S20)	7	1/221	26/217	Fixed-effects model	0.09 [0.03, 0.29]	0%	*p < 0.0001*
Cardiotoxicity (Supplementary Figure S21)	1	0/30	0/30	Not applicable	Not estimable	No	No
Thoracodynia (Supplementary Figure S22)	6	22/175	74/173	Random-effects model	0.15 [0.04, 0.48]	69%	p = 0.002
Fever (Supplementary Figure S23)	5	29/159	31/158	Random-effects model	0.67 [0.12, 3.76]	81%	*p* = 0.65
b. CKI and cisplatin versus cisplatin							
Quality of life (Supplementary Figure S12)	19	497/682	298/670	Fixed-effects model	3.60 [2.84, 4.56]	0%	p < 0.00001
Myelosuppression (Supplementary Figure S14)	17	149/574	229/558	Fixed-effects model	0.34 [0.24, 0.47]	0%	*p* < 0.00001
Leukopenia (Supplementary Figure S15)	20	178/711	291/703	Fixed-effects model	0.35 [0.26, 0.46]	0%	*p* < 0.00001
Anemia (Supplementary Figure S16)	2	5/120	7/118	Fixed-effects model	0.69 [0.21, 2.24]	0%	*p* = 0.54
Thrombocytopenia (Supplementary Figure S17)	5	7/215	9/213	Fixed-effects model	0.76 [0.27, 2.12]	0%	*p* = 0.61
Gastrointestinal reaction (Supplementary Figure S18)	31	254/1,053	440/1,035	Fixed-effects model	0.36 [0.29, 0.44]	8%	*p* < 0.00001
Hepatotoxicity (Supplementary Figure S19)	22	43/837	87/824	Fixed-effects model	0.42 [0.28, 0.63]	0%	*p* < 0.0001
Nephrotoxicity (Supplementary Figure S20)	31	75/1,105	169/1,090	Fixed-effects model	0.32 [0.24, 0.44]	0%	p < 0.00001
Cardiotoxicity (Supplementary Figure S21)	5	0/152	0/151	Not applicable	Not estimable	No	No
Thoracodynia (Supplementary Figure S22)	11	49/402	66/394	Fixed-effects model	0.65 [0.42, 1.00]	0%	*p* = 0.05
Fever (Supplementary Figure S23)	26	65/853	97/828	Fixed-effects model	0.50 [0.30, 0.82]	0%	*p* = 0.006
TRAEs (Supplementary Figure S19)	3	0/144	0/140	Not applicable	Not estimable	No	No
c. *Kang’ai* and cisplatin versus cisplatin							
Quality of life (Supplementary Figure S13)	2	34/57	15/55	Fixed-effects model	3.95 [1.78, 8.74]	0%	p = 0.0007
Leukopenia (Supplementary Figure S15)	4	31/113	67/110	Fixed-effects model	0.20 [0.11, 0.38]	25%	*p* < 0.0001
Gastrointestinal reaction (Supplementary Figure S18)	5	42/133	65/131	Fixed-effects model	0.34 [0.19, 0.63]	0%	*p* = 0.0006
Hepatotoxicity (Supplementary Figure S19)	1	0/24	0/22	Not applicable	Not estimable	No	No
Nephrotoxicity (Supplementary Figure S20)	2	0/57	0/55	Not applicable	Not estimable	No	No
Thoracodynia (Supplementary Figure S22)	2	5/60	10/57	Fixed-effects model	0.41 [0.13, 1.29]	0%	*p* = 0.13
Fever (Supplementary Figure S23)	1	2/36	2/35	Not applicable	0.97 [0.13, 7.30]	No	*p* = 0.98
TRAEs (Supplementary Figure S24)	1	0/24	0/22	Not applicable	Not estimable	No	No
d. Matrine and cisplatin versus cisplatin							
Quality of life (Supplementary Figure S13)	2	32/50	20/50	Fixed-effects model	2.95 [1.25, 6.97]	0%	p = 0.02
Myelosuppression (Supplementary Figure S14)	3	14/97	19/86	Fixed-effects model	0.49 [0.21, 1.11]	43%	P = 0.09
Leukopenia (Supplementary Figure S15)	2	7/70	31/66	Random-effects model	0.10 [0.02, 0.61]	66%	P = 0.01
Anemia (Supplementary Figure S16)	1	14/30	26/30	Not applicable	0.13 [0.04, 0.48]	No	*p* = 0.002
Thrombocytopenia (Supplementary Figure S17)	1	8/30	9/30	Not applicable	0.85 [0.28, 2.61]	No	*p* = 0.77
Gastrointestinal reaction (Supplementary Figure S18)	5	36/167	55/152	Fixed-effects model	0.35 [0.19, 0.66]	0%	*p* = 0.001
Hepatotoxicity (Supplementary Figure S19)	3	15/117	22/102	Fixed-effects model	0.52 [0.23, 1.15]	0%	*p* = 0.10
Nephrotoxicity (Supplementary Figure S20)	4	7/137	11/122	Fixed-effects model	0.56 [0.19, 1.59]	0%	*p* = 0.27
Cardiotoxicity (Supplementary Figure S21)	1	0/20	0/20	Not applicable	Not estimable	No	No
Thoracodynia (Supplementary Figure S22)	4	9/120	31/116	Fixed-effects model	0.21 [0.10, 0.48]	0%	*p* = 0.0002
Fever (Supplementary Figure S23)	4	7/147	15/132	Fixed-effects model	0.41 [0.16, 1.07]	0%	*p* = 0.07
TRAEs (Supplementary Figure S24)	1	0/20	0/20	Not applicable	Not estimable	No	No

Note: CI, confidence interval. TRAEs, thoracentesis-related adverse events.

### 3.7 Adverse events

Nine trials reported eight AEs about CKI alone (Table 3a; Supplementary Figures S14, S15, S18–S23). Cochran’s χ^2^ test and *I* ^2^ statistic only identified statistical heterogeneity for myelosuppression (I^2^ = 69%), gastrointestinal reaction (I^2^ = 67%), thoracodynia (I^2^ = 69%), and fever (I^2^ = 81%), and an REM or FEM was used to synthesize the OR. Compared with cisplatin alone, meta-analysis revealed that perfusion with CKI alone showed a low myelosuppression (0.02, 95% CI 0.00 to 0.15), leukopenia (0.10, 95% CI 0.03–0.35), gastrointestinal reaction (0.03, 95% CI 0.01 to 0.12), hepatotoxicity (0.09, 95% 0.02–0.33), nephrotoxicity (0.09, 95% CI 0.03 to 0.29), and thoracodynia (0.15, 95% CI 0.04 to 0.48).

Ten AEs were reported by 38 trials about CKI and cisplatin (Table 3; Supplementary Figures S14–S23). We only identified minimal heterogeneity for gastrointestinal reaction (I^2^ = 8%), and an FEM was used. The results demonstrated that perfusion with CKI and cisplatin showed a low myelosuppression (0.34, 95% CI 0.24–0.47), neutropenia (0.35, 95% CI 0.26 to 0.46), gastrointestinal reaction (0.36, 95% CI 0.29–0.44), and hepatorenal toxicity (0.42, 95% CI 0.28 to 0.63 and 0.32, 95% CI 0.24–0.44) and fever (0.50, 95% CI 0.30–0.82).

Five trials reported six AEs to *kang’ai* and cisplatin (Table 3c; Supplementary Figures S15–S24). We only identified minimal heterogeneity leukopenia (*I* ^2^ = 25%), and an FEM was used. The results revealed that *kang’ai* and cisplatin showed low neutropenia (OR = 0.20, 95% CI 0.11–0.38) and gastrointestinal reaction (OR = 0.34, 95% CI 0.19–0.63).

Five trials reported ten AEs to matrine and cisplatin (Table 3d; Supplementary Figures S14–S23). We only identified statistical heterogeneity for neutropenia (*I* ^2^ = 66%) and minimal heterogeneity for myelosuppression (*I* ^2^ = 43%), and an REM or FEM was used. The results revealed that matrine and cisplatin showed low neutropenia (0.10, 95% CI 0.02–0.61), gastrointestinal reaction (0.35, 95% CI 0.19–0.66), and thoracodynia (0.21, 95% CI 0.10–0.48).

### 3.8 Subgroup analysis

In targeting perfusion with CKI and cisplatin, subgroup analysis revealed that under different primary tumors, drainage, and evaluation criteria, this treatment plan obtained significant improvement in the complete response and low pleurodesis failure. For patients with moderate-to-large effusion, KPS ≥50 to ≥70 scores, AST ≥3 months, or primary treatment, it significantly improved clinical responses. Perfusion with CKI (20–50 mL/time, once per week, two to four times) and cisplatin (20–80 mg/time) could significantly improve the clinical responses. Moreover, perfusion with low-dosage cisplatin and CKI could obtain clinical responses like high-dosage. However, the univariate regression and multiple meta-regression analysis did not reveal any correlation between clinical response and each variable (Table 4; Supplementary Figures S25–S72).

**TABLE 4:** Subgroups and meta-regression analysis (Supplementary Figures S25–S72).

Subgroups	Trials	Cases	Complete response			Pleurodesis failure		
			Odds ratios (95%CI)	Univariable*	Multiple*	Odds ratios (95%CI)	Univariable*	Multiple*
a. Subgroups analysis via primary disease (Supplementary Figures S25–S28)								
Miscellaneous tumors	28	2053	2.77 [2.29, 3.37]	0.69	0.65	0.25 [0.20, 0.31]	0.89	0.89
Lung cancer	12	724	2.68 [1.93, 3.72]			0.29 [0.21, 0.40]		
Hematologic malignancies	1	46	1.10 [0.30, 3.98]			0.58 [0.16, 2.07]		
b Subgroup analysis via pleural effusion (Supplementary Figures S29–S32)								
Small to large	1	52	1.87 [0.52, 6.73]	0.12	0.28	0.47 [0.23, 0.95]	0.49	0.71
Moderate to large	10	640	2.15 [1.51, 3.05]			0.30 [0.21, 0.43]		
Large	3	158	2.32 [1.20, 4.50]			0.28 [0.13, 0.59]		
Unclear	27	1973	2.98 [2.45, 3.63]			0.25 [0.20, 0.31]		
c. Subgroups analysis via Karnofsky performance status score (Supplementary Figures S33–S36)								
Karnofsky performance status score (≥50)	7	404	2.24 [1.44, 3.49]	0.94	0.38	0.29 [0.19, 0.45]	0.15	0.55
Karnofsky performance status score (≥60)	15	1,195	2.67 [2.07, 3.44]			0.22 [0.17, 0.29]		
Karnofsky performance status score (≥70)	2	174	2.97 [1.52, 5.79]			0.27 [0.12, 0.61]		
Unclear	17	1,050	2.91 [2.22, 3.80]			0.31 [0.23, 0.41]		
d. Subgroup analysis via anticipated survival time (Supplementary Figures S37–S40)								
Anticipated survival time (unclear)	26	1803	2.83 [2.31, 3.47]	0.74	0.77	0.28 [0.23, 0.35]	0.53	0.52
Anticipated survival time (≥3 months)	13	922	2.41 [1.79, 3.25]			0.22 [0.16, 0.31]		
Anticipated survival time (≥1 months)	2	98	3.30 [1.40, 7.82]			0.38 [0.15, 0.99]		
e. Subgroup analysis via treatment history (Supplementary Figures S41–S44)								
Primary treatment	9	549	2.49 [1.71, 3.63]	0.23	0.88	0.28 [0.19, 0.41]	0.47	0.60
Retreatment	2	112	1.57 [0.67, 3.67]			0.36 [0.16, 0.81]		
Others	30	2,162	2.84 [2.35, 3.43]			0.25 [0.21, 0.31]		
f. Subgroup analysis via the drainage method (Supplementary Figures S45–S48)								
Indwelling pleural catheter	33	2,349	2.63 [2.19, 3.16]	0.68	0.76	0.24 [0.20, 0.29]	0.04	0.08
Thoracentesis	8	474	3.09 [2.09, 4.56]			0.40 [0.26, 0.62]		
g. Subgroups analysis via CKI dosage (Supplementary Figures S49–S52)								
Compound *kushen* injection (20–30 mL)	31	2045	2.60 [2.15, 3.16]	0.72	0.61	0.27 [0.22, 0.33]	0.92	0.90
Compound *kushen* injection (40–60 mL)	8	676	3.27 [2.32, 4.59]			0.24 [0.17, 0.34]		
Compound *kushen* injection (others)	2	102	1.71 [0.71, 4.13]			0.37 [0.16, 0.89]		
h. Subgroups analysis via treatment frequency (Supplementary Figures S53–S56)								
One time/week	35	2,493	2.62 [2.20, 3.11]	0.25	0.67	0.27 [0.23, 0.33]	0.34	0.96
Others (1–2 times/week or 2–3 time/week)	6	330	3.66 [2.18, 6.14]			0.20 [0.12, 0.34]		
i Subgroups analysis via treatment times (Supplementary Figures S57–S60)								
Two to four times	25	1783	2.49 [2.02, 3.06]	0.27	0.59	0.27 [0.22, 0.34]	0.76	0.46
Others (>4 times or unclear)	16	1,040	3.13 [2.39, 4.11]			0.25 [0.19, 0.34]		
j Subgroup analysis via cisplatin dosage (Supplementary Figures S61–S64)								
Cisplatin (20–30 mg each time)	6	450	2.47 [1.60, 3.83]	0.31	0.62	0.23 [0.15, 0.36]	0.92	0.93
Cisplatin 40–50 mg each time)	18	1,119	2.55 [1.98, 3.29]			0.28 [0.21, 0.37]		
Cisplatin (60–80 mg each time)	11	724	2.71 [1.95, 3.77]			0.31 [0.22, 0.44]		
Cisplatin (others)	6	530	3.31 [2.25, 4.85]			0.22 [0.15, 0.33]		
k Subgroups analysis via dosage difference of cisplatin (Supplementary Figures S65–S68)								
Equivalent dosage	35	2,439	2.58 [2.16, 3.08]	0.33	0.43	0.26 [0.21, 0.31]	0.49	0.95
Low vs. high dosage	6	384	3.64 [2.34, 5.67]			0.31 [0.19, 0.50]		
l Subgroups analysis via criterion (Supplementary Figures S69–S72)								
Millar	28	1867	2.49 [2.04, 3.04]	0.16	0.18	0.27 [0.21, 0.33]	0.88	0.86
Ostrowskimj	13	956	3.23 [2.40, 4.33]			0.26 [0.19, 0.35]		

Note: Others: unclear or ungroupable; CI: confidence interval. Univariable*: univariable meta-regression (P>|t|); Multiple*: multiple meta-regression (P>|t|).

### 3.9 Publication bias analysis

Only perfusion with CKI and cisplatin was included in more than ten trials (Table 5; Supplementary Figures S73–S83). The funnel plot and Egger’s test did not identify publication bias for the complete response, pleurodesis failure, pleural progression, myelosuppression, neutropenia, hepatorenal toxicity, thoracodynia, and fever, which were objectively reported. Significant publication bias was identified for QOL (coefficient = –2.47, 95% CI –4.62 to –0.32) and gastrointestinal reaction (coefficient = –1.49, 5% CI –2.71 to –0.21); both results were under-estimated.

**TABLE 5:** Publication bias risk (Supplementary Figures S73–S83).

Indicators	Trials	Compound *kushen* injection (CKI) and cisplatin (events/total)	Cisplatin (events/total)	OR (95% CI)	Egger’s test			Risk assessment
					Coefficient	95% CI	P>|t|	
Complete response	41	649/1,424	342/1,399	2.71 [2.30, 3.19]	−1.07	−2.52 to 0.38	0.14	Objective
Pleurodesis failure	41	235/1,424	590/1,399	0.26 [0.22, 0.32]	0.13	−1.35 to 1.60	0.87	Objective
Pleural progression	13	25/481	90/475	0.22 [0.14, 0.36]	−0.28	−2.93 to 2.37	0.82	Objective
Quality of life	19	497/682	298/670	3.60 [2.84, 4.56]	−2.47	−4.62 to -0.32	0.03	Underestimation
Myelosuppression	17	149/574	229/558	0.34 [0.24, 0.47]	1.20	−1.76 to 4.16	0.39	Objective
Leukopenia	20	178/711	291/703	0.35 [0.26, 0.46]	−0.45	−1.59 to 0.67	0.41	Objective
Gastrointestinal reactions	31	254/1,053	440/1,035	0.36 [0.29, 0.44]	−1.46	−2.71 to -0.21	0.02	Underestimation
Hepatotoxicity	22	43/837	87/824	0.42 [0.28, 0.63]	−0.007	−0.76 to 0.78	0.98	Objective
Nephrotoxicity	31	75/1,105	169/1,090	0.32 [0.24, 0.44]	−0.15	−0.91 to 0.60	0.67	Objective
Thoracodynia	11	49/402	66/394	0.66 [0.43, 1.02]	0.06	−1.49 to 1.61	0.93	Objective
Fever	26	65/853	97/828	0.50 [0.33, 0.76]	0.35	−1.42 to 2.13	0.68	Objective

Note: OR, odds ratios; CI, confidence interval.

### 3.10 Sensitivity analysis

In CKI versus cisplatin, 11 outcomes were pooled using meta-analysis. Before and after excluding the trials with high risk and over-estimating efficacy/safety, the OR of QOL, myelosuppression, gastrointestinal reaction, and thoracodynia showed poor robustness, and the others had good robustness. In perfusion with CKI and cisplatin, 13 outcomes were pooled, and QOL, thrombocytopenia and anemia showed poor robustness. In CKI and nedaplatin, lobaplatin, bleomycin, hydroxycamptothecin, interleukin-2, or OK-432, 12 outcomes were pooled, and only the complete response of CKI and nedaplatin, lobaplatin, or OK-432 showed good robustness. In *kang’ai* and cisplatin, six outcomes were pooled, showing poor robustness. In matrine and cisplatin, 11 outcomes were pooled, and the QOL, myelosuppression, neutropenia, and gastrointestinal reaction showed poor robustness (Table 6).

**TABLE 6:** Sensitivity analysis.

Outcomes	Before excluding trials					Excluded trials with high risk and over-estimating efficacy and safety	After excluding trials					Sensitivity
	Trials	SM	OR (95% CI)	I^2^	P		Trials	SM	OR (95% CI)	I^2^	P	
a. Compound *kushen* injection (CKI) alone												
CKI versus cisplatin												
Complete response	9	FEM	1.10 [0.76, 1.60]	0%	*p = 0.60*	Poor*: (Wang and Zhou, 2016), Over*:no	8	FEM	1.07 [0.73, 1.58]	0%	p = 0.72	Robustness
Pleurodesis failure	9	FEM	0.80 [0.56, 1.14]	0%	p = 0.21	Poor*: (Wang and Zhou, 2016), Under*: (Xing, 2013)	8	FEM	0.76 [0.52, 1.11]	0%	p = 0.15	Robustness
Pleural progression	6	FEM	0.63 [0.33, 1.21]	0%	p = 0.17	Poor*:no, Under*:no	6	FEM	0.63 [0.33, 1.21]	0%	p = 0.17	Robustness
Quality of life	6	REM	1.52 [0.69, 3.35]	67%	p = 0.30	Poor*: (Yuan, 2007; Hu et al., 2008b; Liang et al., 2011; Chen, 2013; Xing, 2013; Yan et al., 2016), Over*:no	No	No	No	No	No	Poor
Myelosuppression	6	REM	0.02 [0.00, 0.15]	69%	p < 0.0001	Poor*:no (Wang and Zhou, 2016; Yan et al., 2016), Under*: (Yuan, 2007; Liang et al., 2011; Xing, 2013)	1	No	0.46 [0.09, 2.41]	No	*p* = 0.36	Poor
Neutropenia	4	FEM	0.10 [0.03, 0.35]	0%	*p* = 0.0003	Poor*: (Chen, 2013), Under*: no	2	FEM	0.07 [0.01, 0.60]	0%	p = 0.01	Robustness
Gastrointestinal reaction	9	REM	0.03 [0.01, 0.12]	67%	*p* < 0.00001	Poor*: (Chen, 2013; Wang and Zhou, 2016; Yan et al., 2016), Under*: (Yuan, 2007; Chen, 2010; Liang et al., 2011; Xing, 2013)	2	REM	0.26 [0.02, 3.08]	57%	p = 0.28	Poor
Hepatotoxicity	6	FEM	0.09 [0.02, 0.33]	0%	p = 0.0003	Poor*: (Wang and Zhou, 2016), Under*: (Liang et al., 2011)	4	FEM	0.19 [0.04, 0.92]	0%	p = 0.04	Robustness
Nephrotoxicity	7	FEM	0.09 [0.03, 0.29]	0%	*p < 0.0001*	Poor*: (Wang and Zhou, 2016), Under*: (Liang et al., 2011)	5	FEM	0.16 [0.04, 0.63]	0%	*p = 0.009*	Robustness
Thoracodynia	6	REM	0.15 [0.04, 0.48]	69%	p = 0.002	Poor*: (Chen, 2013), Under*: (Yuan, 2007; Hu et al., 2008b; Chen, 2010; Wang et al., 2023b)	1	Not	0.97 [0.34, 2.78]	No	p = 0.96	Poor
Fever	5	REM	0.67 [0.12, 3.76]	81%	*p* = 0.65	Poor*: (Chen, 2013), Under*: (Hu et al., 2008b)	3	REM	1.96 [0.09, 43.10]	88%	p = 0.67	Robustness
b. CKI and sclerosants												
CKI and cisplatin versus cisplatin												
Complete response	41	FEM	2.71 [2.30, 3.19]	0%	p < 0.00001	Poor*: (Lin et al., 2023), Over *: (Ning et al., 2001; Lin et al., 2007; Deng et al., 2008; Li, 2008; Li et al., 2009; Chen et al., 2011; Han, 2013; Xu, 2014a; Liu and Li, 2015; Song and Jia, 2015; Huang et al., 2017; Wang et al., 2019; Jiang and Li, 2020; Peng, 2020)	26	FEM	1.94 [1.56, 2.43]	0%	p < 0.00001	Robustness
Pleurodesis failure	41	FEM	0.26 [0.22, 0.32]	0%	p < 0.00001	Poor*: Lin et al., 2023 ), Under*: (Lin et al., 2007; Ding et al., 2009; Wang, 2010; Chen et al., 2011; Li and Tian, 2011; Ran and Zang, 2011; Chen and Liao, 2012; Han et al., 2012; Yang, 2012; Guo et al., 2013; Han, 2013; Zheng and Jia, 2013; Jiang, 2014; Xu, 2014b; Liu and Li, 2015; Song and Jia, 2015; Qin and Fan, 2016; Yan et al., 2016; Huang et al., 2017; Liu et al., 2017; Tang et al., 2018; Wang et al., 2019; Wu et al., 2019; Jiang and Li, 2020; Peng, 2020; Feng and Shi, 2023)	14	FEM	0.41 [0.29, 0.56]	0%	p < 0.00001	Robustness
Pleural progression	13	FEM	0.22 [0.14, 0.36]	0%	p < 0.00001	Poor*:no, Under*: (Han, 2013; Wang et al., 2019; Jiang and Li, 2020; Peng, 2020)	9	FEM	0.35 [0.19, 0.64]	0%	P = 0.0006	Robustness
Quality of life	19	FEM	3.60 [2.84, 4.56]	0%	p < 0.00001	Poor*: (Pan et al., 2007; Ding et al., 2009; Ran and Zang, 2011; Han et al., 2012; Yang, 2012; Guo et al., 2013; Han, 2013; Chen et al., 2014; Jiang, 2014; Xu, 2014b; Liu and Li, 2015; Yan et al., 2016; Liu et al., 2017; Shi, 2017; Tang et al., 2018; Wang et al., 2019; Wu et al., 2019; Feng and Shi, 2023; Lin et al., 2023), Over*:no	No	No	No	No	No	Poor
Myelosuppression	17	FEM	0.34 [0.24, 0.47]	0%	p < 0.00001	Poor*: (Lin et al., 2007; He et al., 2010; Li and Tian, 2011; Zheng and Jia, 2013; Jiang, 2014; Xu, 2014a; Liu and Li, 2015; Song and Jia, 2015; Yan et al., 2016; Huang et al., 2017; Lin et al., 2023 ), Under*: (Yang, 2012)	5	FEM	0.35 [0.18, 0.69]	0%	p = 0.002	Robustness
Neutropenia	20	FEM	0.35 [0.26, 0.46]	0%	p < 0.00001	Poor*: (Ning et al., 2001; Pan et al., 2007; Deng et al., 2008; Li, 2008; He et al., 2010; Wang, 2010; Chen et al., 2011; Ran and Zang, 2011; Zhu et al., 2013; Jiang, 2014; Liu et al., 2017; Shi, 2017; Tang et al., 2018), Under*: (Huang, 2007; Li et al., 2009; Chen et al., 2014)	4	FEM	0.43 [0.23, 0.82]	0%	P = 0.01	Robustness
Thrombocytopenia	5	FEM	0.76 [0.27, 2.12]	0%	p = 0.61	Poor*: (Pan et al., 2007; Chen et al., 2011; Jiang, 2014), Under*:no	2	Not	Not	Not	Not	Poor
Anemia	2	FEM	0.69 [0.21, 2.24]	0%	p = 0.54	Poor*: (Pan et al., 2007; Chen et al., 2011), Under*:no	No	No	No	No	No	Poor
Gastrointestinal reaction	31	FEM	0.37 [0.30, 0.47]	8%	p < 0.00001	Poor*: (Lin et al., 2007; He et al., 2010; Ran and Zang, 2011; Zheng and Jia, 2013; Zhu et al., 2013; Jiang, 2014; Xu, 2014a; Liu and Li, 2015; Song and Jia, 2015; Qin and Fan, 2016; Yan et al., 2016; Huang et al., 2017; Liu et al., 2017; Shi, 2017; Tang et al., 2018; Wang et al., 2019; Peng, 2020; Lin et al., 2023), Under*: (Ding et al., 2009; Yang, 2012; Guo et al., 2013; Chen et al., 2014; Wu et al., 2019; Feng and Shi, 2023)	7	FEM	0.48 [0.31, 0.74]	0%	P = 0.0009	Robustness
Hepatotoxicity	22	FEM	0.42 [0.28, 0.63]	0%	p < 0.0001	Poor*: (Lin et al., 2007; Pan et al., 2007; He et al., 2010; Chen et al., 2011; Li and Tian, 2011; Zhu et al., 2013; Jiang, 2014; Liu and Li, 2015; Song and Jia, 2015; Qin and Fan, 2016; Lin et al., 2023), Under*:no	11	FEM	0.37 [0.22, 0.63]	0%	p = 0.0002	Robustness
Nephrotoxicity	31	FEM	0.32 [0.24, 0.44]	0%	p < 0.0001	Poor*: (Ning et al., 2001; Lin et al., 2007; Pan et al., 2007; Deng et al., 2008; Li, 2008; He et al., 2010; Wang, 2010; Chen et al., 2011; Li and Tian, 2011; Ran and Zang, 2011; Zheng and Jia, 2013; Zhu et al., 2013; Jiang, 2014; Xu, 2014a; Liu and Li, 2015; Song and Jia, 2015; Qin and Fan, 2016; Lin et al., 2023), Under*: (Wu et al., 2019)	12	FEM	0.35 [0.21, 0.59]	0%	p < 0.0001	Robustness
Thoracodynia	11	FEM	0.65 [0.42, 1.00]	0%	p = 0.05	Poor*: (Li and Tian, 2011; Ran and Zang, 2011; Liu and Li, 2015; Wang et al., 2019; Peng, 2020), Under*:no	6	FEM	0.50 [0.28, 0.89]	0%	p = 0.02	Robustness
Fever	15	FEM	0.50 [0.30, 0.82]	0%	p = 0.006	Poor*: (Lin et al., 2007; Li and Tian, 2011; Ran and Zang, 2011; Zhu et al., 2013; Liu and Li, 2015; Qin and Fan, 2016; Wang et al., 2019; Peng, 2020), Under*:no	7	FEM	0.35 [0.15, 0.79]	0%	p = 0.01	Robustness
CKI and nedaplatin versus nedaplatin												
Complete response	3	FEM	1.72 [0.99, 2.98]	0%	p = 0.05	Poor*:no, Over*:no	3	FEM	1.72 [0.99, 2.98]	0%	p = 0.05	Robustness
Pleurodesis failure	3	FEM	0.33 [0.19, 0.57]	0%	p < 0.0001	Poor*:no, Under*: (Li, 2014; Zhang et al., 2015a; Li et al., 2017)	No	No	No	No	No	Poor
CKI and lobaplatin versus lobaplatin												
Complete response	2	FEM	1.57 [0.73, 3.36]	44%	p = 0.25	Poor*:no, Over *:no	2	FEM	1.57 [0.73, 3.36]	44%	p = 0.25	Robustness
Pleurodesis failure	2	FEM	0.35 [0.13, 0.93]	0%	p = 0.04	Poor*:no, Under*: (Huang, 2021)	1	No	0.46 [0.08, 2.75]	No	p = 0.40	Poor
CKI and bleomycin versus bleomycin												
Complete response	3	FEM	2.62 [1.23, 5.58]	0%	p = 0.01	Poor*:no, Over *: (Chen and He, 2003)	2	FEM	2.17 [0.91, 5.16]	0%	p = 0.08	Poor
Pleurodesis failure	3	FEM	0.23 [0.11, 0.52]	0%	p = 0.0004	Poor*:no, Under*: (Liu and Wan, 2011; Sun, 2012)	1	No	0.13 [0.01, 1.29]	No	p = 0.08	Poor
CKI and hydroxycamptothecin versus hydroxycamptothecin												
Complete response	2	FEM	3.01 [1.54, 5.87]	0%	p = 0.001	Poor*:no, Under*: (He et al., 2009; Cai and Wang, 2019)	No	No	No	No	No	Poor
Pleurodesis failure	3	FEM	0.37 [0.19, 0.72]	0%	p = 0.004	Poor*: (Wu et al., 2014), Under*: (Cai and Wang, 2019)	1	No	0.55 [0.16, 1.93]	No	p = 0.35	Poor
CKI and interleukin-2 versus interleukin-2												
Complete response	2	FEM	3.21 [1.41, 7.34]	0%	p = 0.006	Poor*:no, Under*: (Hao and Liang, 2007)	1	No	2.67 [0.92, 7.70]	No	p = 0.07	Poor
Pleurodesis failure	2	FEM	0.24 [0.10, 0.60]	0%	p = 0.002	Poor*:no, Under*: (Hao and Liang, 2007; Zhou et al., 2010)	No	No	No	No	No	Poor
CKI and OK-432 versus OK-432												
Complete response	2	FEM	1.58 [0.77, 3.21]	0%	p = 0.21	Poor*:no, Over*:no	2	FEM	1.58 [0.77, 3.21]	0%	p = 0.21	Robustness
Pleurodesis failure	2	FEM	0.32 [0.16, 0.67]	0%	p = 0.002	Poor*:no, Under*: (Wei et al., 2014; Zhong et al., 2015)	No	No	No	No	No	Poor
c. *Kang’ai* and cisplatin versus cisplatin												
Complete response	5	FEM	3.04 [1.76, 5.26]	0%	p < 0.0001	Poor*:no, Over*: (Hu et al., 2008a; Xu and Xiong, 2008; Wang, 2016)	2	FEM	2.00 [0.72, 5.57]	0%	p = 0.18	Poor
Pleurodesis failure	6	FEM	0.23 [0.14, 0.41]	0%	P < 0.00001	Poor*: (Qu et al., 2012), Under*: (Hu et al., 2008a; Xu and Xiong, 2008; Wang, 2016)	2	FEM	0.49 [0.18, 1.31]	0%	P = 0.15	Poor
Quality of life	2	FEM	3.95 [1.78, 8.74]	0%	p = 0.0007	Poor*: (Xu and Xiong, 2008; Qu et al., 2012), Over*:no	No	No	No	No	No	Poor
Neutropenia	4	FEM	0.20 [0.11, 0.38]	25%	p < 0.0001	Poor*: (Hu et al., 2008a; Xu and Xiong, 2008; He, 2011; Qu et al., 2012), Under*:no	No	No	No	No	No	Poor
Gastrointestinal reaction	5	FEM	0.34 [0.19, 0.63]	0%	p = 0.0006	Poor*: (Zhang, 2006; Hu et al., 2008a; Xu and Xiong, 2008; He, 2011; Qu et al., 2012), Under*:no	No	No	No	No	No	Poor
Thoracodynia	2	FEM	0.41 [0.13, 1.29]	0%	p = 0.13	Poor*: (Hu et al., 2008a; Qu et al., 2012), Under*:no	No	No	No	No	No	Poor
d. Matrine and cisplatin versus cisplatin (six trials)												
Complete response	6	FEM	1.87 [1.26, 2.78]	0%	p = 0.002	Poor*:no, Over*: (Li and Yang, 2009)	5	FEM	1.73 [1.13, 2.66]	0%	p = 0.01	Robustness
Pleurodesis failure	6	FEM	0.27 [0.17, 0.44]	0%	P < 0.00001	Poor*:no, Under*: (Du et al., 2009; Li and Yang, 2009; He, 2010)	2	FEM	0.32 [0.16, 0.64]	0%	P = 0.001	Robustness
Pleural progression	2	FEM	0.29 [0.09, 0.95]	0%	p = 0.04	Poor*:no, Under*:no	2	FEM	0.29 [0.09, 0.95]	0%	p = 0.04	Robustness
Quality of life	2	FEM	2.93 [1.23, 6.96]	0%	p = 0.02	Poor*: (Wang et al., 2010; Ji, 2011), Over*:no	No	No	No	No	No	Poor
Myelosuppression	3	FEM	0.49 [0.21, 1.11]	43%	P = 0.09	Poor*: (He, 2010; Wang et al., 2010; Ji, 2011), Under*:no	No	No	No	No	No	Poor
Neutropenia	2	REM	0.10 [0.02, 0.61]	66%	P = 0.01	Poor*:no, Under*: (Li and Yang, 2009)	1	No	0.26 [0.05, 1.40]	No	P = 0.12	Poor
Gastrointestinal reaction	5	FEM	0.35 [0.19, 0.66]	0%	p = 0.001	Poor*: (He, 2010; Wang et al., 2010; Ji, 2011),Under*: (Li and Yang, 2009)	1	No	0.42 [0.07, 2.45]	No	p = 0.34	Poor
Hepatotoxicity	3	FEM	0.52 [0.23, 1.15]	0%	p = 0.10	Poor*: (He, 2010), Under*:no	2	FEM	0.40 [0.16, 1.04]	0%	p = 0.06	Robustness
Nephrotoxicity	4	FEM	0.56 [0.19, 1.59]	0%	p = 0.27	Poor*: (He, 2010; Wang et al., 2010), Under*:no	2	FEM	0.56 [0.19, 1.59]	0%	p = 0.27	Robustness
Thoracodynia	4	FEM	0.21 [0.10, 0.48]	0%	p = 0.0007	Poor*: (Wang et al., 2010; Ji, 2011), Under*:no	2	FEM	0.24 [0.09, 0.64]	0%	p = 0.004	Robustness
Fever	4	FEM	0.41 [0.16, 1.07]	0%	p = 0.07	Poor*: (He, 2010; Ji, 2011), Under*:no	2	FEM	0.57 [0.17, 1.86]	0%	p = 0.35	Robustness

Note: SM, statistical method; FEM, fixed-effects model, REM: random-effects model, OR, odds ratios; CI, confidence interval. High-risk trials (Poor*) had at least one domain considered as high risk of bias. Over*:over-estimating efficacy or Under*: under-estimating risk, trials with results which were significantly different and beneficial to *kushen* administration.

### 3.11 Trial sequential analyses

**FIGURE 6:**
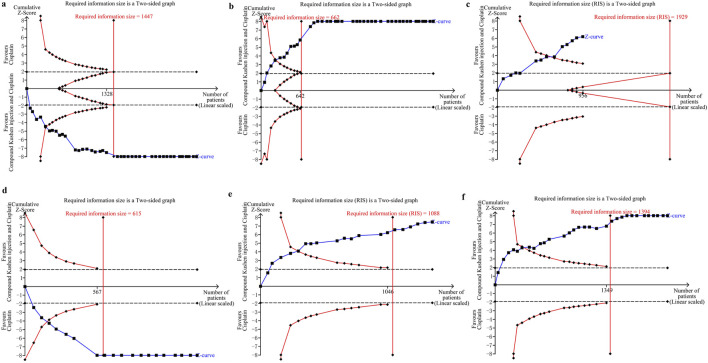
Trial sequential analyses in compound *kushen* injection and cisplatin. **(a)** Complete response; **(b)** pleurodesis failure; **(c)** pleural progression; **(d)** quality of life; **(e)** neutropenia; **(f)** gastrointestinal reaction.

Since the trials were limited, we only assessed the RIS for clinical responses in CKI versus cisplatin. The TSA identified firm information size for supporting a similar complete response and pleurodesis failure between CKI and cisplatin, and no reliable information for pleural progression. We further assessed the RIS for clinical responses, QOL, and AEs in perfusion with CKI and cisplatin. Further analysis identified sufficient and conclusive information sizes for complete response, pleurodesis failure, QOL, neutropenia, and gastrointestinal reaction, and firm information for pleural progression, myelosuppression, and hepatorenal toxicity. Finally, we only assessed the RIS for clinical responses in *kang’ai* or matrine and cisplatin. The analysis identified firm information sizes for pleurodesis failure in both treatments and no reliable information for complete response (Table.7; Figure 6; Supplementary Figures S84–S94).

**TABLE 7:** Results of trial sequential analysis.

Outcomes (trials, patients)	Relative risk reduction (RRR)	Incidence	I^2^	D^2^	RIS	% of RIS attained	Z-curve passed conventional boundaries?	Z-curve passed TSA/futility boundaries?	Z-curve passed RIS?
a. Compound *kushen* injection (CKI) versus cisplatin (Supplementary Figures S84–S86)									
Complete response (9 trials, n = 568)	25%	30%	0%	0%	1,081	52.54	No	Yes	No
Pleurodesis failure (9 trials, n = 568)	25%	36%	0%	0%	837	67.86	No	Yes	No
Pleural progression (6 trials, n = 348)	25%	16%	0%	0%	2,363	14.73	No	No	No
b. CKI and cisplatin versus cisplatin (Figure 6 and Supplementary Figures S87–S90)									
Complete response (41 trials, n = 2,823)	25%	24%	0%	0%	1,447	195.09	Yes	Yes	Yes
Pleurodesis failure (41 trials, n = 2,823)	25%	42%	0%	0%	662	426.44	Yes	Yes	Yes
Pleural progression (13 trials, n = 956)	25%	19%	0%	0%	1929	49.56	Yes	Yes	No
Quality of life (19 trials, n = 1,352)	25%	44%	0%	0%	615	219.84	Yes	Yes	Yes
Myelosuppression (17 trials, n = 1,132)	20%	38%	0%	0%	1,224	92.48	Yes	Yes	No
Neutropenia (20 trials, n = 1,414)	20%	41%	0%	0%	1,088	130.00	Yes	Yes	Yes
Gastrointestinal reaction (31 trials, n = 2088)	20%	41%	19%	22%	1,394	149.78	Yes	Yes	Yes
Hepatotoxicity (22 trials, n = 1,661)	20%	11%	0%	0%	5,787	28.70	Yes	Yes	No
Nephrotoxicity (31 trials, n = 2,195)	20%	16%	0%	0%	3,780	58.07	Yes	Yes	No
Fever (15 trials, n = 954)	20%	10%	0%	0%	6,429	14.84	9	No	No
c. *Kang’ai* and cisplatin versus cisplatin (Supplementary Figures S91, S92)									
Complete response (5 trials, n = 288)	25%	17%	0%	0%	2,201	13.08	Yes	No	No
Pleurodesis failure (6 trials, n = 334)	25%	42%	0%	0%	662	50.45	Yes	Yes	No
c. Matrine and cisplatin versus cisplatin (Supplementary Figures S93, S94)									
Complete response (6 trials, n = 471)	25%	30%	0%	0%	1,081	43.57	Yes	No	No
Pleurodesis failure (6 trials, n = 471)	25%	33%	0%	0%	948	49.68	Yes	Yes	No

Note: I^2^, inconsistency; D^2^, diversity; RIS, required information size; RRR, relative risk reduction.

### 3.12 Evidence quality

We applied a revised GRADE approach to identify the evidence quality as “high”, “moderate”, “low”, and “very low”. In CKI versus cisplatin, 11 results were pooled. Clinical responses, hepatorenal toxicity, and fever were summarized as moderate quality, while other five results were low to very low (Table 8a). In perfusion with CKI and cisplatin, 13 results were pooled. Clinical responses, myelosuppression, neutropenia, gastrointestinal reaction, hepatorenal toxicity, and fever were summarized as moderate, while the other four were low to very low. In CKI and nedaplatin, lobaplatin, bleomycin, hydroxycamptothecin, interleukin-2, or OK-432, 12 results were pooled. The clinical responses were very low to low (Table 8b). In *kang’ai* and cisplatin, six results were pooled at low to very low (Table 8c). In matrine and cisplatin, 11 results were pooled. The complete response and pleurodesis failure were summarized as moderate, with the other nine results as low to very low (Table 8d).

**TABLE 8:** GRADE evidence profiles.

Outcomes (trials)	Quality assessment					Malignant pleural effusion		Clinical effectiveness and safety		Quality
	i	ii	iii	iv	v	RSF	Sclerosants	Odds ratios (95% CI)	Absolute effect	
a. Compound *kushen* injection (CKI) versus cisplatin (DDP)										
Complete response (9)	Serious^a^	Not	Not	Not	None	86/281 (30.6%)	86/287 (30%)	1.07 (0.74–1.54)	14 more per 1,000 (from 59 fewer to 98 more)	⊕⊕⊕Ο
Pleurodesis failure (9)	Serious^a^	Not	Not	Not	None	86/281 (30.6%)	104/287(36.2%)	0.77 (0.54 to 1.1)	58 fewer per 1,000 (from 128 fewer to 22 more)	⊕⊕⊕Ο
Pleural progression (6)	Serious^b^	Not	Not	Not	None	16/175 (9.1%)	28/173 (16.2%)	0.51 (0.26 to 0.98)	72 fewer per 1,000 (from 3 fewer to 114 fewer)	⊕⊕⊕Ο
Quality of life (6)	Very serious^c^	Serious^g^	Not	Not	None	144/207 (69.6%)	127/212 (59.9%)	1.52 (0.69–3.35)	95 more per 1,000 (from 91 fewer to 234 more)	⊕ΟΟΟ
Myelosuppression (6)	Sery serious^c^	serious^g^	Not	Not	None	3/193 (1.6%)	109/197 (55.3%)	0.02 (0 to 0.15)	529 fewer per 1,000 (from 397 fewer to 553 fewer)	⊕ΟΟΟ
Neutropenia (3)	Serious^a^	Not^d^	Not	Serious^e^	none	2/88 (2.3%)	22/90 (24.4%)	0.1 (0.03–0.35)	213 fewer per 1,000 (from 143 fewer to 235 fewer)	⊕⊕ΟΟ
Gastrointestinal reaction (9)	Very serious^c^	Serious^g^	Not	Not	None	10/281 (3.6%)	167/287 (58.2%)	0.03 (0.01 to 0.12)	542 fewer per 1,000 (from 439 fewer to 568 fewer)	⊕ΟΟΟ
Hepatotoxicity (6)	Serious^a^	Not	Not	Not	None	1/201(0.5%)	22/197(11.2%)	0.09 (0.02 to 0.33)	100 fewer per 1,000 (from 72 fewer to 109 fewer)	⊕⊕⊕Ο
Nephrotoxicity (6)	Serious^a^	Not	Not	Not	None	1/221 (0.5%)	26/217 (12%)	0.09 (0.03 to 0.29)	108 fewer per 1,000 (from 82 fewer to 116 fewer)	⊕⊕⊕Ο
Thoracodynia (6)	Very serious^f^	Serious^g^	Not	Not	None	17/159 (10.7%)	63/158 (39.9%)	0.14 (0.03 to 0.61)	314 fewer per 1,000 (from 111 fewer to 379 fewer)	⊕ΟΟΟ
Fever (5)	Serious^a^	Not^d^	Not	Not	None	29/159 (18.2%)	31/158 (19.6%)	0.94 (0.55–1.59)	10 fewer per 1,000 (from 78 fewer to 83 more)	⊕⊕⊕Ο
Table 8b. Intrapleural administration with CKI and sclerosants										
CKI and Cisplatin versus cisplatin										
Complete response (41)	Serious^b^	Not	Not	Not	None	649/1,424 (45.6%)	342/1,399 (24.4%)	2.71 (2.3 to 3.19)	223 more per 1,000 (from 182 more to 263 more)	⊕⊕⊕Ο
Pleurodesis failure (41)	Serious^b^	Not	Not	Not	None	235/1,424 (16.5%)	590/1,399 (42.2%)	0.26 (0.22 to 0.32)	262 fewer per 1,000 (from 233 fewer to 283 fewer)	⊕⊕⊕Ο
Pleural progression (13)	Serious^b^	Not	Not	Not	None	25/481 (5.2%)	90/475 (18.9%)	0.22 (0.14–0.36)	141 fewer per 1,000 (from 112 fewer to 158 fewer)	⊕⊕⊕Ο
Quality of life (19)	Very serious^c^	Not	Not	Not	Reporting bias^h^	497/682 (72.9%)	298/670 (44.5%)	3.56 (2.8 to 4.53)	296 more per 1,000 (from 247 more to 339 more)	⊕ΟΟΟ
Myelosuppression (17)	Serious^a^	Not	Not	Not	None	149/574 (26%)	229/558 (41%)	0.34 (0.24 to 0.47)	219 fewer per 1,000 (from 164 fewer to 267 fewer)	⊕⊕⊕Ο
Neutropenia (20)	Serious^a^	Not	Not	Not	None	178/711 (25%)	291/703 (41.4%)	0.35 (0.27–0.46)	216 fewer per 1,000 (from 169 fewer to 254 fewer)	⊕⊕⊕Ο
Thrombocytopenia (5)	Very serious^f^	Not	Not	Not	None	7/215 (3.3%)	9/213 (4.2%)	0.76 (0.27–2.12)	10 fewer per 1,000 (from 30 fewer to 43 more)	⊕⊕ΟΟ
Anemia (2)	Very serious^c^	Not	Not	Serious^e^	None	5/120 (4.2%)	7/118 (5.9%)	0.69 (0.21–2.24)	18 fewer per 1,000 (from 46 fewer to 64 more)	⊕ΟΟΟ
Gastrointestinal reaction (31)	Serious^a^	Not^d^	Not	Not	None^8i^	254/1,053 (24.1%)	440/1,035 (42.5%)	0.36 (0.29 to 0.44)	215 fewer per 1,000 (from 180 fewer to 249 fewer)	⊕⊕⊕Ο
Hepatotoxicity (22)	Serious^a^	Not	Not	Not	None	43/837 (5.1%)	87/824 (10.6%)	0.42 (0.28 to 0.63)	58 fewer per 1,000 (from 36 fewer to 74 fewer)	⊕⊕⊕Ο
Nephrotoxicity (31)	Serious^a^	Not	Not	Not	None	75/1,105 (6.8%)	169/1,090 (15.5%)	0.32 (0.24 to 0.44)	100 fewer per 1,000 (from 80 fewer to 113 fewer)	⊕⊕⊕Ο
Thoracodynia (11)	Very serious^f^	Not	Not	Not	None	49/402 (12.2%)	66/394 (16.8%)	0.65 (0.42–1)	52 fewer per 1,000 (from 90 fewer to 0 more)	⊕⊕ΟΟ
Fever (15)	Serious^a^	Not	Not	Not	None	25/481 (5.2%)	47/473 (9.9%)	0.5 (0.3–0.82)	47 fewer per 1,000 (from 16 fewer to 67 fewer)	⊕⊕⊕Ο
CKI and Nedaplatin versus nedaplatin										
Complete response (3)	Serious^b^	Not	Not	Serious^e^	None	44/129 (34.1%)	30/129 (23.3%)	1.72 (0.99–2.98)	110 more per 1,000 (from 2 fewer to 242 more)	⊕⊕ΟΟ
Pleurodesis failure (3)	Serious^b^	Not	Not	Serious^e^	None	28/129 (21.7%)	58/129 (45%)	0.33 (0.19–0.57)	237 fewer per 1,000 (from 132 fewer to 315 fewer)	⊕⊕ΟΟ
CKI and lobaplatin versus lobaplatin										
Complete response (2)	Serious^b^	Not^d^	Not	Serious^e^	None	26/55 (47.3%)	20/55 (36.4%)	1.57 (0.73–3.36)	109 more per 1,000 (from 69 fewer to 294 more)	⊕⊕ΟΟ
Pleurodesis failure (2)	Serious^b^	Not	Not	Serious^e^	None	9/55 (16.4%)	18/55 (32.7%)	0.35 (0.13–0.93)	182 fewer per 1,000 (from 16 fewer to 268 fewer)	⊕⊕ΟΟ
CKI and bleomycin versus bleomycin										
Complete response (3)	Serious^b^	Not	Not	Serious^e^	None	33/77 (42.9%)	16/69 (23.2%)	2.62 (1.23–5.58)	210 more per 1,000 (from 39 more to 396 more)	⊕⊕ΟΟ
Pleurodesis failure (3)	Serious^b^	Not	Not	Serious^e^	None	12/77 (15.6%)	30/69 (43.5%)	0.23 (0.11–0.52)	284 fewer per 1,000 (from 149 fewer to 357 fewer)	⊕⊕ΟΟ
CKI and hydroxycamptothecin versus hydroxycamptothecin										
Complete response (2)	Serious^b^	Not	Not	Serious^e^	None	41/78 (52.6%)	21/78 (26.9%)	3.01 (1.54–5.87)	257 more per 1,000 (from 93 more to 415 more)	⊕⊕ΟΟ
Pleurodesis failure (3)	Very serious^f^	Not	Not	Serious^e^	None	15/120 (12.5%)	33/118 (28%)	0.37 (0.19–0.72)	154 fewer per 1,000 (from 61 fewer to 211 fewer)	⊕ΟΟΟ
CKI and interleukin-2 versus interleukin-2										
Complete response (2)	Serious^b^	Not	Not	Serious^e^	None	29/56 (51.8%)	13/51 (25.5%)	3.21 (1.41–7.34)	268 more per 1,000 (from 71 more to 460 more)	⊕⊕ΟΟ
Pleurodesis failure (2)	Serious^b^	Not	Not	Serious^e^	None	9/56 (16.1%)	22/51 (43.1%)	0.24 (0.1–0.6)	277 fewer per 1,000 (from 119 fewer to 361 fewer)	⊕⊕ΟΟ
CKI and OK-432 versus OK-432										
Complete response (2)	Serious^b^	Not	Not	Serious^e^	None	24/84 (28.6%)	17/84 (20.2%)	1.58 (0.77–3.21)	84 more per 1,000 (from 39 fewer to 246 more)	⊕⊕ΟΟ
Pleurodesis failure (2)	Serious^b^	Not	Not	Serious^e^	None	14/84 (16.7%)	32/84 (38.1%)	0.32 (0.16–0.67)	216 fewer per 1,000 (from 89 fewer to 291 fewer)	⊕⊕ΟΟ
c. Intrapleural administration with *kang’ai* and cisplatin versus cisplatin										
Complete response (5)	Very serious^f^	Not	Not	Not	None	56/144 (38.9%)	25/144 (17.4%)	3.04 (1.76–5.26)	216 more per 1,000 (from 96 more to 351 more)	⊕⊕ΟΟ
Pleurodesis failure (6)	Very serious^f^	Not	Not	Not	None	26/168 (15.5%)	69/166 (41.6%)	0.23 (0.14–0.41)	275 fewer per 1,000 (from 190 fewer to 325 fewer)	⊕⊕ΟΟ
Quality of life (2)	Very serious^c^	Not	Not	Serious^e^	None	34/57 (59.6%)	15/55 (27.3%)	3.95 (1.78–8.74)	324 more per 1,000 (from 128 more to 493 more)	⊕ΟΟΟ
Neutropenia (4)	Very serious^c^	Serious^g^	Not	Serious^e^	None	31/113 (27.4%)	67/110 (60.9%)	0.2 (0.11–0.38)	372 fewer per 1,000 (from 237 fewer to 463 fewer)	⊕ΟΟΟ
Gastrointestinal reaction (5)	Very serious^c^	Not	Not	Serious^e^	None	42/133 (31.6%)	65/131 (49.6%)	0.34 (0.19–0.63)	245 fewer per 1,000 (from 113 fewer to 339 fewer)	⊕ΟΟΟ
Thoracodynia (2)	Very serious^c^	Not	Not	Serious^e^	None	5/60 (8.3%)	10/57 (17.5%)	0.41 (0.13–1.29)	95 fewer per 1,000 (from 149 fewer to 40 more)	⊕ΟΟΟ
d. Intrapleural administration with matrine and cisplatin versus cisplatin										
Complete response (6)	Serious^b^	Not	Not	Not	None	106/249 (42.6%)	66/222 (29.7%)	1.87 (1.26–2.78)	144 more per 1,000 (from 50 more to 243 more)	⊕⊕⊕Ο
Pleurodesis failure (6)	Serious^b^	Not	Not	Not	None	32/249 (12.9%)	74/222 (33.3%)	0.27 (0.17–0.44)	214 fewer per 1,000 (from 153 fewer to 255 fewer)	⊕⊕⊕Ο
Pleural progression (2)	Serious^b^	Not	Not	Serious^e^	None	4/122 (3.3%)	11/106 (10.4%)	0.29 (0.09–0.95)	71 fewer per 1,000 (from 5 fewer to 93 fewer)	⊕⊕ΟΟ
Quality of life (2)	Very serious^c^	Not	Not	Serious^e^	None	32/50 (64%)	20/50 (40%)	2.95 (1.25–6.97)	263 more per 1,000 (from 55 more to 423 more)	⊕ΟΟΟ
Myelosuppression (3)	Very serious^c^	Serious^g^	Not	Serious^e^	None	14/97 (14.4%)	19/86 (22.1%)	0.49 (0.21–1.11)	99 fewer per 1,000 (from 165 fewer to 18 more)	⊕ΟΟΟ
Neutropenia (2)	Serious^b^	Serious^g^	Not	Serious^e^	None	7/70 (10%)	31/66 (47%)	0.1 (0.02–0.61)	388 fewer per 1,000 (from 119 fewer to 452 fewer)	⊕ΟΟΟ
Gastrointestinal reaction (5)	Very serious^f^	Not	Not	No	None	36/167 (21.6%)	55/152 (36.2%)	0.35 (0.19–0.66)	196 fewer per 1,000 (from 90 fewer to 265 fewer)	⊕⊕ΟΟ
Hepatotoxicity (3)	Serious^a^	Not	Not	Serious^e^	None	15/117 (12.8%)	22/102 (21.6%)	0.52 (0.23–1.15)	91 fewer per 1,000 (from 156 fewer to 25 more)	⊕⊕ΟΟ
Nephrotoxicity (4)	Serious^a^	Not	Not	Serious^e^	None	7/137 (5.1%)	11/122 (9%)	0.56 (0.19–1.59)	38 fewer per 1,000 (from 72 fewer to 46 more)	⊕⊕ΟΟ
Thoracodynia (4)	Serious^a^	Not	Not	Serious^e^	None	9/120 (7.5%)	31/116 (26.7%)	0.21 (0.1–0.48)	196 fewer per 1,000 (from 118 fewer to 232 fewer)	⊕⊕ΟΟ
Fever (4)	Serious^a^	Not	Not	Serious^e^	None	7/147 (4.8%)	15/132 (11.4%)	0.41 (0.16–1.07)	64 fewer per 1,000 (from 94 fewer to 7 more)	⊕⊕ΟΟ

Note: i: risk of bias; ii: inconsistency; iii: indirectness; iv: imprecision; v: publication bias; OR: odds ratios. RSF: Radix *Sophorae flavescentis*. Not: not serious.

^a^ Most trials had some concerns, and with high risk, sensitivity analysis showed good robustness, and evidence was rated down by only one level.

^b^ All trials had some concerns, and evidence was rated down by only one level.

^c^ All trials had high risk, and evidence was rated down by two levels.

^d^ Heterogeneity was found, sensitivity analysis showed good robustness, and not rated down.

^e^ Sample size for indicator was fewer than 300 cases, and evidence was rated down by one level.

^f^ Most trials had some concerns, and with high risk, sensitivity analysis showed poor robustness, and evidence was rated down by two levels.

^g^ Heterogeneity was found, sensitivity analysis showed poor robustness, and evidence was rated down by one level.

^h^ Publication bias was found, excluded the under- or over-estimated studies and high risk studies, sensitivity analysis showed poor robustness, and evidence was rated down by one level.

^i^ Publication bias was found, excluded the under- or over-estimated studies and high risk studies, sensitivity analysis showed good robustness, and was not downgraded.

## 4 Discussion

**FIGURE 7:**
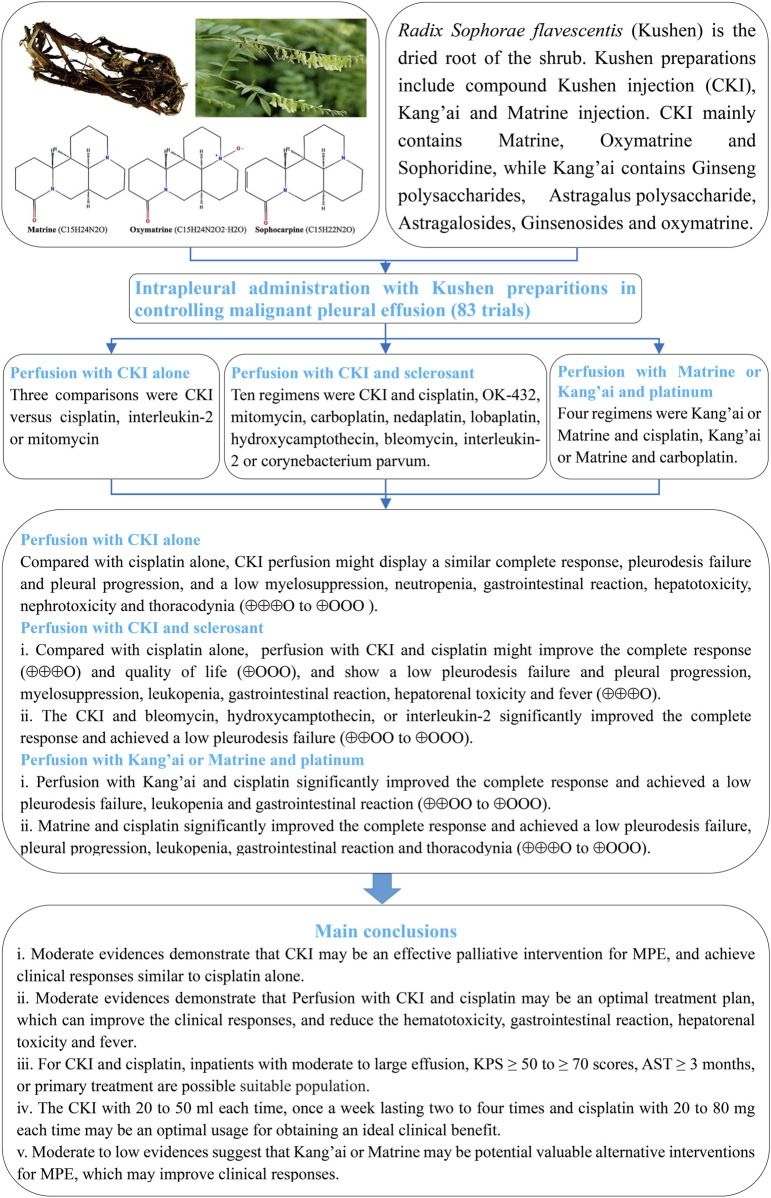
Evidence framework of *kushen* preparations for MPE.

After integrating previous six SRs/meta-analyses (Tian et al., 2010; Tang et al., 2014; Biaoxue et al., 2015; Xu et al., 2015; Yang et al., 2016; Wu et al., 2018) and four network meta-analyses (Yang et al., 2017; Li B. et al., 2019; Li, 2022; Xu et al., 2022), we collected 83 RCTs for analysis and supplemented 39 trials in previous studies. We found three *kushen* preparations—CKI, *kang’ai* and matrine injection—which were administrated for controlling MPE through intrapleural perfusion. For *kushen* preparation alone, nine trials evaluated perfusion with CKI versus cisplatin alone. CKI mainly contains matrine, oxymatrine, and sophoridine, which have significant anti-tumor activity, regulate tumor microenvironment, and downregulate tumor-associated inflammation (Guo et al., 2015; Ma et al., 2016; Cao and He, 2020; Chen et al., 2021; Chen et al., 2022; Liu et al., 2023). The meta-analysis results demonstrated that perfusion with CKI alone showed clinical responses similar to cisplatin and a lower hepatorenal toxicity (Figure 7). These results were of moderate quality following the revised GRADE approach (Wang et al., 2022; Wang C. Q. et al., 2023), and the TSA found firm information sizes for supporting them. CKI perfusion showed low hematotoxicity, gastrointestinal reaction, and thoracodynia of low to very low quality. Zhang Z. et al. (2015), Zhong et al. (2015), Zhu and Hou (2021), Fan et al. (2022); Feng and Shi (2023) reported that CKI perfusion might prevent pleural effusion recurrence by downregulating the vascular endothelial cell growth factor and reducing angiogenesis. In all, these results suggest that CKI may serve as a new palliative intervention for MPE. Clinically, CKI, *kang’ai*, and matrine injections have been widely used as an adjuvant therapy for various solid tumors (Ma et al., 2016; Wang et al., 2016; Li H. et al., 2019; Liu et al., 2022; Liu et al., 2023). Apparently, this analysis further revealed a new therapeutic value and clinical application population of CKI. Unfortunately, no evidence supports the possibility of using *kang’ai* and matrine alone to treat MPE, which requires new trials to investigate.

Clinically, CKI is often combined with other sclerosants to control MPE through intrapleural perfusion. We found that CKI combined with seven chemical drugs or three BRMs to build ten homogenous treatment plans. The clinical values of perfusion with CKI and cisplatin have been reported by 41 trials. Compared with cisplatin alone, the results of meta-analyses demonstrated that perfusion with CKI and cisplatin significantly improved complete response and QOL with a low pleurodesis failure and pleural progression, and showed a low incidence rate of hematotoxicity, gastrointestinal reaction, and hepatorenal toxicity. Excluding QOL, these results were moderate quality following the revised GRADE approach (Wang et al., 2022; Wang C. Q. et al., 2023). The results of pleural progression, myelosuppression, and hepatorenal toxicity had firm information in support, while other results obtained sufficient and conclusive information support. In all, these results demonstrate that CKI infusion can improve clinical responses and QOL and reduce ADRs. Like high dosage, the subgroup analysis revealed that CKI combined with low-dosage cisplatin also obtained similar clinical responses. These results indicate that CKI and cisplatin have cooperative effect, and CKI may reduce cisplatin dosage while ensuring similar clinical benefits. Previous SR/meta-analyses have reported that as important BRMs, staphylococcal enterotoxin C (Jiang et al., 2022) and mannatide (Zhang et al., 2011; Chen et al., 2013) perfusion showed a high risk of fever. In this analysis, we found that perfusion with CKI might reduce the risk of fever. This finding may be the unique value of CKI in controlling MPE. The results of meta-analysis of other nine treatment plans further revealed that perfusion with CKI and lobaplatin, nedaplatin, bleomycin, hydroxycamptothecin, interleukin-2, or OK-432 might also improve clinical responses. However, the results had very low to low quality and lacked sufficient or firm information sizes in support. Comprehensively examining both information sizes and methodological quality, we conclude that among ten treatment plans, perfusion with CKI and cisplatin may be an optimal treatment plan for MPE, which shows significant improvement in clinical responses and low incidence of ADRs, especially fever (Figure 7). Further subgroup analysis revealed that perfusion with CKI (20–50 mL each time, once a week lasting two to four times) and cisplatin (20–80 mg each time) could obtain ideal clinical responses for MPE inpatients with moderate to large effusion, KPS ≥50 to ≥70 scores, AST ≥3 months, or primary treatment. Furthermore, the primary tumor, drainages or evaluation criteria showed no negative effect on clinical responses. These results suggest that inpatients with moderate-to-large effusion, KPS ≥ 50 to ≥ 70 scores, AST ≥ 3 months, or primary treatment are a possible suitable population. The CKI with 20 to 50 ml each time, once a week lasting two to four times and cisplatin with 20 to 80 mg each perfusion may be an optimal usage for obtaining desired responses and safety (Figure 7). Unfortunately, both meta-regression analyses did not find any correlation. These results require new evidence for confirmation.

Matrine and *kang’ai* are also important *kushen* preparations. *Kang’ai* mainly contains *Astragalus* polysaccharides, astragalosides, ginsenosides, ginseng polysaccharides, and oxymatrine (Wan et al., 2018; Sun et al., 2021). Six trials each evaluated the clinical benefit of perfusion with *kang’ai* or matrine and cisplatin (Zhang, 2006; Hu J. et al., 2008; Xu and Xiong, 2008; He, 2011; Qu et al., 2012; Wang, 2016). The meta-analysis results showed that perfusion with *kang’ai* and cisplatin significantly improved the complete response and QOL with low pleurodesis failure. Matrine is a principal active ingredient of CKI and *kang’ai*. The results further demonstrated that matrine and cisplatin could improve complete response and QOL with low pleurodesis failure and pleural progression. These results provide a theoretical basis for the clinical value of *kang’ai* or CKI in MPE. Perfusion with *kang’ai* or matrine and cisplatin all showed low neutropenia and gastrointestinal reaction. However, only the pleurodesis failure of both treatment plans had a firm quantity of information in support, and no reliable information indicated that both can improve the complete response. For matrine and cisplatin, the complete response and pleurodesis failure had moderate quality, while other results were low to very low. Overall, these results suggest that *kang’ai* or matrine may be potentially valuable alternative interventions which may improve clinical responses with firm information size (Figure 7). Further rigorous trials will be needed to reveal their clinical significance, suitable population, and optimal usage.

*Kushen* preparations alone or plus chemical drugs or BRMs form rich treatment plans. To validate their therapeutic value for MPE, we applied clustering SR/meta-analysis, successfully addressing clinical heterogeneity and revealing their clinical efficacy and safety based on homogeneous treatment units. First, we found that CKI may serve as a new palliative intervention for MPE. This analysis confirmed the clinical possibility of using CKI perfusion to control MPE and further revealed its new therapeutic value and clinical application population. Second, among ten treatment plans, we found that perfusion with CKI and cisplatin may be an optimal treatment plan for MPE. Subgroup analysis results further provide a suitable population and optimal use for perfusion with CKI and cisplatin treating MPE. Third, we found that *kang’ai* or matrine may be potential valuable alternative interventions for MPE. In all, this analysis confirms and reveals the therapeutic value and clinical application population for using *kushen* preparations to control MPE. These findings will be beneficial for developing rational medication strategies based on *kushen* preparations to improve clinical benefits and reduce ADRs and medication costs in MPE.

There were some limitations to this new SR/meta-analysis. This analysis customized its retrieval strategies and retrieved both Chinese and English databases, which may exhibit potential bias risk. Among 14 treatment plans, most—like perfusion with CKI, *kang’ai*, or matrine and other sclerosants—only had limited trials reporting their clinical benefit. In particular, only single trials reported the clinical benefit between CKI and interleukin-2 (Huang, 2013) or mitomycin (Zhang, 2011), as well as perfusion with CKI and carboplatin (He and Xie, 2010), mitomycin (Zhang et al., 2013), or corynebacterium parvum (Huang et al., 2012). Most treatment plans lacked reliable information support, and their results were low to very low quality. Obviously, their clinical effectiveness, safety, indications, and optimal usage still require more high-quality evidence and sufficient information to confirm them. Regarding methodological quality, most studies had some concerns at overall bias about clinical response and overall survival. For both outcomes, D1 and D2 had some concerns.

QOL about perfusion with CKI alone were reported by 29 studies, and CKI, *kang’ai*, or matrine and cisplatin. All had high risk of overall bias, and D4 was a high-risk domain. AEs were reported by 57 studies. High risk of overall bias was evident in 35 studies, with D4 and D5 as high-risk domains. Such findings suggest that strengthening random allocation, concealment, and blinding methods, and emphasizing the measurement and complete report of indicators will become key issues for improving methodological quality in future trials. Regarding PICO features, most studies did not clearly report patient characteristics such as pleural fluid volume, KPS, AST, or treatment history. Most studies failed to clearly report the TRAEs. Six studies reported overall survival (Cui et al., 2008; Chen, 2010; He, 2011; Han, 2013; Zhang S. et al., 2015). Only single study reported that perfusion with CKI and cisplatin (Chen et al., 2011; Han, 2013) or nedaplatin (Zhang S. et al., 2015) and matrine and carboplatin (Cui et al., 2008) might improve overall survival or progression-free survival. Additionally, no evidence reported recurrence and hospitalization time or conflicts of interest. Such shortcomings of PICO are important issues for design and quality improvement in future trials.

## 5 Conclusion

Current moderate evidence demonstrates that CKI may be an effective palliative intervention for controlling MPE. Perfusion with CKI and cisplatin may be an optimal treatment plan which can improve clinical responses and QOL and reduce ADRs, especially fever. This analysis further confirms a suitable population and optimal usage for CKI and cisplatin perfusion. CKI, *kang’ai*, or matrine and chemical drugs or BRMs formed rich treatment plans for MPE. More rigorous trials with low-risk and standardized PICOs will be needed to reveal their clinical significance, suitable populations, and optimal usage.

## Data Availability

The original contributions presented in the study are included in the article/Supplementary Material; further inquiries can be directed to the corresponding author.

## Abbreviations

ADRs, adverse drug reactions; AEs, adverse events; AST, anticipated survival time; BRM, biological response modifier; CTCAEs, Common Terminology Criteria for Adverse Events; CKI, compound *kushen* injection; CI, confidence interval; FEM, fixed-effects model; GRADE, Grading of Recommendation Assessment, Development and Evaluation approach; IPCs, indwelling pleural catheters; *Kang’ai*, *kang’ai* injection; KPS, Karnofsky performance status; MPEs, malignant pleural effusions; NMA, network meta-analysis; ORs: odds ratios; PF, pleurodesis failure; PFS, progression-free survival; PRISMA, Preferred Reporting Items for Systematic Reviews and Meta-Analyses guidelines; QOL, quality of life; RCTs, randomized controlled trials; REM, random-effects model; RIS, required information size; RRR, relative risk reduction; SRs, systematic reviews; TCM, traditional Chinese medicine; TCMIs, traditional Chinese medicine injections; TSA, trial sequential analysis; WHO, World Health Organization.

## References

[B1] Biaoxue R. Shuxia M. Wenlong G. Shuanying Y. (2015). Thoracic perfusion of matrine as an adjuvant treatment improves the control of the malignant pleural effusions. World J. Surg. Oncol. 13, 329. 10.1186/s12957-015-0729-9 26631104 PMC4668654

[B2] Bibby A. C. Dorn P. Psallidas I. Porcel J. M. Janssen J. Froudarakis M. (2018). ERS/EACTS statement on the management of malignant pleural effusions. Eur. Respir. J. 52, 1800349. 10.1183/13993003.00349-2018 30054348

[B3] Cai H. Wang Q. (2019). Effect of Compound Kushen Injection on elderly patients with lung cancer complicated with malignant pleural effusion. Contemp. Med. Symp. 17, 161–162.

[B4] Cao X. He Q. (2020). Anti-tumor activities of bioactive phytochemicals in Sophora flavescens for breast cancer. Cancer Manag. Res. 12, 1457–1467. 10.2147/CMAR.S243127 32161498 PMC7051174

[B5] Chen B. Xu X.-M. Yu T.-T. Yang J. (2013). Meta analysis of Lifein combined with Cisplatin in chest catheterization perfusion chemotheapy for malignant pleural effusion. Med J Wuhan Univ 34, 755–761. 10.14188/j.1671-8852.2013.05.039

[B6] Chen F. Liao D. (2012). Clinical observation of compound Kushen injection combined with cisplatin on malignant pleural effusion. World Health Dig. 9, 49–50. 10.3969/j.issn.1672-5085.2012.42.039

[B7] Chen F. Pan Y. Xu J. Liu B. Song H. (2022). Research progress of matrine's anticancer activity and its molecular mechanism. J. Ethnopharmacol. 286, 114914. 10.1016/j.jep.2021.114914 34919987

[B8] Chen L. (2013). Effect of compound matrine injection on malignant pleural effusion. Chin. J. New Drugs 22, 2069–2070+2074.

[B9] Chen M. He B. (2003). Treatment of carcinomatous hydrothorax with small tube for closing drainage and infusing Bieomycini Hydrochkuridum and Yanshu. J. Minim. Invasive Med. 22, 457–458. 10.3969/j.issn.1673-6575.2003.04.011

[B10] Chen M. H. Gu Y. Y. Zhang A. L. Sze D. M. Mo S. L. May B. H. (2021). Biological effects and mechanisms of matrine and other constituents of Sophora flavescens in colorectal cancer. Pharmacol. Res. 171, 105778. 10.1016/j.phrs.2021.105778 34298110

[B11] Chen X. (2010). Yanshu injection in the treatment of malignant pleural effusion -28 cases study. J. Jiangxi TCM 41, 41–42. 10.3969/j.issn.0411-9584.2010.01.019

[B12] Chen Y. (2009). Kangai injection combined with carboplatin in the treatment of malignant pleural effusion. Heilongjiang J. TCM 38, 27–28.

[B13] Chen Y. Li Q. Xiang S. Yang G. (2011). Compound Kushen Injection combined with cisplatin for malignant pleural effusion:A clinical study. Eval. Anal. Drug-Use Hosp. China 11, 366–367. 10.14009/j.issn.1672-2124.2011.04.016

[B14] Chen Y. Luo Y. Qin Z. (2014). Effect of compound Kushen injection combined with cisplatin on malignant pleural effusion in aged patients. Res. Integr. Tradit. Chin. West Med. 6, 196–197+199. 10.3969/j.issn.1674-4616.2014.04.011

[B15] Cui A. Du C. Yan J. Zhou Q. (2008). Clinical study on the treatment of malignant pleural effusion by pleural catheter drainage and infusion of oxymatrine and carboplatin. J. Baotou Med. 32, 65–66. 10.3969/j.issn.1007-3507.2008.02.001

[B16] Deng M. Wei W. Wang Y. (2008). Observation on efficacy of Yanshu injection combined cisplatin in treating malignant hydrothorax of 40 cases. Chin. Med. Mod. Dist. Edu China 6, 270. 10.3969/j.issn.1672-2779.2008.03.048

[B17] Ding L. Cui C. Xin B. (2009). The clinical observation of compound Kushen injection combined with cisplatin in the treatment of malignant pleural effusion by intrathoracic injection. J. Mod. Oncol. 17, 1274–1275. 10.3969/j.issn.1672-4992.2009.07.026

[B18] Dipper A. Jones H. E. Bhatnagar R. Preston N. J. Maskell N. Clive A. O. (2020). Interventions for the management of malignant pleural effusions: a network meta-analysis. Cochrane Database Syst. Rev. 2020. 10.1002/14651858.cd010529.pub3 PMC7173736 32315458

[B19] Du C. Wang A. Yan S. (2009). Comparison of pleural injection of oxymatrine combined with cisplatin in the treatment of malignant pleural effusion. J. Dis. Monit. Control 3, 472–473.

[B20] Fan Q. Q. She G. M. Wei J. Li Z. Q. Chen M. L. Dong Y. (2022). Anti-tumor and analgesic activity evaluation and mechanism of Compound Kushen Injection. Zhongguo Zhong Yao Za Zhi 47, 2712–2720. 10.19540/j.cnki.cjcmm.20211129.701 35718491

[B21] Feller-Kopman D. J. Reddy C. B. Decamp M. M. Diekemper R. L. Gould M. K. Henry T. (2018). Management of malignant pleural effusions. An official ATS/STS/STR clinical practice guideline. Am. J. Respir. Crit. Care Med. 198, 839–849. 10.1164/rccm.201807-1415ST 30272503

[B22] Feng F. Shi X. (2023). Clinical study of Compound Kushen Injection combined with cisplatin in treatment of non-small cell lung cancer malignant pleural effusion. Drugs and Clin. 38, 1717–1721. 10.7501/j.issn.1674-5515.2023.07.028

[B23] Gayen S. (2022). Malignant pleural effusion: presentation, diagnosis, and management. Am. J. Med. 135, 1188–1192. 10.1016/j.amjmed.2022.04.017 35576996

[B24] Guo L. Luo D. Yang S. (2013). Observation on efficacy of compound Kushen injection combined with cisplatin in the treatment of malignant pleural fluid. China Med. Her. 10, 79–81. 10.3969/j.issn.1673-7210.2013.25.029

[B25] Guo Y. M. Huang Y. X. Shen H. H. Sang X. X. Ma X. Zhao Y. L. (2015). Efficacy of compound kushen injection in relieving cancer-related pain: a systematic review and meta-analysis. Evid. Based Complement. Altern. Med. 2015, 840742. 10.1155/2015/840742 PMC4609400 26504481

[B26] Guyatt G. H. Oxman A. D. Vist G. E. Kunz R. Falck-Ytter Y. Alonso-Coello P. (2008). GRADE: an emerging consensus on rating quality of evidence and strength of recommendations. Bmj 336, 924–926. 10.1136/bmj.39489.470347.AD 18436948 PMC2335261

[B27] Guyot P. Ades A. E. Ouwens M. J. Welton N. J. (2012). Enhanced secondary analysis of survival data: reconstructing the data from published Kaplan-Meier survival curves. BMC Med. Res. Methodol. 12, 9. 10.1186/1471-2288-12-9 22297116 PMC3313891

[B28] Han S. (2013). Effect of compound Kushen injection combined with cisplatin pleural perfusion on malignant pleural effusion. J. Clin. Res. 30, 1191–1193. 10.3969/j.issn.1671-7171.2013.06.055

[B29] Han Z. Tian M. Chen X. Shi F. (2012). Effect of closed drainage combined with compound Kushen injection and cisplatin on malignant pleural effusion of lung cancer. Zhejiang J. ITCWM 22, 524–526. 10.3969/j.issn.1005-4561.2012.07.011

[B30] Hao J. Liang K. (2007). Continuous catheter drainage combined with Yanshu injection and interleukin-2 intrapleural injection in the treatment of malignant pleural effusion. Chin. J. IMCCD 5, 1131–1132. 10.3969/j.issn.1672-1349.2007.11.051

[B31] Hassan M. Harriss E. Mercer R. M. Rahman N. M. (2021). Survival and pleurodesis outcome in patients with malignant pleural effusion - a systematic review. Pleura Perit. 6, 1–5. 10.1515/pp-2020-0147 PMC8223802 34222645

[B32] He J. (2011). Clinical study of Kangai injection combined with cisplatin in treatment of malignant pleural effusion. Med. Inf. 24, 3756–3757. 10.3969/j.issn.1006-1959.2011.08.280

[B33] He L. Chen Z. Wen S. Ren D. Chen H. (2010). Compound Kushen Injection as a local therapy for patients with advanced lung cancer associated with malignant pleural effusion. Eval. Anal. Drug-Use Hosp. China 10, 1025–1027. 10.14009/j.issn.1672-2124.2010.11.012

[B34] He P. Zeng S. Xue Z. Wang Y. Pan B. (2009). Effect of hydroxycamptothecin and Yanshu injection by intrapleural infusion in treatment of 30 cases malignant pleural effusion. J. Mod. Clin. Med. 35, 261–262. 10.3969/j.issn.1673-1557.2009.04.009

[B35] He R. Xie X. (2010). Effect of compound Kushen injection combined with carboplatin on malignant pleural effusion. J. Shandong Med. 50, 27. 10.3969/j.issn.1002-266X.2010.33.017

[B36] He X. Fang J. Huang L. Wang J. Huang X. (2015). Sophora flavescens Ait.: traditional usage, phytochemistry and pharmacology of an important traditional Chinese medicine. J. Ethnopharmacol. 172, 10–29. 10.1016/j.jep.2015.06.010 26087234

[B37] He Y. (2010). Clinical observation of cisplatin combined with oxymatrine in the treatment of malignant pleural effusion. Med. Forum 14, 415–416. 10.3969/j.issn.1672-1721.2010.13.018

[B38] Higgins J. Thomas J. Chandler J. Cumpston M. L. T. Page Mj Welch V. A. (2021). Cochrane handbook for systematic reviews of interventions version 6.2 (updated february 2021). Cochrane. Available online at: www.training.cochrane.org/handbook.

[B39] Hu J. Li Q. Xie Y. (2008a). Intrathoracic administration of Kushenhuangqi injection combined with cisplatin in treatment of malignant pleural efusion. Chin. J. Gen. Pract. 7, 561–563. 10.3760/cma.j.issn.1671-7368.2008.08.027

[B40] Hu Q. Wang H. Pan J. (2008b). Effect of Yanshu injection on malignant pleural effusion of advanced lung cancer. J. Chengdu Univ. TCM 31, 15–17. 10.3969/j.issn.1004-0668.2008.01.006

[B41] Huang H. He Z. Li X. Wang R. (2017). Analysis of curative effect by compound Kushen injection combined with cisplatin through intrapleural infusion in the treatment of malignant pleural effusion. Chin. J. Mod. Drug Appl. 11, 29–31. 10.14164/j.cnki.cn11-5581/r.2017.07.012

[B42] Huang L. (2021). Clinical effect of compound sophora flavescens injection in palliative treatment of advanced lung cancer patients with malignant pleural effusion. Health Manag., 71–72.

[B43] Huang X. (2007). Clinical observation on treating pleural effusion of lung cancer with Yan-shu inject plus cisplatin J. Pract. Chin. Mod. Med. 20 **,** 1106–1106.

[B44] Huang X. (2013). Clinical study on intrapleural compound matrine injection in treating malignant hepatocellular carcinoma with bloody pleural effusion. Acta Med. Sin. 26, 692–693. 10.3969/j.issn.1008-2409.2013.04.010

[B45] Huang Z. Zhang H. Zhang K. Gao L. (2012). The clinical research of compound Kushen injection and corynebacterium parvum as a local therapy for patients with advanced lung cancer associated with malignant pleural effusion. J. Clin. Pulm. Med. 17, 497–498. 10.3969/j.issn.1009-6663.2012.03.056

[B46] Ji F. Shen N. Jin H. (2012). Clinical observation of 82 cases of malignant pleural effusion treated with cisplatin combined with matrine. J. Shandong Med. 52, 92–93. 10.3969/j.issn.1002-266X.2012.29.039

[B47] Ji H. (2011). Clinical study of 30 cases of malignant pleural effusion treated by cisplatin combined with oxymatrine. China Prac. Med. 6, 155–156. 10.3969/j.issn.1673-7555.2011.29.121

[B48] Jiang H. Yang X. M. Wang C. Q. Xu J. Huang J. Feng J. H. (2022). Intrapleural perfusion with staphylococcal enterotoxin C for malignant pleural effusion: a clustered systematic review and meta-analysis. Front. Med. (Lausanne) 9, 816973. 10.3389/fmed.2022.816973 35547209 PMC9081816

[B49] Jiang J. (2014). Clinical effect of compound Kushen Injection combined with cisplatin in treatment of malignant pleural effusion. China Mod. Med. 21, 113–115.

[B50] Jiang T. Li J. (2020). Compound Sophora flavescens injection and cisplatin in the treatment of malignant pleural effusion: a clinical study. Med. and Health 11, 1–2.

[B51] Jie Wang X. Miao K. Luo Y. Li R. Shou T. Wang P. (2018). Randomized controlled trial of endostar combined with cisplatin/pemetrexed chemotherapy for elderly patients with advanced malignant pleural effusion of lung adenocarcinoma. J. buon 23, 92–97. 29552766

[B52] Keeratichananont W. Limthon T. Keeratichananont S. (2015). Efficacy and safety profile of autologous blood versus tetracycline pleurodesis for malignant pleural effusion. Ther. Adv. Respir. Dis. 9, 42–48. 10.1177/1753465815570307 25663279

[B53] Kessinger A. Wigton R. S. (1987). Intracavitary bleomycin and tetracycline in the management of malignant pleural effusions: a randomized study. J. Surg. Oncol. 36, 81–83. 10.1002/jso.2930360202 2443762

[B54] Li B. Yuan Q. Wang Y. Shi M. Ren X. Dong Y. (2019a). Network meta-analysis of 8 traditional Chinese medicine injections combined with cisplatin for malignant pleural effusion. Chin. Hosp. Pharm. J. 39, 1052–1057. 10.13286/j.cnki.chinhosppharmacyj.2019.10.13

[B55] Li H. Ji Y. Zhang S. Gao Z. Hu C. Jiang R. (2019b). Kangai injection combined with platinum-based chemotherapy for the treatment of stage III/IV non-small cell lung cancer: a meta-analysis and systematic review of 35 randomized controlled trials. J. Cancer 10, 5283–5298. 10.7150/jca.31928 31602279 PMC6775612

[B56] Li L. Tian F. (2011). Clinical study of cisplatin compound Kushen injection in treating malignant pleural efusion. China J. Chi Med. 26, 1289–1290. 10.16368/j.issn.1674-8999.2011.11.062

[B57] Li L. Yang F. (2009). Clinical study of oxymatrine injection combined with cisplatin injection in the treatment of malignant pleural effusion. China Prac. Med. 4, 39–41. 10.3969/j.issn.1673-7555.2009.36.023

[B58] Li R. Yu J. Zhang S. Wang A. (2017). Eficacy of compound Kushen Injection combined with nedaplatin for malignant pleurai efusion. Liaoning J. TCM 44, 789–790. 10.13192/j.issn.1000-1719.2017.04.041

[B59] Li S. (2014). Clinical observation of compound Kushen injection combined with nedaplatin in the treatment of malignant pleural effusion. Res. Integr. Tradit. Chin. West Med. 6, 88–89+91. 10.3969/j.issn.1674-4616.2014.02.010

[B60] Li Y. (2008). The clinical value of cisplatin combined with compound Kushen Injection in treatment of cancerours hydrothorax. Mod. Prev. Med. 35, 1978–1979. 10.3969/j.issn.1003-8507.2008.10.078

[B61] Li Y. Chen C. Li Q. (2009). Compound Kushen injection combined with cisplatin pleural injection for lung cancer pleural effusion. China Naturop. 17, 42. 10.3969/j.issn.1007-5798.2009.04.043

[B62] Li Z. (2022). “Network meta-analysis and network pharmacology study of Chinese medicine injection combined with cisplatin in the treatment of malignant pleural effusion of lung cancer,” in Master master. Nanning: Guangxi University of Traditional Chinese Medicine. 10.27879/d.cnki.ggxzy.2022.000202

[B63] Liang N. Kong Z. Lu C. L. Ma S. S. Li Y. Q. Nikolova D. (2019). Radix Sophorae flavescentis versus other drugs or herbs for chronic hepatitis B. Cochrane Database Syst. Rev. 6, Cd013106. 10.1002/14651858.CD013106.pub2 31232459 PMC6589939

[B64] Liang Z. Li X. Liu Z. Li R. Zhai Y. (2011). Efficacy of compound matrine injection for malignant pleural effusion. Eval. Anal. Drug-Use Hosp. China 11, 547–548. 10.14009/j.issn.1672-2124.2011.06.014

[B65] Lin S. Wang Y. Cong X. (2007). Thoracic perfusion of compound Kushen injection plus cisplatin for malignant pleural fluid. Eval. Anal. Drug-Use Hosp. China 7, 465–466. 10.3969/j.issn.1672-2124.2007.06.024

[B66] Lin W. Yuan S. Li F. Yu Y. Liu Y. Deng M. (2023). Effect of intracavitary perfusion of compound Sophora Flavescens injection on the immune function and tumor markers in patients with malignant pleural effusion. Prog. Mod. Biomed. 23, 3758–3762. 10.13241/j.cnki.pmb.2023.19.032

[B67] Liu D. Li D. (2015). Curative effect and nursing of thoracic cavity drainage and compound Kushen injection combined with cisplatin in the treatment of lung cancer patients companying with malignant pleural effusion. J. Clin. Med. Pract. 19, 21–24. 10.7619/jcmp.201508007

[B68] Liu J. Yu Q. Wang X. S. Shi Q. Wang J. Wang F. (2023). Compound kushen injection reduces severe toxicity and symptom burden associated with curative radiotherapy in patients with lung cancer. J. Natl. Compr. Canc Netw. 21, 821–830.e3. 10.6004/jnccn.2023.7036 37549911

[B69] Liu L. Zhong S. Li G. (2017). Effects and safety of compound Kushen injection combined with cisplatin on malignant pleural effusion. J. Mod. Oncol. 25, 230–233. 10.3969/j.issn.1672-4992.2017.02.018

[B70] Liu S. Zhang K. Hu X. (2022). Comparative efficacy and safety of Chinese medicine injections combined with capecitabine and oxaliplatin chemotherapies in treatment of colorectal cancer: a bayesian network meta-analysis. Front. Pharmacol. 13, 1004259. 10.3389/fphar.2022.1004259 36523501 PMC9745148

[B71] Liu X. Xu J. (2016). Therapeutic effects and action mechanism of lobaplatin combined with Kushen injection on malignant pleural effusion in patients with advanced lung cancer. Hebei Med. J. 38, 1461–1464. 10.3969/j.issn.1002-7386.2016.10.005

[B72] Liu Y. Wan L. (2011). Clinical study of compound Kushen injection combined with bleomycin in the treatment of malignant pleural effusion. Chin. Community Doct 13, 178–179. 10.3969/j.issn.1007-614x.2011.09.176

[B73] Ma X. Li R. S. Wang J. Huang Y. Q. Li P. Y. Wang J. (2016). The therapeutic efficacy and safety of compound kushen injection combined with transarterial chemoembolization in unresectable hepatocellular carcinoma: an update systematic review and meta-analysis. Front. Pharmacol. 7, 70. 10.3389/fphar.2016.00070 27065861 PMC4814457

[B74] Miller A. B. Hoogstraten B. Staquet M. Winkler A. (1981). Reporting results of cancer treatment. Cancer 47, 207–214. 10.1002/1097-0142(19810101)47:1<207::aid-cncr2820470134>3.0.co;2-6 7459811

[B75] Ning X. Yang S. Jin Q. (2001). Clinical obversation of treatint 30 cases of cancerous hydrothorax with injection of cisplation and Yanshu. Guid. J. TCMP 7, 70–71. 10.3969/j.issn.1672-951X.2001.02.016

[B76] Page M. J. Mckenzie J. E. Bossuyt P. M. Boutron I. Hoffmann T. C. Mulrow C. D. (2021). The PRISMA 2020 statement: an updated guideline for reporting systematic reviews. Bmj 372, n71. 10.1136/bmj.n71 33782057 PMC8005924

[B77] Paladine W. Cunningham T. J. Sponzo R. Donavan M. Olson K. Horton J. (1976). Intracavitary bleomycin in the management of malignant effusions. Cancer 38, 1903–1908. 10.1002/1097-0142(197611)38:5<1903::aid-cncr2820380506>3.0.co;2-a 62609

[B78] Pan J. Chu D. Hu Z. Sun S. (2007). Clinical observation intracavity Kushen injection combined with cisplatin for the treatment of malignant pleural efusion. Chin. J. Clin. Pharm. 16, 139–141. 10.3969/j.issn.1007-4406.2007.03.002

[B79] Peng H. (2020). Clinical effect of compound sophora flavescens injection in palliative treatment of advanced lung cancer patients with malignant pleural effusion. Health Everyone 27, 595.

[B80] Qin D. Fan K. (2016). Analysis of the therapeutic effect of compound sophora flavescens injection and cisplatin thoracic perfusion therapy in patients with malignant pleural effusion. Med. Health, 257.

[B81] Qu D. Liang X. Zhou B. (2012). Clinical observation of Kangai injection combined with cisplatin in the treatment of malignant pleural effusion. Mod. J. ITCWM 21, 2311–2312. 10.3969/j.issn.1008-8849.2012.21.014

[B82] Ran F. Zang A. (2011). Efficacy of Compound Kushen Injection combined with cisplatin for malignant pleural effusion. Eval. Anal. Drug-Use Hosp. China 11, 739–740. 10.14009/j.issn.1672-2124.2011.08.015

[B83] Shi W. (2017). Clinical efficacy and safety of compound Kushen injection combined with cisplatin in the treatment of malignant pleural effusion caused by lung cancer. Chi J. Conval. Med. 26, 857–859. 10.13517/j.cnki.ccm.2017.08.032

[B84] Song X. Jia Y. (2015). Compound Kushen injection combined with cisplatin pleural perfusion in the treatment of 59 cases of malignant pleural effusion. Forum TCM 30, 30–31. 10.13913/j.cnki.41-1110/r.2015.03.020

[B85] Sterne J. a.C. Savović J. Page M. J. Elbers R. G. Blencowe N. S. Boutron I. (2019). RoB 2: a revised tool for assessing risk of bias in randomised trials. Bmj 366, l4898. 10.1136/bmj.l4898 31462531

[B86] Sun C. Dong F. Xiao T. Gao W. (2021). Efficacy and safety of Chinese patent medicine (Kang-ai injection) as an adjuvant in the treatment of patients with hepatocellular carcinoma: a meta-analysis. Pharm. Biol. 59, 472–483. 10.1080/13880209.2021.1915340 33905666 PMC8081330

[B87] Sun Y. (2012). Bleomycin combined with compound Kushen injection in the treatment of 25 cases of malignant pleural effusion. Zhejiang J. TCM 47, 143. 10.3969/j.issn.0411-8421.2012.02.048

[B88] Tang J. Fang S. Fu S. Liu G. (2014). Meta-analysis of Kang’ai injection combined with cisplatin in the treatment of malignant pleural effusion. Chin. J. Ethnomed Ethnopharm 23, 19–21+25. 10.3969/j.issn.1007-8517.2014.19.zgmzmjyyzz201419013

[B89] Tang X. Jiang M. Li J. Luo B. (2018). Cinical objective on sixty cases of compound Kushen Injection combined with cisplatin in treatment of lung cancer pleural effusion. Liaoning J. TCM 45, 1668–1670. 10.13192/j.issn.1000-1719.2018.08.036

[B90] Thorlund K. Devereaux P. J. Wetterslev J. Guyatt G. Ioannidis J. P. Thabane L. (2009). Can trial sequential monitoring boundaries reduce spurious inferences from meta-analyses? Int. J. Epidemiol. 38, 276–286. 10.1093/ije/dyn179 18824467

[B91] Thorlund K. Engstrøm J. Wetterslev J. Brok J. Imberger G. Gluud C. (2016). User manual for trial sequential analysis (TSA). Copenhagen: Copenhagen Trial Unit, Centre for Clinical Intervention Research. Rigshospitalet.

[B92] Tian X. Wang W. Jia L. (2010). Meta-Analysis of TCM injection in the treatment of malignant pleural effusion. Chin. Med. MDE China 8, 175–178. 10.3969/j.issn.1672-2779.2010.18.133

[B93] Trotti A. Colevas A. D. Setser A. Rusch V. Jaques D. Budach V. (2003). CTCAE v3.0: development of a comprehensive grading system for the adverse effects of cancer treatment. Semin. Radiat. Oncol. 13, 176–181. 10.1016/S1053-4296(03)00031-6 12903007

[B94] Wan Y. M. Li Y. H. Xu Z. Y. Wu H. M. Xu Y. Yang M. (2018). The effect of transarterial chemoembolization in combination with Kang'ai injection on patients with intermediate stage hepatocellular carcinoma: a prospective study. Integr. Cancer Ther. 17, 477–485. 10.1177/1534735417734913 29108428 PMC6041935

[B95] Wang C. Q. Shen Y. S. Chen X. F. Jiang H. Yang X. M. Fan T. Y. (2022). The intrapleural administration with thymic peptides in malignant pleural effusion: a clustered systematicreview and meta-analysis. Int. Immunopharmacol. 107, 108688. 10.1016/j.intimp.2022.108688 35293322

[B96] Wang C. Q. Xu J. Jiang H. Zheng X. T. Zhang Y. Huang X. R. (2023a). The evidence framework of traditional Chinese medicine injection (Aidi injection) in controlling malignant pleural effusion: a clustered systematic review and meta-analysis. Phytomedicine 115, 154847. 10.1016/j.phymed.2023.154847 37149965

[B97] Wang C. Q. Zheng X. T. Chen X. F. Jiang H. Huang J. Jiang Y. (2021). The optimal adjuvant strategy of aidi injection with gemcitabine and cisplatin in advanced non-small cell lung cancer: a meta-analysis of 70 randomized controlled trials. Front. Pharmacol. 12, 582447. 10.3389/fphar.2021.582447 34122057 PMC8194277

[B98] Wang H. (2016). Sophora radix astragali injection combined cisplatin intrathoracic injection clinical observation on treatment of lung cancer hydrothorax. Clin. Res. 24, 2–3.

[B99] Wang R. Chen M. Wang Y. Liu X. Hai L. Shuai B. (2023b). Efficacy and safety of compound Kushen injection in the treatment of malignant pleural effusions caused by breast cancer. China Licens. Pharm. 20, 67–71. 10.3969/j.issn.2096-3327.2023.10.011

[B100] Wang S. Lian X. Sun M. Luo L. Guo L. (2016). Efficacy of compound Kushen injection plus radiotherapy on nonsmall-cell lungcancer: a systematic review and meta-analysis. J. Cancer Res. Ther. 12, 1298–1306. 10.4103/0973-1482.199538 28169243

[B101] Wang S. Zhou W. (2016). Clinical observation of Compound Kushen Injection in the treatment of malignant pleural effusion. China Pract. Med. 11, 148–149. 10.4103/0973-1482.199538

[B102] Wang Y. (2010). Clinical study of compound Kushen injection combined with cisplatin in the treatment of malignant pleural effusion. Chin. J. Mod. Drug Appl. 4, 148–149. 10.3969/j.issn.1673-9523.2010.05.144

[B103] Wang Y. Fan S. Luo Q. Bai C. Li T. (2019). Short term efficacy of pleural cavity drainage and infusion of compound Sophora flavescens injection combined with cisplatin in the treatment of malignant pleural effusion in lung cancer. World Latest Med. Inf. 19, 116+121. 10.19613/j.cnki.1671-3141.2019.02.075

[B104] Wang Y. Lin C. Xiao R. (2010). Effect of closed thoracic drainage with central venous catheters and combining cisplatinum(DDP) with matrine to pure into the thoracic cavity with malignant pleural effusion. Hainan Med. J. 21, 13–15.

[B105] Wei M. Sun Q. (2011). Efficacy of compound Kushen injection combined with cisplatin in the treatment of malignant pleural effusion. JCM 9, 26–27.

[B106] Wei W. Lan Y. Li Y. Zhong B. Li P. Bai L. (2014). A clinical observation of intrathoracic injection of OK-432 combined with compound Kushen injection for patients with malignant pleural effusion. J. Mod. Oncol. 22, 352–354. 10.3969/j.issn.1672-4992.2014.02.33

[B107] Wetterslev J. Thorlund K. Brok J. Gluud C. (2008). Trial sequential analysis may establish when firm evidence is reached in cumulative meta-analysis. J. Clin. Epidemiol. 61, 64–75. 10.1016/j.jclinepi.2007.03.013 18083463

[B108] Wetterslev J. Thorlund K. Brok J. Gluud C. (2009). Estimating required information size by quantifying diversity in random-effects model meta-analyses. BMC Med. Res. Methodol. 9, 86. 10.1186/1471-2288-9-86 20042080 PMC2809074

[B109] Wu C. Wu J. Wu X. Cheng D. (2019). Clinical study of compound radix sophorae flavescentis injection combined with cisplatin in treatment of malignant pleural effusion. Liaoning J. TCM 46, 85–87. 10.13192/j.issn.1000-1719.2019.01.029

[B110] Wu H. Qiu X. Dong Z. Liu H. (2018). Systematic review on the efficacy and safety of adjuvant therapy of compound kushen injection for pleural effusion in elderly patients with malignant cancer. China Pharm. 29, 2421–2425. 10.6039/j.issn.1001-0408.2018.17.27

[B111] Wu Z. Zhang R. Liu Q. Liu G. (2014). Therapeutic effect of hydroxycamptothecin combined with Kushen Injection on malignant pleural effusion in elderly patients with lung cancer Hebei Med. J. 36 **,** 3705–3707. 10.3969/j.issn.1002-7386.2014.24.007

[B112] Xiao Z. Jiang Y. Chen X. F. Wang C. Q. Zheng X. T. Xu W. H. (2020a). Intrathoracic infusion therapy with Lentinan and chemical irritants for malignant pleural effusion: a systematic review and meta-analysis of 65 randomized controlled trials. Phytomedicine 76, 153260. 10.1016/j.phymed.2020.153260 32535483

[B113] Xiao Z. Jiang Y. Wang C. Q. Hu S. S. Huang X. R. Chen X. F. (2020b). Clinical efficacy and safety of aidi injection combination with vinorelbine and cisplatin for advanced non-small-cell lung carcinoma: a systematic review and meta-analysis of 54 randomized controlled trials. Pharmacol. Res. 153, 104637. 10.1016/j.phrs.2020.104637 31935454

[B114] Xiao Z. Wang C. Q. Zhou M. H. Li N. N. Liu S. Y. He Y. J. (2018). Clinical efficacy and safety of CIK plus radiotherapy for lung cancer: a meta-analysis of 16 randomized controlled trials. Int. Immunopharmacol. 61, 363–375. 10.1016/j.intimp.2018.06.012 29945024

[B115] Xing H. (2013). Clinical observation of compound Kushen injection in the treatment of malignant pleural effusion. Chin. J. Mod. Drug Appl. 7, 84–85. 10.14164/j.cnki.cn11-5581/r.2013.17.204

[B116] Xu B. (2014a). Pleural instillation by Yanshu injection and chemotherapy drugs in the treatment of malignant pleural effusion. J. Basic Clin. Oncol. 27, 44–45. 10.3969/j.issn.1673-5412.2014.01.014

[B117] Xu C. Hu Z. Xie M. Liu Y. (2015). Meta-analysis of intrapleural injection of compound kushen injection combined with chemotherapy in treating malignant pleural effusion. China Pharm. 24, 31–32.

[B118] Xu L. (2014b). Efficacy observation of compound Kushen injection and cisplatin for malignant pleural effusion. Contemp. Med. Symp. 12, 88–89. 10.3969/j.issn.2095-7629.2014.03.079

[B119] Xu M. Xiong B. (2008). Intrapleural injection of Kangai zhusheye with cisplatin in malignant pleural efusion. Pract. J. Card. Cereb. Pneum. Vasc. Dis. 16, 114–117. 10.3969/j.issn.1008-5971.2008.02.010

[B120] Xu Y. F. Chen Y. R. Bu F. L. Huang Y. B. Sun Y. X. Li C. Y. (2022). Chinese herbal injections versus intrapleural cisplatin for lung cancer patients with malignant pleural effusion: a Bayesian network meta-analysis of randomized controlled trials. Front. Oncol. 12, 942941. 10.3389/fonc.2022.942941 36203451 PMC9531116

[B121] Yan G. Jiang H. Wang P. Du H. (2016). The application of compound sophora injection combined with cisplatin in the treatment of malignant pleural effusion by pleural perfusion. Jilin Med. J. 37, 574–576. 10.3969/j.issn.1004-0412.2016.03.025

[B122] Yang G. (2012). The recent efficacy of compound Kushen injection combined with cisplafin chest perfusion for malignant pleurai effussion. China Med. 7, 1079–1080.

[B123] Yang M. Ren J. Wen S. Guo C. Pan R. Fu X. (2016). Compound Kushen injection combined with cisplatin for treatment of malignant pleural effusion:A Meta -analysis. J. Mod. Oncol. 24, 3393–3398. 10.3969/j.issn.1672-4992.2016.21.013

[B124] Yang X. Wei X. Jiang L. (2017). Network meta-analysis of 5 kinds of TCM injections in the treatment of malignant pleural effusion. China Pharm. 28, 4686–4690. 10.6039/j.issn.1001-0408.2017.33.22

[B125] Yuan Y. (2007). Thoracentesis and drainage combined with Yanshu injection for malignant pleural effusion. China Pharm. 18, 1891–1892. 10.3969/j.issn.1001-0408.2007.24.023

[B126] Zhang L.-H. Guo Y.-F. Sheng Y.-C. Xie W. Ji R. Mei Q.-B. (2011). Systematic review of platinum antitumor drugs combined with Mannatide in the treatment of malignant pleural effusion. China Pharm. 22, 1691–1695.

[B127] Zhang S. Chen X. Chen Y. Bao L. Zhang T. (2015a). Effiacy and safety of compound Kushen injection plus nedaplatin for malignant pleural effusion in patients with lung cancer. Eval. Anal. Drug-Use Hosp. China 15, 601–603. 10.14009/j.issn.1672-2124.2015.05.015

[B128] Zhang S. Jia Y. Yao L. Yin S. Ye X. (2008). Clinical study of compound Kushen injection combined with cisplatin on malignant pleural effusion. World Health Dig. 5, 1218–1219.

[B129] Zhang X. (2006). Clinical effect of Kangai injection combined with cisplatin on malignant pleural effusion. Liaoning J. TCM 33, 1427–1428. 10.3969/j.issn.1000-1719.2006.11.028

[B130] Zhang X. (2011). Clinical observation of compound sophora flavescens injection in the treatment of cancerous Pleural effusion. J. Chin. Pract. Diagn Ther. 25, 281–282.

[B131] Zhang X. Geng S. Wang Y. (2013). Clinical study of compound Kushen injection combined with mitomycin for malignant pleural efusion. Chin. J. Clin. Oncol. Rehabil. 20, 780–782. 10.13455/j.cnki.cjcor.2013.07.019

[B132] Zhang Z. An J. Zhang F. Yuan Y. (2015b). Effect of compound matrine intracavity perfusion on VEGF, MMP levels in hydrothorax in patients of malignant pleural effusion. Hainan Med. J. 26, 166–168. 10.3969/j.issn.1003-6350.2015.02.0059

[B133] Zheng S. Jia X. (2013). Clinical observation of pleural perfusion in the treatment of malignant pleural effusion. Hebei Med. J. 35, 416–417.

[B134] Zhong B. Wei W. Li Y. Li P. Lan Y. Bai L. (2015). A clinical observation of intrapleural perfusion of OK-432 combined with compound Kushen injection in treatment of malignant pleural effusion. Mod. Med. J. China 17, 8–10. 10.3969/j.issn.1672-9463.2015.03.003

[B135] Zhou Y. Xu X. Zhou P. (2010). Clinical observation on compound matrine injection plus interleukin-2 in the treatment of malignant pleural effusion induced by lung cancer in elder. Chin. J. Clin. Pharm. Ther. 15, 1402–1405.

[B136] Zhu M. Jiang H. Zhu Z. Xu H. (2013). Clinical study of perfusion with compound Kushen injection plus cisplatin for malignant pleural fluid. Tianjin Pharm. 25, 30–32.

[B137] Zhu N. Hou J. (2021). Molecular mechanism of the anti-inflammatory effects of Sophorae Flavescentis Aiton identified by network pharmacology. Sci. Rep. 11, 1005. 10.1038/s41598-020-80297-y 33441867 PMC7806711

[B138] Zhuang H. Ren J. Zhou Y. Chen X. Wu Y. Li F. (2012). Matrine injection combined with intrapleural cisplatin in treatment of 24 patients with hematologic malignancies complicated by pleural effusion. Chin. J. New Drugs 21, 1013–1015. 10.3969/j.issn.1002-7386.2013.03.055

