# Supplementary material for: Intrapleural administration with traditional Chinese medicine injections (Sophorae flavescentis preparations) in controlling malignant pleural effusion: a clustered systematic review and meta-analysis
Source: Front Pharmacol. 2025 Apr 24;16:1519794. doi: 10.3389/fphar.2025.1519794 (PMC12058796; doi:10.3389/fphar.2025.1519794)
Supplement: Supplementary file 1 [file DataSheet2.pdf]

Supplementary materials.3 Retrieval and screening results

1.The retrieval form

(1) The retrieval form for China National Knowledge Infrastructure Database (CNKI) using the “Theme, Title/Abstract and keywords”

(SU%=苦参 OR SU%=复方苦参 OR SU%=岩舒 OR SU%=苦参素 OR SU%=苦参碱 OR SU%=槐定碱 OR SU%=康艾 OR SU%=吗特灵 OR SU%=苦黄 OR SU%=苦参黄芪 OR TKA=苦参 OR TKA=复方苦参 OR TKA=岩舒 OR TKA=苦参素 OR TKA=苦参碱 OR TKA=槐定碱 OR TKA=康艾 OR TKA=吗特灵 OR TKA=苦黄 OR TKA=苦参黄芪) AND (SU%=胸腔积液 OR SU%=胸水 OR SU%=胸腔积水 OR SU%=MPE OR SU%=胸膜腔积液 OR SU%=胸膜炎 OR TKA=胸腔积液 OR TKA=胸水 OR TKA=胸腔积水 OR TKA=MPE OR TKA=胸膜腔积液 OR TKA=胸膜炎)

(2) The retrieval form for Wanfang Database using the “Theme, Title/Abstract and keywords”

(主题:(苦参 OR 复方苦参 OR 岩舒 OR 苦参素 OR 苦参碱 OR 槐定碱 OR 康艾 OR 吗特灵 OR 苦黄 OR 苦参黄芪) OR 题名或关键词:(苦参 OR 复方苦参 OR 岩舒 OR 苦参素 OR 苦参碱 OR 槐定碱 OR 康艾 OR 吗特灵 OR 苦黄 OR 苦参黄芪) OR 摘要:(苦参 OR 复方苦参 OR 岩舒 OR 苦参素 OR 苦参碱 OR 槐定碱 OR 康艾 OR 吗特灵 OR 苦黄 OR 苦参黄芪)) AND (主题:(胸腔积液 OR 胸水 OR 胸腔积水 OR MPE OR 胸膜腔积液 OR 胸膜炎) OR 题名或关键词:(胸腔积液 OR 胸水 OR 胸腔积水 OR MPE OR 胸膜腔积液 OR 胸膜炎) OR 摘要:(胸腔积液 OR 胸水 OR 胸腔积水 OR MPE OR 胸膜腔积液 OR 胸膜炎))

(3) The retrieval form for Chinese Scientific Journals Full-Text Database (VIP) using the “All fields, Title/Abstract and keywords”

(U=苦参 OR U=复方苦参 OR U=岩舒 OR U=苦参素 OR U=苦参碱 OR U=槐定碱 OR U=康艾 OR U=吗特灵 OR U=苦黄 OR U=苦参黄芪 OR M=苦参 OR M=复方苦参 OR M=岩舒 OR M=苦参素 OR M=苦参碱 OR M=槐定碱 OR M=康艾 OR M=吗特灵 OR M=苦黄 OR M=苦参黄芪 OR R=苦参 OR R=复方苦参 OR R=岩舒 OR R=苦参素 OR R=苦参碱 OR R=槐定碱 OR R=康艾 OR R=吗特灵 OR R=苦黄 OR R=苦参黄芪) AND (U=胸腔积液 OR U=胸水 OR U=胸腔积水 OR U=MPE OR U=胸膜腔积液 OR U=胸膜炎 OR M=胸腔积液 OR M=胸水 OR M=胸腔积水 OR M=MPE OR M=胸膜腔积液 OR M=胸膜炎 OR R=胸腔积液 OR R=胸水 OR R=胸腔积水 OR R=MPE OR R=胸膜腔积液 OR R=胸膜炎)

(4) The retrieval form for SinoMed (191) using the “All fields and Title/Abstract”

("苦参"[常用字段:智能] OR "复方苦参"[常用字段:智能] OR "岩舒"[常用字段:智能] OR "苦参素"[常用字段:智能] OR "苦参碱"[常用字段:智能] OR "槐定碱"[常用字段:智能] OR "康艾"[常用字段:智能] OR "吗特灵"[常用字段:智能] OR "苦参"[标题:智能] OR "复方苦参"[标题:智能] OR "岩舒"[标题:智能] OR "苦参素"[标题:智能] OR "苦参碱"[标题:智能] OR "槐定碱"[标题:智能] OR "康艾"[标题:智能] OR "吗特灵"[标题:智能] OR "苦参"[摘要:智能] OR "复方苦参"[摘要:智能] OR "岩舒"[摘要:智能] OR "苦参素"[摘要:智能] OR "苦参碱"[摘要:智能] OR "槐定碱"[摘要:智能] OR "康艾"[摘要:智能] OR "吗特灵"[摘要:智能]) AND ("胸腔积液"[常用字段:智能] OR "胸水"[常用字段:智能] OR "胸腔积水"[常用字段:智能] OR "MPE"[常用字段:智能] OR "胸膜腔积液"[常用字段:智能] OR "胸膜炎"[常用字段:智能] OR "胸腔积液"[标题:

智能] OR "胸水"[标题:智能] OR "胸腔积水"[标题:智能] OR "MPE"[标题:智能] OR "胸膜腔积液"[标题:智能] OR "胸膜炎"[标题:智能] OR "胸腔积液"[摘要:智能] OR "胸水"[摘要:智能] OR "胸腔积水"[摘要:智能] OR "MPE"[摘要:智能] OR "胸膜腔积液"[摘要:智能] OR "胸膜炎"[摘要:智能])

(5) The retrieval form for Guizhou Digital Library using the "Title/Abstract and keywords"

Chinese databases

(T=苦参| T=复方苦参| T=岩舒| T=苦参素| T=苦参碱| T=槐定碱| T=康艾| T=吗特灵| T=苦黄| T=苦参黄芪|K=苦参| K=复方苦参| K=岩舒| K=苦参素| K=苦参碱| K=槐定碱| K=康艾| K=吗特灵| K=苦黄| K=苦参黄芪| S=苦参| S=复方苦参| S=岩舒| S=苦参素| S=苦参碱| S=槐定碱| S=康艾| S=吗特灵| S=苦黄| S=苦参黄芪) * (T=MPE | T=胸腔积水| T=胸水| T=胸腔积液 | T=胸膜腔积液| T=胸膜炎| K=MPE | K=胸腔积水| K=胸水| K=胸腔积液 | K=胸膜腔积液| K=胸膜炎| S=MPE | S=胸腔积水| S=胸水| S=胸腔积液 | S=胸膜腔积液| S=胸膜炎)

(T=苦黄| T=苦参黄芪| K=苦黄| K=苦参黄芪| S=苦黄| S=苦参黄芪) * (T=MPE | T=胸腔积水 | T=胸水| T=胸腔积液 | T=胸膜腔积液| T=胸膜炎| K=MPE | K=胸腔积水| K=胸水| K=胸腔积液 | K=胸膜腔积液| K=胸膜炎| S=MPE | S=胸腔积水| S=胸水| S=胸腔积液 | S=胸膜腔积液 | S=胸膜炎)

English and other databases

(T='Sophora flavescens'|T='Sophora flavescen'|T='kushen'|T='Ku shen'|T='Matrines'|T='Matrine'|T='Yanshu'|T='Yanshu'|T='Yan shu'|T='Kangai'|T='Kang ai'|T='Complex Sophorae'|T='Sophoridine'|T='Kushenin'|T='Oxymatrine'|K='Sophora flavescens'|K='Sophora flavescen'|K='kushen'|K='Ku shen'|K='MaKrines'|K='MaKrine'|K='Yanshu'|K='Yanshu'|K='Yan shu'|K='Kangai'|K='Kang ai'|K='Complex Sophorae'|K='Sophoridine'|K='Kushenin'|K='OxymaKrine'|S='Sophora flavescens'|S='Sophora flavescen'|S='Sushen'|S='Su shen'|S='MaSrines'|S='MaSrine'|S='Yanshu'|S='Yanshu'|S='Yan shu'|S='Sangai'|S='Sang ai'|S='Complex Sophorae'|S='Sophoridine'|S='Sushenin'|S='OxymaSrine')*(T='Pleural Effusion'|T='Pleural Effusions'|T='MPE'|T='MPEs'|T='Pleurisies'|T='Pleuritis'|T='Pleuritides'|T='Pleurisy'|T=' hydrothorax'|K='Pleural Effusion'|K='Pleural Effusions'|K='MPE'|K='MPEs'|K='Pleurisies'|K='Pleuritis'|K='Pleuritides'|K='Pleurisy'|K=' hydrothorax'|S='Pleural Effusion'|S='Pleural Effusions'|S='MPE'|S='MPEs'|S='Pleurisies'|S='Pleuritis'|S='Pleuritides'|S='Pleurisy'|S=' hydrothorax')

(6) The retrieval form for *Cochrane* Central Register of Controlled Trials using the "Title/Abstract keywords"

("Pleural Effusion" OR "Pleural Effusions" OR "MPE" OR "MPEs" OR "Pleurisy" OR "Pleurisies" OR "Pleuritis" OR "Pleuritides" OR "hydrothorax") AND (**"Sophora flavescens" OR** "Sophora flavescen" OR "Kushen" OR "Ku shen" OR "Matrines" OR "Matrine" OR "Yanshu" OR "Yan shu" OR "Kangai" OR "Kang ai" OR "Complex Sophorae" OR "Sophoridine" OR "Kushenin" OR "Oxymatrine")

(7) The retrieval form for Pubmed using the "Mesh, Title, Abstract or free words"

("Pleural Effusion"[Mesh] OR “Pleural Effusion” OR “Pleural Effusions” OR “MPE” OR “MPEs” OR "Pleurisy"[Mesh] OR “Pleurisy” OR “Pleurisies” OR “Pleuritis” OR “Pleuritides” OR "Hydrothorax"[Mesh] OR “hydrothorax”) AND ("Sophora flavescens"[Mesh] OR “Sophora flavescens” ***OR*** “Sophora flavescen” OR "kushen" [Supplementary Concept] OR “Kushen” OR “Ku shen” OR "Matrines" [Supplementary Concept] OR “Matrines” OR “Matrine” OR “Yanshu” OR “Yan shu” OR “Kangai” OR “Kang ai” OR “Sophorae” OR “Sophoridine” OR “Kushenin” OR “Oxymatrine”)

(8) The retrieval form for Embase using the “Title or Abstract or All fields”

(“Pleural Effusion” OR “Pleural Effusions” OR “MPE” OR “MPEs” OR “Pleurisy” OR “Pleurisies” OR “Pleuritis” OR “Pleuritides” OR “hydrothorax”) AND (“Sophora flavescens” OR “Sophora flavescen” OR “Kushen” OR “Ku shen” OR “Matrines” OR “Matrine” OR “Yanshu” OR “Yan shu” OR “Kangai” OR “Kang ai” OR “Complex Sophorae” OR “Sophoridine” OR “Kushenin” OR “Oxymatrine”)

(9) The retrieval form for Web of science using the “Title, Abstract, and Index”

(“Pleural Effusion” OR “Pleural Effusions” OR “MPE” OR “MPEs” OR “Pleurisy” OR “Pleurisies” OR “Pleuritis” OR “Pleuritides” OR “hydrothorax”) AND (“Sophora flavescens” OR “Sophora flavescen” OR “Kushen” OR “Ku shen” OR “Matrines” OR “Matrine” OR “Yanshu” OR “Yan shu” OR “Kangai” OR “Kang ai” OR “Complex Sophorae” OR “Sophoridine” OR “Kushenin” OR “Oxymatrine”)

2. Retrieval results

Table S1. Identified 6 records from Pubmed

Search	Query	Results
#58	Search: (((((((((((((((((((((((((((((((((("Sophora flavescens"[Mesh]) OR ("Sophora flavescens")) OR ("Sophora flavescens"[Title/Abstract])) OR ("Sophora flavescen"[Title/Abstract])) OR ("Sophora flavescen")) OR ("kushen" [Supplementary Concept])) OR ("Kushen"[Title/Abstract])) OR ("Kushen")) OR ("Ku shen")) OR ("Ku shen")) OR ("Matrines" [Supplementary Concept])) OR ("Matrines"[Title/Abstract])) OR ("Matrines")) OR ("Matrine")) OR ("Matrine"[Title/Abstract])) OR ("Yanshu" [Title/Abstract])) OR ("Yanshu")) OR ("Yan shu")) OR ("Yan shu"[Title/Abstract] AND ("schema"[All Fields] OR "schema s"[All Fields] OR "schemas"[All Fields]))) OR ("Kangai" [Title/Abstract])) OR ("Yan shu"[Title/Abstract])) OR ("Kangai")) OR ("Kang ai")) OR ("Kang ai"[Title/Abstract])) OR ("Sophorae"[Title/Abstract])) OR ("Sophorae")) OR ("Sophoridine")) OR ("Sophoridine"[Title/Abstract])) OR ("Kushenin"[Title/Abstract])) OR ("Kushenin")) OR ("Oxymatrine")) OR ("Oxymatrine"[Title/Abstract])) OR ("kuhuang")) OR ("kuhuang"[Title/Abstract])) AND ((((((((((((((((((((("Pleural Effusion"[Mesh]) OR ("Pleural Effusion")) OR ("Pleural Effusions")) OR ("MPE")) OR ("MPEs")) OR ("Pleurisy"[Mesh])) OR ("Pleurisy")) OR ("Pleurisies")) OR ("Pleuritis")) OR ("Pleuritides")) OR ("Hydrothorax"[Mesh])) OR ("hydrothorax")) OR ("Pleural Effusion"[Title/Abstract])) OR ("Pleural Effusions"[Title/Abstract])) OR ("MPE"[Title/Abstract])) OR ("MPEs" [Title/Abstract])) OR ("Pleurisy"[Title/Abstract])) OR ("Pleurisies" [Title/Abstract])) OR ("Pleuritis" [Title/Abstract])) OR ("Pleuritides"[Title/Abstract])) OR ("hydrothorax"[Title/Abstract]))	6
#57	Search: ((((((((((((((((((((((((((((((((("Sophora flavescens"[Mesh]) OR ("Sophora flavescens")) OR ("Sophora flavescens"[Title/Abstract])) OR ("Sophora flavescen"[Title/Abstract])) OR ("Sophora flavescen")) OR ("kushen" [Supplementary Concept])) OR ("Kushen"[Title/Abstract])) OR ("Kushen")) OR ("Ku shen")) OR ("Ku shen")) OR ("Matrines" [Supplementary Concept])) OR ("Matrines"[Title/Abstract])) OR ("Matrines")) OR ("Matrine")) OR ("Matrine"[Title/Abstract])) OR ("Yanshu" [Title/Abstract])) OR ("Yanshu")) OR ("Yan shu")) OR ("Yan shu"[Title/Abstract] AND ("schema"[All Fields] OR "schema s"[All Fields] OR "schemas"[All Fields]))) OR ("Kangai" [Title/Abstract])) OR ("Yan shu"[Title/Abstract])) OR ("Kangai")) OR ("Kang ai")) OR ("Kang ai"[Title/Abstract])) OR ("Sophorae"[Title/Abstract])) OR ("Sophorae")) OR ("Sophoridine")) OR ("Sophoridine"[Title/Abstract])) OR ("Kushenin"[Title/Abstract])) OR ("Kushenin")) OR ("Oxymatrine")) OR ("Oxymatrine"[Title/Abstract])) OR ("kuhuang")) OR ("kuhuang"[Title/Abstract])	

#56	Search: "kuhuang"[Title/Abstract]	9
#55	Search: "kuhuang"	9
#54	Search: "Oxymatrine"[Title/Abstract]	696
#53	Search: "Oxymatrine"	715
#52	Search: "Kushenin"	11
#51	Search: "Kushenin"[Title/Abstract]	11
#50	Search: "Sophoridine"[Title/Abstract]	162
#49	Search: "Sophoridine"	162
#48	Search: "Sophorae"	356
#47	Search: "Sophorae"[Title/Abstract]	356
#46	Search: "Kang ai"[Title/Abstract]	45
#45	Search: "Kang ai"	54
#44	Search: "Kangai"	583
#43	Search: "Yan shu"[Title/Abstract]	1
#42	Search: "Kangai" [Title/Abstract]	99
#41	Search: "Yan shu"[Title/Abstract] - Schema: all	0
#40	Search: "Yan shu"	161
#39	Search: "Yanshu"	35
#38	Search: "Yanshu" [Title/Abstract]	17
#37	Search: "Matrine"[Title/Abstract]	1,164
#36	Search: "Matrine"	1,165
#35	Search: "Matrines"	844
#34	Search: "Matrines"[Title/Abstract]	35
#33	Search: "Matrines" [Supplementary Concept]	834
#32	Search: "Ku shen"[Title/Abstract]	20
#31	Search: "Ku shen"	20
#30	Search: "Kushen"	264
#29	Search: "Kushen"[Title/Abstract]	254
#28	Search: "kushen" [Supplementary Concept]	94
#27	Search: "Sophora flavescen"	1
#26	Search: "Sophora flavescen"[Title/Abstract]	1
#25	Search: "Sophora flavescens"[Title/Abstract]	775
#24	Search: "Sophora flavescens"	776
#23	Search: "Sophora flavescens"[Mesh]	36
#22	Search: ((((((((((((((((((("Pleural Effusion"[Mesh]) OR ("Pleural Effusion")) OR ("Pleural Effusions")) OR ("MPE")) OR ("MPEs")) OR ("Pleurisy"[Mesh])) OR ("Pleurisy")) OR ("Pleurisies")) OR ("Pleuritis")) OR ("Pleuritides")) OR ("Hydrothorax"[Mesh])) OR ("hydrothorax")) OR ("Pleural Effusion"[Title/Abstract])) OR ("Pleural Effusions"[Title/Abstract])) OR ("MPE"[Title/Abstract])) OR ("MPEs" [Title/Abstract])) OR ("Pleurisy"[Title/Abstract])) OR ("Pleurisies"	50,784

	[Title/Abstract])) OR ("Pleuritis" [Title/Abstract])) OR ("Pleuritides"[Title/Abstract])) OR ("hydrothorax"[Title/Abstract])	
#21	Search: "hydrothorax"[Title/Abstract]	2,176
#20	Search: "Pleuritides"[Title/Abstract]	1
#19	Search: "Pleuritis" [Title/Abstract]	2,773
#18	Search: "Pleurisies" [Title/Abstract]	141
#17	Search: "Pleurisy"[Title/Abstract]	5,187
#16	Search: "MPEs" [Title/Abstract]	430
#15	Search: "MPE"[Title/Abstract]	2,967
#14	Search: "Pleural Effusions"[Title/Abstract]	8,787
#13	Search: "Pleural Effusion"[Title/Abstract]	25,226
#12	Search: "hydrothorax"	2,526
#11	Search: "Hydrothorax"[Mesh]	1,408
#10	Search: "Pleuritides"	2
#9	Search: "Pleuritis"	2,904
#8	Search: "Pleurisies"	141
#7	Search: "Pleurisy"	7,493
#6	Search: "Pleurisy"[Mesh]	5,946
#5	Search: "MPEs"	442
#4	Search: "MPE"	3,464
#3	Search: "Pleural Effusions"	8,790
#2	Search: "Pleural Effusion"	36,446
#1	Search: "Pleural Effusion"[Mesh]	22,097

Table S2. Identified 191 records from SinoMed

Search	Query	Results
45	(("胸膜炎"[摘要:智能]) OR ("胸膜腔积液"[摘要:智能]) OR ("MPE"[摘要:智能]) OR ("胸腔积水"[摘要:智能]) OR ("胸水"[摘要:智能]) OR ("胸腔积液"[摘要:智能]) OR ("胸膜炎"[标题:智能]) OR ("胸膜腔积液"[标题:智能]) OR ("MPE"[标题:智能]) OR ("胸腔积水"[标题:智能]) OR ("胸水"[标题:智能]) OR ("胸腔积液"[标题:智能]) OR ("胸膜炎"[常用字段:智能]) OR ("胸膜腔积液"[常用字段:智能]) OR ("MPE"[常用字段:智能]) OR ("胸腔积水"[常用字段:智能]) OR ("胸水"[常用字段:智能]) OR ("胸腔积液"[常用字段:智能])) AND (("吗特灵"[摘要:智能]) OR ("康艾"[摘要:智能]) OR ("槐定碱"[摘要:智能]) OR ("苦参碱"[摘要:智能]) OR ("苦参素"[摘要:智能]) OR ("岩舒"[摘要:智能]) OR ("复方苦参"[摘要:智能]) OR ("苦参"[摘要:智能]) OR ("吗特灵"[标题:智能]) OR ("康艾"[标题:智能]) OR ("槐定碱"[标题:智能]) OR ("苦参碱"[标题:智能]) OR ("苦参素"[标题:智能]) OR ("岩舒"[标题:智能]) OR ("复方苦参"[标题:智能]) OR ("苦参"[标题:智能]) OR ("吗特灵"[常用字段:智能]) OR ("康艾"[常用字段:智能]) OR ("槐定碱"[常用字段:智能]) OR ("苦参碱"[常用字段:智能]) OR ("苦参素"[常用字段:智能]) OR ("岩舒"[常用字段:智能]) OR ("复方苦参"[常用字段:智能]) OR ("苦参"[常用字段:智能]))	197
44	("胸膜炎"[摘要:智能]) OR ("胸膜腔积液"[摘要:智能]) OR ("MPE"[摘要:智能]) OR ("胸腔积水"[摘要:智能]) OR ("胸水"[摘要:智能]) OR ("胸腔积液"[摘要:智能]) OR ("胸膜炎"[标题:智能]) OR ("胸膜腔积液"[标题:智能]) OR ("MPE"[标题:智能]) OR ("胸腔积水"[标题:智能]) OR ("胸水"[标题:智能]) OR ("胸腔积液"[标题:智能]) OR ("胸膜炎"[常用字段:智能]) OR ("胸膜腔积液"[常用字段:智能]) OR ("MPE"[常用字段:智能]) OR ("胸腔积水"[常用字段:智能]) OR ("胸水"[常用字段:智能]) OR ("胸腔积液"[常用字段:智能])	89,198
43	"胸膜炎"[摘要:智能]	10,909
42	"胸膜腔积液"[摘要:智能]	230
41	"MPE"[摘要:智能]	3,389
40	"胸腔积水"[摘要:智能]	53
39	"胸水"[摘要:智能]	9,249
38	"胸腔积液"[摘要:智能]	47,040
37	"胸膜炎"[标题:智能]	7,915
36	"胸膜腔积液"[标题:智能]	86
35	"MPE"[标题:智能]	61
34	"胸腔积水"[标题:智能]	5
33	"胸水"[标题:智能]	4,862
32	"胸腔积液"[标题:智能]	22,854
31	"胸膜炎"[常用字段:智能]	18,888
30	"胸膜腔积液"[常用字段:智能]	309
29	"MPE"[常用字段:智能]	3,407
28	"胸腔积水"[常用字段:智能]	60
27	"胸水"[常用字段:智能]	12,176

26	"胸腔积液"[常用字段:智能]	69,072
25	("吗特灵"[摘要:智能]) OR ("康艾"[摘要:智能]) OR ("槐定碱"[摘要:智能]) OR ("苦参碱"[摘要:智能]) OR ("苦参素"[摘要:智能]) OR ("岩舒"[摘要:智能]) OR ("复方苦参"[摘要:智能]) OR ("苦参"[摘要:智能]) OR ("吗特灵"[标题:智能]) OR ("康艾"[标题:智能]) OR ("槐定碱"[标题:智能]) OR ("苦参碱"[标题:智能]) OR ("苦参素"[标题:智能]) OR ("岩舒"[标题:智能]) OR ("复方苦参"[标题:智能]) OR ("苦参"[标题:智能]) OR ("吗特灵"[常用字段:智能]) OR ("康艾"[常用字段:智能]) OR ("槐定碱"[常用字段:智能]) OR ("苦参碱"[常用字段:智能]) OR ("苦参素"[常用字段:智能]) OR ("岩舒"[常用字段:智能]) OR ("复方苦参"[常用字段:智能]) OR ("苦参"[常用字段:智能])	17,463
24	"吗特灵"[摘要:智能]	14
23	"康艾"[摘要:智能]	905
22	"槐定碱"[摘要:智能]	561
21	"苦参碱"[摘要:智能]	5,436
20	"苦参素"[摘要:智能]	1,701
19	"岩舒"[摘要:智能]	2,022
18	"复方苦参"[摘要:智能]	2,206
17	"苦参"[摘要:智能]	12,573
16	"吗特灵"[标题:智能]	39
15	"康艾"[标题:智能]	752
14	"槐定碱"[标题:智能]	296
13	"苦参碱"[标题:智能]	4,266
12	"苦参素"[标题:智能]	1,765
11	"岩舒"[标题:智能]	1,913
10	"复方苦参"[标题:智能]	2,103
9	"苦参"[标题:智能]	10,128
8	"吗特灵"[常用字段:智能]	45
7	"康艾"[常用字段:智能]	936
6	"槐定碱"[常用字段:智能]	591
5	"苦参碱"[常用字段:智能]	7,637
4	"苦参素"[常用字段:智能]	2,087
3	"岩舒"[常用字段:智能]	2,173
2	"复方苦参"[常用字段:智能]	2,459
1	"苦参"[常用字段:智能]	15,303

3. List of eligible and ineligible studies from systematic review and meta-analysis

In all, we included 42 eligible studies [1-42] and excluded 16 ineligible studies[43-58] after evaluating previous 10 SRs/meta analyses [59-68].

Table S3.The included trials from the systematic review and meta-analysis

**Authors**	**Title**	**Included trials**	**Included Trials from previous 10 SRs/meta analyses**	**Journals**
Tian, X. 2010	Meta-Analysis of TCM injection In the treatment of malignant pleural effusion	19trials	One trial [4]	Chin Med MDE China **8**(18): 175-178.
Tang, J. 2014	Meta-analysis of Kang'ai Injection combined with Cisplatin in the Treatment of Malignant Pleural Effusion	9 trials	Four trials[1, 6, 14, 21]	Chin J Ethnomed Ethnopharm **23**(19): 19-21+25.
Xu, C. 2015	Meta-Analysis of Intrapleural Injection of Compound Kushen Injection Combined with Chemotherapy in Treating Malignant Pleural Effusion	11 trials	Nine trials[13, 16-18, 22, 26, 29, 33, 34]	China Pharmaceuticals **24**(18): 31-32,33.
Yang, M.2016	Compound Kushen injection combined with cisplatin for treatment of malignant pleural effusion:A Meta -analysis	18 trials	15 trials[7, 8, 10, 11, 13, 17-19, 23, 26, 27, 30-33]	J Modern Oncol **24**(21): 3393-3398.
Yang, X.2017	Network Meta-analysis of 5 Kinds of TCM Injections in the Treatment of Malignant Pleural Effusion	6 trials	Three trials[15, 25, 28]	China Pharmacy **28**(33): 4686-4690
Wu, H.2018	Systematic Review on the Efficacy and Safety of Adjuvant Therapy of Compound Kushen Injection for Pleural Effusion in Elderly Patients with Malignant Cancer	7 trials	Three trials [12, 32, 35]	China Pharmacy **29**(17): 2421-2425.
Li, B . 2019	Network meta-analysis of 8 traditional Chinese medicine injections combined with cisplatin for malignant pleural effusion	92 trials	Two trials[1, 24]	CHin Hosp Pharm J 39(10): 1052-1057.
Li, Z. 2022	Network meta-analysis and network pharmacology study of Chinese medicine injection combined with cisplatin in the treatment of malignant pleural effusion of lung cancer	30 trials	Nine trials [9, 10, 19, 36-41]	Ruikang Clinical Medical College. Nan Ning, Guangxi University of Traditional Chinese Medicine. Master.
Biaoxue, R,2015	Thoracic perfusion of matrine as an adjuvant treatment improves the control of the malignant pleural effusions	12 trials	Six trials [13, 15, 20, 25, 29, 35]	World J Surg Oncol 13: 329.
Xu, Y. F. 2022	Chinese herbal injections versus intrapleural cisplatin for lung cancer patients with malignant pleural effusion: A Bayesian network meta-analysis of randomized controlled trials	15 trials	15trials[2, 3, 5, 9, 10, 14, 19, 21, 28, 36-39, 41, 42]	Front Oncol 12: 942941.

Table S4. We excluded 16 ineligible studies [43-58] from previous SR/Meta-analysis

Authors	Title	Included trials	Excluded 16 ineligible studies from previous SR/Meta-analysis	Journals
Tang, J. 2014	Meta-analysis of Kang'ai Injection combined with Cisplatin in the Treatment of Malignant Pleural Effusion	9 trials	Non-randomized controlled trial [54, 55]<br>Intravenous infusion [56-58]	Chin J Ethnomed Ethnopharm **23**(19): 19-21+25.
Xu, C. 2015	Meta-Analysis of Intrapleural Injection of Compound Kushen Injection Combined with Chemotherapy in Treating Malignant Pleural Effusion	11 trials	Non-randomized controlled trial [52, 53]	China Pharmaceuticals **24**(18): 31-32,33.
Yang, M.2016	Compound Kushen injection combined with cisplatin for treatment of malignant pleural effusion:A Meta -analysis	18 trials	Non-randomized controlled trial [47, 52, 53]	J Modern Oncol **24**(21): 3393-3398.
Wu, H.2018	Systematic Review on the Efficacy and Safety of Adjuvant Therapy of Compound Kushen Injection for Pleural Effusion in Elderly Patients with Malignant Cancer	7 trials	Non-randomized controlled trial [43]; RCTs with unclear objectives [44, 45]; Duplicates [46]	China Pharmacy **29**(17): 2421-2425.
Biaoxue, R, 2015	Thoracic perfusion of matrine as an adjuvant treatment improves the control of the malignant pleural effusions	12 trials	Non-randomized controlled trial [47]; Non intrapleural perfusion[48, 49]; RCTs with unclear objectives[45, 50, 51]	World J Surg Oncol 13: 329.

[1] Zhang X. Clinical effect of Kangai injection combined with cisplatin on malignant pleural effusion. Liaoning J TCM, 2006, 33(11): 1427-1428.

[2] Huang X. Clinical observation on treating pleural effusion of lung cancer with Yan-shu inject plus cisplatin J Pract Chin Modern Med, 2007, 20(12): 1106-1106,1108.

[3] Pan J, Chu D, Hu Z*, et al.* Clinical observation intracavity Kushen injection combined with cisplatin for the treatment of malignant pleural efusion. Chin J Clin Pharm, 2007, 16(3): 139-141.

[4] Yuan Y. Thoracentesis and drainage combined with Yanshu injection for malignant pleural effusion. China Pharm, 2007, 18(24): 1891-1892.

[5] Hu Q, Wang H, Pan J. Effect of Yanshu injection on malignant pleural effusion of advanced lung cancer. J Chengdu Univ TCM, 2008, 31(1): 15-17.

[6] Xu M, Xiong B. Intrapleural injection of Kangai zhusheye with cisplatin in malignant pleural efusion. Pract J Cardiac Cereb Pneum Vasc Dis, 2008, 16(2): 114-117.

[7] Zhang S, Jia Y, Yao L*, et al.* Clinical study of compound Kushen injection combined with cisplatin on malignant pleural effusion. World Health Digest, 2008, 5(8): 1218-1219.

[8] Ding L, Cui C, Xin B. The clinical observation of compound Kushen injection combined with cisplatin in the treatment of malignant pleural effusion by intrathoracic injection. J Modern Oncol, 2009, 17(7): 1274-1275.

[9] Li Y, Chen C, Li Q. Compound Kushen injection combined with cisplatin pleural injection for lung cancer pleural effusion. China Naturopathy, 2009, 17(4): 42-42.

[10] He L, Chen Z, Wen S*, et al.* Compound Kushen Injection as a local therapy for patients with advanced lung cancer associated with malignant pleural effusion. Eval Anal Drug-Use Hosp China, 2010, 10(11): 1025-1027.

[11] Wang Y. Clinical study of compound Kushen injection combined with cisplatin in the treatment of

malignant pleural effusion. Chin J Mod Drug Appl, 2010, 4(5): 148-149.
[12] Zhou Y, Xu X, Zhou P. Clinical observation on compound matrine injection plus interleukin-2 in the treatment of malignant pleural effusion induced by lung cancer in elder. Chin J Clin Pharm Ther, 2010, 15(12): 1402-1405.
[13] Chen Y, Li Q, Xiang S*, et al.* Compound Kushen Injection combined with cisplatin for malignant pleural effusion：A clinical study. Eval Anal Drug-Use Hosp China, 2011, 11(4): 366-367.
[14] He J. Clinical study of Kangai injection combined with cisplatin in treatment of malignant pleural effusion. Med Inform, 2011, 24(8): 3756-3757.
[15] Liang Z, Li X, Liu Z*, et al.* Efficacy of compound matrine injection for malignant pleural effusion. Eval Anal Drug-Use Hosp China, 2011, 11(6): 547-548.
[16] Liu Y, Wan L. Clinical study of compound Kushen injection combined with bleomycin in the treatment of malignant pleural effusion. Chin Community Doct, 2011, 13(9): 178-179.
[17] Ran F, Zang A. Efficacy of Compound Kushen Injection combined with cisplatin for malignant pleural effusion. Eval Anal Drug-Use Hosp China, 2011, 11(8): 739-740.
[18] Wei M, Sun Q. Efficacy of compound Kushen injection combined with cisplatin in the treatment of malignant pleural effusion. JCM, 2011, 9(2): 26-27.
[19] Han Z, Tian M, Chen X*, et al.* Effect of closed drainage combined with compound Kushen injection and cisplatin on malignant pleural effusion of lung cancer. Zhejiang J ITCWM, 2012, 22(7): 524-526.
[20] Huang Z, Zhang H, Zhang K*, et al.* The clinical research of compound Kushen injection and corynebacterium parvum as a local therapy for patients with advanced lung cancer associated with malignant pleural effusion. J Clin Pulm Med, 2012, 17(3): 497-498.
[21] Qu D, Liang X, Zhou B. Clinical observation of Kangai injection combined with cisplatin in the treatment of malignant pleural effusion. Modern J ITCWM, 2012, 21(21): 2311-2312.
[22] Sun Y. Bleomycin combined with compound Kushen injection in the treatment of 25 cases of malignant pleural effusion. Zhejiang J TCM, 2012, 47(2): 143-143.
[23] Yang G. The recent efficacy of compound Kushen injection combined with cisplafin chest perfusion for malignant pleurai effussion. China Med, 2012, 7(9): 1079-1080.
[24] Zhuang H, Ren J, Zhou Y*, et al.* Matrine injection combined with intrapleural cisplatin in treatment of 24 patients with hematologic malignancies complicated by pleural effusion. Chin J New Drugs, 2012, 21(9): 1013-1015.
[25] Chen L. Effect of compound matrine injection on malignant pleural effusion. Chin J New Drugs, 2013, 22(17): 2069-2070+2074.
[26] Guo L, Luo D, Yang S. Observation on efficacy of compound Kushen injection combined with cisplatin in the treatment of malignant pleural fluid. China Med Herald, 2013, 10(25): 79-81.
[27] Han S. Effect of compound Kushen injection combined with cisplatin pleural perfusion on malignant pleural effusion. J Clin Res, 2013, 30(6): 1191-1193.
[28] Xing H. Clinical observation of compound Kushen injection in the treatment of malignant pleural effusion. Chin J Mod Drug Appl, 2013, 7(17): 84-85.
[29] Zhang X, Geng S, Wang Y. Clinical study of compound Kushen injection combined with mitomycin for malignant pleural efusion. Chin J Clin Oncol Rehabil, 2013, 20(7): 780-782.
[30] Zheng S, Jia X. Clinical observation of pleural perfusion in the treatment of malignant pleural effusion. Hebei Med J, 2013, 35(3): 416-417.
[31] Zhu M, Jiang H, Zhu Z*, et al.* Clinical study of perfusion with compound Kushen injection plus

cisplatin for malignant pleural fluid. Tianjin Pharm, 2013, 25(6): 30-32.
[32] Chen Y, Luo Y, Qin Z. Effect of compound Kushen injection combined with cisplatin on malignant pleural effusion in aged patients. Res Integr Tradit Chin West Med, 2014, 6(4): 196-197+199.
[33] Jiang J. Clinical effect of compound Kushen Injection combined with cisplatin in treatment of malignant pleural effusion. China Modern Med, 2014, 21(26): 113-115.
[34] Li S. Clinical observation of compound Kushen injection combined with nedaplatin in the treatment of malignant pleural effusion. Res Integr Tradit Chin West Med, 2014, 6(2): 88-89+91.
[35] Wu Z, Zhang R, Liu Q*, et al.* Therapeutic effect of hydroxycamptothecin combined with Kushen Injection on malignant pleural effusion in elderly patients with lung cancer Hebei Med J, 2014, 36(24): 3705-3707.
[36] Liu D, Li D. Curative effect and nursing of thoracic cavity drainage and compound Kushen injection combined with cisplatin in the treatment of lung cancer patients companying with malignant pleural effusion. J Clin Med Pract, 2015, 19(8): 21-24.
[37] Liu L, Zhong S, Li G. Effects and safety of compound Kushen injection combined with cisplatin on malignant pleural effusion. J Modern Oncol, 2017, 25(2): 230-233.
[38] Shi W. Clinical efficacy and safety of compound Kushen injection combined with cisplatin in the treatment of malignant pleural effusion caused by lung cancer. Chi J Convalescent Med, 2017, 26(8): 857-859.
[39] Tang X, Jiang M, Li J*, et al.* Cinical objective on sixty cases of compound Kushen Injection combined with cisplatin in treatment of lung cancer pleural effusion. Liaoning J TCM, 2018, 45(8): 1668-1670.
[40] Wang Y, Fan S, Luo Q*, et al.* Short term efficacy of pleural cavity drainage and infusion of compound Sophora flavescens injection combined with cisplatin in the treatment of malignant pleural effusion in lung cancer. World Latest Med Inform, 2019, 19(02): 116+121.
[41] Wu C, Wu J, Wu X*, et al.* Clinical study of compound radix sophorae flavescentis injection combined with cisplatin in treatment of malignant pleural effusion. Liaoning J TCM, 2019, 46(1): 85-87.
[42] Peng H. Clinical effect of compound sophora flavescens injection in palliative treatment of advanced lung cancer patients with malignant pleural effusion. Health for Everyone, 2020, 27(14): 595-595.
[43] 周永清, 陈旭, 丁海斌. 复方苦参注射液 IL-2 及干扰素辅助治疗肺癌伴恶性胸腔积液疗效和不良反应. 西部医学, 2016, 28(9): 1261-1263,1270.
[44] 李玮, 任华, 朱金如*, et al.* 老年恶性胸腔积液药物联合治疗临床观察. 中国医院用药评价与分析, 2011, 11(7): 636-638.
[45] 夏国安. 博莱霉素联合复方苦参注射液治疗老年肺癌恶性胸腔积液疗效观察. 安徽医药, 2013, 17(1): 131-132.
[46] 林振怀, 孙砚诚, 王永仓. 复方苦参注射液联合白细胞介素 2 治疗老年肺癌恶性胸腔积液的临床观察. 中国医药指南, 2011, 9(7): 63-65.
[47] 周良发, 胡作为, 徐涛. 复方苦参注射液联合顺铂治疗恶性胸腔积液的近期疗效观察. 贵阳中医学院学报, 2012, 34(5): 145-147.
[48] 刘思杰, 刘宏, 边建伟. 复方苦参注射液联合白介素-II、顺铂治疗恶性胸腔积液的近期疗效观察. 中国现代医药杂志, 2011, 13(7): 42-44.
[49] 江雨顺, 杨杰. 周剂量顺铂胸腔灌注联合复方苦参注射液治疗非小细胞肺癌并恶性胸腔积液的临床研究. 现代医药卫生, 2013, 29(11): 1619-1620.

[50] 郭志强. 复方苦参注射液及白介素-2 治疗恶性胸水的疗效观察. 河南医学研究, 2013, 22(4): 499-501.
[51] 胡代菊, 梅晓冬. 复方苦参注射液、IL-2 及 α-干扰素治疗肺癌伴恶性胸腔积液疗效. 临床肺科杂志, 2012, 17(10): 1844-1845.
[52] 裴锋. 复方苦参注射液联合顺铂治疗恶性胸腔积液的疗效观察. 时珍国医国药, 2012, 23(6): 1580.
[53] 汤伟忠. 复方苦参注射液加顺铂治疗晚期恶性胸腔积液的观察. 中国医院用药评价与分析, 2009, 9(7): 538-539.
[54] 冯青, 蔡正凤. 康艾注射液联合顺铂治疗恶性胸腔积液. 中国民族民间医药, 2010, 19(09): 25+32.
[55] 刘明伟. 康艾注射液治疗肺癌胸水的临床研究. 河南职工医学院学报, 2011, 23(06): 677-678.
[56] 宋春燕, 许士珍, 王翠英. 康艾注射液联合热化疗治疗恶性胸腔积液 45 例. 陕西中医, 2013, 34(12): 1586-1587.
[57] 许士珍, 宋春燕, 王翠英. 康艾注射液联合热化疗治疗恶性胸腔积液 80 例. 临床荟萃, 2013, 28(11): 1278-1279.
[58] 谢新梅, 张洪亮, 肖春霞. 康艾注射液联合顺铂治疗恶性胸腔积液 32 例. 陕西中医, 2011, 32(10): 1329-1331.
[59] Xu YF, Chen YR, Bu FL*, et al.* Chinese herbal injections versus intrapleural cisplatin for lung cancer patients with malignant pleural effusion: A Bayesian network meta-analysis of randomized controlled trials. Front Oncol, 2022, 12: 942941.
[60] Li Z. Network meta-analysis and network pharmacology study of Chinese medicine injection combined with cisplatin in the treatment of malignant pleural effusion of lung cancer. Ruikang Clinical Medical College, 2022, Master.
[61] Li B, Yuan Q, Wang Y*, et al.* Network meta-analysis of 8 traditional Chinese medicine injections combined with cisplatin for malignant pleural effusion. CHin Hosp Pharm J, 2019, 39(10): 1052-1057.
[62] Wu H, Qiu X, Dong Z*, et al.* Systematic Review on the Efficacy and Safety of Adjuvant Therapy of Compound Kushen Injection for Pleural Effusion in Elderly Patients with Malignant Cancer. China Pharmacy, 2018, 29(17): 2421-2425.
[63] Yang X, Wei X, Jiang L. Network Meta-analysis of 5 Kinds of TCM Injections in the Treatment of Malignant Pleural Effusion. China Pharmacy, 2017, 28(33): 4686-4690.
[64] Yang M, Ren J, Wen S*, et al.* Compound Kushen injection combined with cisplatin for treatment of malignant pleural effusion:A Meta -analysis. J Modern Oncol, 2016, 24(21): 3393-3398.
[65] Xu C, Hu Z, Xie M*, et al.* Meta-Analysis of Intrapleural Injection of Compound Kushen Injection Combined with Chemotherapy in Treating Malignant Pleural Effusion. China Pharmaceuticals, 2015, 24(18): 31-32,33.
[66] Biaoxue R, Shuxia M, Wenlong G*, et al.* Thoracic perfusion of matrine as an adjuvant treatment improves the control of the malignant pleural effusions. World J Surg Oncol, 2015, 13: 329.
[67] Tang J, Fang S, Fu S*, et al.* Meta-analysis of Kang'ai Injection combined with Cisplatin in the Treatment of Malignant Pleural Effusion. Chin J Ethnomed Ethnopharm, 2014, 23(19): 19-21+25.
[68] Tian X, Wang W, Jia L. Meta-Analysis of TCM injection In the treatment of malignant pleural effusion. Chin Med MDE China, 2010, 8(18): 175-178.

4. List of newly added trials since the publication of latest SRs/meta-analysis in 2018

Twenty-three trials have been published since the publication of latest SRs/meta-analysis in 2018 [62]

[1] Liu D, Li D. Curative effect and nursing of thoracic cavity drainage and compound Kushen injection combined with cisplatin in the treatment of lung cancer patients companying with malignant pleural effusion. J Clin Med Pract, 2015, 19(8): 21-24.

[2] Song X, Jia Y. Compound Kushen injection combined with cisplatin pleural perfusion in the treatment of 59 cases of malignant pleural effusion. Forum TCM, 2015, 30(3): 30-31.

[3] Zhang S, Chen X, Chen Y*, et al.* Effiacy and safety of compound Kushen injection plus nedaplatin for malignant pleural effusion in patients with lung cancer. Eval Anal Drug-Use Hosp China, 2015, 15(5): 601-603.

[4] Zhong B, Wei W, Li Y*, et al.* A clinical observation of intrapleural perfusion of OK-432 combined with compound Kushen injection in treatment of malignant pleural effusion. Modern Med J China, 2015, 17(3): 8-10.

[5] Liu X, Xu J. Therapeutic effects and action mechanism of lobaplatin combined with Kushen injection on malignant pleural effusion in patients with advanced lung cancer. Hebei Med J, 2016, 38(10): 1461-1464.

[6] Qin D, Fan K. Analysis of the therapeutic effect of compound sophora flavescens injection and cisplatin thoracic perfusion therapy in patients with malignant pleural effusion. Med Health, 2016(6): 257-257.

[7] Wang H. Sophora radix astragali injection combined cisplatin intrathoracic injection clinical observation on treatment of lung cancer hydrothorax. Clin Res, 2016, 24(6): 2-3.

[8] Wang S, Zhou W. Clinical observation of Compound Kushen Injection in the treatment of malignant pleural effusion. China Pract Med, 2016, 11(16): 148-149.

[9] Yan G, Jiang H, Wang P*, et al.* The application of compound sophora injection combined with cisplatin in the treatment of malignant pleural effusion by pleural perfusion. Jilin Med J, 2016, 37(3): 574-576.

[10] Huang H, He Z, Li X*, et al.* Analysis of curative effect by compound Kushen injection combined with cisplatin through intrapleural infusion in the treatment of malignant pleural effusion. Chin J Mod Drug Appl, 2017, 11(7): 29-31.

[11] Li R, Yu J, Zhang S*, et al.* Eficacy of compound Kushen Injection combined with nedaplatin for malignant pleurai efusion. Liaoning J TCM, 2017, 44(4): 789-790.

[12] Liu L, Zhong S, Li G. Effects and safety of compound Kushen injection combined with cisplatin on malignant pleural effusion. J Modern Oncol, 2017, 25(2): 230-233.

[13] Shi W. Clinical efficacy and safety of compound Kushen injection combined with cisplatin in the treatment of malignant pleural effusion caused by lung cancer. Chi J Convalescent Med, 2017, 26(8): 857-859.

[14] Tang X, Jiang M, Li J*, et al.* Cinical objective on sixty cases of compound Kushen Injection combined with cisplatin in treatment of lung cancer pleural effusion. Liaoning J TCM, 2018, 45(8): 1668-1670.

[15] Cai H, Wang Q. Effect of Compound Kushen Injection on elderly patients with lung cancer

complicated with malignant pleural effusion. Contemp Med Symp, 2019, 17(11): 161-162.
[16] Wang Y, Fan S, Luo Q, *et al.* Short term efficacy of pleural cavity drainage and infusion of compound Sophora flavescens injection combined with cisplatin in the treatment of malignant pleural effusion in lung cancer. World Latest Med Inform, 2019, 19(02): 116+121.
[17] Wu C, Wu J, Wu X, *et al.* Clinical study of compound radix sophorae flavescentis injection combined with cisplatin in treatment of malignant pleural effusion. Liaoning J TCM, 2019, 46(1): 85-87.
[18] Jiang T, Li J. Compound Sophora flavescens injection and cisplatin in the treatment of malignant pleural effusion: A clinical study. Medicine & Health, 2020, 11(11): 1-2.
[19] Peng H. Clinical effect of compound sophora flavescens injection in palliative treatment of advanced lung cancer patients with malignant pleural effusion. Health for Everyone, 2020, 27(14): 595-595.
[20] Huang L. Clinical effect of compound sophora flavescens injection in palliative treatment of advanced lung cancer patients with malignant pleural effusion. Health Manag, 2021(22): 71-72.
[21] Feng F, Shi X. Clinical study of Compound Kushen Injection combined with cisplatin in treatment of non-small cell lung cancer malignant pleural effusion. Drugs & Clinic, 2023, 38(7): 1717-1721.
[22] Lin W, Yuan S, Li F, *et al.* Effect of intracavitary perfusion of compound Sophora Flavescens injection on the immune function and tumor markers in patients with malignant pleural effusion. Prog Modern Biomed, 2023, 23(19): 3758-3762.
[23] Wang R, Chen M, Wang Y, *et al.* Efficacy and safety of compound Kushen injection in the treatment of malignant pleural effusions caused by breast cancer. China Licensed Pharmacist 2023, 20(10): 67-71.

5.List of 64 ineligible studies

After evaluating full-texts, we excluded 64 ineligible studies in terms of objects, intervention measures, controls and et. al

1.Subjects confounding with ascites or pericardial effusion (7 studies)

[1] 刘玉艳，黄秀莲．复方苦参联合卡介菌多糖治疗恶性胸腹水疗效观察．山东医药, 2007(16): 10.
[2] 冉之蓉．岩舒联合斯奇康治疗恶性胸腹水疗效观察．现代医药卫生, 2007, 23(2).
[3] 代新幼，曾学富，黄舒, et al. 复方苦参注射液联合顺铂热灌注治疗恶性胸腹腔积液近期疗效分析．肿瘤研究与临床, 2012, 24(7): 486-487.
[4] 丁旭东．复方苦参联合卡介菌多糖治疗恶性胸腹水疗效观察．中外医疗, 2012, 31(3).
[5] 宋春燕，王翠英．康艾注射液联合顺铂热灌注治疗恶性胸腹腔积液 80 例．西部中医药, 2013, 26(06): 87-88.
[6] 邓建林．肿瘤恶性积液顺铂热灌注治疗疗效分析．系统医学, 2017, 2(23).
[7] 姚娟，李彦磊，姜玉华, et al. 复方苦参注射液联合顺铂治疗恶性胸腹水疗效观察．辽宁中医杂志, 2018, 45(03): 563-564.

2.Non thoracic perfusion (18 studies)

[1]．奉拉拉，李世杰，曾红萍，蒋晓玲．加用康艾注射液治疗恶性胸腔积液的临床观察．广西中医药 2015;38:23-25. doi.
[2]．郭兰萍，陈莉延，易震南，张艳．复方苦参注射液与 A 群链球菌联合治疗恶性胸腔积液

24 例. 肿瘤研究与临床 2007;19:622-623. doi: 10.3760/cma.j.issn.1006-9801.2007.09.019.
[3]. 贺娟娟, 李延玲, 郭瑞霞, 温凤霞. 复方苦参注射液联合 TP 方案治疗晚期肺癌恶性胸腔积液疗效及对 CEA、AFP 和 NSE 水平的影响. 现代中西医结合杂志 2020;29:3670-3673,3678. doi: 10.3969/j.issn.1008-8849.2020.33.006.
[4]. 江雨顺, 杨杰. 周剂量顺铂胸腔灌注联合复方苦参注射液治疗非小细胞肺癌并恶性胸腔积液的临床研究. 现代医药卫生 2013;29:1619-1620. doi: 10.3969/j.issn.1009-5519.2013.11.008.
[5]. 刘春秋, 李国欢, 刘卫东. 复方苦参注射液在晚期肺癌恶性胸腔积液患者姑息治疗中的临床研究. 辽宁中医杂志 2016;43:74-75. doi: 10.13192/j.issn.1000-1719.2016.01.032.
[6]. 刘思杰, 刘宏, 边建伟. 复方苦参注射液联合白介素-Ⅱ、顺铂治疗恶性胸腔积液的近期疗效观察. 中国现代医药杂志 2011;13:42-44. doi: 10.3969/j.issn.1672-9463.2011.07.017.
[7]. 刘新平, 刘睿, 许国磊, 黄静娟, 李树斌. 消水方联合顺铂胸腔灌注治疗恶性胸腔积液的疗效观察. 现代药物与临床 2014899-902. doi: 10.7501/j.issn.1674-5515.2014.08.017.
[8]. 欧立文, 曹爱国, 卢平, 李之茂. 周剂量吉西他滨联合苦参素胸腔灌注治疗非小细胞肺癌患者胸腔积液的疗效观察. 中国医药导刊 2011;13:1354-1355. doi: 10.3969/j.issn.1009-0959.2011.08.039.
[9]. 欧涛, 覃世运, 王文尖, 黄庆茂, 毛海宏, 龚海英. 复方苦参注射液联合博莱霉素治疗恶性胸腔积液临床观察. 中国医院用药评价与分析 2010;10:50-52. doi.
[10]. 司廷林, 侯慧珍. 复方苦参注射液配合胸腔灌注化疗治疗肺癌胸腔积液的临床研究. 中国医药导报 2008;5:84-85,91. doi: 10.3969/j.issn.1673-7210.2008.25.056.
[11]. 宋春燕, 许士珍, 王翠英. 康艾注射液联合热化疗治疗恶性胸腔积液 45 例. 陕西中医 2013;34:1586-1587. doi.
[12]. 宋军俊, 戈伟. 恩度联合复方苦参注射液治疗晚期非小细胞肺癌恶性胸腔积液的效果. 中国医药导报 2018;15:143-146. doi.
[13]. 吴幸谕, 李劲, 黄光位, 陈文宇. 复方苦参碱联合甘露聚糖肽胸腔灌注治疗肺癌伴恶性胸腔积液的疗效观察. 慢性病学杂志 2018. doi: 10.16440/j.cnki.1674-8166.2018.04.020.
[14]. 谢新梅, 张洪亮, 肖春霞. 康艾注射液联合顺铂治疗恶性胸腔积液 32 例. 陕西中医 2011;32:1329-1331. doi.
[15]. 许士珍, 宋春燕, 王翠英. 康艾注射液联合热化疗治疗恶性胸腔积液 80 例. 临床荟萃 2013;28:1278-1279. doi.
[16]. 杨光华, 陈连生. 复方苦参注射液联合胸腔内注射治疗恶性肿瘤并发胸腔积液临床观察. 中国中医急症 2010;19:29,46. doi: 10.3969/j.issn.1004-745X.2010.01.018.
[17]. 张喜峰. 复方苦参注射液联合顺铂胸腔灌注治疗恶性胸腔积液 49 例. 北方药学 2017;14:39. doi: 10.3969/j.issn.1672-8351.2017.10.030.
[18]. 张玉芳, 张素芳, 常万里. 复方苦参注射液联合顺铂治疗癌性胸水. 中外健康文摘·医药月刊 2007;4:14-16. doi.

3. Radix Sophorae flavescentis preparitions with hyperthermia (8 studies)

[1] 蒋新建, 明静, 李建蓉. 高温注射用水与苦参注射液序贯胸腔灌注治疗肺癌恶性胸水的观察. 现代肿瘤医学, 2009, 17(11): 2148-2149.
[2] 李际强. 热物理疗法配合中药腔内灌注治疗恶性胸腔积液的临床研究, 2007.
[3] 林振怀, 孙砚诚, 冀学红, et al. 顺铂加苦参胸腔灌注联合微波热疗治疗恶性胸水的疗效观察. 中国医药指南, 2011, 9(6): 64-65.
[4] 林振怀, 孙砚诚, 王永仓. 顺铂加苦参胸腔灌注化疗联合微波热疗在不同阶段治疗恶性胸水的疗效观察. 中国医药指南, 2011, 9(8): 76-78.

[5] 刘福蓉, 廖大忠, 鲁光红. 羟喜树碱联合复方苦参注射液胸腔灌注配合深部热疗治疗恶性胸水. 药物流行病学杂志, 2012, 21(6): 266-267.
[6] 鲁光洪, 刘福蓉, 余朝锈. 化疗药胸腔灌注配合深部热疗治疗恶性胸水的护理观察. 泸州医学院学报, 2013(5): 519-522.
[7] 梅林, 郭日昌, 罗俊生, et al. 持续胸腔热灌注化疗恶性胸腔积液近期疗效的分析. 国际数字医学会数字中医药分会成立大会暨首届数字中医药学术交流会, 2016: 2.
[8] 武智刚, 吴铁鹰, 张峻青. 岩舒注射液联合胸腔热灌注治疗恶性胸腔积液 36 例疗效观察. 山东医药, 2010, 50(13): 105-106.

4. Kushen plus systemic chemotherapy (4 studies)

[1] 李多, 邓述恺, 刘丹. 岩舒注射液联合顺铂治疗肺癌恶性胸腔积液疗效观察. 时珍国医国药, 2009, 20(5): 1253-1254.
[2] 胡代菊, 梅晓冬. 复方苦参注射液、IL-2 及 α-干扰素治疗肺癌伴恶性胸腔积液疗效. 临床肺科杂志, 2012, 17(10): 1844-1845.
[3] 温宏升, 杨芳, 李旭, et al. 卡铂、复方苦参注射液序贯胸腔灌注联合静脉化疗治疗恶性胸腔积液的疗效观察. 中国药房, 2012, 23(40): 3790-3792.
[4] 王鹏飞, 刘凤娟, 徐涛, et al. 内科胸腔镜术后注入复方苦参注射液治疗肺癌胸腔积液. 齐齐哈尔医学院学报, 2015(28): 4277-4277.

5.Kushen with oral TCM (3 studies)

[1]. 郭燕蓉, 茆建国. 中药联合胸腔内化疗治疗肺癌胸腔积液 30 例. 中国中医药现代远程教育 2010;8:43-44. doi: 10.3969/j.issn.1672-2779.2010.02.036.
[2]. 秦庆寅, 陆方阳, 黄贵佳, 张力, 康智岷, 何咏梅. 复方苦参注射液胸腔灌注联合扶正消水方内服治疗恶性胸腔积液 44 例. 中国实验方剂学杂志 2013;19:306-309. doi: 10.11653/syfj2013150306.
[3]. 张金玲, 刘宇, 张会平, 姚蓓, 和婧, 郭小培, *et al.* 鲫鱼利水方联合复方苦参注射液治疗肺癌胸水的疗效观察. 中医药临床杂志 2024;36:1976-1980. doi: 10.16448/j.cjtcm.2024.1030.

6. Both groups with inconsistent agent (11 studies)

[1] 邓健麟, 吴奕奕. 康艾注射液联合洛铂治疗恶性胸腔积液的药学作用探讨. 中国现代药物应用, 2023, 17(04): 27-30.
[2] 冯活林, 倪雪莉, 黄锡英, et al. 复方苦参注射液联合顺铂胸腔灌注治疗老年恶性胸腔积液的临床研究. 中医临床研究, 2019, 11(02): 141-142.
[3] 郭志强. 复方苦参注射液及白介素-2 治疗恶性胸水的疗效观察. 河南医学研究, 2013, 22(4): 499-501.
[4] 李玮, 任华, 朱金如, et al. 老年恶性胸腔积液药物联合治疗临床观察. 中国医院用药评价与分析, 2011, 11(7): 636-638.
[5] 刘颖华, 张晓伟, 刘丽娜. 复方苦参注射液联合斑蝥酸钠维生素 B6 注射液治疗癌性胸腔积液临床观察. 中国美容医学, 2012, 21(18): 369-370.
[6] 汤艳丽, 邵丽黎. 复方苦参注射液合白介素-2 治疗恶性胸水 35 例. 山西中医, 2011, 27(4): 31-32.
[7] 王志强. 康艾注射液治疗恶性胸腔积液 51 例. 中国中医药现代远程教育, 2014(21): 41-42.
[8] 王子熹, 夏宝芳. 胸腔内注射复方苦参注射液与白细胞介素-2 治疗恶性胸水的疗效观察. 国际中医中药杂志, 2013, 35(10): 927-929.
[9] 翁冠裕，刘宜禄. 胸腔留置导管引流并注入药物治疗恶性胸腔积液的临床观察. 中国健康月刊：学术版, 2010(第 9 期): 7.

[10] 夏国安. 博莱霉素联合复方苦参注射液治疗老年肺癌恶性胸腔积液疗效观察. 安徽医药, 2013, 17(1): 131-132.
[11] 赵培, 刘璇. 康艾注射液联合洛铂治疗恶性胸腔积液. 中西医结合心血管病电子杂志, 2020, 8(7): 38,51.

7.No data available (7 studies).

[1] 李丽. 顺铂联合复方苦参注射液治疗恶性胸腔积液 42 例. 第十五届全国临床肿瘤学大会暨 2012 年 CSCO 学术年会论文集, 2012: 332-332.
[2] 李丽, 田菲. 顺铂联合中医药治疗恶性胸腔积液疗效观察. 第十四届全国临床肿瘤学大会暨 2011 年 CSCO 学术年会论文集, 2011: 252-252.
[3] 林成辉, 王云, 肖汝平. 苦参碱对恶性胸水患者癌胚抗原和免疫功能的影响. 海南医学, 2011, 22(10): 38-40.
[4] 冉凤鸣, 臧爱华. 复方苦参联合顺铂治疗恶性胸腔积液的临床观察. 湖北省抗癌协会青年委员会成立大会暨第一届青年学术论坛, 2009: 2.
[5] 任金贤, 庄海峰, 周郁鸿, et al. 复方苦参注射液(岩舒)联合顺铂胸腔内注射治疗恶性血液病并发胸腔积液临床观察. 2011 年浙江省中医药学会血液病分会学术年会暨国家中医临床研究基地血液病研究 2011 高峰论坛暨国家级继续教育中西医结合血液病新进展学习班, 2011: 1.
[6] 张振安, 安静, 张风林, et al. 复方苦参腔内灌注治疗对恶性胸腔积液患者胸水中 VEGF、MMP 含量的影响. 海南医学, 2015(2): 166-168.
[7] 周惠明. 苦参治疗恶性胸腔积液 53 例观察. 中外健康文摘, 2010, 7(11): 346-347.

8.The duplicates (6 studies)

[1] 林振怀, 孙砚诚, 王永仓, 复方苦参注射液联合白细胞介素 2 治疗老年肺癌恶性胸腔积液的临床观察, 中国医药指南 9(7) (2011) 63-65.
[2] 刘煜, 胸腔闭式引流术后复方苦参注射液胸腔注射治疗肺癌胸腔积液, 中国保健营养 27(34) (2017) 131-132.
[3] 马海龙, 马琦, 洛铂联合复方苦参注射液治疗晚期肺癌恶性胸腔积液疗效及机制研究, 现代肿瘤医学 25(24) (2017) 4000-4003.
[4] 唐兰珊, 康艾注射液联合顺铂治疗恶性胸腔积液临床观察, 中外健康文摘 8(23) (2011) 199-200.
[5] 袁亚军, 张凤林, 复方苦参注射液治疗恶性胸腔积液临床观察, 山东医药 48(37) (2008) 101-102.
[[1] Zhang X. Clinical effect of Kangai injection combined with cisplatin on malignant pleural effusion. Liaoning J TCM, 2006, 33(11): 1427-1428.
[2] Huang X. Clinical observation on treating pleural effusion of lung cancer with Yan-shu inject plus cisplatin J Pract Chin Modern Med, 2007, 20(12): 1106-1106,1108.
[3] Pan J, Chu D, Hu Z*, et al.* Clinical observation intracavity Kushen injection combined with cisplatin for the treatment of malignant pleural efusion. Chin J Clin Pharm, 2007, 16(3): 139-141.
[4] Yuan Y. Thoracentesis and drainage combined with Yanshu injection for malignant pleural effusion. China Pharm, 2007, 18(24): 1891-1892.
[5] Hu Q, Wang H, Pan J. Effect of Yanshu injection on malignant pleural effusion of advanced lung cancer. J Chengdu Univ TCM, 2008, 31(1): 15-17.
[6] Xu M, Xiong B. Intrapleural injection of Kangai zhusheye with cisplatin in malignant pleural efusion. Pract J Cardiac Cereb Pneum Vasc Dis, 2008, 16(2): 114-117.

[7] Zhang S, Jia Y, Yao L*, et al.* Clinical study of compound Kushen injection combined with cisplatin on malignant pleural effusion. World Health Digest, 2008, 5(8): 1218-1219.
[8] Ding L, Cui C, Xin B. The clinical observation of compound Kushen injection combined with cisplatin in the treatment of malignant pleural effusion by intrathoracic injection. J Modern Oncol, 2009, 17(7): 1274-1275.
[9] Li Y, Chen C, Li Q. Compound Kushen injection combined with cisplatin pleural injection for lung cancer pleural effusion. China Naturopathy, 2009, 17(4): 42-42.
[10] He L, Chen Z, Wen S*, et al.* Compound Kushen Injection as a local therapy for patients with advanced lung cancer associated with malignant pleural effusion. Eval Anal Drug-Use Hosp China, 2010, 10(11): 1025-1027.
[11] Wang Y. Clinical study of compound Kushen injection combined with cisplatin in the treatment of malignant pleural effusion. Chin J Mod Drug Appl, 2010, 4(5): 148-149.
[12] Zhou Y, Xu X, Zhou P. Clinical observation on compound matrine injection plus interleukin-2 in the treatment of malignant pleural effusion induced by lung cancer in elder. Chin J Clin Pharm Ther, 2010, 15(12): 1402-1405.
[13] Chen Y, Li Q, Xiang S*, et al.* Compound Kushen Injection combined with cisplatin for malignant pleural effusion： A clinical study. Eval Anal Drug-Use Hosp China, 2011, 11(4): 366-367.
[14] He J. Clinical study of Kangai injection combined with cisplatin in treatment of malignant pleural effusion. Med Inform, 2011, 24(8): 3756-3757.
[15] Liang Z, Li X, Liu Z*, et al.* Efficacy of compound matrine injection for malignant pleural effusion. Eval Anal Drug-Use Hosp China, 2011, 11(6): 547-548.
[16] Liu Y, Wan L. Clinical study of compound Kushen injection combined with bleomycin in the treatment of malignant pleural effusion. Chin Community Doct, 2011, 13(9): 178-179.
[17] Ran F, Zang A. Efficacy of Compound Kushen Injection combined with cisplatin for malignant pleural effusion. Eval Anal Drug-Use Hosp China, 2011, 11(8): 739-740.
[18] Wei M, Sun Q. Efficacy of compound Kushen injection combined with cisplatin in the treatment of malignant pleural effusion. JCM, 2011, 9(2): 26-27.
[19] Han Z, Tian M, Chen X*, et al.* Effect of closed drainage combined with compound Kushen injection and cisplatin on malignant pleural effusion of lung cancer. Zhejiang J ITCWM, 2012, 22(7): 524-526.
[20] Huang Z, Zhang H, Zhang K*, et al.* The clinical research of compound Kushen injection and corynebacterium parvum as a local therapy for patients with advanced lung cancer associated with malignant pleural effusion. J Clin Pulm Med, 2012, 17(3): 497-498.
[21] Qu D, Liang X, Zhou B. Clinical observation of Kangai injection combined with cisplatin in the treatment of malignant pleural effusion. Modern J ITCWM, 2012, 21(21): 2311-2312.
[22] Sun Y. Bleomycin combined with compound Kushen injection in the treatment of 25 cases of malignant pleural effusion. Zhejiang J TCM, 2012, 47(2): 143-143.
[23] Yang G. The recent efficacy of compound Kushen injection combined with cisplafin chest perfusion for malignant pleurai effussion. China Med, 2012, 7(9): 1079-1080.
[24] Zhuang H, Ren J, Zhou Y*, et al.* Matrine injection combined with intrapleural cisplatin in treatment of 24 patients with hematologic malignancies complicated by pleural effusion. Chin J New Drugs, 2012, 21(9): 1013-1015.
[25] Chen L. Effect of compound matrine injection on malignant pleural effusion. Chin J New Drugs, 2013, 22(17): 2069-2070+2074.
[26] Guo L, Luo D, Yang S. Observation on efficacy of compound Kushen injection combined with

cisplatin in the treatment of malignant pleural fluid. China Med Herald, 2013, 10(25): 79-81.
[27] Han S. Effect of compound Kushen injection combined with cisplatin pleural perfusion on malignant pleural effusion. J Clin Res, 2013, 30(6): 1191-1193.
[28] Xing H. Clinical observation of compound Kushen injection in the treatment of malignant pleural effusion. Chin J Mod Drug Appl, 2013, 7(17): 84-85.
[29] Zhang X, Geng S, Wang Y. Clinical study of compound Kushen injection combined with mitomycin for malignant pleural efusion. Chin J Clin Oncol Rehabil, 2013, 20(7): 780-782.
[30] Zheng S, Jia X. Clinical observation of pleural perfusion in the treatment of malignant pleural effusion. Hebei Med J, 2013, 35(3): 416-417.
[31] Zhu M, Jiang H, Zhu Z*, et al.* Clinical study of perfusion with compound Kushen injection plus cisplatin for malignant pleural fluid. Tianjin Pharm, 2013, 25(6): 30-32.
[32] Chen Y, Luo Y, Qin Z. Effect of compound Kushen injection combined with cisplatin on malignant pleural effusion in aged patients. Res Integr Tradit Chin West Med, 2014, 6(4): 196-197+199.
[33] Jiang J. Clinical effect of compound Kushen Injection combined with cisplatin in treatment of malignant pleural effusion. China Modern Med, 2014, 21(26): 113-115.
[34] Li S. Clinical observation of compound Kushen injection combined with nedaplatin in the treatment of malignant pleural effusion. Res Integr Tradit Chin West Med, 2014, 6(2): 88-89+91.
[35] Wu Z, Zhang R, Liu Q*, et al.* Therapeutic effect of hydroxycamptothecin combined with Kushen Injection on malignant pleural effusion in elderly patients with lung cancer Hebei Med J, 2014, 36(24): 3705-3707.
[36] Liu D, Li D. Curative effect and nursing of thoracic cavity drainage and compound Kushen injection combined with cisplatin in the treatment of lung cancer patients companying with malignant pleural effusion. J Clin Med Pract, 2015, 19(8): 21-24.
[37] Liu L, Zhong S, Li G. Effects and safety of compound Kushen injection combined with cisplatin on malignant pleural effusion. J Modern Oncol, 2017, 25(2): 230-233.
[38] Shi W. Clinical efficacy and safety of compound Kushen injection combined with cisplatin in the treatment of malignant pleural effusion caused by lung cancer. Chi J Convalescent Med, 2017, 26(8): 857-859.
[39] Tang X, Jiang M, Li J*, et al.* Cinical objective on sixty cases of compound Kushen Injection combined with cisplatin in treatment of lung cancer pleural effusion. Liaoning J TCM, 2018, 45(8): 1668-1670.
[40] Wang Y, Fan S, Luo Q*, et al.* Short term efficacy of pleural cavity drainage and infusion of compound Sophora flavescens injection combined with cisplatin in the treatment of malignant pleural effusion in lung cancer. World Latest Med Inform, 2019, 19(02): 116+121.
[41] Wu C, Wu J, Wu X*, et al.* Clinical study of compound radix sophorae flavescentis injection combined with cisplatin in treatment of malignant pleural effusion. Liaoning J TCM, 2019, 46(1): 85-87.
[42] Peng H. Clinical effect of compound sophora flavescens injection in palliative treatment of advanced lung cancer patients with malignant pleural effusion. Health for Everyone, 2020, 27(14): 595-595.
[43] 周永清, 陈旭, 丁海斌. 复方苦参注射液 IL-2 及干扰素辅助治疗肺癌伴恶性胸腔积液疗效和不良反应. 西部医学, 2016, 28(9): 1261-1263,1270.
[44] 李玮, 任华, 朱金如*, et al.* 老年恶性胸腔积液药物联合治疗临床观察. 中国医院用药评价与分析, 2011, 11(7): 636-638.

[45] 夏国安. 博莱霉素联合复方苦参注射液治疗老年肺癌恶性胸腔积液疗效观察. 安徽医药, 2013, 17(1): 131-132.
[46] 林振怀, 孙砚诚, 王永仓. 复方苦参注射液联合白细胞介素 2 治疗老年肺癌恶性胸腔积液的临床观察. 中国医药指南, 2011, 9(7): 63-65.
[47] 周良发, 胡作为, 徐涛. 复方苦参注射液联合顺铂治疗恶性胸腔积液的近期疗效观察. 贵阳中医学院学报, 2012, 34(5): 145-147.
[48] 刘思杰, 刘宏, 边建伟. 复方苦参注射液联合白介素-Ⅱ、顺铂治疗恶性胸腔积液的近期疗效观察. 中国现代医药杂志, 2011, 13(7): 42-44.
[49] 江雨顺, 杨杰. 周剂量顺铂胸腔灌注联合复方苦参注射液治疗非小细胞肺癌并恶性胸腔积液的临床研究. 现代医药卫生, 2013, 29(11): 1619-1620.
[50] 郭志强. 复方苦参注射液及白介素-2 治疗恶性胸水的疗效观察. 河南医学研究, 2013, 22(4): 499-501.
[51] 胡代菊, 梅晓冬. 复方苦参注射液、IL-2 及α-干扰素治疗肺癌伴恶性胸腔积液疗效. 临床肺科杂志, 2012, 17(10): 1844-1845.
[52] 裴锋. 复方苦参注射液联合顺铂治疗恶性胸腔积液的疗效观察. 时珍国医国药, 2012, 23(6): 1580.
[53] 汤伟忠. 复方苦参注射液加顺铂治疗晚期恶性胸腔积液的观察. 中国医院用药评价与分析, 2009, 9(7): 538-539.
[54] 冯青, 蔡正凤. 康艾注射液联合顺铂治疗恶性胸腔积液. 中国民族民间医药, 2010, 19(09): 25+32.
[55] 刘明伟. 康艾注射液治疗肺癌胸水的临床研究. 河南职工医学院学报, 2011, 23(06): 677-678.
[56] 宋春燕, 许士珍, 王翠英. 康艾注射液联合热化疗治疗恶性胸腔积液 45 例. 陕西中医, 2013, 34(12): 1586-1587.
[57] 许士珍, 宋春燕, 王翠英. 康艾注射液联合热化疗治疗恶性胸腔积液 80 例. 临床荟萃, 2013, 28(11): 1278-1279.
[58] 谢新梅, 张洪亮, 肖春霞. 康艾注射液联合顺铂治疗恶性胸腔积液 32 例. 陕西中医, 2011, 32(10): 1329-1331.
[59] Xu YF, Chen YR, Bu FL*, et al.* Chinese herbal injections versus intrapleural cisplatin for lung cancer patients with malignant pleural effusion: A Bayesian network meta-analysis of randomized controlled trials. Front Oncol, 2022, 12: 942941.
[60] Li Z. Network meta-analysis and network pharmacology study of Chinese medicine injection combined with cisplatin in the treatment of malignant pleural effusion of lung cancer. Ruikang Clinical Medical College, 2022, Master.
[61] Li B, Yuan Q, Wang Y*, et al.* Network meta-analysis of 8 traditional Chinese medicine injections combined with cisplatin for malignant pleural effusion. CHin Hosp Pharm J, 2019, 39(10): 1052-1057.
[62] Wu H, Qiu X, Dong Z*, et al.* Systematic Review on the Efficacy and Safety of Adjuvant Therapy of Compound Kushen Injection for Pleural Effusion in Elderly Patients with Malignant Cancer. China Pharmacy, 2018, 29(17): 2421-2425.
[63] Yang X, Wei X, Jiang L. Network Meta-analysis of 5 Kinds of TCM Injections in the Treatment of Malignant Pleural Effusion. China Pharmacy, 2017, 28(33): 4686-4690.
[64] Yang M, Ren J, Wen S*, et al.* Compound Kushen injection combined with cisplatin for treatment of malignant pleural effusion:A Meta -analysis. J Modern Oncol, 2016, 24(21): 3393-3398.
[65] Xu C, Hu Z, Xie M*, et al.* Meta-Analysis of Intrapleural Injection of Compound Kushen Injection Combined with Chemotherapy in Treating Malignant Pleural Effusion. China Pharmaceuticals, 2015,

24(18): 31-32,33.
[66] Biaoxue R, Shuxia M, Wenlong G*, et al.* Thoracic perfusion of matrine as an adjuvant treatment improves the control of the malignant pleural effusions. World J Surg Oncol, 2015, 13: 329.
[67] Tang J, Fang S, Fu S*, et al.* Meta-analysis of Kang'ai Injection combined with Cisplatin in the Treatment of Malignant Pleural Effusion. Chin J Ethnomed Ethnopharm, 2014, 23(19): 19-21+25.
[68] Tian X, Wang W, Jia L. Meta-Analysis of TCM injection In the treatment of malignant pleural effusion. Chin Med MDE China, 2010, 8(18): 175-178.
