# Supplementary material for: Intrapleural administration with traditional Chinese medicine injections (Sophorae flavescentis preparations) in controlling malignant pleural effusion: a clustered systematic review and meta-analysis
Source: Front Pharmacol. 2025 Apr 24;16:1519794. doi: 10.3389/fphar.2025.1519794 (PMC12058796; doi:10.3389/fphar.2025.1519794)
Supplement: Supplementary file 2 [file DataSheet4.pdf]

Supplementary materials.5 Meta-analysis results (Clinical responses, QOL and Adverse events (Figures.S6 to S24)

Study or Subgroup	Kushen Events	Kushen Total	Sclerosants Events	Sclerosants Total	Weight	Odds Ratio M-H, Fixed, 95% CI
**Compound Kushen injection vs Cisplatin(Nine trials)**						
Hu, Q. 2008	7	20	8	20	9.7%	0.81 [0.22, 2.91]
Chen, X. 2010	15	28	17	30	14.2%	0.88 [0.31, 2.49]
Yan, G. 2016	8	20	12	30	10.7%	1.00 [0.32, 3.17]
Yuan, Y. 2007	6	26	6	26	8.6%	1.00 [0.28, 3.63]
Chen, L. 2013	10	40	9	40	12.6%	1.15 [0.41, 3.22]
Xing, H. 2013	12	45	10	42	14.2%	1.16 [0.44, 3.07]
Liang, Z. 2011	19	56	16	54	20.1%	1.22 [0.55, 2.73]
Wang, S. 2016	8	30	6	30	8.2%	1.45 [0.44, 4.86]
Wang, R. 2023	2	16	1	15	1.7%	2.00 [0.16, 24.66]
**Subtotal (95% CI)**		**281**		**287**	**100.0%**	**1.10 [0.76, 1.60]**
Total events	87		85			
Heterogeneity: Chi² = 0.95, df = 8 (P = 1.00); I² = 0%						
Test for overall effect: Z = 0.53 (P = 0.60)						
**Compound Kushen injection vs Interleukin-2(One trial)**						
Huang,X.2013	45	65	31	63	100.0%	2.32 [1.13, 4.78]
**Subtotal (95% CI)**		**65**		**63**	**100.0%**	**2.32 [1.13, 4.78]**
Total events	45		31			
Heterogeneity: Not applicable						
Test for overall effect: Z = 2.29 (P = 0.02)						
**Compound Kushen injection vs Mitomycin(One trial)**						
Zhang, X. 2011	16	50	15	50	100.0%	1.10 [0.47, 2.56]
**Subtotal (95% CI)**		**50**		**50**	**100.0%**	**1.10 [0.47, 2.56]**
Total events	16		15			
Heterogeneity: Not applicable						
Test for overall effect: Z = 0.22 (P = 0.83)						

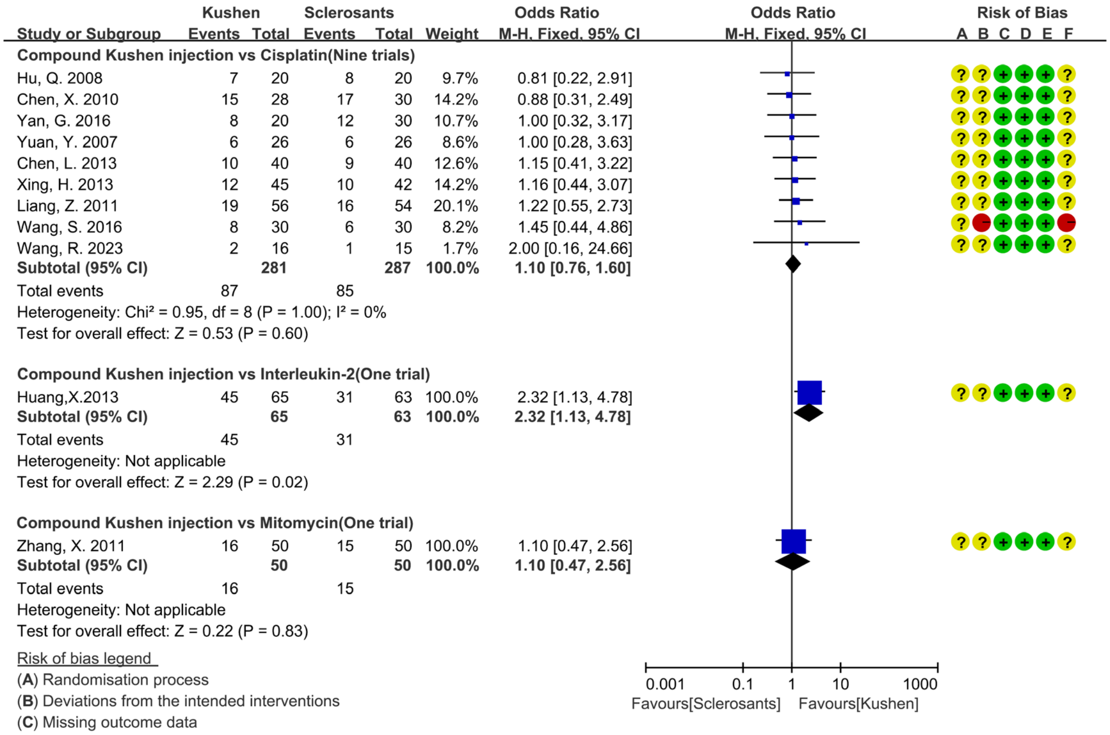

Risk of bias legend
(**A**) Randomisation process
(**B**) Deviations from the intended interventions
(**C**) Missing outcome data
(**D**) Measurement of the outcome
(**E**) Selection of the reported result
(**F**) Overall bias

Figure.S6 The complete response of compound Kushen injection alone

Study or Subgroup	Kushen Events	Kushen Total	Sclerosants Events	Sclerosants Total	Weight	Odds Ratio M-H, Fixed, 95% CI
**Compound Kushen injection vs Cisplatin(Nine trials)**						
Xing, H. 2013	9	45	19	42	22.8%	0.30 [0.12, 0.78]
Yuan, Y. 2007	8	26	12	26	12.0%	0.52 [0.17, 1.61]
Liang, Z. 2011	14	56	17	54	18.8%	0.73 [0.32, 1.67]
Chen, X. 2010	5	28	5	30	5.7%	1.09 [0.28, 4.25]
Chen, L. 2013	15	40	14	40	12.7%	1.11 [0.45, 2.77]
Wang, R. 2023	8	16	7	15	5.2%	1.14 [0.28, 4.68]
Wang, S. 2016	14	30	13	30	10.0%	1.14 [0.41, 3.17]
Yan, G. 2016	8	20	11	30	7.6%	1.15 [0.36, 3.68]
Hu, Q. 2008	6	20	5	20	5.1%	1.29 [0.32, 5.17]
**Subtotal (95% CI)**		**281**		**287**	**100.0%**	**0.80 [0.56, 1.14]**
Total events	87		103			
Heterogeneity: Chi² = 6.88, df = 8 (P = 0.55); I² = 0%						
Test for overall effect: Z = 1.24 (P = 0.21)						
**Compound Kushen injection vs Interleukin-2(One trial)**						
Huang,X.2013	9	65	25	63	100.0%	0.24 [0.10, 0.58]
**Subtotal (95% CI)**		**65**		**63**	**100.0%**	**0.24 [0.10, 0.58]**
Total events	9		25			
Heterogeneity: Not applicable						
Test for overall effect: Z = 3.19 (P = 0.001)						
**Compound Kushen injection vs Mitomycin(One trial)**						
Zhang, X. 2011	22	50	21	50	100.0%	1.09 [0.49, 2.40]
**Subtotal (95% CI)**		**50**		**50**	**100.0%**	**1.09 [0.49, 2.40]**
Total events	22		21			
Heterogeneity: Not applicable						
Test for overall effect: Z = 0.20 (P = 0.84)						

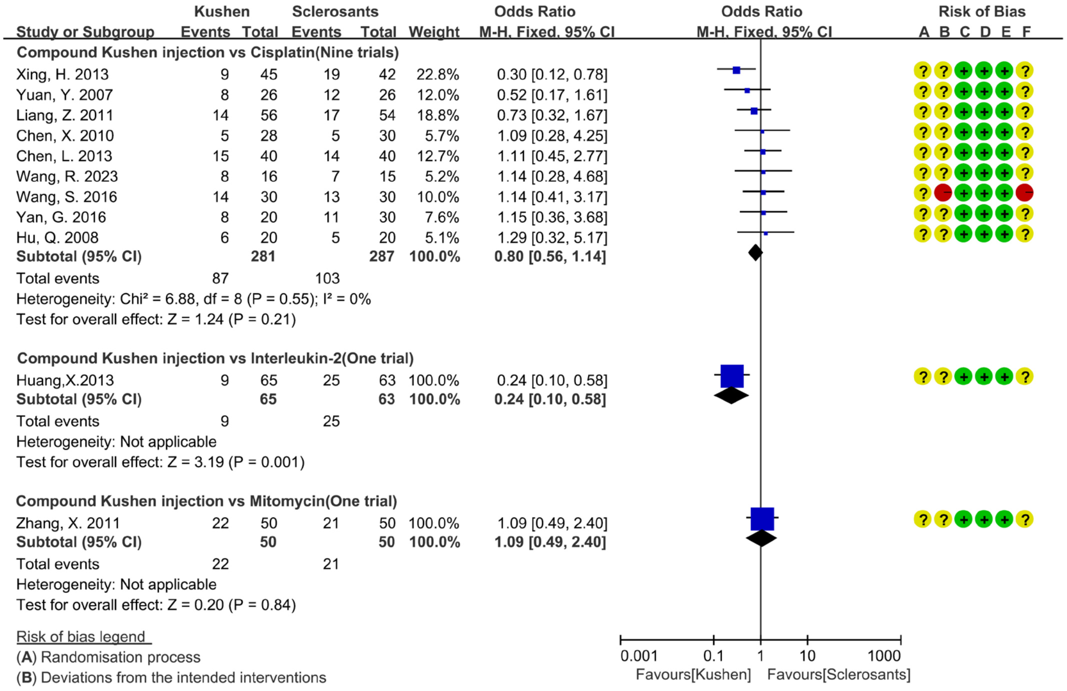

Risk of bias legend
(**A**) Randomisation process
(**B**) Deviations from the intended interventions
(**C**) Missing outcome data
(**D**) Measurement of the outcome
(**E**) Selection of the reported result
(**F**) Overall bias

Figure.S7 The pleurodesis failure of compound Kushen injection alone

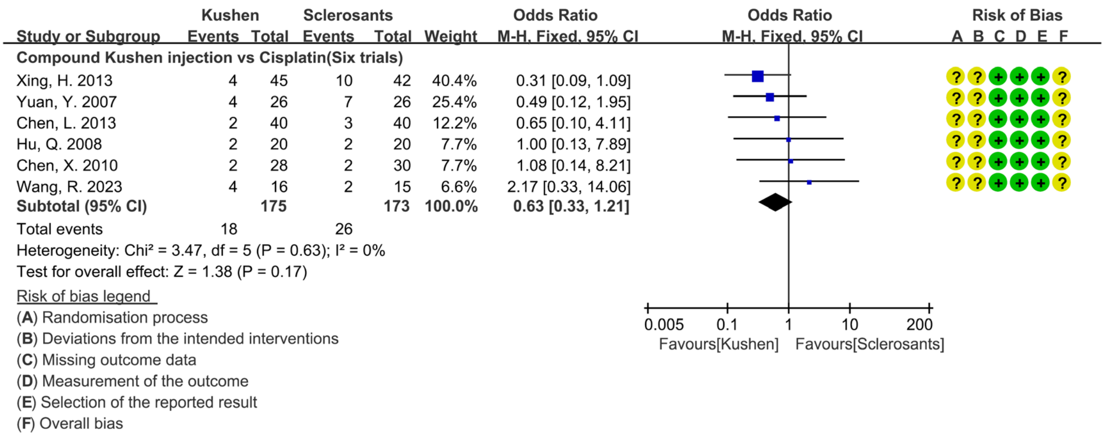

Figure.S8 The pleural progression of compound Kushen injection alone

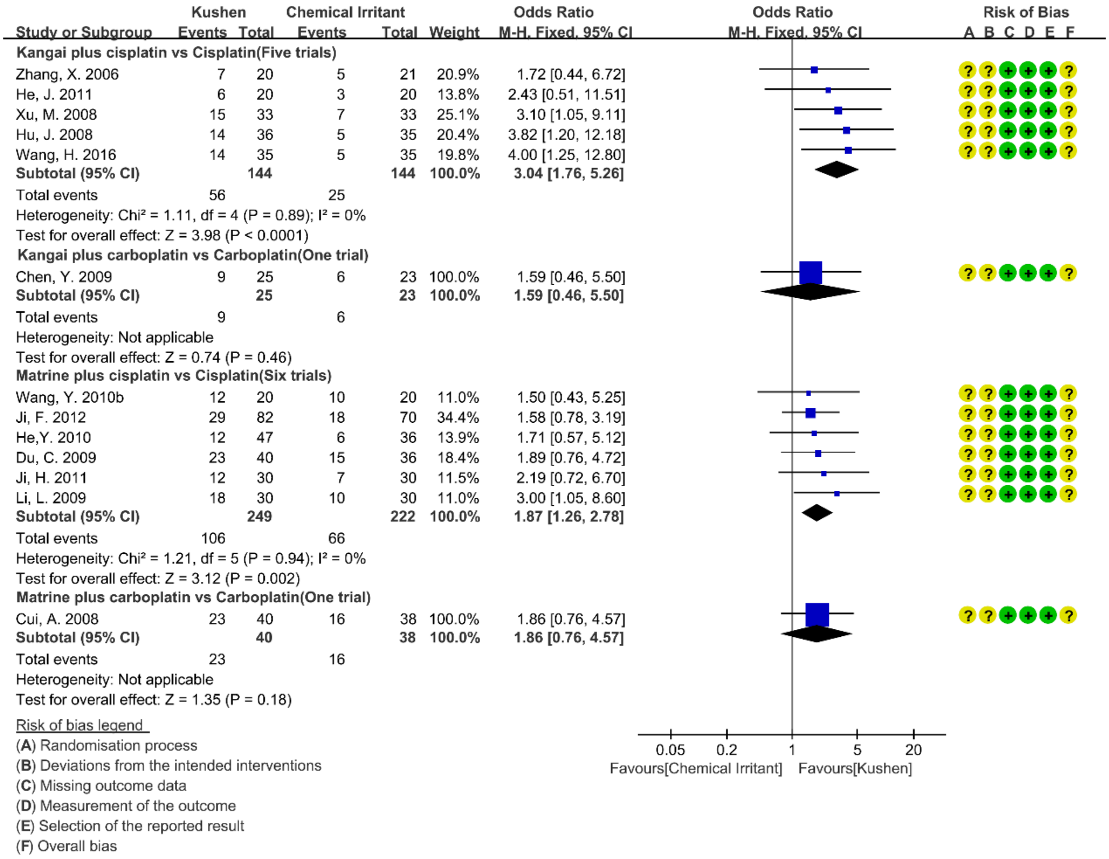

Figure.S9 The complete response of Kangai or Matrine and sclerosants

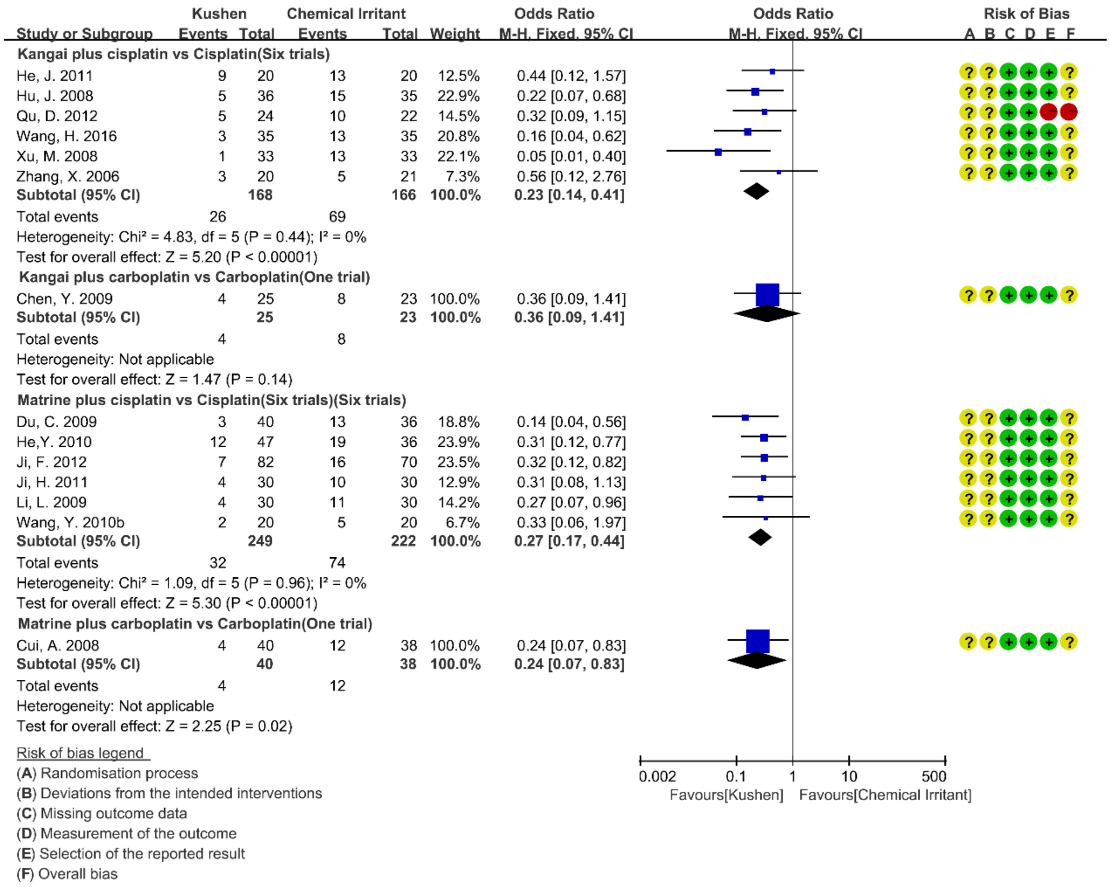

Figure.S10 The pleurodesis failure of Kangai or Matrine and sclerosants

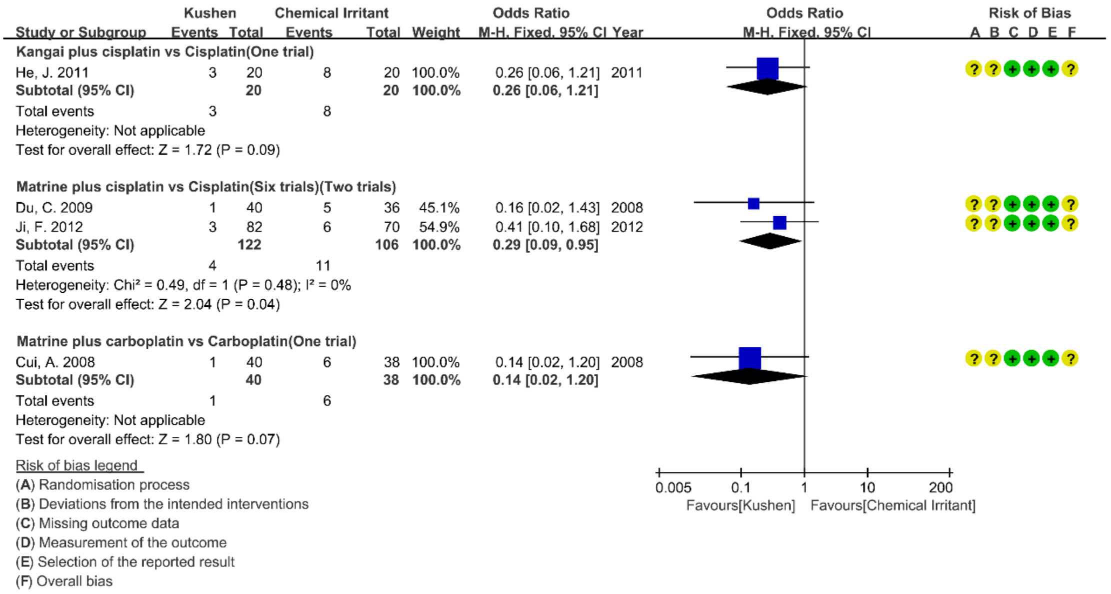

Figure.S11 The pleural progression of Kangai or Matrine and sclerosants

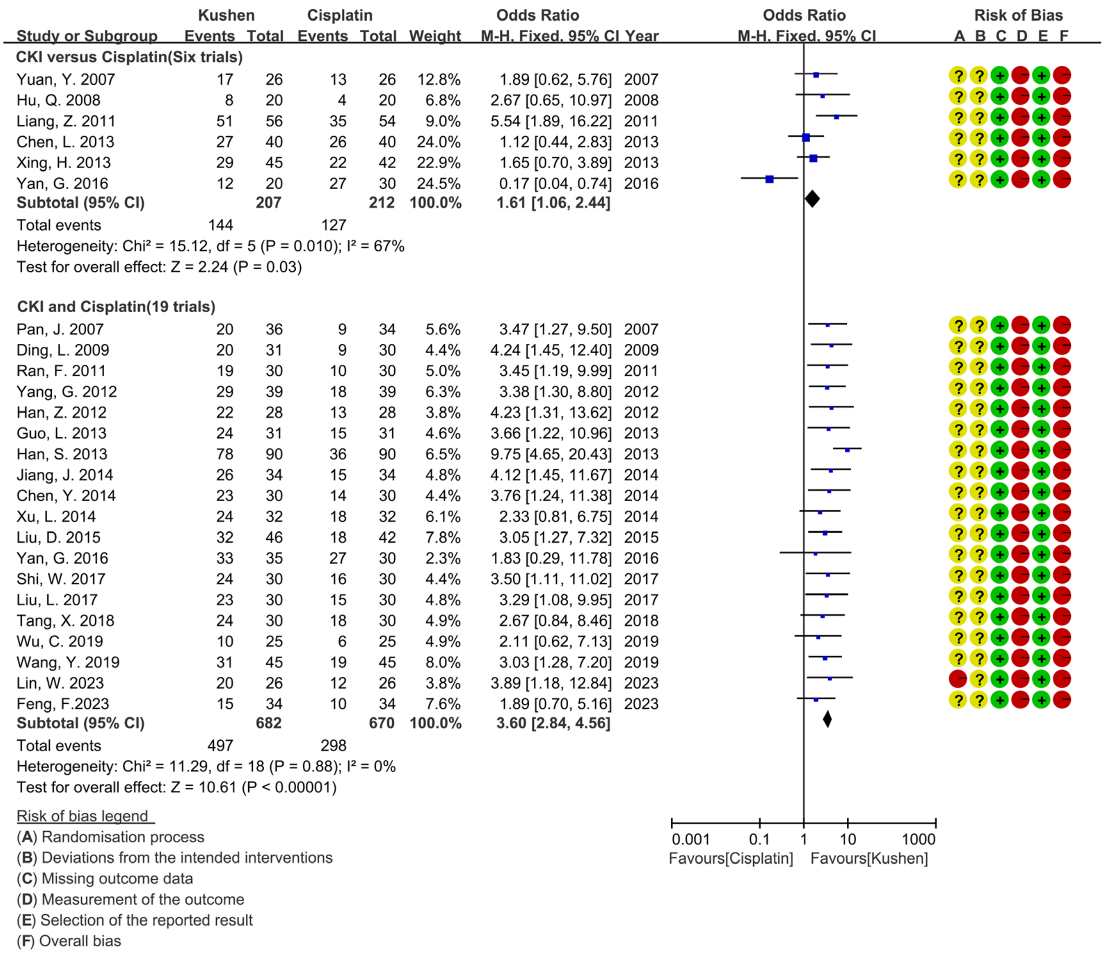

Figure.S12 The QOL of CKI alone or and cisplatin

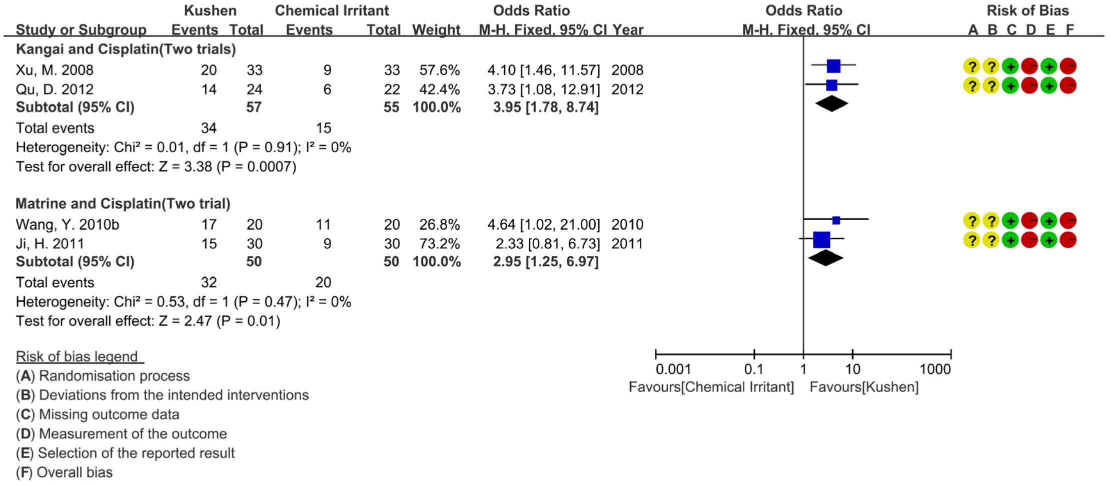

Figure.S13 The QOL of Kangai or Matrine and cisplatin

Study or Subgroup	Kushen Events	Kushen Total	Cisplatin Events	Cisplatin Total	Weight	Odds Ratio M-H, Fixed, 95% CI
**CKI versus Cisplatin(Six trials)**						
Liang, Z. 2011	0	56	18	54	17.7%	0.02 [0.00, 0.30]
Wang, R.2023	3	16	5	15	4.0%	0.46 [0.09, 2.41]
Wang, S. 2016	0	30	7	30	7.0%	0.05 [0.00, 0.95]
Xing, H. 2013	0	45	33	42	32.4%	0.00 [0.00, 0.06]
Yan, G. 2016	0	20	25	30	19.0%	0.01 [0.00, 0.10]
Yuan, Y. 2007	0	26	21	26	20.0%	0.00 [0.00, 0.09]
**Subtotal (95% CI)**		**193**		**197**	**100.0%**	**0.03 [0.01, 0.07]**
Total events	3		109			
Heterogeneity: Chi² = 16.16, df = 5 (P = 0.006); I² = 69%						
Test for overall effect: Z = 7.97 (P < 0.00001)						
**CKI plus Cisplatin(17 trials)**						
Chen, F.2012	2	43	4	43	2.9%	0.48 [0.08, 2.75]
Ding, L. 2009	31	31	30	30		Not estimable
Feng, F.2023	4	34	11	34	7.4%	0.28 [0.08, 0.99]
He, L. 2010	9	24	9	20	4.7%	0.73 [0.22, 2.45]
Huang, H. 2017	14	30	22	30	9.0%	0.32 [0.11, 0.94]
Jiang, J. 2014	1	34	3	34	2.2%	0.31 [0.03, 3.17]
Li, L. 2011	0	30	0	30		Not estimable
Lin, S. 2007	6	33	12	33	7.5%	0.39 [0.13, 1.21]
Lin, W. 2023	21	26	25	26	3.7%	0.17 [0.02, 1.55]
Liu, D. 2015	0	46	0	42		Not estimable
Song, X. 2015	14	59	26	59	15.2%	0.39 [0.18, 0.87]
Wu, C. 2019	0	25	8	25	6.4%	0.04 [0.00, 0.75]
Xu, B. 2014	8	30	12	30	6.7%	0.55 [0.18, 1.62]
Yan, G. 2016	20	35	25	30	8.8%	0.27 [0.08, 0.86]
Yang, G. 2012	10	39	20	39	11.4%	0.33 [0.13, 0.85]
Zheng, S. 2013	6	31	17	31	10.5%	0.20 [0.06, 0.62]
Zhuang, H. 2012	3	24	5	22	3.5%	0.49 [0.10, 2.33]
**Subtotal (95% CI)**		**574**		**558**	**100.0%**	**0.34 [0.24, 0.47]**
Total events	149		229			
Heterogeneity: Chi² = 6.42, df = 13 (P = 0.93); I² = 0%						
Test for overall effect: Z = 6.57 (P < 0.00001)						
**Matrine plus Cisplatin(Three trials)**						
He,Y. 2010	13	47	13	36	64.7%	0.68 [0.27, 1.72]
Ji, H. 2011	1	30	6	30	35.3%	0.14 [0.02, 1.23]
Wang, Y. 2010b	0	20	0	20		Not estimable
**Subtotal (95% CI)**		**97**		**86**	**100.0%**	**0.49 [0.21, 1.11]**
Total events	14		19			
Heterogeneity: Chi² = 1.76, df = 1 (P = 0.18); I² = 43%						
Test for overall effect: Z = 1.71 (P = 0.09)						

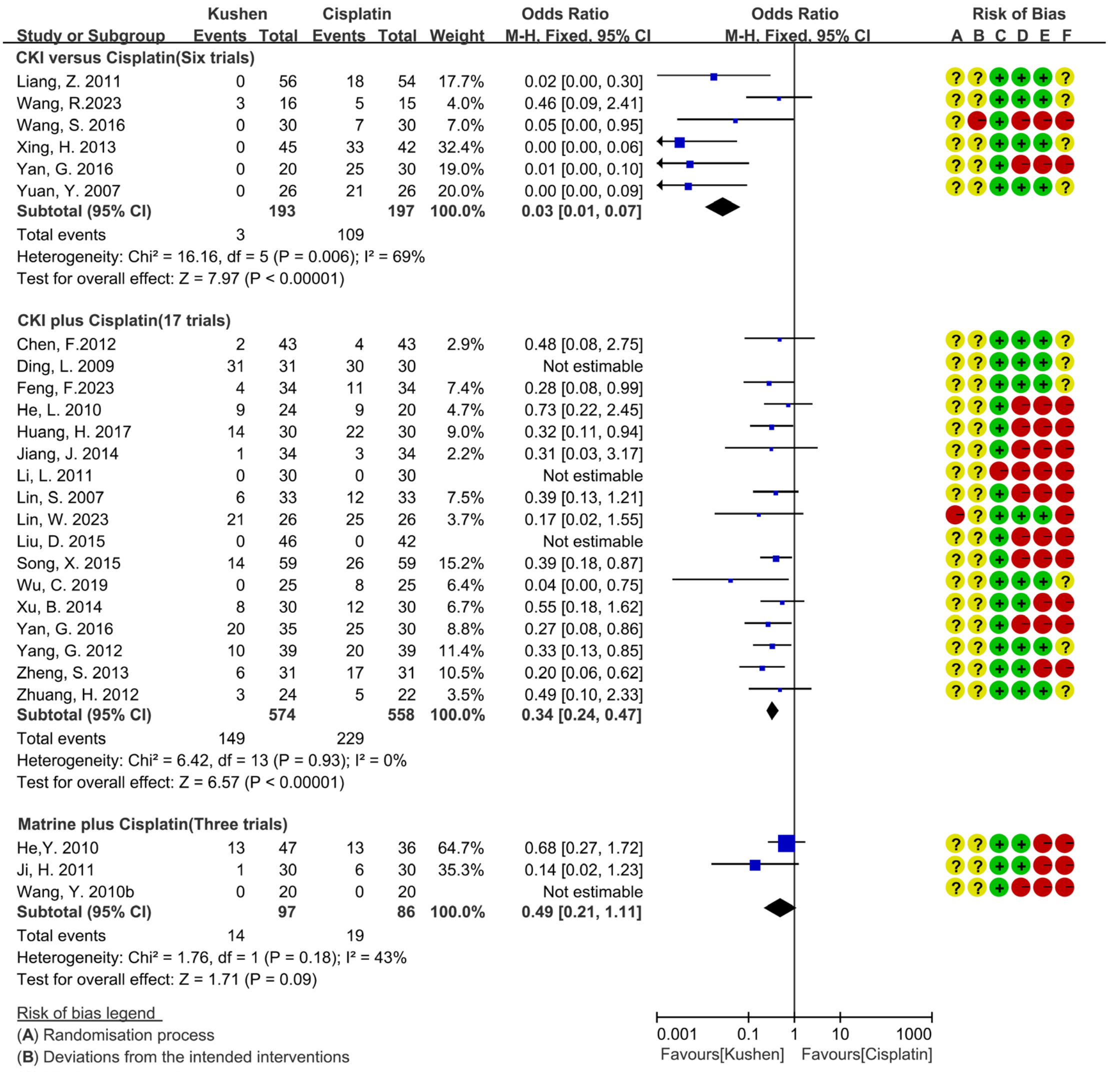

Risk of bias legend
(**A**) Randomisation process
(**B**) Deviations from the intended interventions
(**C**) Missing outcome data
(**D**) Measurement of the outcome
(**E**) Selection of the reported result
(**F**) Overall bias

Figure.S14 The analysis of myelosuppression between the two groups

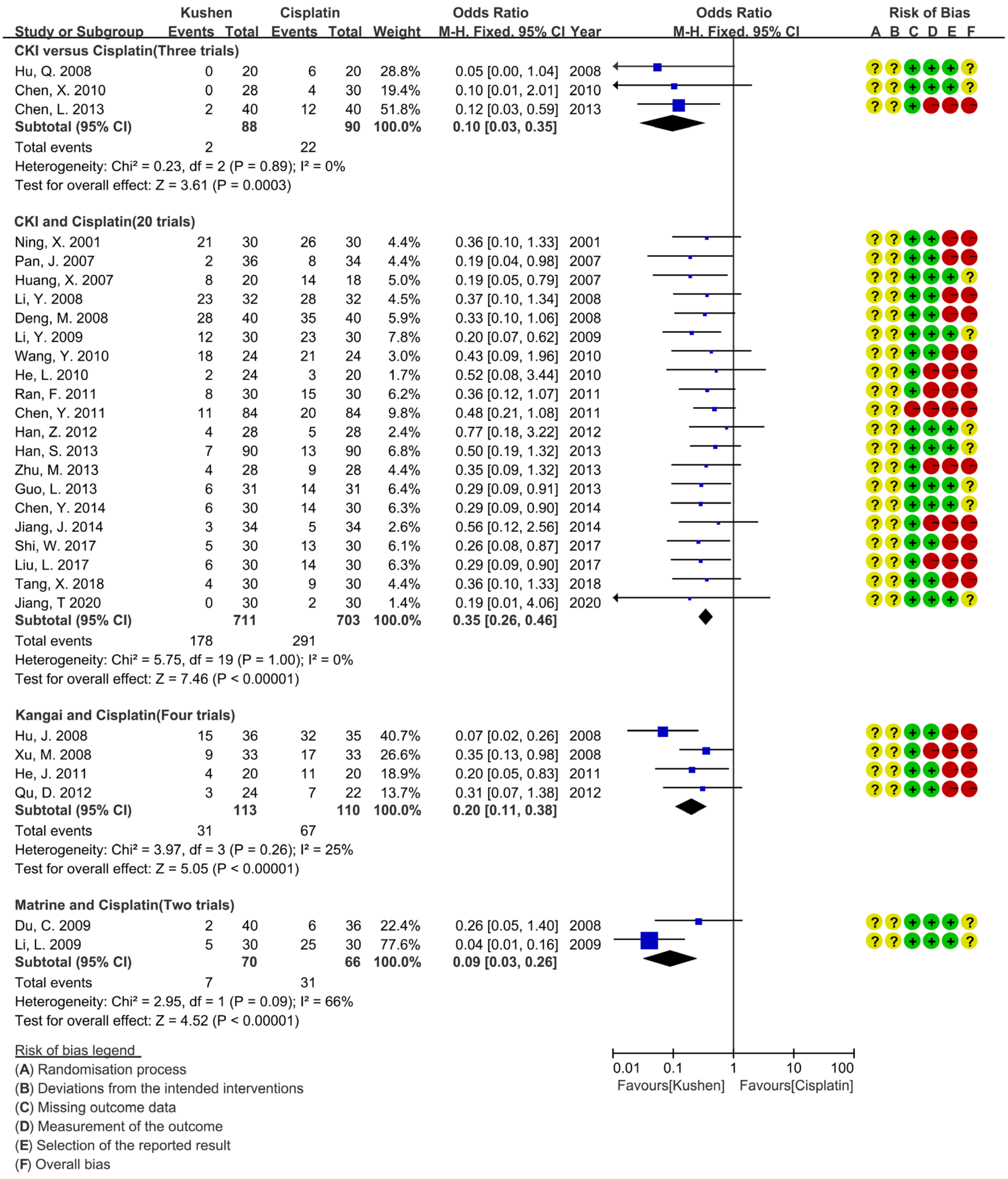

Figure.S15 The analysis of leukopenia between the two groups

Study or Subgroup	Kushen Events	Kushen Total	Cisplatin Events	Cisplatin Total	Weight	Odds Ratio M-H, Fixed, 95% CI	Year
**CKI and Cisplatin(Two trials)**							
Pan, J. 2007	1	36	2	34	29.6%	0.46 [0.04, 5.29]	2007
Chen, Y. 2011	4	84	5	84	70.4%	0.79 [0.20, 3.05]	2011
**Subtotal (95% CI)**		**120**		**118**	**100.0%**	**0.69 [0.21, 2.24]**	
Total events	5		7				
Heterogeneity: Chi² = 0.15, df = 1 (P = 0.70); I² = 0%							
Test for overall effect: Z = 0.61 (P = 0.54)							
**Matrine and Cisplatin(One trial)**							
Li, L. 2009	14	30	26	30	100.0%	0.13 [0.04, 0.48]	2009
**Subtotal (95% CI)**		**30**		**30**	**100.0%**	**0.13 [0.04, 0.48]**	
Total events	14		26				
Heterogeneity: Not applicable							
Test for overall effect: Z = 3.09 (P = 0.002)							

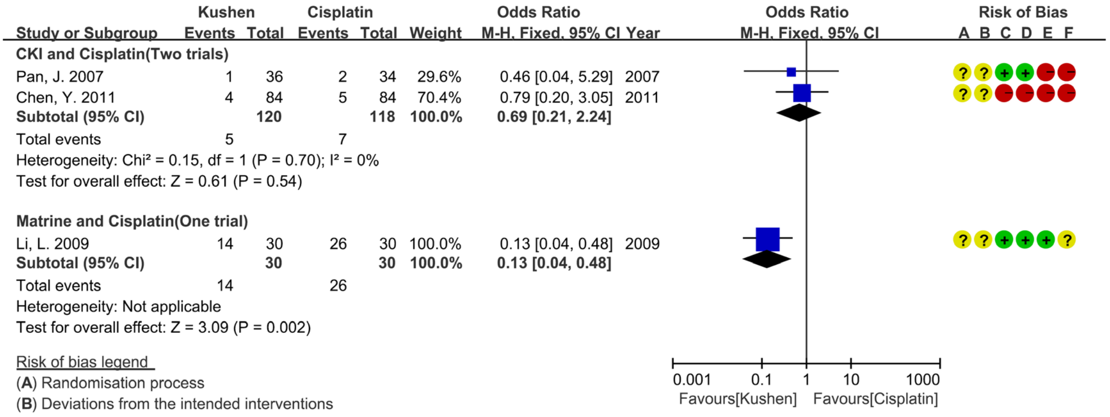

Risk of bias legend
(**A**) Randomisation process
(**B**) Deviations from the intended interventions
(**C**) Missing outcome data
(**D**) Measurement of the outcome
(**E**) Selection of the reported result
(**F**) Overall bias

Figure.S16 The anemia between the two groups

Study or Subgroup	Kushen Events	Kushen Total	Cisplatin Events	Cisplatin Total	Weight	Odds Ratio M-H, Fixed, 95% CI	Year
**CKI and Cisplatin(Five trials)**							
Pan, J. 2007	0	36	0	34		Not estimable	2007
Chen, Y. 2011	5	84	5	84	55.5%	1.00 [0.28, 3.59]	2011
Guo, L. 2013	0	31	0	31		Not estimable	2013
Chen, Y. 2014	0	30	0	30		Not estimable	2014
Jiang, J. 2014	2	34	4	34	44.5%	0.47 [0.08, 2.75]	2014
**Subtotal (95% CI)**		**215**		**213**	**100.0%**	**0.76 [0.27, 2.12]**	
Total events	7		9				
Heterogeneity: Chi² = 0.46, df = 1 (P = 0.50); I² = 0%							
Test for overall effect: Z = 0.52 (P = 0.61)							
**Matrine and Cisplatin(One trial)**							
Li, L. 2009	8	30	9	30	100.0%	0.85 [0.28, 2.61]	2009
**Subtotal (95% CI)**		**30**		**30**	**100.0%**	**0.85 [0.28, 2.61]**	
Total events	8		9				
Heterogeneity: Not applicable							
Test for overall effect: Z = 0.29 (P = 0.77)							

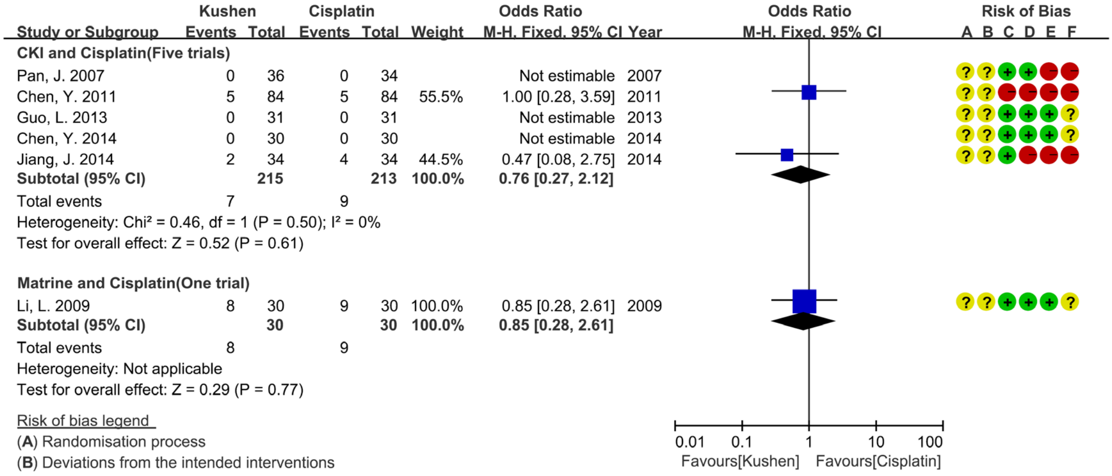

Risk of bias legend
(**A**) Randomisation process
(**B**) Deviations from the intended interventions
(**C**) Missing outcome data
(**D**) Measurement of the outcome
(**E**) Selection of the reported result
(**F**) Overall bias

Figure.S17 The thrombocytopenia between the two groups

Study or Subgroup	Kushen Events	Kushen Total	Cisplatin Events	Cisplatin Total	Weight	Odds Ratio M-H, Fixed, 95% CI	Year
**CKI plus Cisplatin(Nine trials)**							
Yuan, Y. 2007	0	26	16	26	10.1%	0.01 [0.00, 0.22]	2007
Hu, Q. 2008	0	20	6	20	4.0%	0.05 [0.00, 1.04]	2008
Chen, X. 2010	0	28	16	30	9.8%	0.02 [0.00, 0.28]	2010
Liang, Z. 2011	1	56	49	54	30.6%	0.00 [0.00, 0.02]	2011
Chen, L. 2013	3	40	22	40	12.7%	0.07 [0.02, 0.25]	2013
Xing, H. 2013	0	45	18	42	11.8%	0.01 [0.00, 0.25]	2013
Wang, S. 2016	2	30	13	30	7.6%	0.09 [0.02, 0.47]	2016
Yan, G. 2016	0	20	22	30	11.1%	0.01 [0.00, 0.17]	2016
Wang, R.2023	4	16	5	15	2.4%	0.67 [0.14, 3.17]	2023
**Subtotal (95% CI)**		**281**		**287**	**100.0%**	**0.04 [0.02, 0.07]**	
Total events	10		167				
Heterogeneity: Chi² = 24.34, df = 8 (P = 0.002); I² = 67%							
Test for overall effect: Z = 10.50 (P < 0.00001)							
**CKI plus Cisplatin (Thirty-one trials)**							
Huang, X. 2007	2	20	5	18	1.6%	0.29 [0.05, 1.73]	2007
Lin, S. 2007	6	33	16	33	4.3%	0.24 [0.08, 0.72]	2007
Li, Y. 2009	3	30	8	30	2.4%	0.31 [0.07, 1.29]	2009
Ding, L. 2009	22	31	28	30	2.7%	0.17 [0.03, 0.89]	2009
He, L. 2010	16	24	15	20	1.8%	0.67 [0.18, 2.50]	2010
Ran, F. 2011	12	30	19	30	3.8%	0.39 [0.14, 1.09]	2011
Zhuang, H. 2012	2	24	4	22	1.3%	0.41 [0.07, 2.49]	2012
Yang, G. 2012	14	39	24	39	5.1%	0.35 [0.14, 0.88]	2012
Chen, F.2012	16	43	22	43	4.6%	0.57 [0.24, 1.34]	2012
Han, Z. 2012	6	28	7	28	1.8%	0.82 [0.24, 2.84]	2012
Zheng, S. 2013	7	31	15	31	3.8%	0.31 [0.10, 0.93]	2013
Zhu, M. 2013	10	28	18	28	3.8%	0.31 [0.10, 0.92]	2013
Guo, L. 2013	4	31	11	31	3.2%	0.27 [0.07, 0.97]	2013
Han, S. 2013	16	90	28	90	7.6%	0.48 [0.24, 0.96]	2013
Jiang, J. 2014	2	34	4	34	1.2%	0.47 [0.08, 2.75]	2014
Xu, B. 2014	8	30	11	30	2.7%	0.63 [0.21, 1.88]	2014
Chen, Y. 2014	4	30	11	30	3.1%	0.27 [0.07, 0.96]	2014
Liu, D. 2015	8	46	7	42	2.0%	1.05 [0.35, 3.21]	2015
Song, X. 2015	9	59	18	59	5.0%	0.41 [0.17, 1.01]	2015
Yan, G. 2016	16	35	22	30	4.2%	0.31 [0.11, 0.87]	2016
Qin,D.2016	3	32	15	32	4.5%	0.12 [0.03, 0.46]	2016
Huang, H. 2017	15	30	21	30	3.5%	0.43 [0.15, 1.24]	2017
Liu, L. 2017	5	30	13	30	3.6%	0.26 [0.08, 0.87]	2017
Shi, W. 2017	5	30	14	30	3.9%	0.23 [0.07, 0.76]	2017
Tang, X. 2018	6	30	13	30	3.4%	0.33 [0.10, 1.03]	2018
Wu, C. 2019	1	25	23	25	7.3%	0.00 [0.00, 0.04]	2019
Wang, Y. 2019	8	45	7	45	1.9%	1.17 [0.39, 3.56]	2019
Jiang, T 2020	1	30	3	30	1.0%	0.31 [0.03, 3.17]	2020
Peng,H.2020	0	25	1	25	0.5%	0.32 [0.01, 8.25]	2020
Lin, W. 2023	23	26	25	26	1.0%	0.31 [0.03, 3.16]	2023
Feng, F.2023	4	34	12	34	3.5%	0.24 [0.07, 0.86]	2023
**Subtotal (95% CI)**		**1053**		**1035**	**100.0%**	**0.36 [0.29, 0.44]**	
Total events	254		440				
Heterogeneity: Chi² = 32.63, df = 30 (P = 0.34); I² = 8%							
Test for overall effect: Z = 9.64 (P < 0.00001)							
**Kangai plus Cisplatin(Five trials)**							
Zhang, X. 2006	1	20	2	21	5.1%	0.50 [0.04, 5.99]	2006
Xu, M. 2008	14	33	21	33	33.0%	0.42 [0.16, 1.13]	2008
Hu, J. 2008	22	36	30	35	32.3%	0.26 [0.08, 0.84]	2008
He, J. 2011	2	20	5	20	12.3%	0.33 [0.06, 1.97]	2011
Qu, D. 2012	3	24	7	22	17.4%	0.31 [0.07, 1.38]	2012
**Subtotal (95% CI)**		**133**		**131**	**100.0%**	**0.34 [0.19, 0.63]**	
Total events	42		65				
Heterogeneity: Chi² = 0.48, df = 4 (P = 0.98); I² = 0%							
Test for overall effect: Z = 3.45 (P = 0.0006)							
**Matrine plus Cisplatin(Five trials)**							
Du, C. 2009	2	40	4	36	11.7%	0.42 [0.07, 2.45]	2008
Li, L. 2009	20	30	28	30	27.2%	0.14 [0.03, 0.72]	2009
He,Y. 2010	11	47	11	36	27.8%	0.69 [0.26, 1.85]	2010
Wang, Y. 2010b	0	20	2	20	7.1%	0.18 [0.01, 4.01]	2010
Ji, H. 2011	3	30	10	30	26.2%	0.22 [0.05, 0.91]	2011
**Subtotal (95% CI)**		**167**		**152**	**100.0%**	**0.35 [0.19, 0.66]**	
Total events	36		55				
Heterogeneity: Chi² = 3.66, df = 4 (P = 0.45); I² = 0%							
Test for overall effect: Z = 3.26 (P = 0.001)							

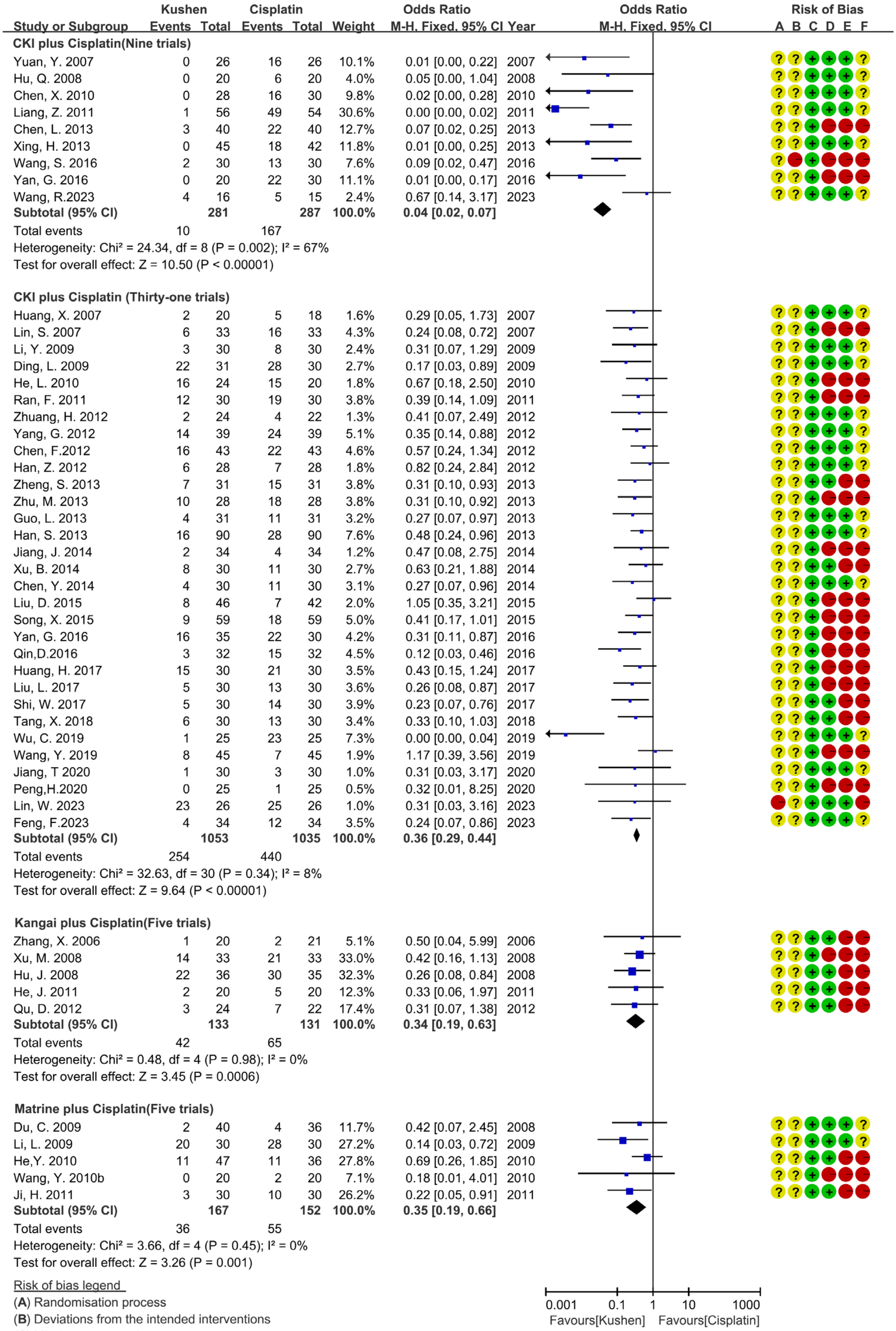

Risk of bias legend
(**A**) Randomisation process
(**B**) Deviations from the intended interventions
(**C**) Missing outcome data
(**D**) Measurement of the outcome
(**E**) Selection of the reported result
(**F**) Overall bias

Figure.S18 The gastrointestinal reactions between the two groups

Study or Subgroup	Kushen Events	Kushen Total	Cisplatin Events	Cisplatin Total	Weight	Odds Ratio M-H, Fixed, 95% CI	Year
**CKI versus Cisplatin(Six trials)**							
Yuan, Y. 2007	0	26	2	26	10.4%	0.18 [0.01, 4.05]	2007
Chen, X. 2010	0	28	0	30		Not estimable	2010
Liang, Z. 2011	0	56	14	54	61.9%	0.02 [0.00, 0.43]	2011
Xing, H. 2013	0	45	4	42	19.5%	0.09 [0.00, 1.80]	2013
Wang, S. 2016	0	30	0	30		Not estimable	2016
Wang, R.2023	1	16	2	15	8.2%	0.43 [0.04, 5.35]	2023
**Subtotal (95% CI)**		**201**		**197**	**100.0%**	**0.09 [0.02, 0.33]**	
Total events	1		22				
Heterogeneity: Chi² = 2.53, df = 3 (P = 0.47); I² = 0%							
Test for overall effect: Z = 3.62 (P = 0.0003)							
**CKI plus Cisplatin(22 trials)**							
Lin, S. 2007	0	33	0	33		Not estimable	2007
Pan, J. 2007	1	36	2	34	2.6%	0.46 [0.04, 5.29]	2007
Ding, L. 2009	0	31	0	30		Not estimable	2009
He, L. 2010	0	24	0	20		Not estimable	2010
Li, L. 2011	0	30	0	30		Not estimable	2011
Chen, Y. 2011	3	84	4	84	5.1%	0.74 [0.16, 3.42]	2011
Han, Z. 2012	3	28	5	28	5.8%	0.55 [0.12, 2.57]	2012
Chen, F.2012	1	43	2	43	2.6%	0.49 [0.04, 5.59]	2012
Zhuang, H. 2012	2	24	4	22	5.0%	0.41 [0.07, 2.49]	2012
Yang, G. 2012	9	39	17	39	17.1%	0.39 [0.15, 1.03]	2012
Han, S. 2013	7	90	15	90	18.1%	0.42 [0.16, 1.09]	2013
Zhu, M. 2013	0	28	0	28		Not estimable	2013
Guo, L. 2013	0	31	0	31		Not estimable	2013
Jiang, J. 2014	2	34	3	34	3.7%	0.65 [0.10, 4.13]	2014
Chen, Y. 2014	0	30	0	30		Not estimable	2014
Song, X. 2015	12	59	23	59	24.0%	0.40 [0.18, 0.91]	2015
Liu, D. 2015	0	46	0	42		Not estimable	2015
Qin,D.2016	0	32	0	32		Not estimable	2016
Wu, C. 2019	0	25	6	25	8.4%	0.06 [0.00, 1.11]	2019
Jiang, T 2020	1	30	3	30	3.8%	0.31 [0.03, 3.17]	2020
Lin, W. 2023	1	26	1	26	1.3%	1.00 [0.06, 16.89]	2023
Feng, F.2023	1	34	2	34	2.5%	0.48 [0.04, 5.61]	2023
**Subtotal (95% CI)**		**837**		**824**	**100.0%**	**0.42 [0.28, 0.63]**	
Total events	43		87				
Heterogeneity: Chi² = 3.08, df = 12 (P = 0.99); I² = 0%							
Test for overall effect: Z = 4.24 (P < 0.0001)							
**Kangai plus Cisplatin(One trial)**							
Qu, D. 2012	0	24	0	22		Not estimable	2012
**Subtotal (95% CI)**		**24**		**22**		**Not estimable**	
Total events	0		0				
Heterogeneity: Not applicable							
Test for overall effect: Not applicable							
**Matrine plus Cisplatin(Three trials)**							
Du, C. 2009	1	40	3	36	18.3%	0.28 [0.03, 2.84]	2008
Li, L. 2009	10	30	16	30	63.3%	0.44 [0.15, 1.24]	2009
He,Y. 2010	4	47	3	36	18.4%	1.02 [0.21, 4.89]	2010
**Subtotal (95% CI)**		**117**		**102**	**100.0%**	**0.52 [0.23, 1.15]**	
Total events	15		22				
Heterogeneity: Chi² = 1.09, df = 2 (P = 0.58); I² = 0%							
Test for overall effect: Z = 1.62 (P = 0.10)							

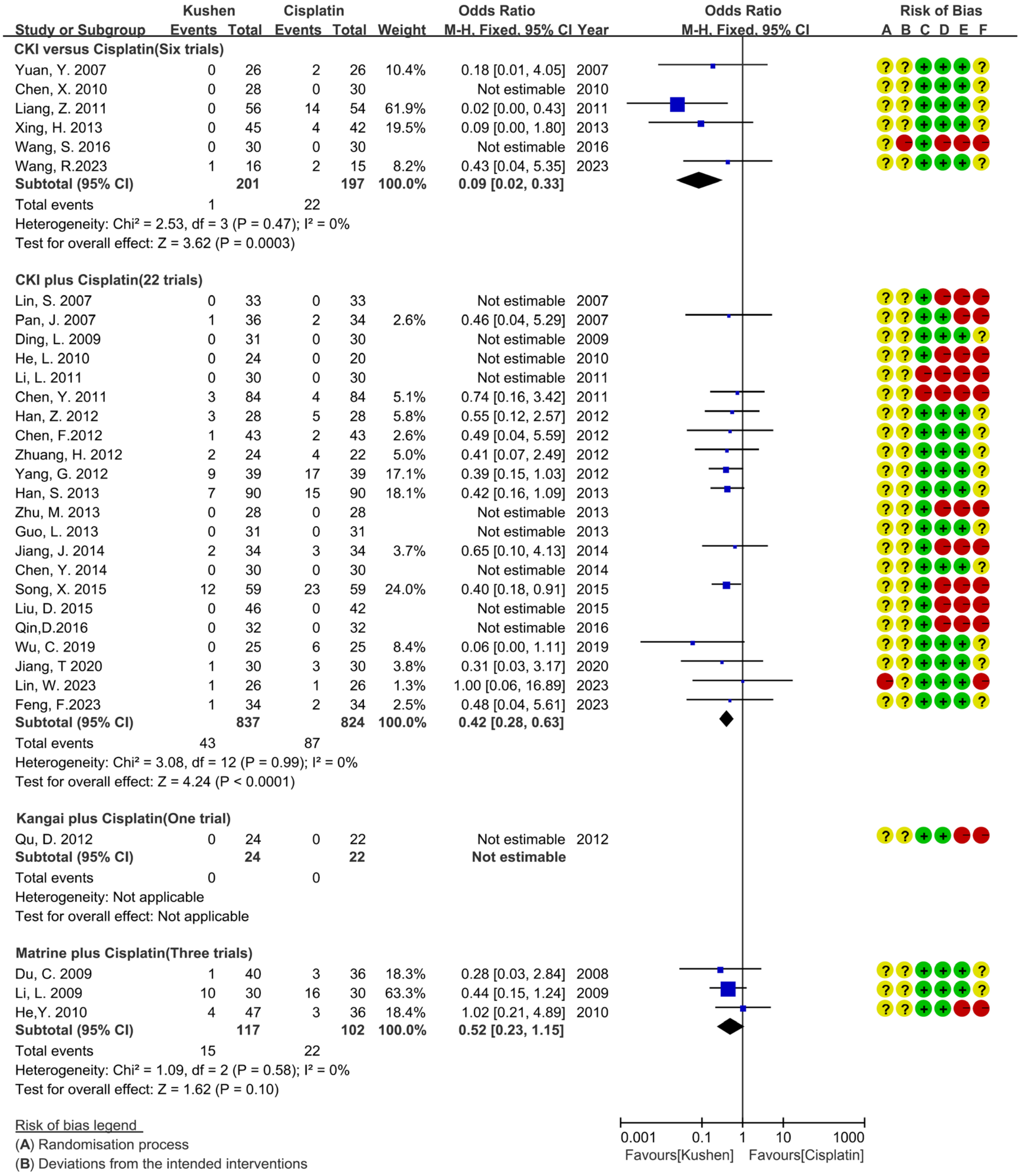

Risk of bias legend
(**A**) Randomisation process
(**B**) Deviations from the intended interventions
(**C**) Missing outcome data
(**D**) Measurement of the outcome
(**E**) Selection of the reported result
(**F**) Overall bias

Figure.S19 The analysis of hepatotoxicity between the two groups

Study or Subgroup	Kushen Events	Kushen Total	Cisplatin Events	Cisplatin Total	Weight	Odds Ratio M-H, Fixed, 95% CI	Year
**CKI versus Cisplatin(Seven trials)**							
Yuan, Y. 2007	0	26	2	26	8.8%	0.18 [0.01, 4.05]	2007
Hu, Q. 2008	0	20	5	20	19.2%	0.07 [0.00, 1.34]	2008
Chen, X. 2010	0	28	0	30		Not estimable	2010
Liang, Z. 2011	0	56	14	54	52.3%	0.02 [0.00, 0.43]	2011
Xing, H. 2013	0	45	3	42	12.8%	0.12 [0.01, 2.48]	2013
Wang, S. 2016	0	30	0	30		Not estimable	2016
Wang, R.2023	1	16	2	15	6.9%	0.43 [0.04, 5.35]	2023
**Subtotal (95% CI)**		**221**		**217**	**100.0%**	**0.09 [0.03, 0.29]**	
Total events	1		26				
Heterogeneity: Chi² = 2.61, df = 4 (P = 0.63); I² = 0%							
Test for overall effect: Z = 3.96 (P < 0.0001)							
**CKI plus Cisplatin(31 trials)**							
Ning, X. 2001	5	30	14	30	8.3%	0.23 [0.07, 0.76]	2001
Pan, J. 2007	0	36	1	34	1.1%	0.31 [0.01, 7.77]	2007
Lin, S. 2007	0	33	0	33		Not estimable	2007
Huang, X. 2007	5	20	10	18	5.6%	0.27 [0.07, 1.05]	2007
Deng, M. 2008	7	40	19	40	11.1%	0.23 [0.08, 0.65]	2008
Li, Y. 2008	8	32	20	32	10.7%	0.20 [0.07, 0.59]	2008
Li, Y. 2009	7	30	13	30	7.1%	0.40 [0.13, 1.21]	2009
Ding, L. 2009	0	31	0	30		Not estimable	2009
Wang, Y. 2010	8	24	14	24	6.6%	0.36 [0.11, 1.16]	2010
He, L. 2010	0	24	0	20		Not estimable	2010
Chen, Y. 2011	5	84	7	84	4.7%	0.70 [0.21, 2.29]	2011
Li, L. 2011	0	30	0	30		Not estimable	2011
Ran, F. 2011	0	30	0	30		Not estimable	2011
Han, Z. 2012	0	28	1	28	1.0%	0.32 [0.01, 8.24]	2012
Zhuang, H. 2012	2	24	4	22	2.7%	0.41 [0.07, 2.49]	2012
Yang, G. 2012	8	39	15	39	8.5%	0.41 [0.15, 1.13]	2012
Chen, F.2012	0	43	1	43	1.1%	0.33 [0.01, 8.22]	2012
Guo, L. 2013	0	31	0	31		Not estimable	2013
Han, S. 2013	2	90	8	90	5.6%	0.23 [0.05, 1.13]	2013
Zheng, S. 2013	0	31	0	31		Not estimable	2013
Zhu, M. 2013	0	28	0	28		Not estimable	2013
Jiang, J. 2014	2	34	3	34	2.0%	0.65 [0.10, 4.13]	2014
Xu, B. 2014	1	30	2	30	1.4%	0.48 [0.04, 5.63]	2014
Chen, Y. 2014	0	30	0	30		Not estimable	2014
Liu, D. 2015	0	46	0	42		Not estimable	2015
Song, X. 2015	13	60	25	60	13.9%	0.39 [0.17, 0.86]	2015
Qin,D.2016	0	32	0	32		Not estimable	2016
Wu, C. 2019	0	25	7	25	5.2%	0.05 [0.00, 0.90]	2019
Jiang, T 2020	1	30	3	30	2.1%	0.31 [0.03, 3.17]	2020
Feng, F.2023	1	34	2	34	1.4%	0.48 [0.04, 5.61]	2023
Lin, W. 2023	0	26	0	26		Not estimable	2023
**Subtotal (95% CI)**		**1105**		**1090**	**100.0%**	**0.32 [0.24, 0.44]**	
Total events	75		169				
Heterogeneity: Chi² = 6.31, df = 18 (P = 0.99); I² = 0%							
Test for overall effect: Z = 6.98 (P < 0.00001)							
**Kangai plus Cisplatin(Two trials)**							
Xu, M. 2008	0	33	0	33		Not estimable	2008
Qu, D. 2012	0	24	0	22		Not estimable	2012
**Subtotal (95% CI)**		**57**		**55**		**Not estimable**	
Total events	0		0				
Heterogeneity: Not applicable							
Test for overall effect: Not applicable							
**Matrine plus Cisplatin(Four trials)**							
Du, C. 2009	1	40	3	36	32.5%	0.28 [0.03, 2.84]	2008
Li, L. 2009	6	30	8	30	67.5%	0.69 [0.21, 2.30]	2009
Wang, Y. 2010b	0	20	0	20		Not estimable	2010
He,Y. 2010	0	47	0	36		Not estimable	2010
**Subtotal (95% CI)**		**137**		**122**	**100.0%**	**0.56 [0.19, 1.59]**	
Total events	7		11				
Heterogeneity: Chi² = 0.45, df = 1 (P = 0.50); I² = 0%							
Test for overall effect: Z = 1.09 (P = 0.27)							

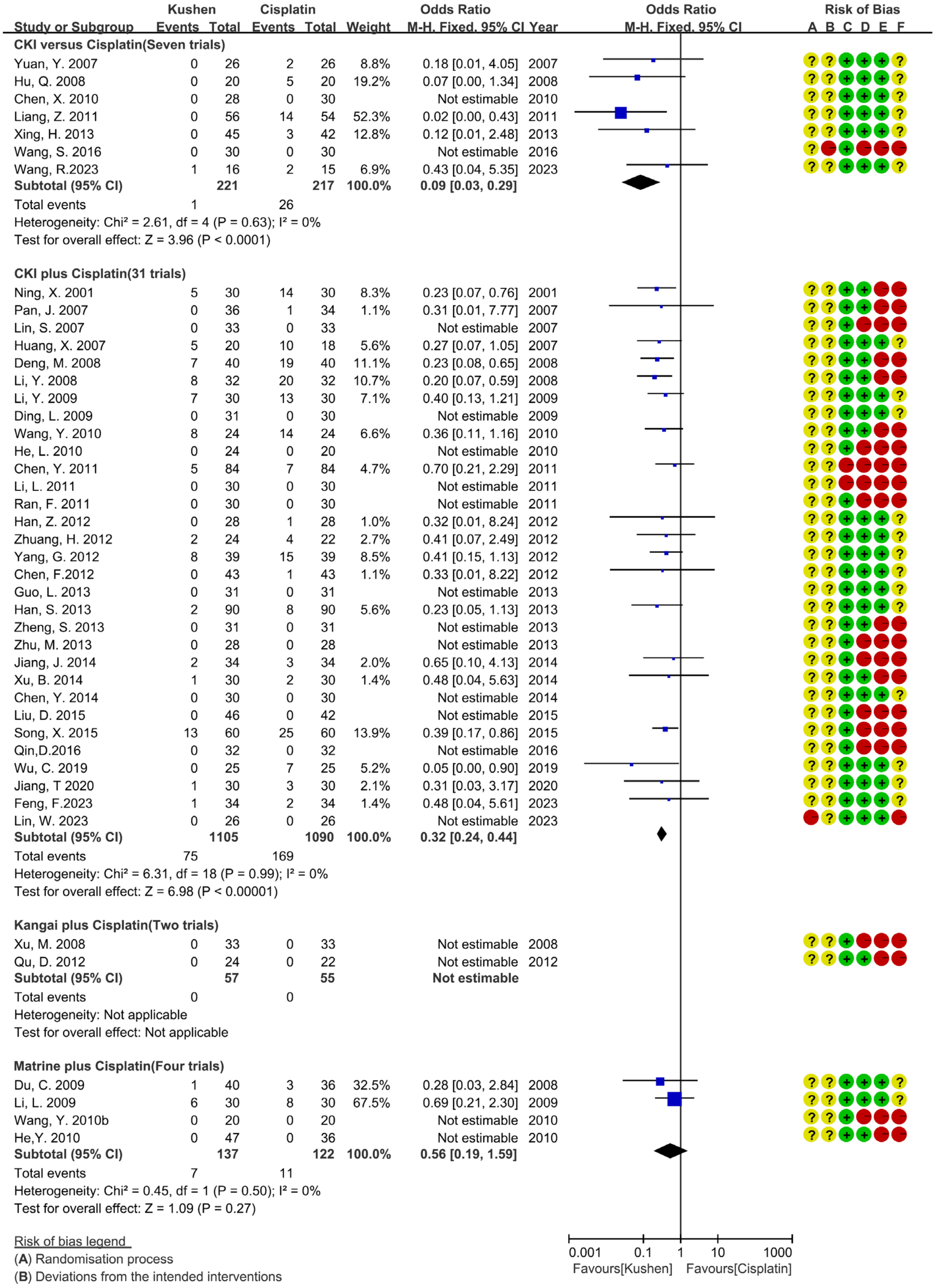

Risk of bias legend
(**A**) Randomisation process
(**B**) Deviations from the intended interventions
(**C**) Missing outcome data
(**D**) Measurement of the outcome
(**E**) Selection of the reported result
(**F**) Overall bias

Figure.S20 The analysis of nephrotoxicity between the two groups

Study or Subgroup	Kushen Events	Kushen Total	Cisplatin Events	Cisplatin Total	Weight	Odds Ratio M-H, Fixed, 95% CI	Year	A	B	C	D	E	F
**CKI versus Cisplatin(One trial)**													
Wang, S. 2016	0	30	0	30		Not estimable	2016	?	−	+	−	−	−
**Subtotal (95% CI)**		**30**		**30**		**Not estimable**							
Total events	0		0										
Heterogeneity: Not applicable													
Test for overall effect: Not applicable													
**CKI and Cisplatin(Five trials)**													
Ding, L. 2009	0	31	0	30		Not estimable	2009	?	?	+	+	+	?
Ran, F. 2011	0	30	0	30		Not estimable	2011	?	?	+	−	−	−
Li, L. 2011	0	30	0	30		Not estimable	2011	?	?	−	−	−	−
Guo, L. 2013	0	31	0	31		Not estimable	2013	?	?	+	+	+	?
Chen, Y. 2014	0	30	0	30		Not estimable	2014	?	?	+	+	+	?
**Subtotal (95% CI)**		**152**		**151**		**Not estimable**							
Total events	0		0										
Heterogeneity: Not applicable													
Test for overall effect: Not applicable													
**Kangai and Cisplatin(Two trials)**													
Xu, M. 2008	0	33	0	33		Not estimable	2008	?	?	+	−	−	−
Qu, D. 2012	0	24	0	22		Not estimable	2012	?	?	+	+	−	−
**Subtotal (95% CI)**		**57**		**55**		**Not estimable**							
Total events	0		0										
Heterogeneity: Not applicable													
Test for overall effect: Not applicable													
**Matrine and Cisplatin(One trial)**													
Wang, Y. 2010b	0	20	0	20		Not estimable	2010	?	?	+	−	−	−
**Subtotal (95% CI)**		**20**		**20**		**Not estimable**							
Total events	0		0										
Heterogeneity: Not applicable													
Test for overall effect: Not applicable													

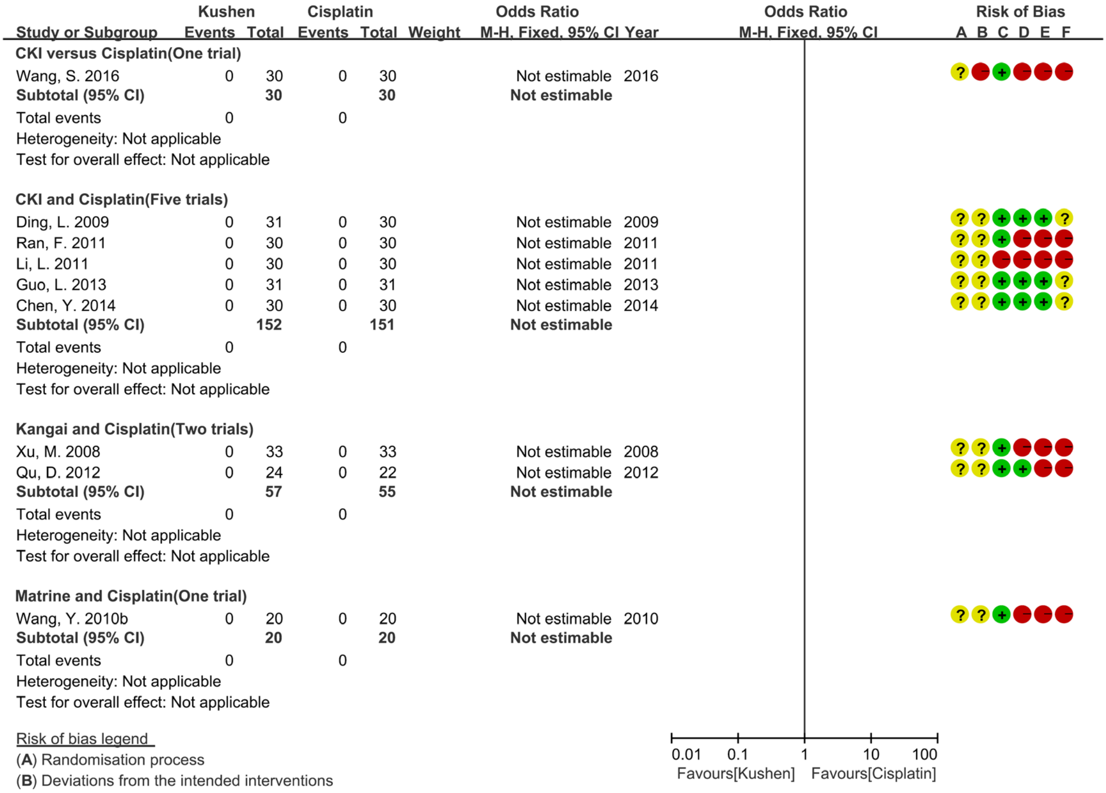

Risk of bias legend

(**A**) Randomisation process
(**B**) Deviations from the intended interventions
(**C**) Missing outcome data
(**D**) Measurement of the outcome
(**E**) Selection of the reported result
(**F**) Overall bias

Figure.S21 The analysis of cardiotoxicity between the two groups

Study or Subgroup	Kushen Events	Kushen Total	Cisplatin Events	Cisplatin Total	Weight	Odds Ratio M-H, Fixed, 95% CI	Year	A	B	C	D	E	F
**CKI versus Cisplatin(Six trials)**													
Yuan, Y. 2007	1	26	11	26	16.5%	0.05 [0.01, 0.47]	2007	?	?	+	+	+	?
Hu, Q. 2008	2	20	8	20	11.2%	0.17 [0.03, 0.92]	2008	?	?	+	+	+	?
Chen, X. 2010	11	28	12	30	11.0%	0.97 [0.34, 2.78]	2010	?	?	+	+	+	?
Xing, H. 2013	2	45	25	42	38.5%	0.03 [0.01, 0.15]	2013	?	?	+	+	+	?
Chen, L. 2013	1	40	7	40	10.6%	0.12 [0.01, 1.03]	2013	?	?	+	-	-	-
Wang, R.2023	5	16	11	15	12.2%	0.17 [0.03, 0.78]	2023	?	?	+	+	+	?
**Subtotal (95% CI)**		**175**		**173**	**100.0%**	**0.18 [0.10, 0.32]**							
Total events	22		74										
Heterogeneity: Chi² = 16.05, df = 5 (P = 0.007); I² = 69%													
Test for overall effect: Z = 5.94 (P < 0.00001)													
**CKI plus Cisplatin(11 trials)**													
Huang, X. 2007	2	20	4	18	7.3%	0.39 [0.06, 2.44]	2007	?	?	+	+	+	?
Li, Y. 2009	3	30	8	30	13.8%	0.31 [0.07, 1.29]	2009	?	?	+	+	+	?
Ran, F. 2011	2	30	2	30	3.6%	1.00 [0.13, 7.60]	2011	?	?	+	-	-	-
Li, L. 2011	16	30	18	30	16.1%	0.76 [0.27, 2.12]	2011	?	?	-	-	-	-
Zhuang, H. 2012	3	24	5	22	8.8%	0.49 [0.10, 2.33]	2012	?	?	+	+	+	?
Han, Z. 2012	2	28	3	28	5.4%	0.64 [0.10, 4.17]	2012	?	?	+	+	+	?
Han, S. 2013	5	90	9	90	16.3%	0.53 [0.17, 1.65]	2013	?	?	+	+	+	?
Liu, D. 2015	5	46	4	42	7.2%	1.16 [0.29, 4.64]	2015	?	?	+	-	-	-
Wang, Y. 2019	4	45	4	45	7.0%	1.00 [0.23, 4.27]	2019	?	?	+	-	-	-
Peng,H.2020	1	25	1	25	1.8%	1.00 [0.06, 16.93]	2020	?	?	+	-	-	-
Feng, F.2023	6	34	8	34	12.7%	0.70 [0.21, 2.28]	2023	?	?	+	+	+	?
**Subtotal (95% CI)**		**402**		**394**	**100.0%**	**0.65 [0.42, 1.00]**							
Total events	49		66										
Heterogeneity: Chi² = 2.98, df = 10 (P = 0.98); I² = 0%													
Test for overall effect: Z = 1.95 (P = 0.05)													
**Kangai plus Cisplatin(Two trials)**													
Hu, J. 2008	2	36	3	35	31.0%	0.63 [0.10, 4.00]	2008	?	?	+	+	-	-
Qu, D. 2012	3	24	7	22	69.0%	0.31 [0.07, 1.38]	2012	?	?	+	+	-	-
**Subtotal (95% CI)**		**60**		**57**	**100.0%**	**0.41 [0.13, 1.29]**							
Total events	5		10										
Heterogeneity: Chi² = 0.35, df = 1 (P = 0.56); I² = 0%													
Test for overall effect: Z = 1.52 (P = 0.13)													
**Matrine plus Cisplatin(Four trials)**													
Du, C. 2009	1	40	7	36	25.2%	0.11 [0.01, 0.91]	2008	?	?	+	+	+	?
Li, L. 2009	6	30	13	30	36.4%	0.33 [0.10, 1.03]	2009	?	?	+	+	+	?
Wang, Y. 2010b	0	20	5	20	18.8%	0.07 [0.00, 1.34]	2010	?	?	+	-	-	-
Ji, H. 2011	2	30	6	30	19.6%	0.29 [0.05, 1.55]	2011	?	?	+	+	-	-
**Subtotal (95% CI)**		**120**		**116**	**100.0%**	**0.21 [0.10, 0.48]**							
Total events	9		31										
Heterogeneity: Chi² = 1.60, df = 3 (P = 0.66); I² = 0%													
Test for overall effect: Z = 3.71 (P = 0.0002)													

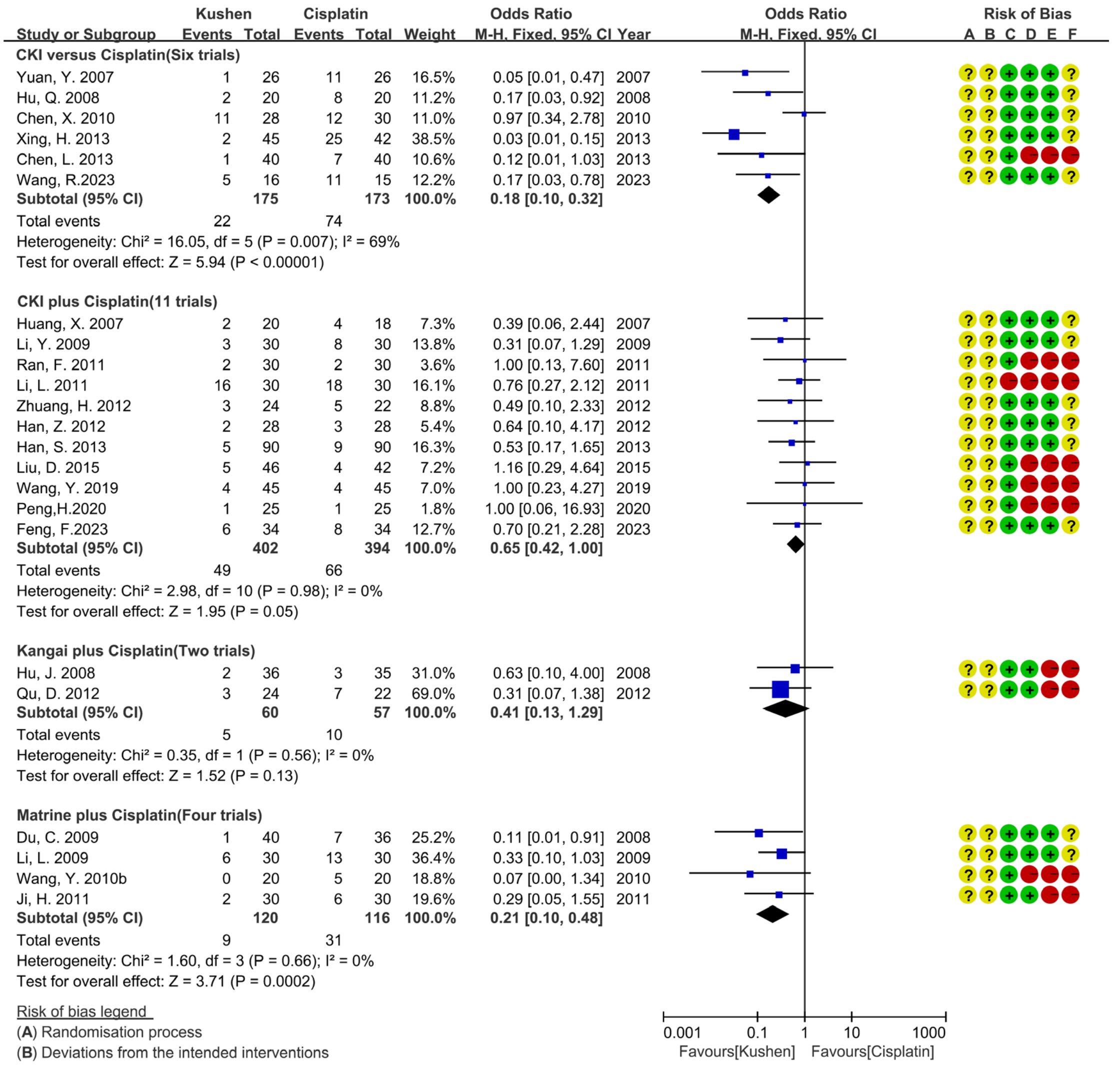

Risk of bias legend

(**A**) Randomisation process
(**B**) Deviations from the intended interventions
(**C**) Missing outcome data
(**D**) Measurement of the outcome
(**E**) Selection of the reported result
(**F**) Overall bias

Figure.S22 The analysis of thoracodynia between the two groups

Study or Subgroup	Kushen Events	Kushen Total	Cisplatin Events	Cisplatin Total	Weight	Odds Ratio M-H, Fixed, 95% CI	Year	A	B	C	D	E	F
**CKI versus Cisplatin(Four trials)**													
Yuan, Y. 2007	2	26	5	26	16.2%	0.35 [0.06, 2.00]	2007	?	?	+	+	+	?
Hu, Q. 2008	3	20	9	20	26.8%	0.22 [0.05, 0.98]	2008	?	?	+	+	+	?
Chen, X. 2010	18	28	0	30	0.6%	107.48 [5.94, 1944.00]	2010	?	?	+	+	+	?
Xing, H. 2013	4	45	8	42	26.4%	0.41 [0.11, 1.50]	2013	?	?	+	+	+	?
Chen, L. 2013	2	40	9	40	30.0%	0.18 [0.04, 0.90]	2013	?	?	+	-	-	-
**Subtotal (95% CI)**		**159**		**158**	**100.0%**	**0.94 [0.55, 1.59]**							
Total events	29		31										
Heterogeneity: Chi² = 20.75, df = 4 (P = 0.0004); I² = 81%													
Test for overall effect: Z = 0.24 (P = 0.81)													
**CKI and Cisplatin(15 trials)**													
Lin, S. 2007	2	33	4	33	8.3%	0.47 [0.08, 2.75]	2007	?	?	+	-	-	-
Huang, X. 2007	0	20	2	18	5.7%	0.16 [0.01, 3.59]	2007	?	?	+	+	+	?
Li, Y. 2009	0	30	3	30	7.6%	0.13 [0.01, 2.61]	2009	?	?	+	+	+	?
Ran, F. 2011	0	30	0	30		Not estimable	2011	?	?	+	-	-	-
Li, L. 2011	2	30	3	30	6.2%	0.64 [0.10, 4.15]	2011	?	?	-	-	-	-
Chen, F.2012	5	43	10	43	19.5%	0.43 [0.13, 1.40]	2012	?	?	+	+	+	?
Zhuang, H. 2012	2	24	5	22	10.6%	0.31 [0.05, 1.79]	2012	?	?	+	+	+	?
Zhu, M. 2013	0	28	3	28	7.6%	0.13 [0.01, 2.60]	2013	?	?	+	-	-	-
Guo, L. 2013	0	31	0	31		Not estimable	2013	?	?	+	+	+	?
Chen, Y. 2014	0	30	0	30		Not estimable	2014	?	?	+	+	+	?
Liu, D. 2015	5	46	4	42	8.2%	1.16 [0.29, 4.64]	2015	?	?	+	-	-	-
Qin,D.2016	3	32	6	32	12.0%	0.45 [0.10, 1.98]	2016	?	?	+	-	-	-
Wang, Y. 2019	4	45	4	45	8.0%	1.00 [0.23, 4.27]	2019	?	?	+	-	-	-
Peng,H.2020	1	25	2	25	4.2%	0.48 [0.04, 5.65]	2020	?	?	+	-	-	-
Feng, F.2023	1	34	1	34	2.1%	1.00 [0.06, 16.67]	2023	?	?	+	+	+	?
**Subtotal (95% CI)**		**481**		**473**	**100.0%**	**0.50 [0.30, 0.82]**							
Total events	25		47										
Heterogeneity: Chi² = 5.05, df = 11 (P = 0.93); I² = 0%													
Test for overall effect: Z = 2.76 (P = 0.006)													
**Kangai and Cisplatin(One trial)**													
Hu, J. 2008	2	36	2	35	100.0%	0.97 [0.13, 7.30]	2008	?	?	+	+	-	-
**Subtotal (95% CI)**		**36**		**35**	**100.0%**	**0.97 [0.13, 7.30]**							
Total events	2		2										
Heterogeneity: Not applicable													
Test for overall effect: Z = 0.03 (P = 0.98)													
**Matrine and Cisplatin(Four trials)**													
Du, C. 2009	1	40	3	36	22.1%	0.28 [0.03, 2.84]	2008	?	?	+	+	+	?
Li, L. 2009	4	30	5	30	31.1%	0.77 [0.19, 3.20]	2009	?	?	+	+	+	?
He,Y. 2010	0	47	0	36		Not estimable	2010	?	?	+	+	-	-
Ji, H. 2011	2	30	7	30	46.8%	0.23 [0.04, 1.24]	2011	?	?	+	+	-	-
**Subtotal (95% CI)**		**147**		**132**	**100.0%**	**0.41 [0.16, 1.07]**							
Total events	7		15										
Heterogeneity: Chi² = 1.28, df = 2 (P = 0.53); I² = 0%													
Test for overall effect: Z = 1.83 (P = 0.07)													

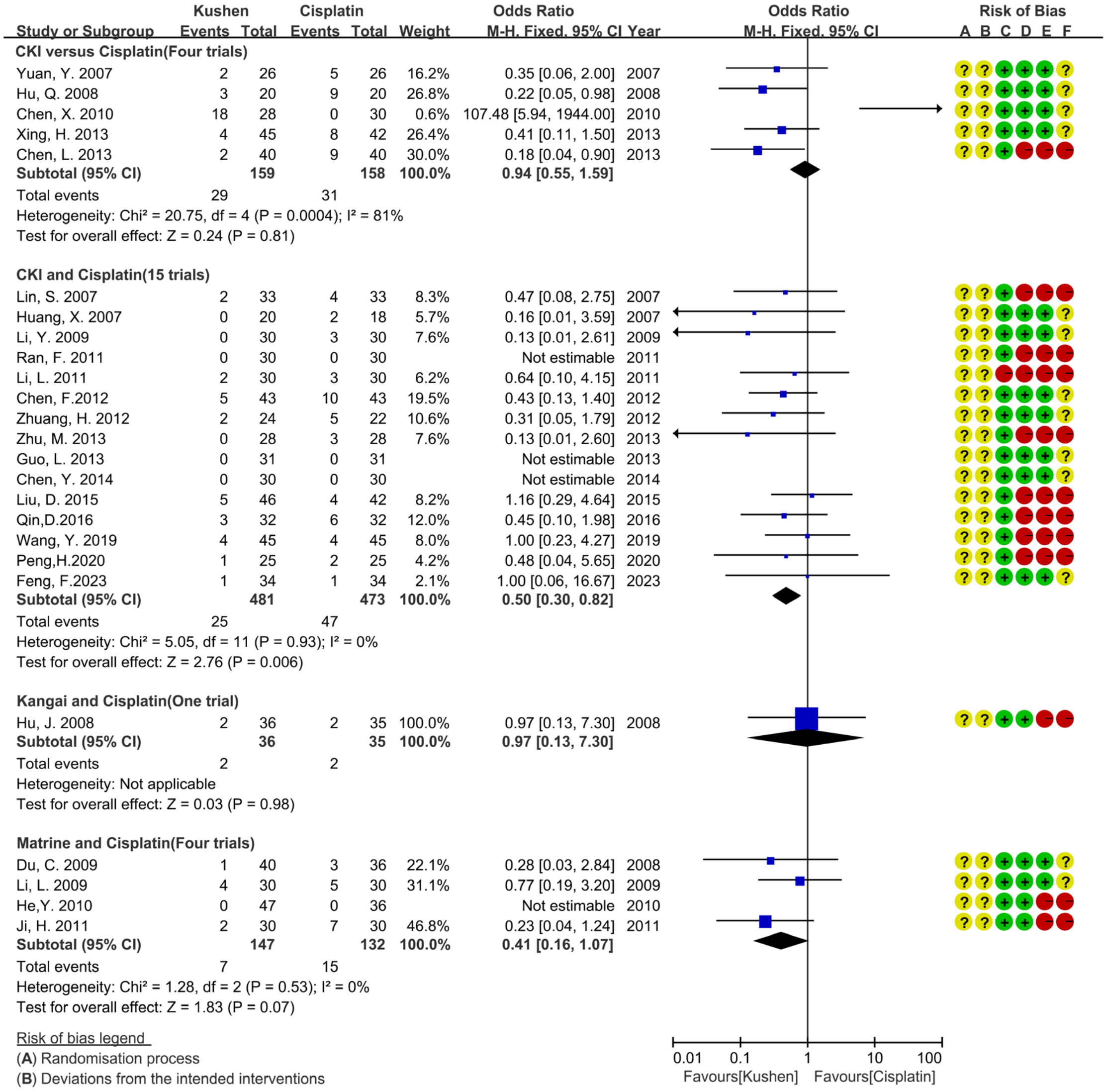

Risk of bias legend
(**A**) Randomisation process
(**B**) Deviations from the intended interventions
(**C**) Missing outcome data
(**D**) Measurement of the outcome
(**E**) Selection of the reported result
(**F**) Overall bias

Figure.S23 The analysis of fever between the two groups

Study or Subgroup	Kushen Events	Kushen Total	Cisplatin Events	Cisplatin Total	Weight	Odds Ratio M-H, Fixed, 95% CI	Year	A	B	C	D	E	F
**CKI and Cisplatin(Three trials)**													
Yang, G. 2012	0	39	0	39		Not estimable	2012	?	?	+	+	+	?
Liu, D. 2015	0	46	0	42		Not estimable	2015	?	?	+	−	−	−
Song, X. 2015	0	59	0	59		Not estimable	2015	?	?	+	−	−	−
**Subtotal (95% CI)**		**144**		**140**		**Not estimable**							
Total events	0		0										
Heterogeneity: Not applicable													
Test for overall effect: Not applicable													
**Kangai and Cisplatin(One trial)**													
Qu, D. 2012	0	24	0	22		Not estimable	2012	?	?	+	+	−	−
**Subtotal (95% CI)**		**24**		**22**		**Not estimable**							
Total events	0		0										
Heterogeneity: Not applicable													
Test for overall effect: Not applicable													
**Matrine and Cisplatin(One trial)**													
Wang, Y. 2010b	0	20	0	20		Not estimable	2010	?	?	+	−	−	−
**Subtotal (95% CI)**		**20**		**20**		**Not estimable**							
Total events	0		0										
Heterogeneity: Not applicable													
Test for overall effect: Not applicable													

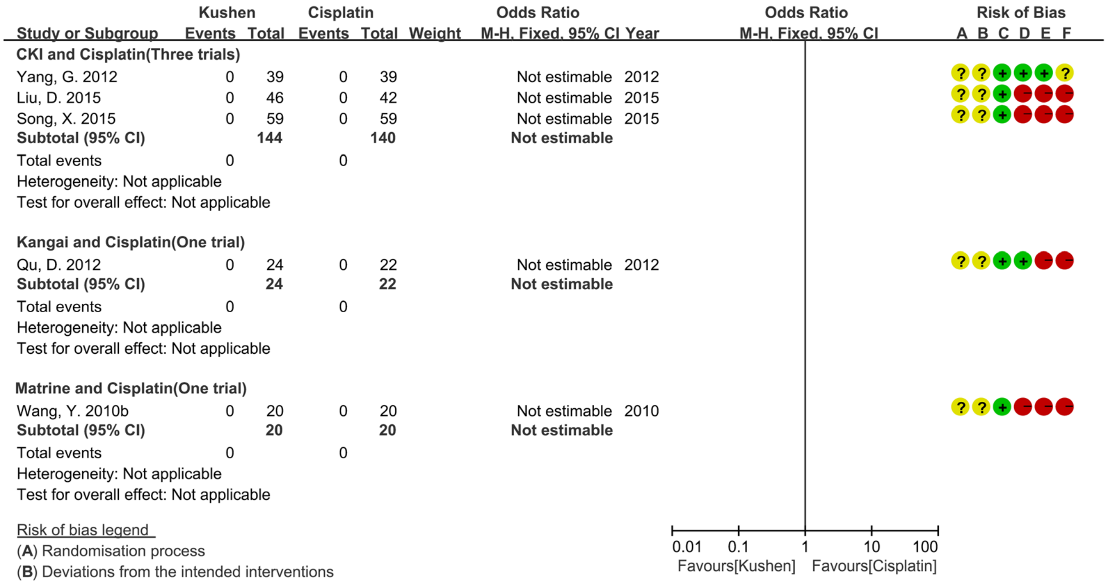

Risk of bias legend
(**A**) Randomisation process
(**B**) Deviations from the intended interventions
(**C**) Missing outcome data
(**D**) Measurement of the outcome
(**E**) Selection of the reported result
(**F**) Overall bias

Figure.S24 The TRAEs between the two groups
