# Supplementary material for: Intrapleural administration with traditional Chinese medicine injections (Sophorae flavescentis preparations) in controlling malignant pleural effusion: a clustered systematic review and meta-analysis
Source: Front Pharmacol. 2025 Apr 24;16:1519794. doi: 10.3389/fphar.2025.1519794 (PMC12058796; doi:10.3389/fphar.2025.1519794)
Supplement: Supplementary file 3 [file DataSheet6.pdf]

Supplementary materials.7 Publication bias analysis (Figures.S73 to S94)

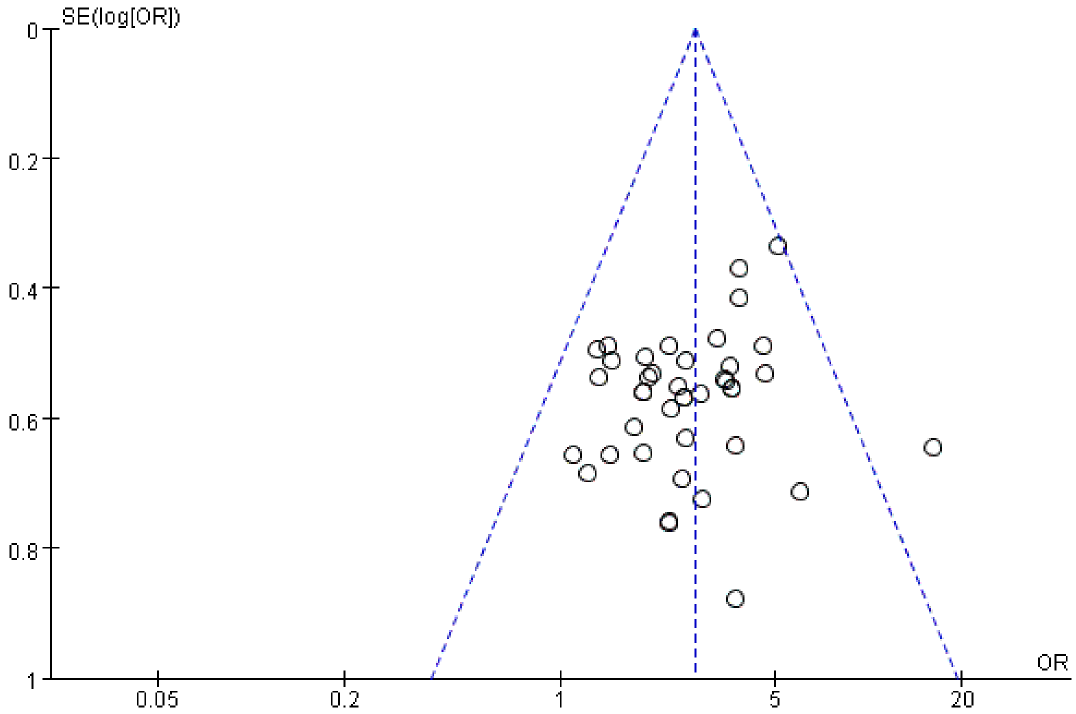

Figure.S73 The funnel plot of complete response in compound Kushen injection(CKI) plus cisplatin (DDP)

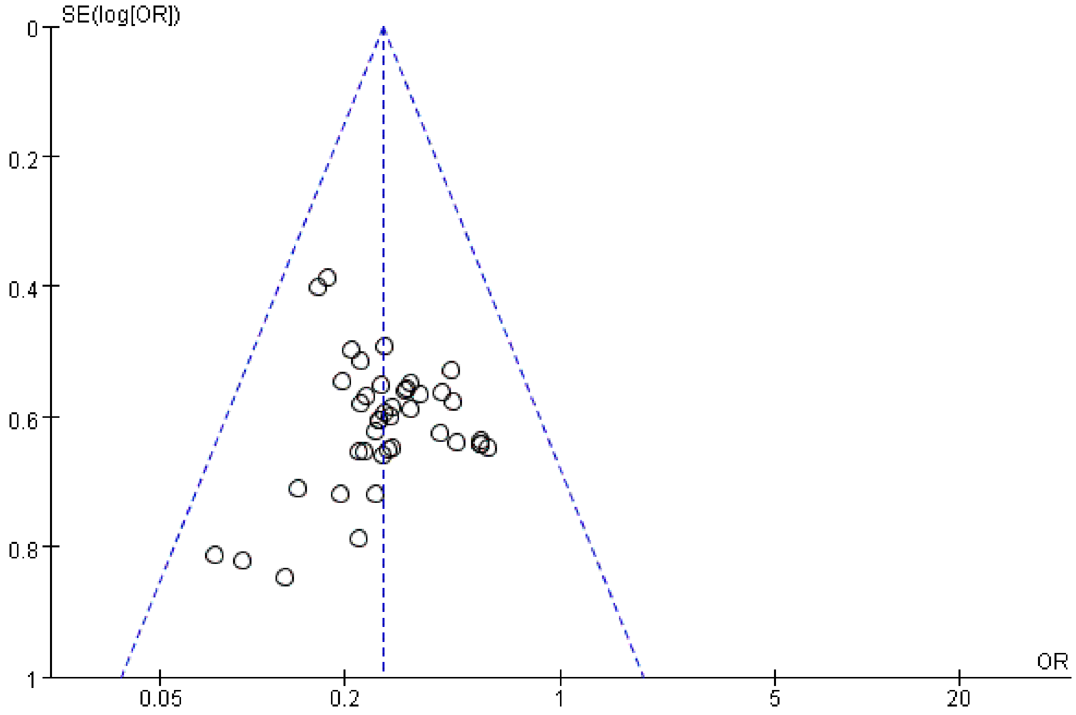

Figure.S74 The funnel plot of treatment failure in CKI plus cisplatin (DDP)

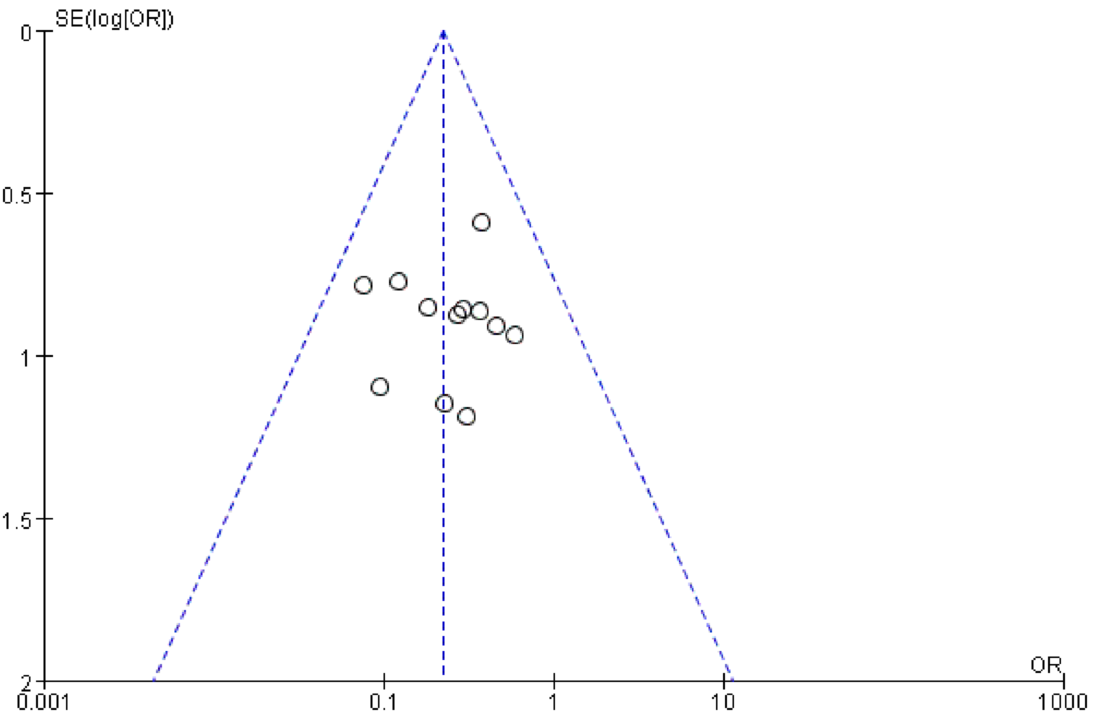

Figure.S75 The funnel plot of disease progression in CKI plus cisplatin (DDP)

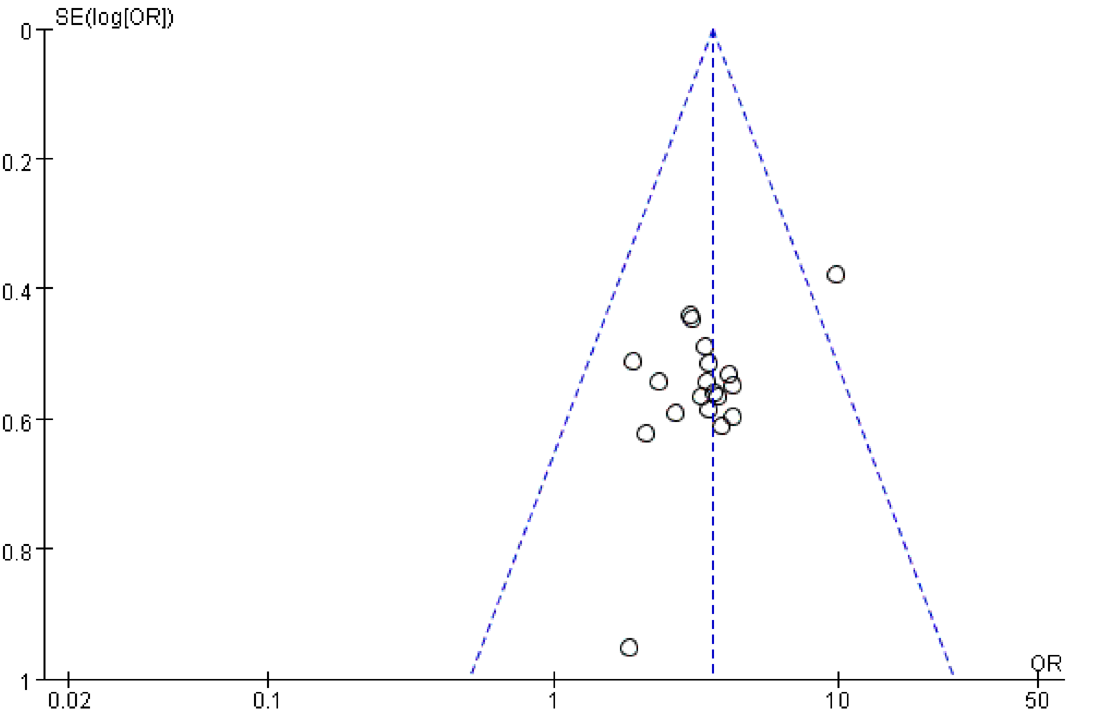

Figure.S76 The funnel plot of quality of life in CKI plus cisplatin (DDP)

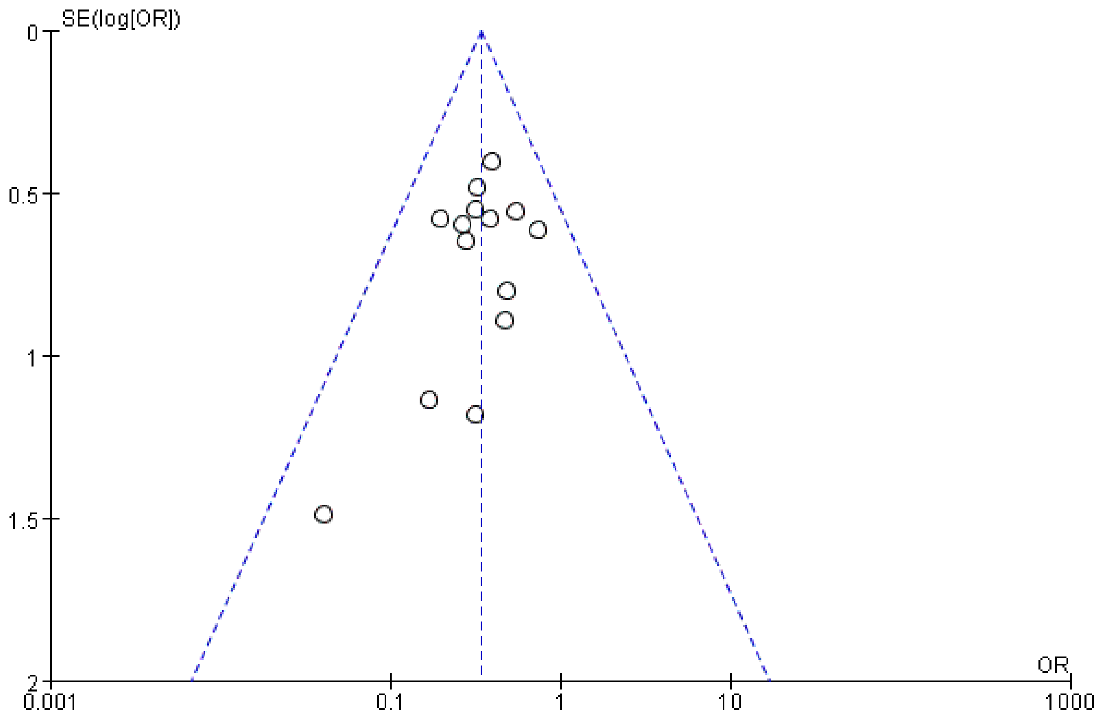

Figure.S77 The funnel plot of myelosuppression in CKI plus cisplatin (DDP)

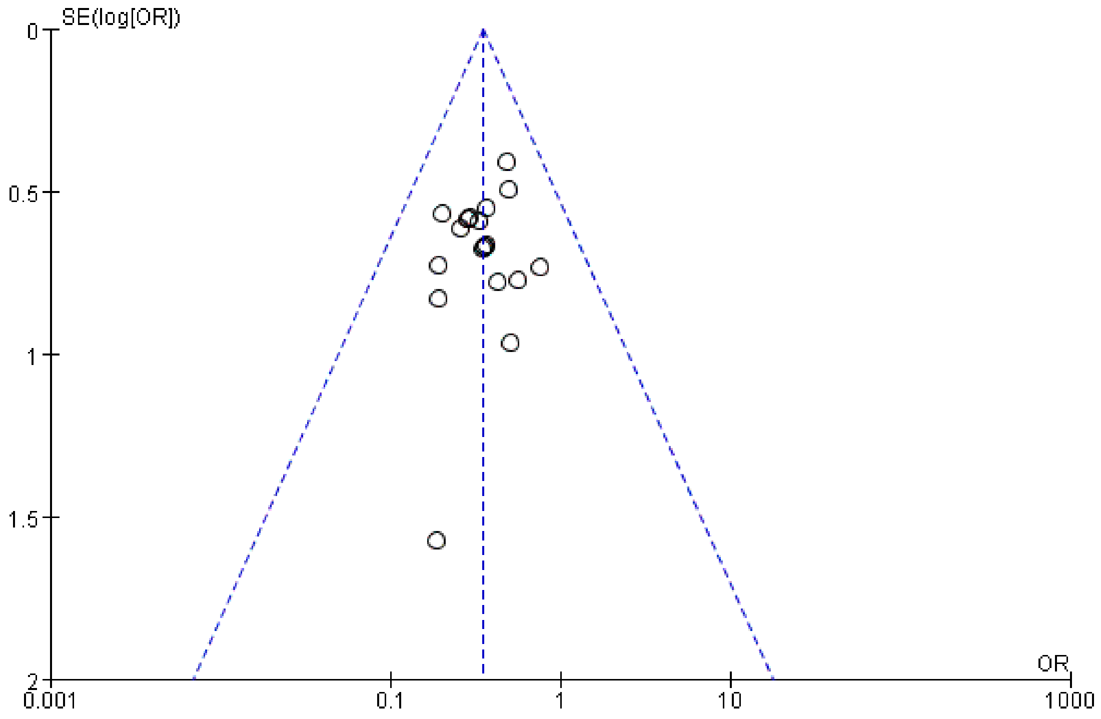

Figure.S78 The funnel plot of leukopenia in CKI plus cisplatin (DDP)

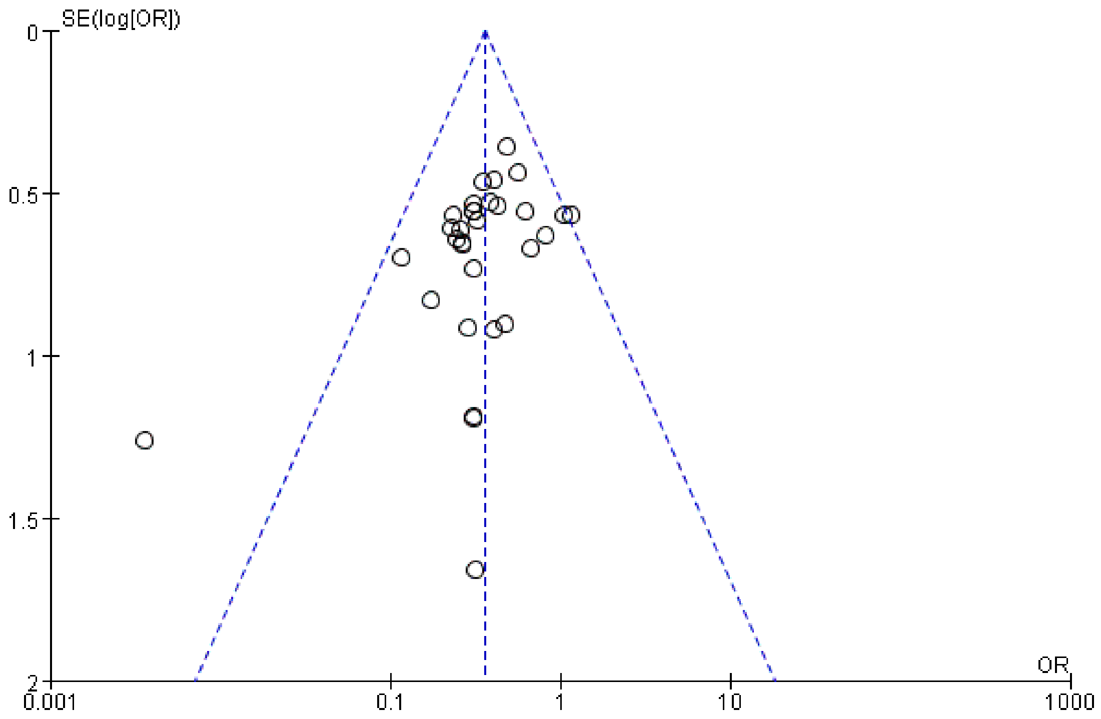

Figure.S79 The funnel plot of gastrointestinal reactions in CKI plus cisplatin (DDP)

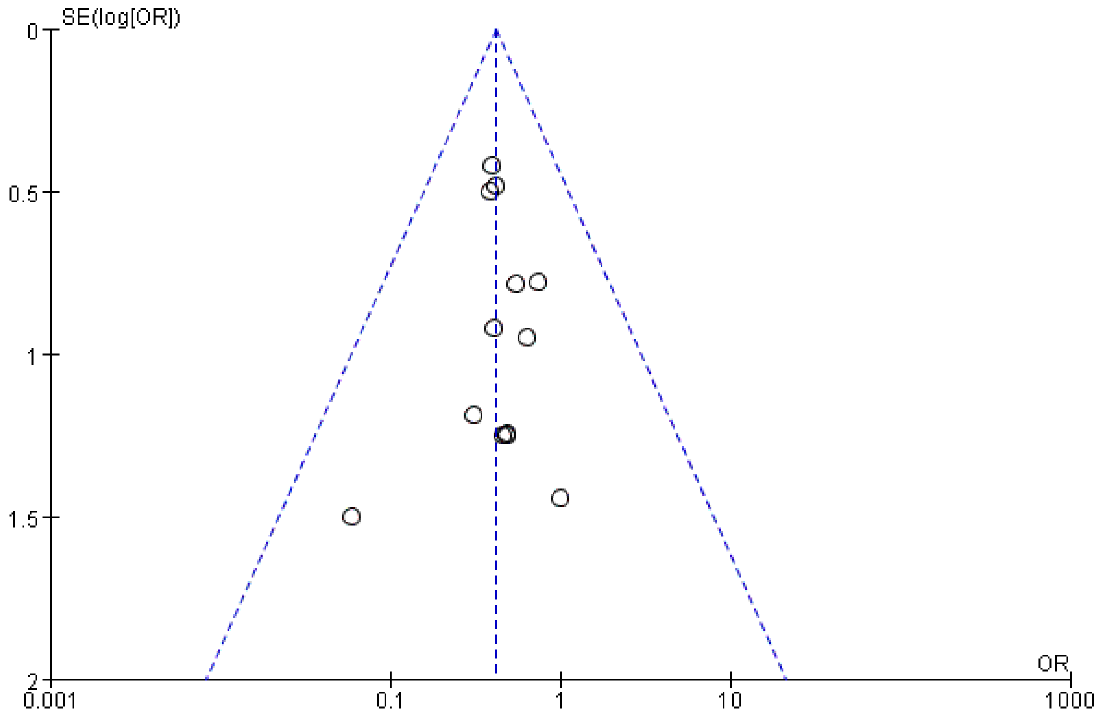

Figure.S80 The funnel plot of hepatic dysfunction in CKI plus cisplatin (DDP)

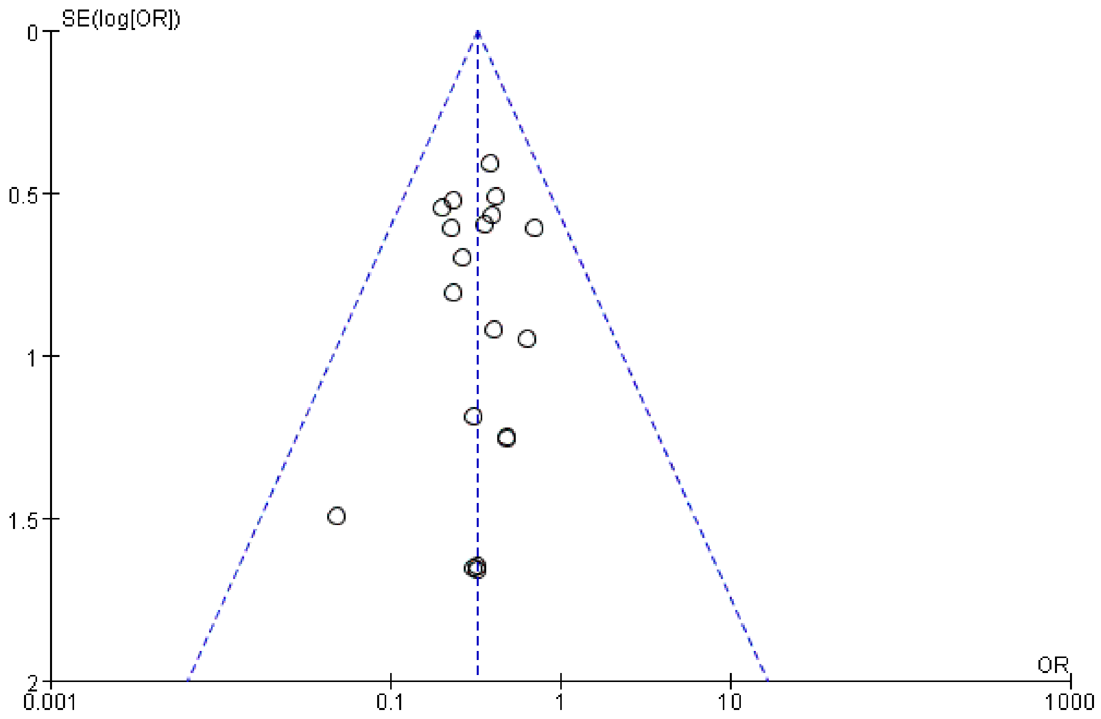

Figure.S81 The funnel plot of renal dysfunction in CKI plus cisplatin (DDP)

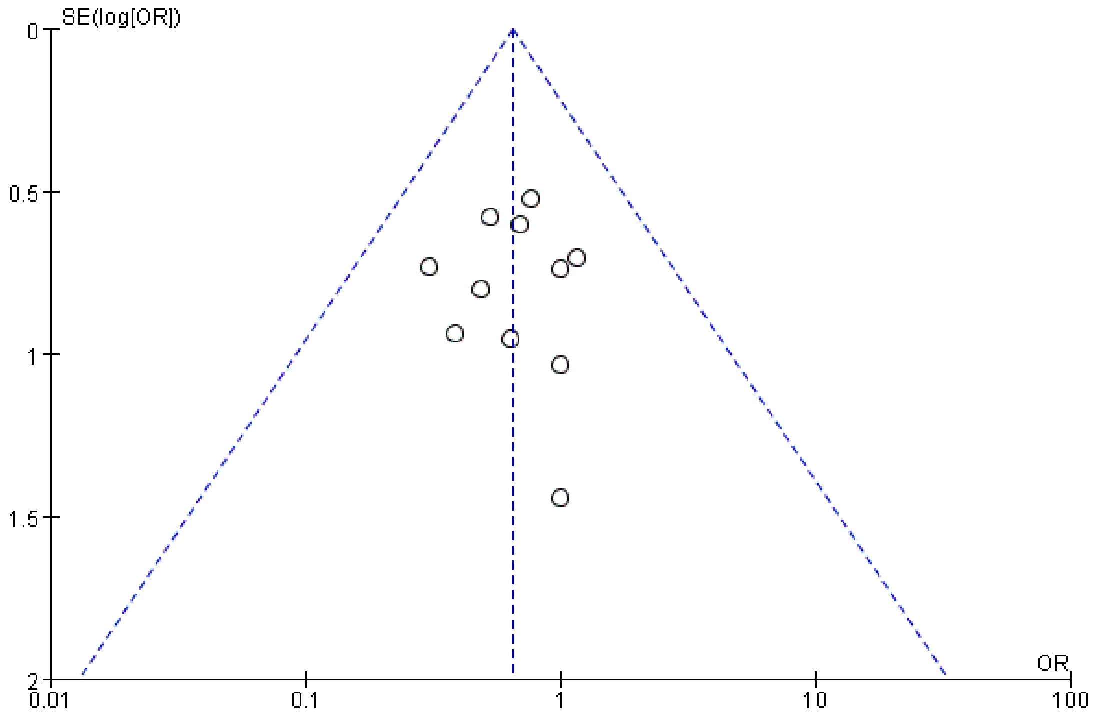

Figure.S82 The funnel plot of thoracodynia in CKI plus cisplatin (DDP)

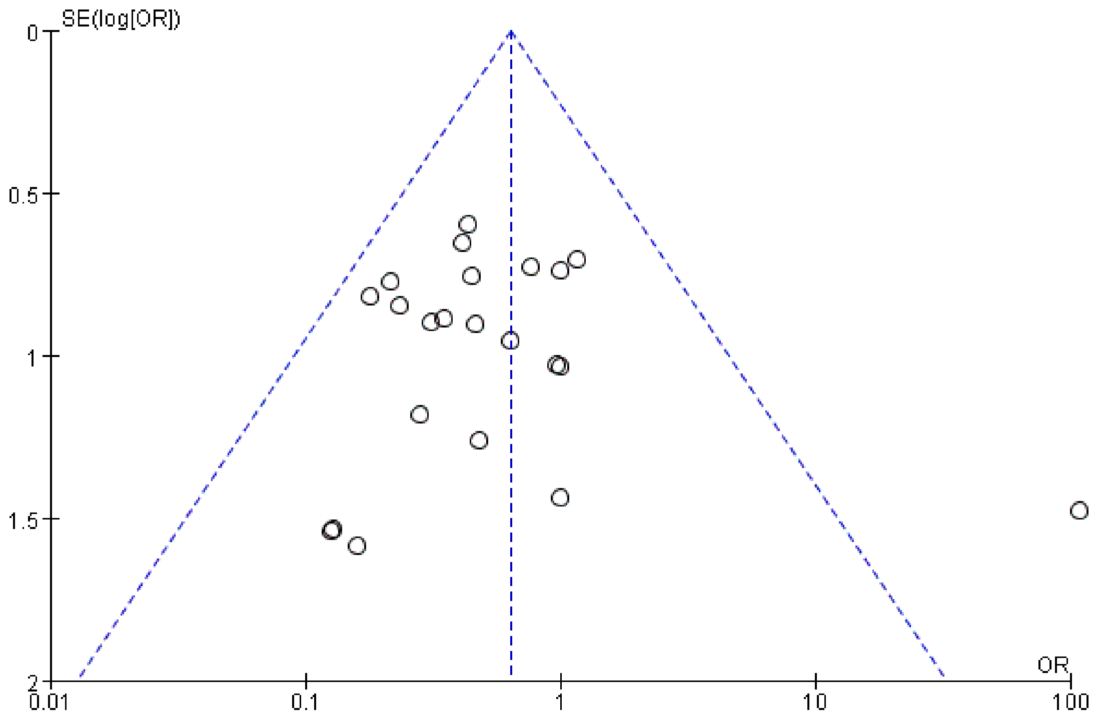

Figure.S83 The funnel plot of fever in CKI plus cisplatin (DDP)

Supplementary materials.8 Trial sequential analysis (Figures.S84 to S94)

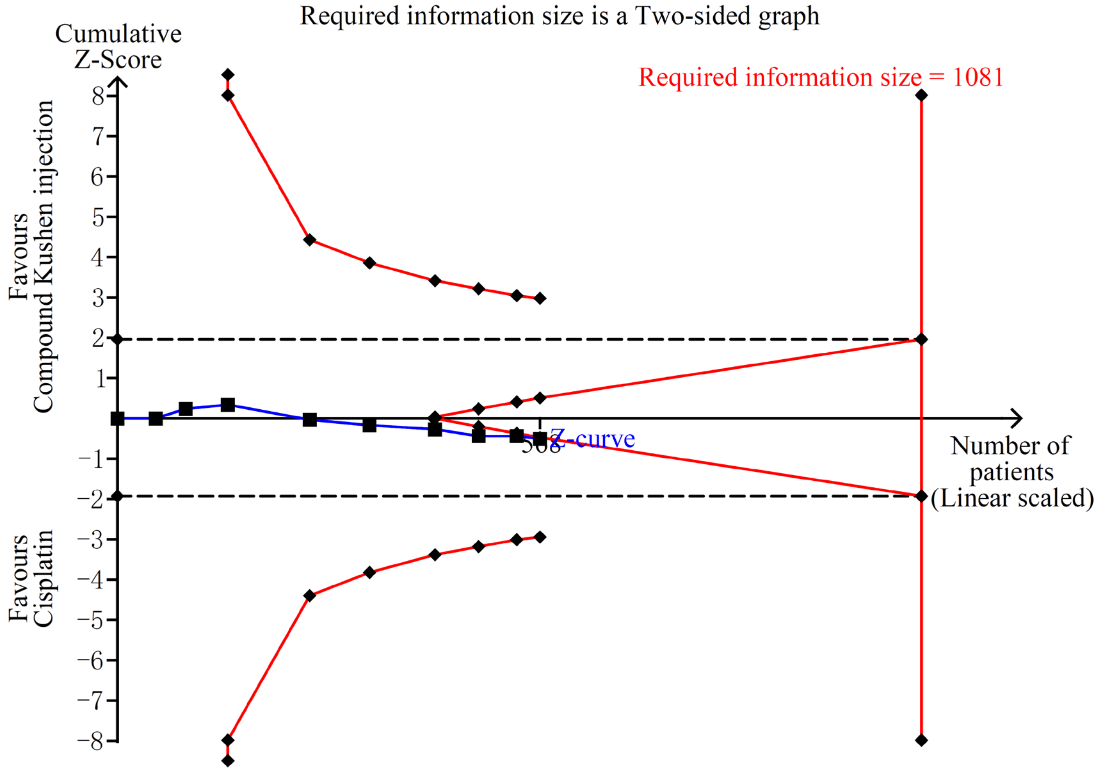

Figure.S84 The complete response in Compound Kushen injection versus Cisplatin

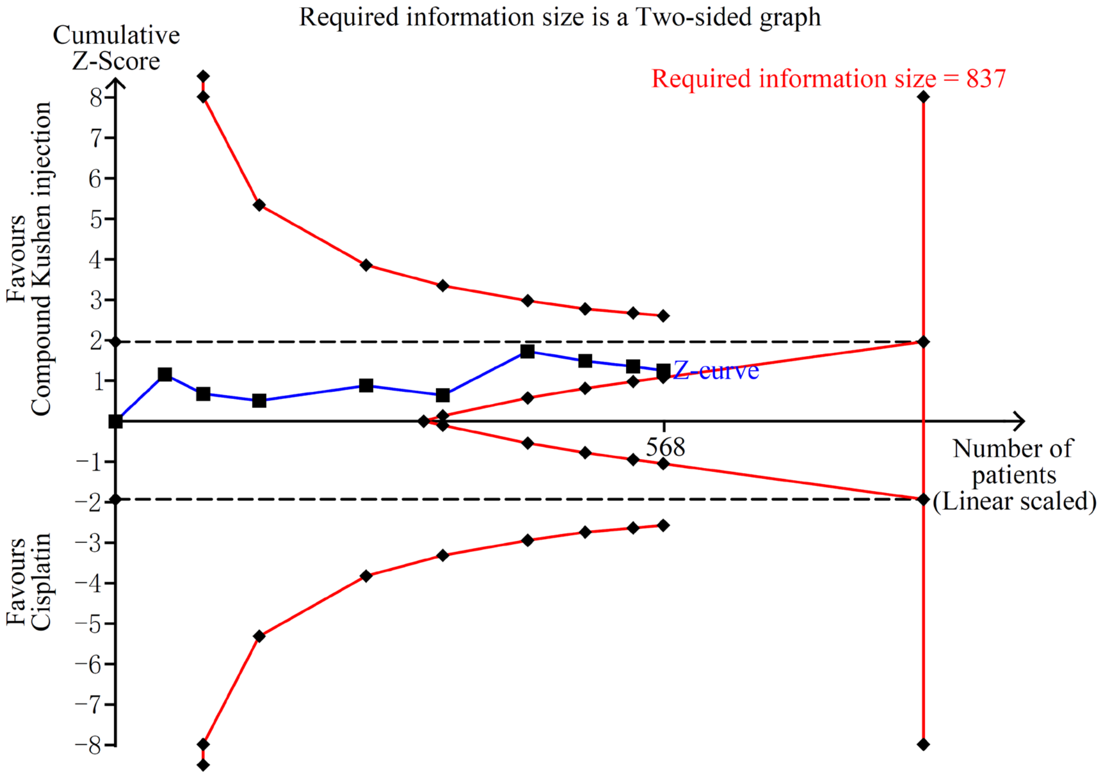

Figure.S85 The pleurodesis failure in Compound Kushen injection versus Cisplatin

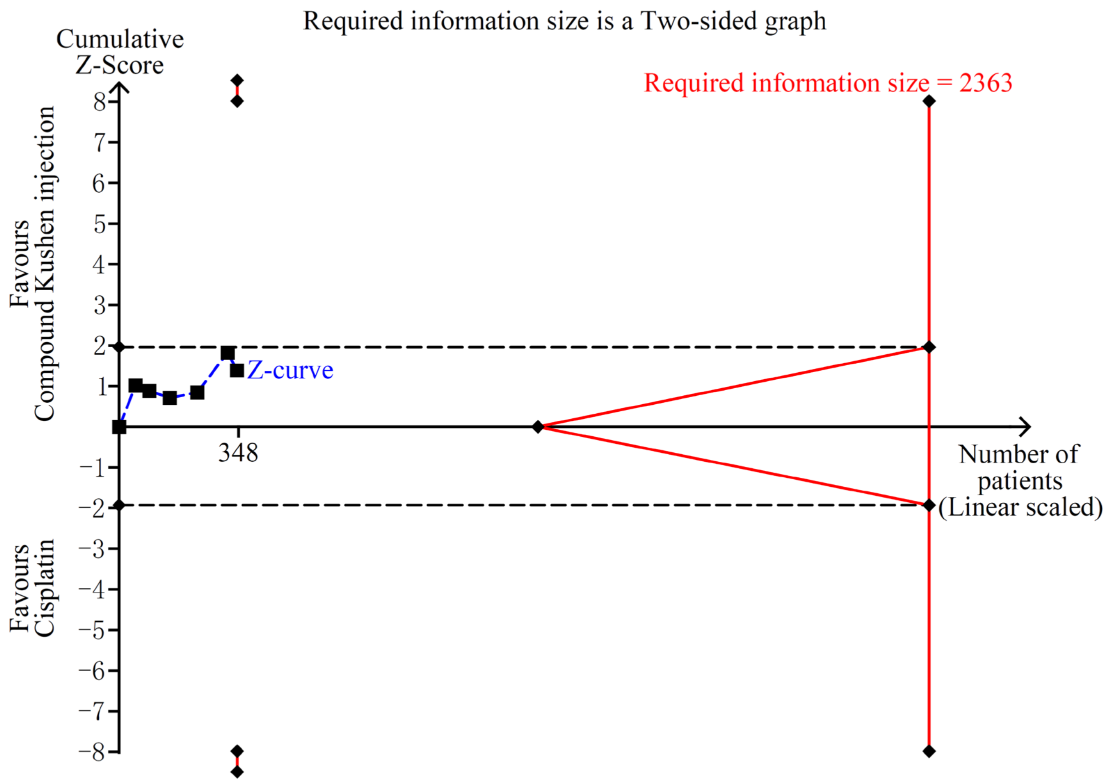

Figure.S86 The pleural progression in Compound Kushen injection versus Cisplatin

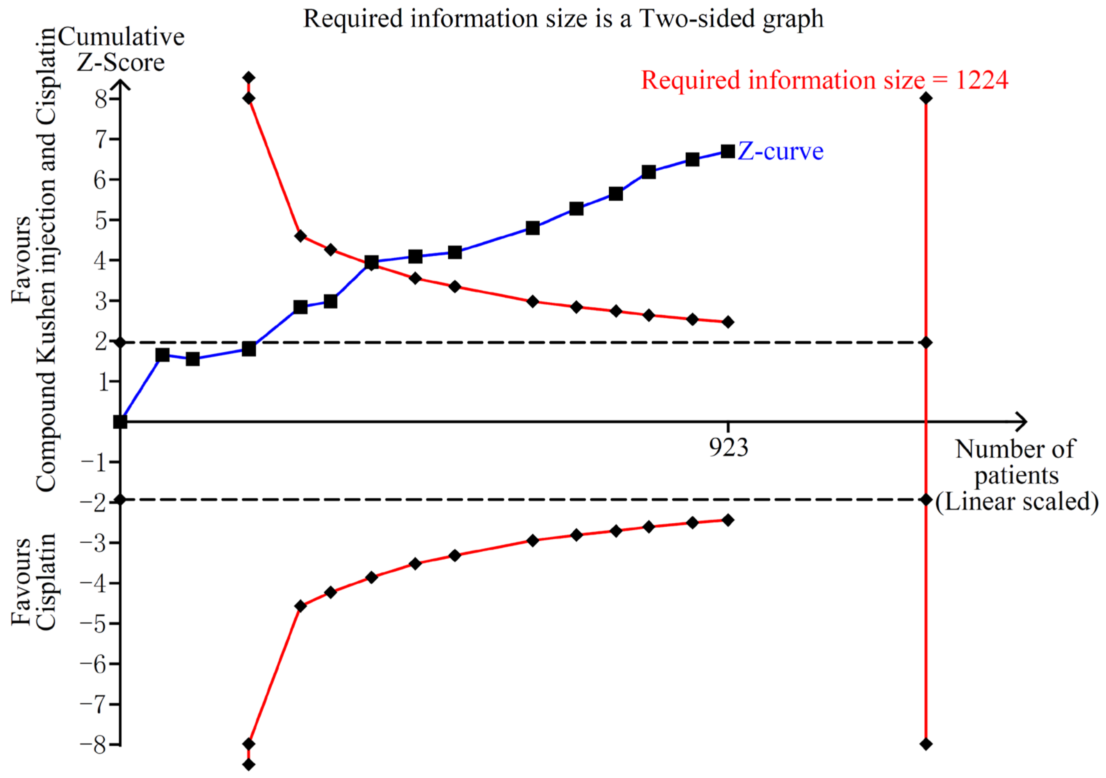

Figure.S87 The trial sequential analysis for myelosuppression in CKI and Cisplatin

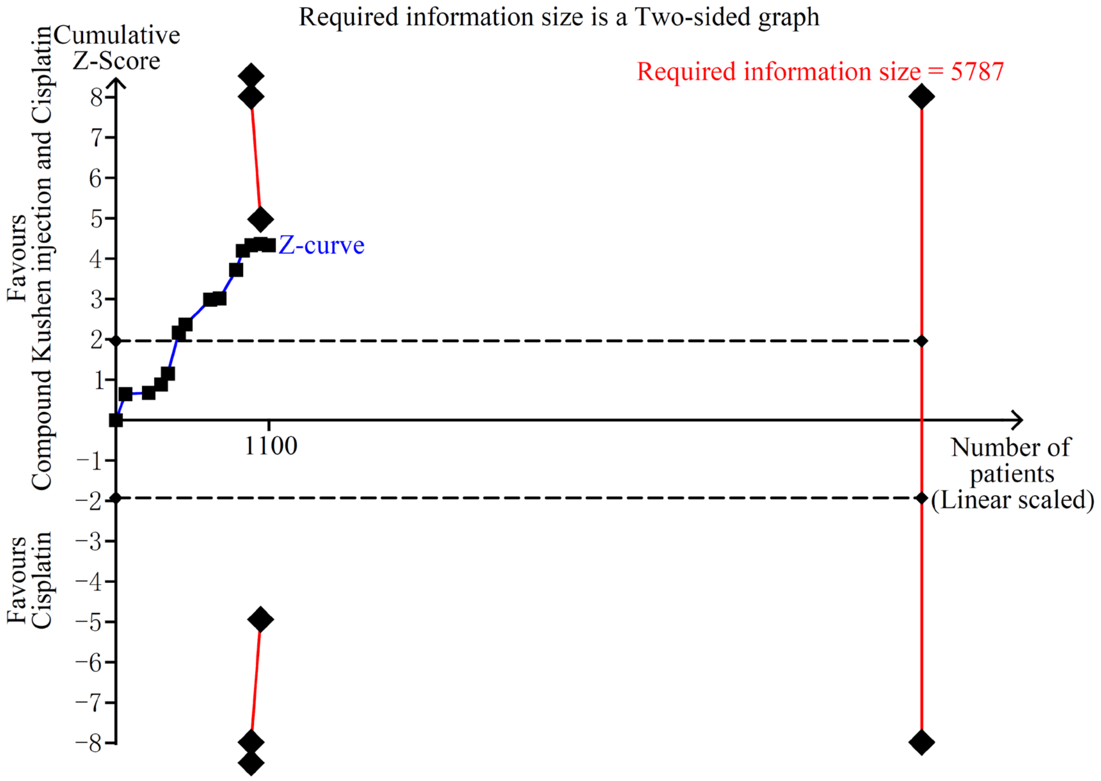

Figure.S88 The trial sequential analysis for hepatotoxicity in CKI and Cisplatin

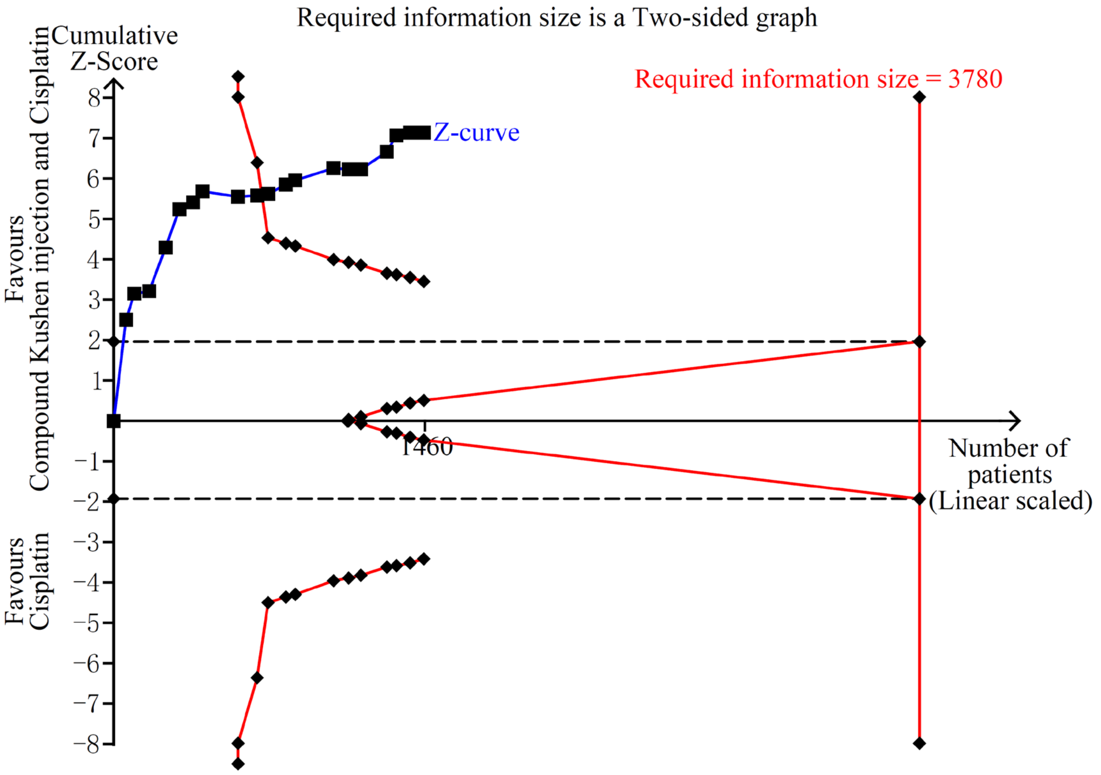

Figure.S89 The trial sequential analysis for nephrotoxicity in CKI and Cisplatin

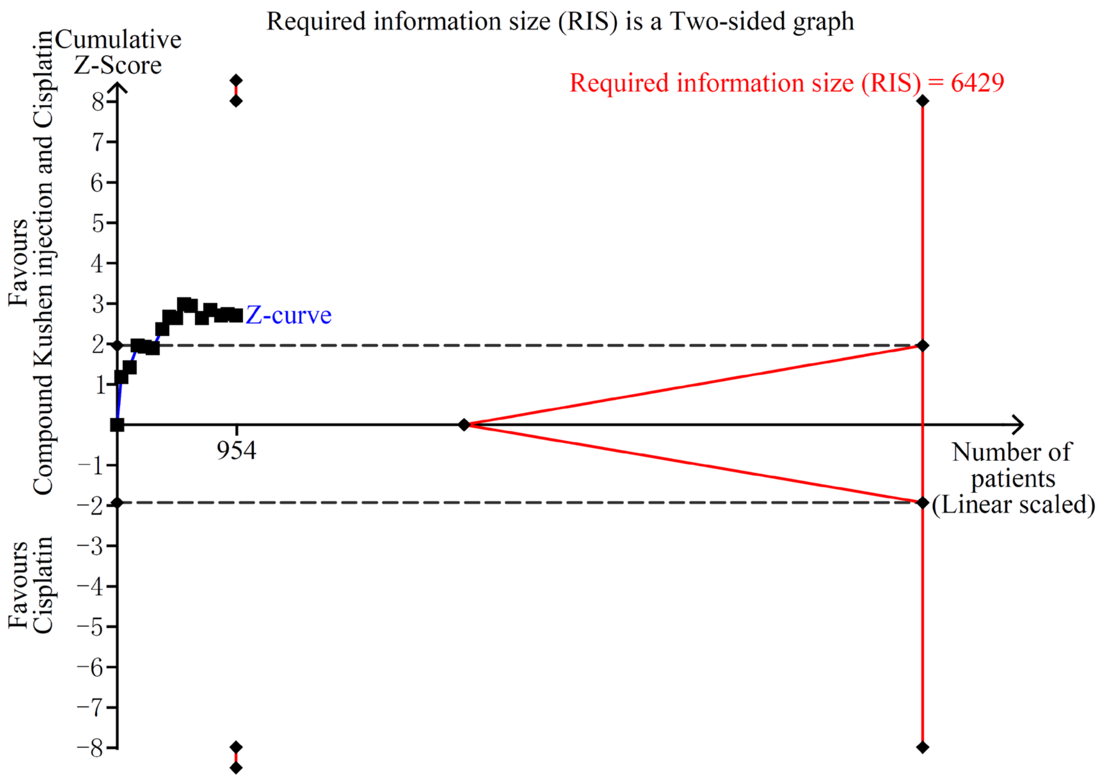

Figure.S90 The trial sequential analysis for fever in CKI and Cisplatin

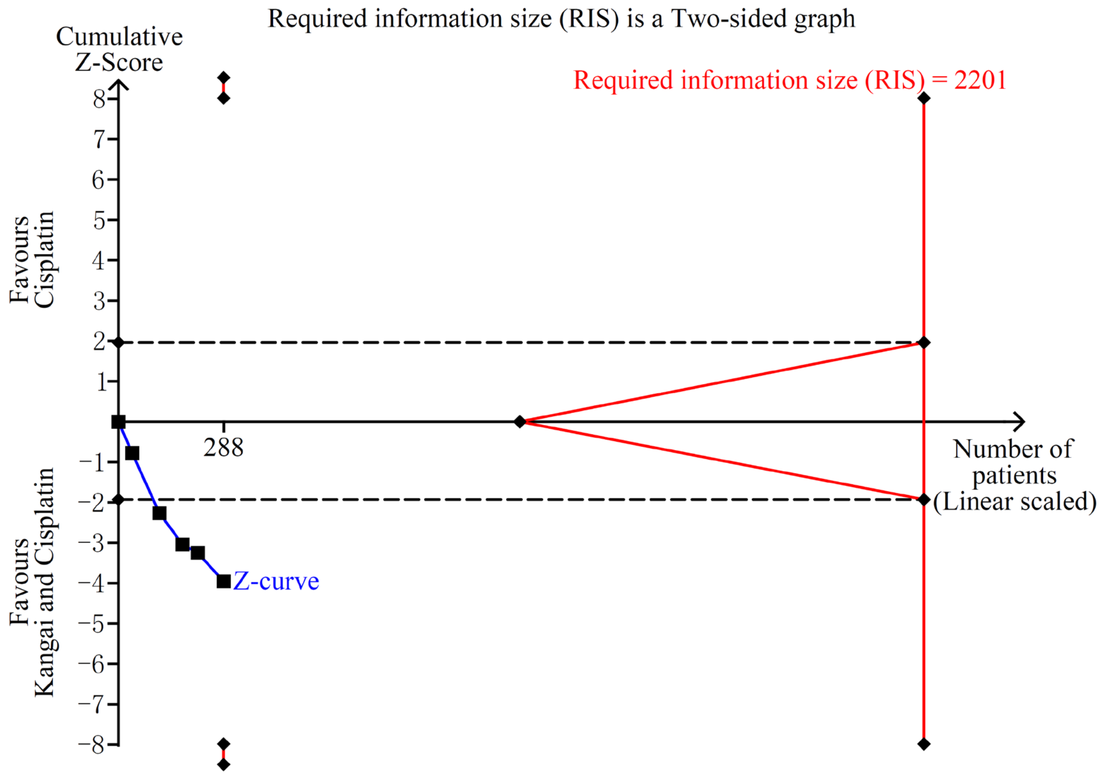

Figure.S91 The trial sequential analysis for complete response in Kangai and Cisplatin

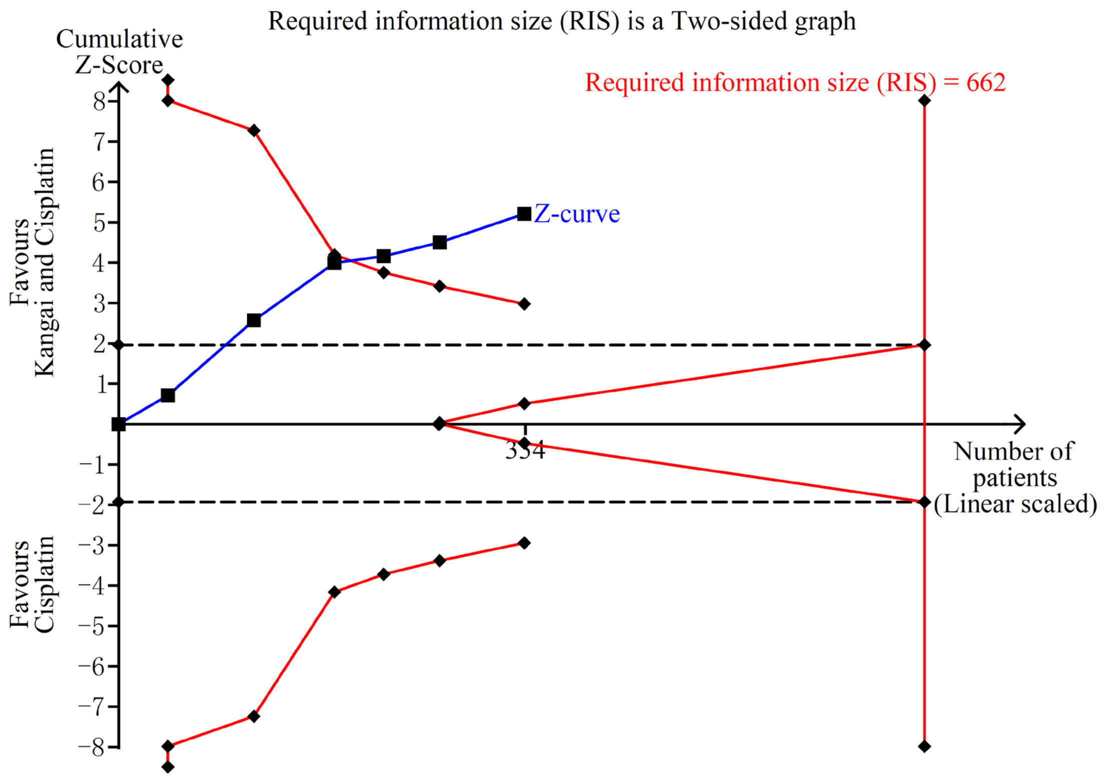

Figure.S92 The trial sequential analysis for pleurodesis failure in Kangai and Cisplatin

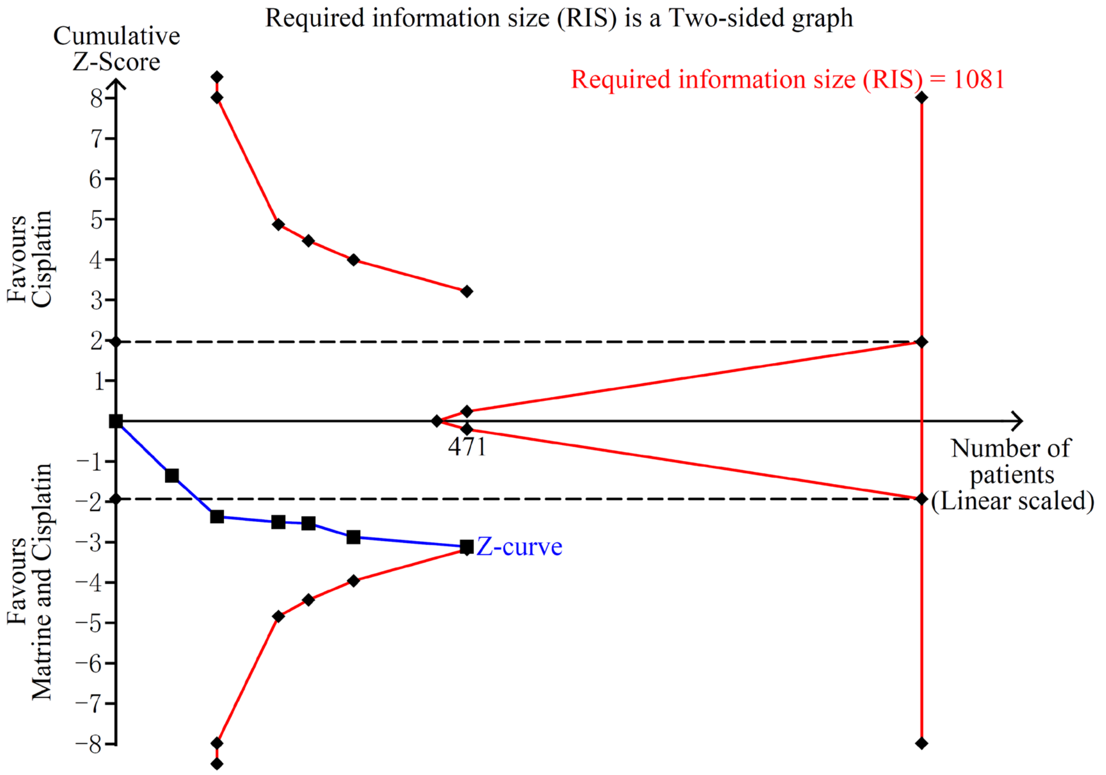

Figure.S93 The trial sequential analysis for complete response in Matrine and Cisplatin

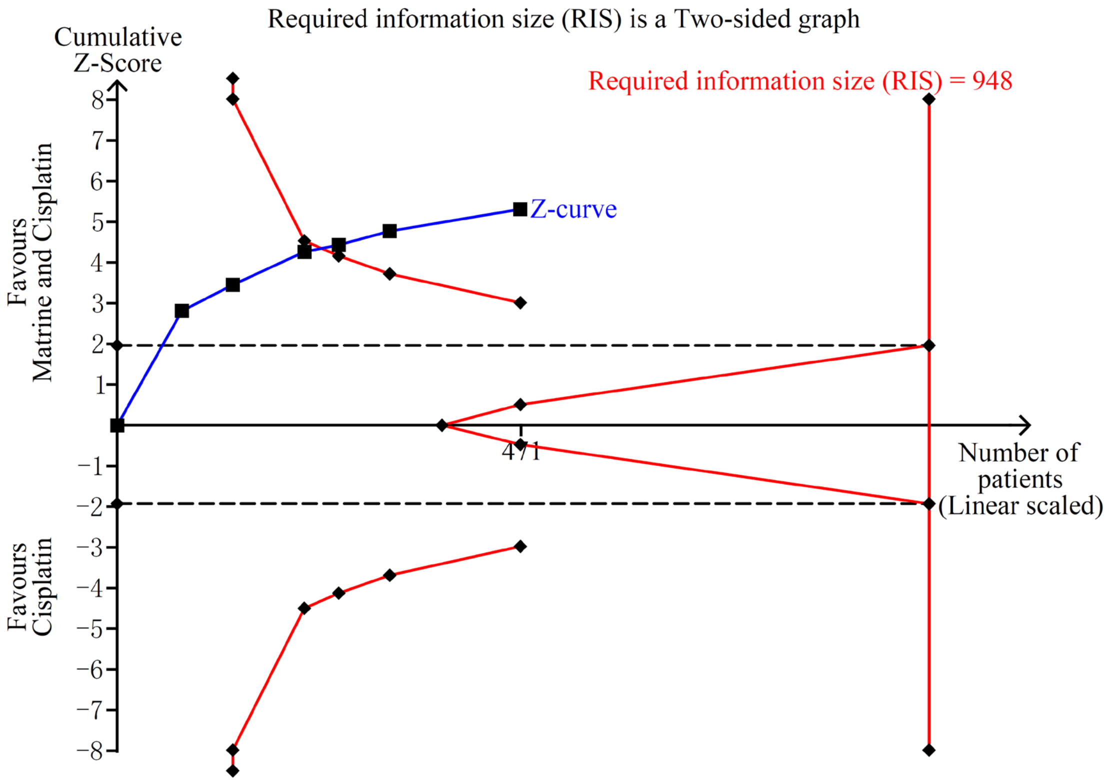

Figure.S94 The trial sequential analysis for pleurodesis failure in Matrine and Cisplatin
