# Supplementary material for: Intrapleural administration with traditional Chinese medicine injections (Sophorae flavescentis preparations) in controlling malignant pleural effusion: a clustered systematic review and meta-analysis
Source: Front Pharmacol. 2025 Apr 24;16:1519794. doi: 10.3389/fphar.2025.1519794 (PMC12058796; doi:10.3389/fphar.2025.1519794)
Supplement: Supplementary file 4 [file DataSheet3.pdf]

Supplementary materials.4 The risk-of-bias (Figure.S1 to S5)

A.Clinical response

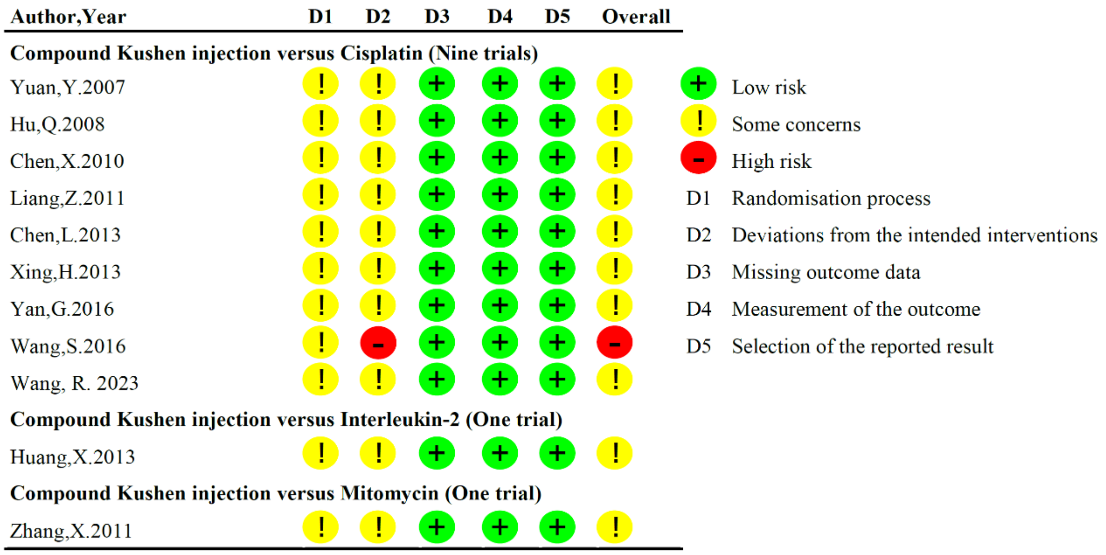

Figure.S1a The risk-of-bias in Compound Kushen injection (CKI) alone

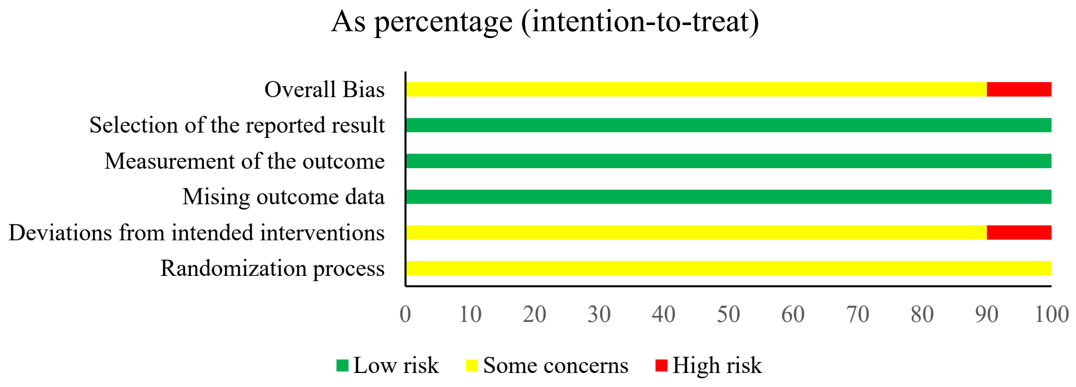

Figure.S1b. The risk-of-bias in CKI versus Cisplatin (DDP)

Author year	D1	D2	D3	D4	D5	Overall
**Compound Kushen injection (CKI) plus Nedaplatin(Three trials)**						
Li,S.2014	!	!	+	+	+	!
Zhang,S.2015	!	!	+	+	+	!
Li,R.2017	!	!	+	+	+	!
**CKI plus Carboplatin(One trial)**						
He,R.2010	!	!	+	+	+	!
**CKI plus Lobaplatin(Two trials)**						
Liu,X.2016	!	!	+	+	+	!
Huang,L.2021	!	!	+	+	+	!
**CKI plus Bleomycin(Three trials)**						
Chen,M.2003	!	!	+	+	+	!
Liu,Y.2011	!	!	+	+	+	!
Sun,Y.2012	!	!	+	+	+	!
**CKI plus Hydroxycamptothecin(Three trials)**						
He,P.2009	!	!	+	+	+	!
Wu,Z.2014	!	!	+	+	-	-
Cai,H.2019	!	!	+	+	+	!
**CKI plus Interleukin-2 (Two trials)**						
Zhou,Y.2010	!	!	+	+	+	!
Hao,J.2007	!	!	+	+	+	!
**CKI plus OK-432 (Two trials)**						
Wei,W.2014	!	!	+	+	+	!
Zhong,B.2015	!	!	+	+	+	!
**CKI plus Mitomycin (One trial)**						
Zhang,X.2013	!	!	+	+	+	!
**CKI plus Corynebacterium Parvum (One trial)**						
Huang,Z.2012	!	!	+	+	+	!

+ Low risk
! Some concerns
- High risk

D1 Randomisation process
D2 Deviations from the intended interventions
D3 Missing outcome data
D4 Measurement of the outcome
D5 Selection of the reported result

Figure.S2a. The risk-of-bias in CKI plus other sclerosants

Author year	D1	D2	D3	D4	D5	Overall
**Kangai plus Cisplatin(Six trials)**						
Zhang,X.2006	!	!	+	+	+	!
Hu, J.2008	!	!	+	+	+	!
Xu,M.2008	!	!	+	+	+	!
He,J.2011	!	!	+	+	+	!
Qu,D.2012	!	!	+	+	-	-
Wang,H.2016	!	!	+	+	+	!
**Kangai plus Carboplatin(one trial)**						
Chen,Y.2009	!	!	+	+	+	!

+ Low risk
! Some concerns
- High risk
D1 Randomisation process
D2 Deviations from the intended interventions
D3 Missing outcome data
D4 Measurement of the outcome
D5 Selection of the reported result

Figure.S2b. The risk-of-bias in Kangai injection (Kangai) plus Sclerosants

Author year	D1	D2	D3	D4	D5	Overall
**Matrine plus Cisplatin(Six trials)**						
Du,C.2009	!	!	+	+	+	!
Li,L.2009	!	!	+	+	+	!
He,Y.2010	!	!	+	+	+	!
Wang,Y.2010b	!	!	+	+	+	!
Ji,H.2011	!	!	+	+	+	!
Ji,F.2012	!	!	+	+	+	!
**Matrine plus Carboplatin(One trial)**						
Cui,A.2008	!	!	+	+	+	!

+ Low risk
! Some concerns
- High risk
D1 Randomisation process
D2 Deviations from the intended interventions
D3 Missing outcome data
D4 Measurement of the outcome
D5 Selection of the reported result

Figure.S2c. The risk-of-bias in Matrine plus Sclerosants

B. Quality of life

Author year	D1	D2	D3	D4	D5	Overall
Yuan,Y.2007	!	!	+	-	+	-
Hu,Q.2008	!	!	+	-	+	-
Liang,Z.2011	!	!	+	-	+	-
Chen,L.2013	!	!	+	-	+	-
Xing,H.2013	!	!	+	-	+	-
Yan,G.2016	!	!	+	-	+	-

+ Low risk
! Some concerns
- High risk
D1 Randomisation process
D2 Deviations from the intended interventions
D3 Missing outcome data
D4 Measurement of the outcome
D5 Selection of the reported result

Figure.S3a. The risk-of-bias in Compound Kushen injection (CKI) versus Cisplatin

Author year	D1	D2	D3	D4	D5	Overall
Kangai plus Cisplatin(Two trials)						
Xu,M.2008	!	!	+	-	+	-
Qu,D.2012	!	!	+	-	+	-
Matrine plus Cisplatin(Two trials)						
Wang,Y.2010b	!	!	+	-	+	-
Ji,H.2011	!	!	+	-	+	-

+ Low risk
! Some concerns
- High risk
D1 Randomisation process
D2 Deviations from the intended intervention
D3 Missing outcome data
D4 Measurement of the outcome
D5 Selection of the reported result

Figure.S3b. The risk-of-bias in Kangai or Matrine plus Cisplatin

C. Overall survival

Author year	D1	D2	D3	D4	D5	Overall
**CKI vs Cisplatin(one trial)**						
Chen,X.2010	!	!	+	+	+	!
**CKI plus Cisplatin(Two trials)**						
Chen,Y.2011	!	!	+	+	+	!
Han,S.2013	!	!	+	+	+	!
**CKI plus Nedaplatin(One trial)**						
Zhang,S.2015	!	!	+	+	+	!
**Matrine plus Carboplatin(One trial)**						
Cui,A.2008	!	!	+	+	+	!
**Kangai plus Cisplatin(One trial)**						
He,J.2011	!	!	+	+	+	!

+ Low risk
! Some concerns
- High risk
D1 Randomisation process
D2 Deviations from the intended interventions
D3 Missing outcome data
D4 Measurement of the outcome
D5 Selection of the reported result

Figure.S4. The risk-of-bias in Kushen plus Sclerosants

D. Adverse events

Author year	D1	D2	D3	D4	D5	Overall
Yuan,Y.2007	!	!	+	+	+	!
Hu,Q.2008	!	!	+	+	+	!
Chen,X.2010	!	!	+	+	+	!
Liang,Z.2011	!	!	+	+	+	!
Chen,L.2013	!	!	+	+	-	-
Xing,H.2013	!	!	+	+	+	!
Yan,G.2016	!	!	+	-	-	-
Wang,S.2016	!	-	+	-	-	-
Wang, R. 2023	!	!	+	+	+	!

+ Low risk
! Some concerns
- High risk
D1 Randomisation process
D2 Deviations from the intended interventions
D3 Missing outcome data
D4 Measurement of the outcome
D5 Selection of the reported result

Figure.S5a. The risk-of-bias in CKI versus Cisplatin

Author year	D1	D2	D3	D4	D5	Overall
**Kangai plus Cisplatin(Five trials)**						
Zhang,X.2006	!	!	+	+	-	-
Hu, J.2008	!	!	+	+	-	-
Xu,M.2008	!	!	+	-	-	-
He,J.2011	!	!	+	+	-	-
Qu,D.2012	!	!	+	+	-	-
**Matrine plus Cisplatin(Five trials)**						
Du,C.2009	!	!	+	+	+	!
Li,L.2009	!	!	+	+	+	!
He,Y.2010	!	!	+	+	-	-
Wang,Y.2010b	!	!	+	-	-	-
Ji,H.2011	!	!	+	+	-	-

+ Low risk
! Some concerns
- High risk
D1 Randomisation process
D2 Deviations from the intended interventions
D3 Missing outcome data
D4 Measurement of the outcome
D5 Selection of the reported result

Figure.S5b. The risk-of-bias in Kangai or Matrine plus Cisplatin
