# Supplementary material for: Intrapleural administration with traditional Chinese medicine injections (Sophorae flavescentis preparations) in controlling malignant pleural effusion: a clustered systematic review and meta-analysis
Source: Front Pharmacol. 2025 Apr 24;16:1519794. doi: 10.3389/fphar.2025.1519794 (PMC12058796; doi:10.3389/fphar.2025.1519794)
Supplement: Supplementary file 5 [file DataSheet1.pdf]

Supplementary materials.1 The composition of Kushen preparitions

Radix Sophorae flavescentis (Chinese name: Kushen) contains various active components such as alkaloids, flavonoids, alkylxanthones, quinones, triterpene glycosides, fatty acids, and essential oils, notably the alkaloids matrine, oxymatrine, and sophoridine [1-3].
Compound Kushen injection mainly contains matrine, oxymatrine and sophoridine [4-6], and Kang-ai injection contains active ingredients such as Astragalus *polysaccharides, astragalosides, ginsenosides, ginseng polysaccharides and oxymatrine* [7, 8].

1.Matrine

PubChem CID:91466

Formula:C15H24N2O

Molecular Weight:248.37 g/mol

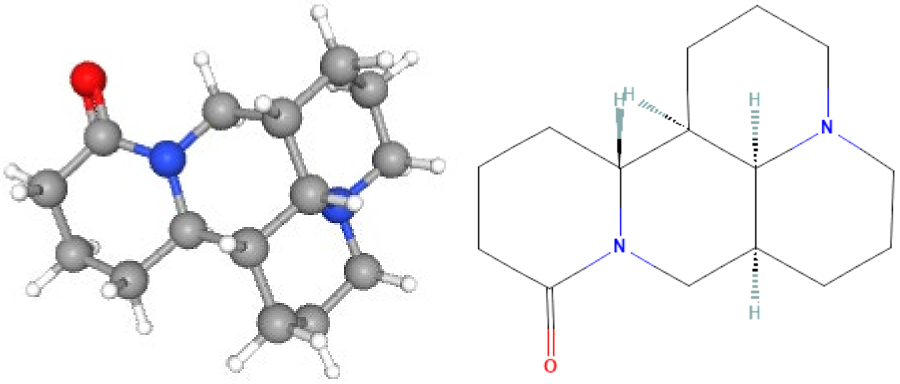

2. Oxymatrine

PubChem CID:114850

Formula: C15H24N2O2·H2O

Molecular Weight: 264.36 g/mol

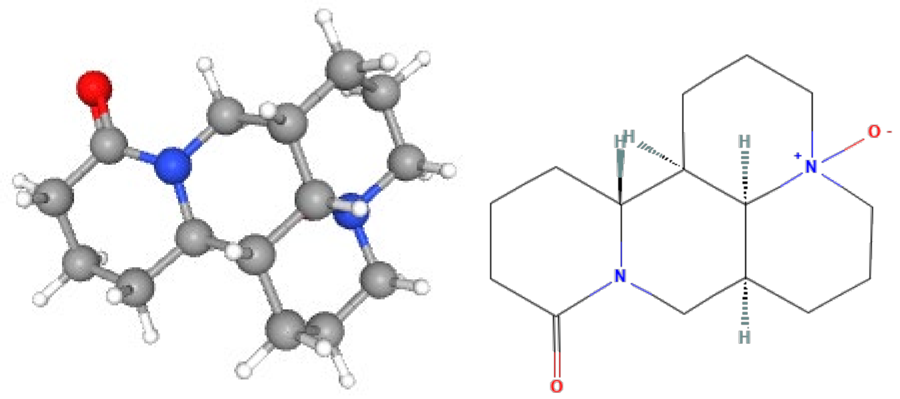

3. Sophocarpine

PubChem CID：115269

Formula:	$C_{15}H_{22}N_2O$

Molecular Weight:246.35 g/mol

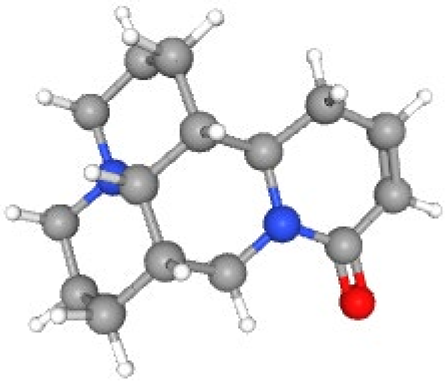

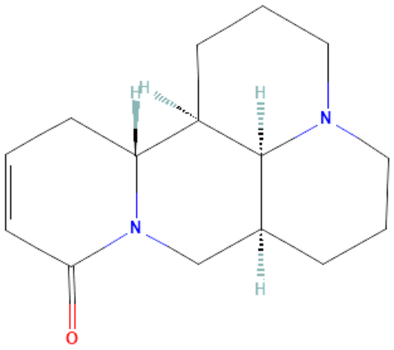

Table. S1 The composition of Kushen preparitions

Study	Formulation	Source	Species, concentration	Quality control reported? (Y/N)	Chemical analysis reported? (Y/N)
compound Kushen injection	Sophora flavescens Ait. (Kushen) and *Heterosmilax yunnanensis* Gagnep. (Baituling).	Shanxi Zhendong Pharmaceutical Co., Ltd	Sophora flavescens Ait. (Kushen) 14g and *Heterosmilax yunnanensis* Gagnep. (Baituling) 6g	Y-National Food and Drug Administration National Drug Standards, WS3-B-2752-2004； National Pharmaceutical Approval Z14021230 and Z14021231	Y –Sephadex LH-20 gel columns and reverse phase semi-preparation HPLC
Kang'ai injection	Ginseng (*Panax ginseng* C.A. Mey. [Araliaceae]), milkvetch root (*Astragalus membranaceus* [Fisch.] Bunge [Fabaceae]), and Kushen (*Sophora flavescens* Ait. [Fabaceae])	Changbai Mountain Pharmaceutical Co., Ltd	every 10 mL Kang'ai injection contains 1 g ginseng, 3 g milkvetch root, and 100 mg oxymatrine	Y-National Food and Drug Administration National Drug Standards, WS-11222(ZD-1222)-2002-2012Z； National Pharmaceutical Approval Z20026868	Y –Sephadex LH-20 gel columns and reverse phase semi-preparation HPLC
Matrine injection	Oxymatrine	Changzhou Lanling Pharmaceutical Co., Ltd，Guizhou Jinqiao Pharmaceutical Co., Ltd，Harbin Sanlian Pharmaceutical Co., Ltd， and et.al	Sophora flavescens Ait. (Kushen)	Y-National Food and Drug Administration National Drug Standards, YBH25202005 National Pharmaceutical Approval H20053736, H52020891, H20030784	N

References

[1] X. Cao, Q. He, Anti-Tumor Activities of Bioactive Phytochemicals in Sophora flavescens for Breast Cancer, Cancer management and research 12 (2020) 1457-1467.

[2] F. Chen, Y. Pan, J. Xu, B. Liu, H. Song, Research progress of matrine's anticancer activity and its molecular mechanism, Journal of ethnopharmacology 286 (2022) 114914.

[3] M.H. Chen, Y.Y. Gu, A.L. Zhang, D.M. Sze, S.L. Mo, B.H. May, Biological effects and mechanisms of matrine and other constituents of Sophora flavescens in colorectal cancer, Pharmacological research 171 (2021) 105778.

[4] Y.M. Guo, Y.X. Huang, H.H. Shen, X.X. Sang, X. Ma, Y.L. Zhao, X.H. Xiao, Efficacy of Compound Kushen Injection in Relieving Cancer-Related Pain: A Systematic Review and Meta-Analysis, Evid Based Complement Alternat Med 2015 (2015) 840742.

[5] J. Liu, Q. Yu, X.S. Wang, Q. Shi, J. Wang, F. Wang, S. Ren, J. Jin, B. Han, W. Zhang, X. Su, S. Yuan, H. Lin, Compound Kushen Injection Reduces Severe Toxicity and Symptom Burden Associated With Curative Radiotherapy in Patients With Lung Cancer, J Natl Compr Canc Netw 21(8) (2023) 821-830.e3.

[6] X. Ma, R.S. Li, J. Wang, Y.Q. Huang, P.Y. Li, J. Wang, H.B. Su, R.L. Wang, Y.M. Zhang, H.H. Liu, C.E. Zhang, Z.J. Ma, J.B. Wang, Y.L. Zhao, X.H. Xiao, The Therapeutic Efficacy and Safety of Compound Kushen Injection Combined with Transarterial Chemoembolization in Unresectable Hepatocellular Carcinoma: An Update Systematic Review and Meta-Analysis, Front Pharmacol 7 (2016) 70.

[7] C. Sun, F. Dong, T. Xiao, W. Gao, Efficacy and safety of Chinese patent medicine (Kang-ai injection) as an adjuvant in the treatment of patients with hepatocellular carcinoma: a meta-analysis, Pharm Biol 59(1) (2021) 472-483.

[8] Y.M. Wan, Y.H. Li, Z.Y. Xu, H.M. Wu, Y. Xu, M. Yang, X.N. Wu, The Effect of Transarterial Chemoembolization in Combination With Kang'ai Injection on Patients With Intermediate Stage Hepatocellular Carcinoma: A Prospective Study, Integr Cancer Ther 17(2) (2018) 477-485.

Supplementary materials.2 Related definitions and models

A. clinical responses

This analysis assessed the clinical responses using the complete response, pleurodesis failure and disease progression. The pleurodesis failure was defined as no response or stable disease combined with disease progression. Referring to previous studies [1-5], we integrated the Millar and Ostrowskimj criteria as following: (i) complete response (CR) is the disappearance of pleural effusion for more than 30 days, or the lack of accumulation of fluid; (ii) partial response(PR) is less than 50% reduction of pleural effusion for more than 30 days; (iii) no response(NR) /stable disease (SD) is less than 50% reduction of pleural effusion or less than 25% increase or the recurrence of fluid accumulation without further therapy; and (iv) pleural progression(PP) is more than 25% increase of pleural effusion, or symptomatic fluid accumulation again requiring further therapy.

B. Adverse events

This analysis assessed the adverse events using the ADRs and thoracentesis-related adverse events (TRAEs). According to the World Health Organization (WHO) [6] or Common Terminology Criteria for Adverse Events (CTCAE) standards,[7] the ADR was defined as hematotoxicity (neutropenia, thrombocytopenia, or anemia), hepatotoxicity (serum aminotransferase or alkaline phosphatase > 1.25×N), and nephrotoxicity (serum urea nitrogen or creatinine > 1.25×N), cardiotoxicity, or gastrointestinal reactions, etc.

C. Summary model of evidence quality

Following the GRADE approach [8] and integrating the results of sensitivity analysis, we developed a revised GRADE approach [4, 5, 9] to summarize the evidence quality as a “high”, “moderate”, “low” and “very low”. The quality was downgraded by according to the methodological bias risk, heterogeneity, indirectness, imprecision, or publication bias.

1. The methodological bias risk

(1) All trials had high risk, and the evidence was rated down by two levels.

(2)Most trials had some concerns and with high risk, the sensitivity analysis showed poor robustness, and the evidence was rated down by two levels.

(3)Most trials had some concerns and with high risk, the sensitivity analysis showed good robustness, and the evidence was rated down by only one level.

(4) All trials had some concerns, and the evidence was rated down by only one level.

2. Heterogeneity

(1) Heterogeneity was found in them, the sensitivity analysis showed good robustness, and not rated down.

(2) Heterogeneity was found in them, the sensitivity analysis showed poor robustness, and the evidence was rated down by one level.

3. Indirectness (following the GRADE approach)

4. Imprecision

(1) The sample size for indicator was fewer than 300 cases, and the evidence was rated down by one level.

5. Publication bias

(1) Publication bias was found among them, excluded the under- or over-estimated studies and high risk studies, the sensitivity analysis showed good robustness, and not be downgraded.

(2) Publication bias was found among them, excluded the under- or over-estimated studies and high risk studies, the sensitivity analysis showed poor robustness, and the evidence was rated down by one level.

References

[1] A. Emad, G.R. Rezaian, Treatment of malignant pleural effusions with a combination of bleomycin and tetracycline. A comparison of bleomycin or tetracycline alone versus a combination of bleomycin and tetracycline, Cancer 78(12) (1996) 2498-501.

[2] A.J. Zaloznik, S.G. Oswald, M. Langin, Intrapleural tetracycline in malignant pleural effusions. A randomized study, Cancer 51(4) (1983) 752-5.

[3] W. Keeratichananont, A. Kaewdech, S. Keeratichananont, Efficacy and safety profile of autologous blood versus talc pleurodesis for malignant pleural effusion: a randomized controlled trial, Therapeutic advances in respiratory disease 12 (2018) 1753466618816625.

[4] C.Q. Wang, X.R. Huang, M. He, X.T. Zheng, H. Jiang, Q. Chen, T.Y. Fan, L. Zhan, J. Ling, J.H. Feng, X. Xiao, X.F. Chen, Z. Xiao, Intrapleural Administration With Rh-Endostatin and Chemical Irritants in the Control of Malignant Pleural Effusion: A Systematic Review and Meta-Analysis, Front Oncol 11 (2021) 649999.

[5] Z. Xiao, Y. Jiang, X.F. Chen, C.Q. Wang, X.T. Zheng, W.H. Xu, X.X. Zou, J.M. Zhou, Y.H. Yang, S.S. Hu, L.J. Shan, Q.Y. Cai, Y.H. Tang, J.H. Feng, X. Xiao, Intrathoracic infusion therapy with Lentinan and chemical irritants for malignant pleural effusion: a systematic review and meta-analysis of 65 randomized controlled trials, Phytomedicine 76 (2020) 153260.

[6] A.B. Miller, B. Hoogstraten, M. Staquet, A. Winkler, Reporting results of cancer treatment, Cancer 47(1) (1981) 207-14.

[7] A. Trotti, A.D. Colevas, A. Setser, V. Rusch, D. Jaques, V. Budach, C. Langer, B. Murphy, R. Cumberlin,

C.N. Coleman, P. Rubin, CTCAE v3.0: development of a comprehensive grading system for the adverse effects of cancer treatment, Semin Radiat Oncol 13(3) (2003) 176-81.
[8] G.H. Guyatt, A.D. Oxman, G.E. Vist, R. Kunz, Y. Falck-Ytter, P. Alonso-Coello, H.J. Schünemann, GRADE: an emerging consensus on rating quality of evidence and strength of recommendations, Bmj 336(7650) (2008) 924-6.
[9] Z. Xiao, Y. Jiang, C.Q. Wang, S.S. Hu, X.R. Huang, X.F. Chen, J. Huang, L.J. Shan, Y.H. Tang, Y.H. Wang, Q.H. Gong, J.H. Feng, X. Xiao, X.F. Li, Clinical efficacy and safety of aidi injection combination with vinorelbine and cisplatin for advanced non-small-cell lung carcinoma: A systematic review and meta-analysis of 54 randomized controlled trials, Pharmacol Res 153 (2020) 104637.
