# Supplementary material for: Intrapleural administration with traditional Chinese medicine injections (Sophorae flavescentis preparations) in controlling malignant pleural effusion: a clustered systematic review and meta-analysis
Source: Front Pharmacol. 2025 Apr 24;16:1519794. doi: 10.3389/fphar.2025.1519794 (PMC12058796; doi:10.3389/fphar.2025.1519794)
Supplement: Supplementary file 6 [file DataSheet5.pdf]

# Supplementary materials.6 Subgroup and meta-regression analysis results (Figures.S25 to S72)

| Study or Subgroup | Kushen Events | Kushen Total | Cisplatin Events | Cisplatin Total | Weight | Odds Ratio M-H, Fixed, 95% CI | Year |
|---|---|---|---|---|---|---|---|
| **1.1.1 Miscellaneous tumors** | | | | | | | |
| Ning, X. 2001 | 17 | 30 | 8 | 30 | 2.0% | 3.60 [1.22, 10.64] | 2001 |
| Lin, S. 2007 | 20 | 33 | 10 | 33 | 2.3% | 3.54 [1.28, 9.80] | 2007 |
| Pan, J. 2007 | 15 | 36 | 12 | 34 | 4.1% | 1.31 [0.50, 3.44] | 2007 |
| Zhang, S. 2008 | 8 | 28 | 5 | 23 | 2.2% | 1.44 [0.40, 5.21] | 2008 |
| Deng, M. 2008 | 24 | 40 | 10 | 40 | 2.3% | 4.50 [1.73, 11.70] | 2008 |
| Li, Y. 2008 | 17 | 32 | 8 | 32 | 2.1% | 3.40 [1.18, 9.81] | 2008 |
| Ding, L. 2009 | 14 | 31 | 9 | 30 | 2.9% | 1.92 [0.67, 5.51] | 2009 |
| Wang, Y. 2010 | 6 | 24 | 2 | 24 | 0.9% | 3.67 [0.66, 20.42] | 2010 |
| Chen, Y. 2011 | 54 | 84 | 22 | 84 | 4.5% | 5.07 [2.62, 9.82] | 2011 |
| Ran, F. 2011 | 12 | 30 | 10 | 30 | 3.4% | 1.33 [0.46, 3.82] | 2011 |
| Wei, M. 2011 | 13 | 35 | 10 | 35 | 3.6% | 1.48 [0.54, 4.03] | 2011 |
| Li, L. 2011 | 6 | 30 | 3 | 30 | 1.4% | 2.25 [0.51, 9.99] | 2011 |
| Chen, F.2012 | 16 | 43 | 9 | 43 | 3.2% | 2.24 [0.86, 5.85] | 2012 |
| Yang, G. 2012 | 14 | 39 | 11 | 39 | 4.0% | 1.43 [0.55, 3.71] | 2012 |
| Guo, L. 2013 | 14 | 31 | 7 | 31 | 2.2% | 2.82 [0.94, 8.48] | 2013 |
| Han, S. 2013 | 35 | 90 | 13 | 90 | 4.5% | 3.77 [1.83, 7.78] | 2013 |
| Zhu, M. 2013 | 9 | 28 | 6 | 28 | 2.3% | 1.74 [0.52, 5.78] | 2013 |
| Zheng, S. 2013 | 6 | 31 | 3 | 31 | 1.4% | 2.24 [0.51, 9.91] | 2013 |
| Chen, Y. 2014 | 13 | 30 | 7 | 30 | 2.3% | 2.51 [0.83, 7.64] | 2014 |
| Jiang, J. 2014 | 11 | 34 | 7 | 34 | 2.7% | 1.84 [0.61, 5.54] | 2014 |
| Xu, B. 2014 | 12 | 30 | 3 | 30 | 1.0% | 6.00 [1.48, 24.30] | 2014 |
| Xu, L. 2014 | 18 | 32 | 13 | 32 | 3.3% | 1.88 [0.70, 5.07] | 2014 |
| Song, X. 2015 | 47 | 59 | 30 | 59 | 3.5% | 3.79 [1.68, 8.54] | 2015 |
| Yan, G. 2016 | 22 | 35 | 12 | 30 | 2.7% | 2.54 [0.93, 6.91] | 2016 |
| Qin,D.2016 | 11 | 32 | 6 | 32 | 2.3% | 2.27 [0.72, 7.16] | 2016 |
| Huang, H. 2017 | 20 | 30 | 11 | 30 | 2.1% | 3.45 [1.19, 9.99] | 2017 |
| Jiang, T 2020 | 24 | 30 | 6 | 30 | 0.7% | 16.00 [4.52, 56.70] | 2020 |
| Lin, W.2023 | 8 | 26 | 5 | 26 | 2.0% | 1.87 [0.52, 6.73] | 2023 |
| **Subtotal (95% CI)** | | **1033** | | **1020** | **71.9%** | **2.77 [2.29, 3.37]** | |
| Total events | 486 | | 258 | | | | |
| Heterogeneity: Chi² = 26.44, df = 27 (P = 0.49); I² = 0% | | | | | | | |
| Test for overall effect: Z = 10.35 (P < 0.00001) | | | | | | | |
| **1.1.2 Lung cancer** | | | | | | | |
| Huang, X. 2007 | 9 | 20 | 4 | 18 | 1.3% | 2.86 [0.69, 11.82] | 2007 |
| Li, Y. 2009 | 17 | 30 | 8 | 30 | 2.0% | 3.60 [1.22, 10.64] | 2009 |
| He, L. 2010 | 7 | 24 | 5 | 20 | 2.2% | 1.24 [0.32, 4.72] | 2010 |
| Han, Z. 2012 | 10 | 28 | 5 | 28 | 1.8% | 2.56 [0.74, 8.81] | 2012 |
| Liu, D. 2015 | 20 | 46 | 6 | 42 | 2.0% | 4.62 [1.63, 13.09] | 2015 |
| Liu, L. 2017 | 13 | 30 | 7 | 30 | 2.3% | 2.51 [0.83, 7.64] | 2017 |
| Shi, W. 2017 | 14 | 30 | 8 | 30 | 2.4% | 2.41 [0.82, 7.10] | 2017 |
| Tang, X. 2018 | 15 | 30 | 10 | 30 | 2.9% | 2.00 [0.70, 5.68] | 2018 |
| Wang, Y. 2019 | 20 | 45 | 9 | 45 | 2.9% | 3.20 [1.25, 8.17] | 2019 |
| Wu, C. 2019 | 8 | 25 | 4 | 25 | 1.6% | 2.47 [0.63, 9.63] | 2019 |
| Peng,H.2020 | 12 | 25 | 5 | 25 | 1.5% | 3.69 [1.05, 12.96] | 2020 |
| Feng, F.2023 | 11 | 34 | 7 | 34 | 2.7% | 1.84 [0.61, 5.54] | 2023 |
| **Subtotal (95% CI)** | | **367** | | **357** | **25.6%** | **2.68 [1.93, 3.72]** | |
| Total events | 156 | | 78 | | | | |
| Heterogeneity: Chi² = 3.82, df = 11 (P = 0.97); I² = 0% | | | | | | | |
| Test for overall effect: Z = 5.90 (P < 0.00001) | | | | | | | |
| **1.1.3 Hematologic malignancies** | | | | | | | |
| Zhuang, H. 2012 | 7 | 24 | 6 | 22 | 2.5% | 1.10 [0.30, 3.98] | 2012 |
| **Subtotal (95% CI)** | | **24** | | **22** | **2.5%** | **1.10 [0.30, 3.98]** | |
| Total events | 7 | | 6 | | | | |
| Heterogeneity: Not applicable | | | | | | | |
| Test for overall effect: Z = 0.14 (P = 0.89) | | | | | | | |
| **Total (95% CI)** | | **1424** | | **1399** | **100.0%** | **2.71 [2.30, 3.19]** | |
| Total events | 649 | | 342 | | | | |
| Heterogeneity: Chi² = 32.18, df = 40 (P = 0.81); I² = 0% | | | | | | | |
| Test for overall effect: Z = 11.84 (P < 0.00001) | | | | | | | |
| Test for subgroup differences: Chi² = 1.96, df = 2 (P = 0.38), I² = 0% | | | | | | | |

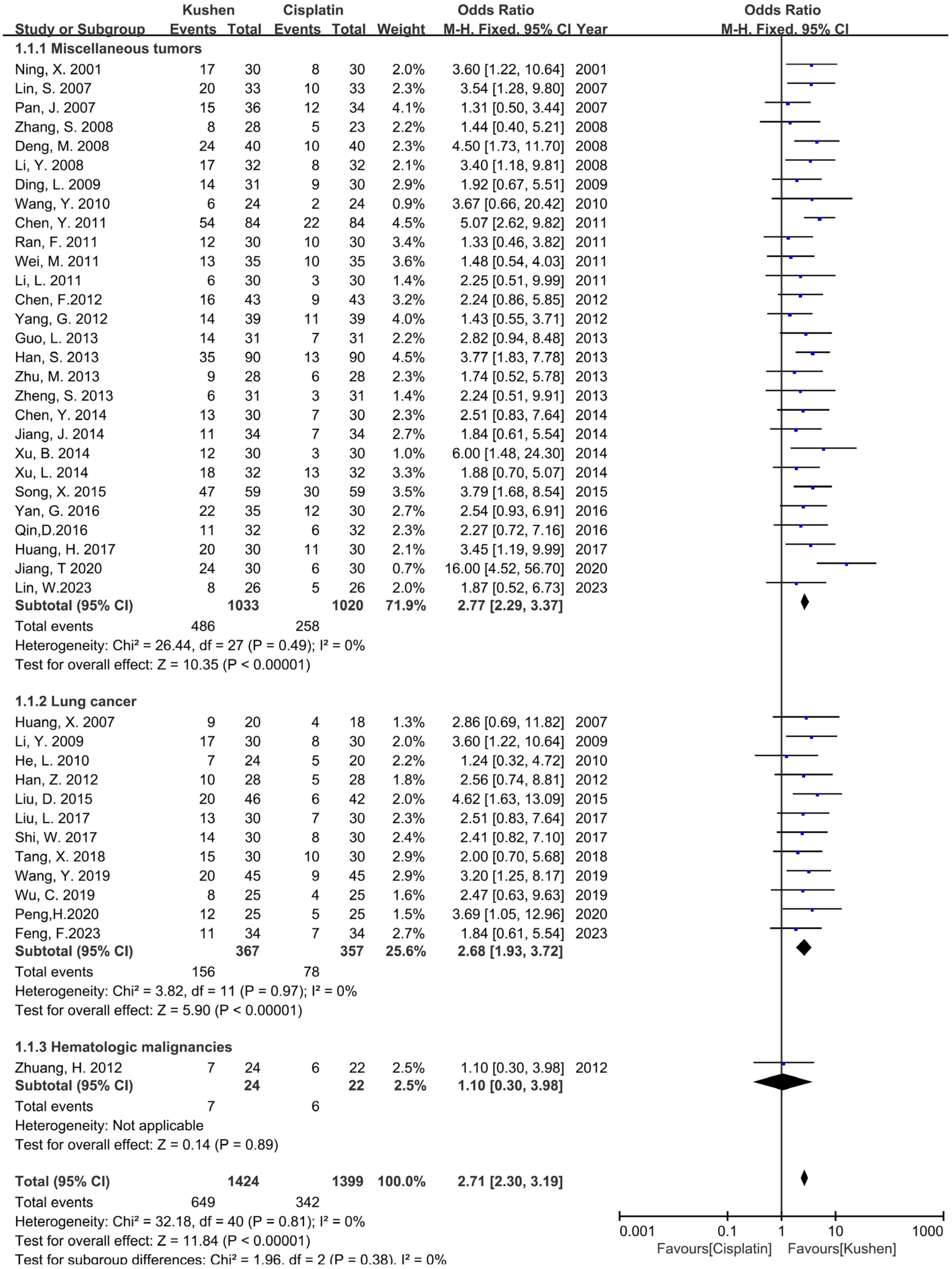


**Figure.S25 Subgroups analysis of complete response via primary disease**

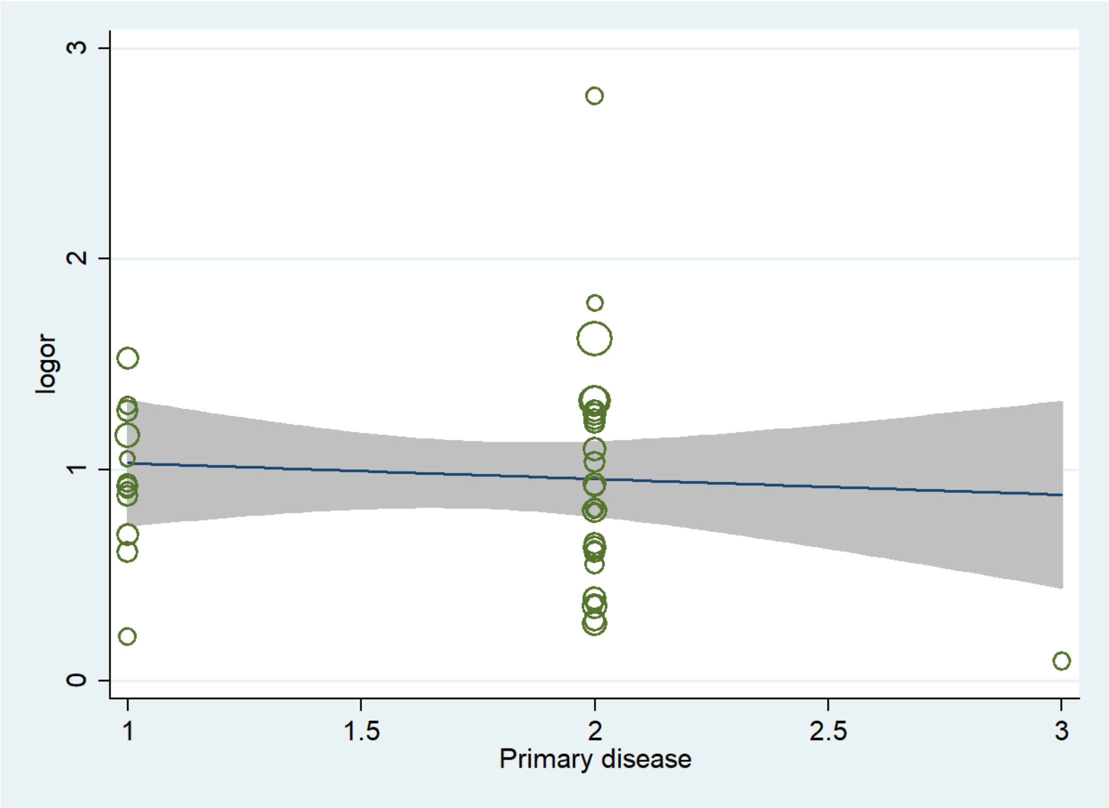


**Figure.S26 Meta-regression of complete response via primary disease**

| Study or Subgroup | Kushen Events | Kushen Total | Cisplatin Events | Cisplatin Total | Weight | Odds Ratio M-H, Fixed, 95% CI | Year |
|---|---|---|---|---|---|---|---|
| **1.2.1 Miscellaneous tumors** | | | | | | | |
| Ning, X. 2001 | 5 | 30 | 8 | 30 | 1.4% | 0.55 [0.16, 1.93] | 2001 |
| Pan, J. 2007 | 3 | 36 | 9 | 34 | 1.7% | 0.25 [0.06, 1.03] | 2007 |
| Lin, S. 2007 | 4 | 33 | 11 | 33 | 2.0% | 0.28 [0.08, 0.98] | 2007 |
| Li, Y. 2008 | 5 | 32 | 8 | 32 | 1.4% | 0.56 [0.16, 1.93] | 2008 |
| Zhang, S. 2008 | 5 | 28 | 10 | 23 | 1.8% | 0.28 [0.08, 1.01] | 2008 |
| Deng, M. 2008 | 6 | 40 | 12 | 40 | 2.1% | 0.41 [0.14, 1.24] | 2008 |
| Ding, L. 2009 | 3 | 31 | 13 | 30 | 2.4% | 0.14 [0.03, 0.56] | 2009 |
| Wang, Y. 2010 | 2 | 24 | 10 | 24 | 1.9% | 0.13 [0.02, 0.67] | 2010 |
| Chen, Y. 2011 | 10 | 84 | 38 | 84 | 6.8% | 0.16 [0.07, 0.36] | 2011 |
| Wei, M. 2011 | 6 | 35 | 11 | 35 | 1.9% | 0.45 [0.15, 1.40] | 2011 |
| Li, L. 2011 | 7 | 30 | 17 | 30 | 2.6% | 0.23 [0.08, 0.71] | 2011 |
| Ran, F. 2011 | 4 | 30 | 12 | 30 | 2.1% | 0.23 [0.06, 0.83] | 2011 |
| Chen, F.2012 | 7 | 43 | 20 | 43 | 3.4% | 0.22 [0.08, 0.61] | 2012 |
| Yang, G. 2012 | 6 | 39 | 16 | 39 | 2.7% | 0.26 [0.09, 0.77] | 2012 |
| Han, S. 2013 | 11 | 90 | 40 | 90 | 7.1% | 0.17 [0.08, 0.37] | 2013 |
| Zhu, M. 2013 | 5 | 28 | 9 | 28 | 1.5% | 0.46 [0.13, 1.60] | 2013 |
| Guo, L. 2013 | 7 | 31 | 15 | 31 | 2.4% | 0.31 [0.10, 0.93] | 2013 |
| Zheng, S. 2013 | 6 | 31 | 16 | 31 | 2.6% | 0.23 [0.07, 0.70] | 2013 |
| Jiang, J. 2014 | 5 | 34 | 13 | 34 | 2.3% | 0.28 [0.09, 0.90] | 2014 |
| Chen, Y. 2014 | 7 | 30 | 14 | 30 | 2.2% | 0.35 [0.11, 1.05] | 2014 |
| Xu, L. 2014 | 7 | 32 | 15 | 32 | 2.4% | 0.32 [0.11, 0.94] | 2014 |
| Xu, B. 2014 | 11 | 30 | 17 | 30 | 2.2% | 0.44 [0.16, 1.25] | 2014 |
| Song, X. 2015 | 5 | 59 | 19 | 59 | 3.5% | 0.19 [0.07, 0.57] | 2015 |
| Qin,D.2016 | 2 | 32 | 15 | 32 | 2.9% | 0.08 [0.02, 0.37] | 2016 |
| Yan, G. 2016 | 4 | 35 | 11 | 30 | 2.1% | 0.22 [0.06, 0.80] | 2016 |
| Huang, H. 2017 | 3 | 30 | 11 | 30 | 2.0% | 0.19 [0.05, 0.78] | 2017 |
| Jiang, T 2020 | 2 | 30 | 13 | 30 | 2.5% | 0.09 [0.02, 0.47] | 2020 |
| Lin, W.2023 | 7 | 26 | 15 | 26 | 2.2% | 0.27 [0.08, 0.87] | 2023 |
| **Subtotal (95% CI)** | | **1033** | | **1020** | **72.0%** | **0.25 [0.20, 0.31]** | |
| Total events | 155 | | 418 | | | | |
| Heterogeneity: Chi² = 15.21, df = 27 (P = 0.97); I² = 0% | | | | | | | |
| Test for overall effect: Z = 12.73 (P < 0.00001) | | | | | | | |
| **1.2.2 Lung cancer** | | | | | | | |
| Huang, X. 2007 | 3 | 20 | 8 | 18 | 1.5% | 0.22 [0.05, 1.03] | 2007 |
| Li, Y. 2009 | 5 | 30 | 8 | 30 | 1.4% | 0.55 [0.16, 1.93] | 2009 |
| He, L. 2010 | 8 | 24 | 11 | 20 | 1.6% | 0.41 [0.12, 1.39] | 2010 |
| Han, Z. 2012 | 5 | 28 | 13 | 28 | 2.2% | 0.25 [0.07, 0.85] | 2012 |
| Liu, D. 2015 | 8 | 46 | 21 | 42 | 3.7% | 0.21 [0.08, 0.56] | 2015 |
| Liu, L. 2017 | 6 | 30 | 14 | 30 | 2.3% | 0.29 [0.09, 0.90] | 2017 |
| Shi, W. 2017 | 6 | 30 | 13 | 30 | 2.1% | 0.33 [0.10, 1.03] | 2017 |
| Tang, X. 2018 | 4 | 30 | 11 | 30 | 1.9% | 0.27 [0.07, 0.96] | 2018 |
| Wang, Y. 2019 | 8 | 45 | 20 | 45 | 3.3% | 0.27 [0.10, 0.71] | 2019 |
| Wu, C. 2019 | 7 | 25 | 15 | 25 | 2.2% | 0.26 [0.08, 0.85] | 2019 |
| Peng,H.2020 | 7 | 25 | 15 | 25 | 2.2% | 0.26 [0.08, 0.85] | 2020 |
| Feng, F.2023 | 7 | 34 | 15 | 34 | 2.4% | 0.33 [0.11, 0.96] | 2023 |
| **Subtotal (95% CI)** | | **367** | | **357** | **26.7%** | **0.29 [0.21, 0.40]** | |
| Total events | 74 | | 164 | | | | |
| Heterogeneity: Chi² = 2.09, df = 11 (P = 1.00); I² = 0% | | | | | | | |
| Test for overall effect: Z = 7.30 (P < 0.00001) | | | | | | | |
| **1.2.3 Hematologic malignancies** | | | | | | | |
| Zhuang, H. 2012 | 6 | 24 | 8 | 22 | 1.3% | 0.58 [0.16, 2.07] | 2012 |
| **Subtotal (95% CI)** | | **24** | | **22** | **1.3%** | **0.58 [0.16, 2.07]** | |
| Total events | 6 | | 8 | | | | |
| Heterogeneity: Not applicable | | | | | | | |
| Test for overall effect: Z = 0.83 (P = 0.40) | | | | | | | |
| **Total (95% CI)** | | **1424** | | **1399** | **100.0%** | **0.26 [0.22, 0.32]** | |
| Total events | 235 | | 590 | | | | |
| Heterogeneity: Chi² = 19.19, df = 40 (P = 1.00); I² = 0% | | | | | | | |
| Test for overall effect: Z = 14.66 (P < 0.00001) | | | | | | | |
| Test for subgroup differences: Chi² = 2.08, df = 2 (P = 0.35), I² = 3.8% | | | | | | | |

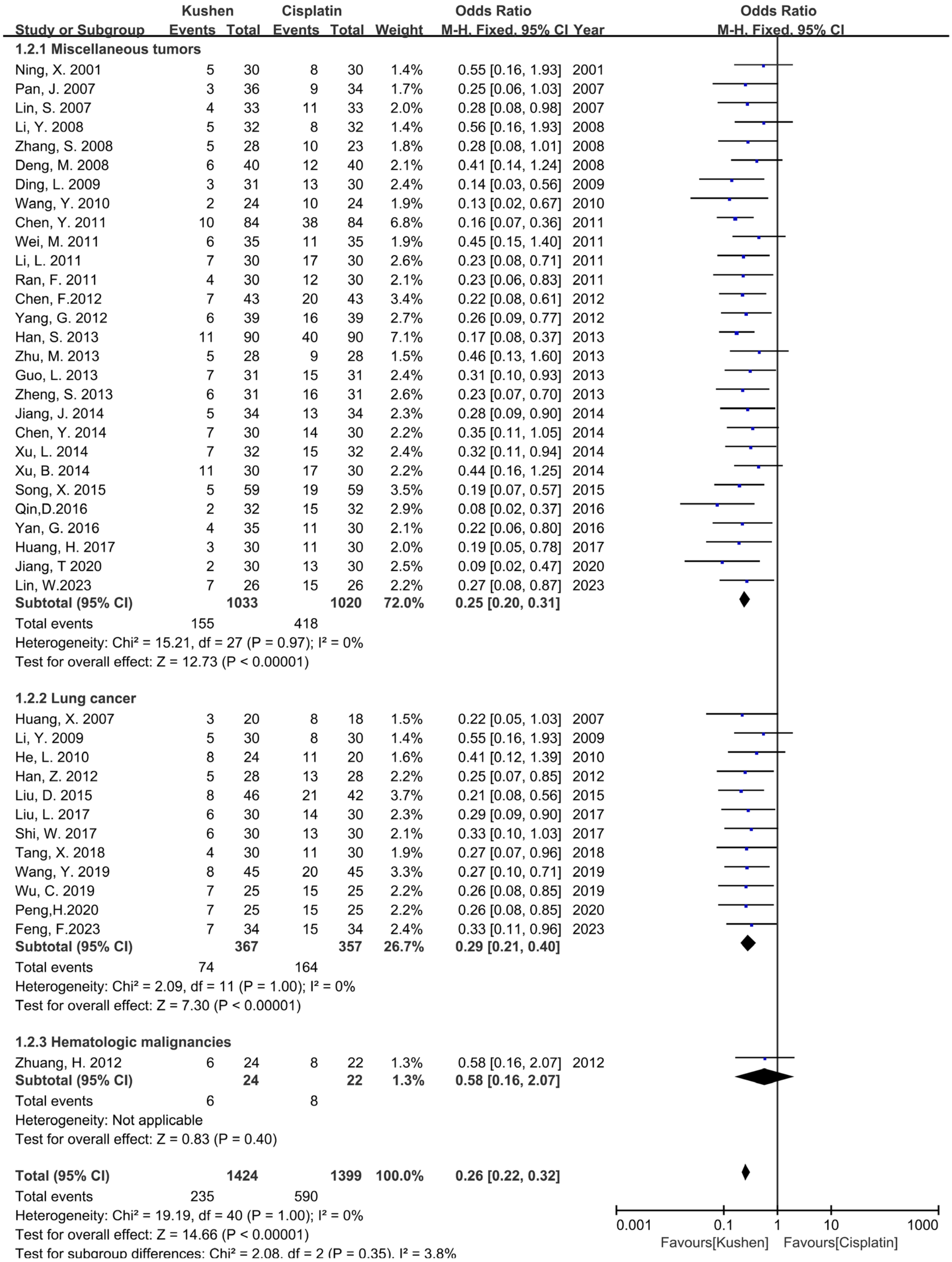


**Figure.S27 Subgroups analysis of pleurodesis failure via primary disease**

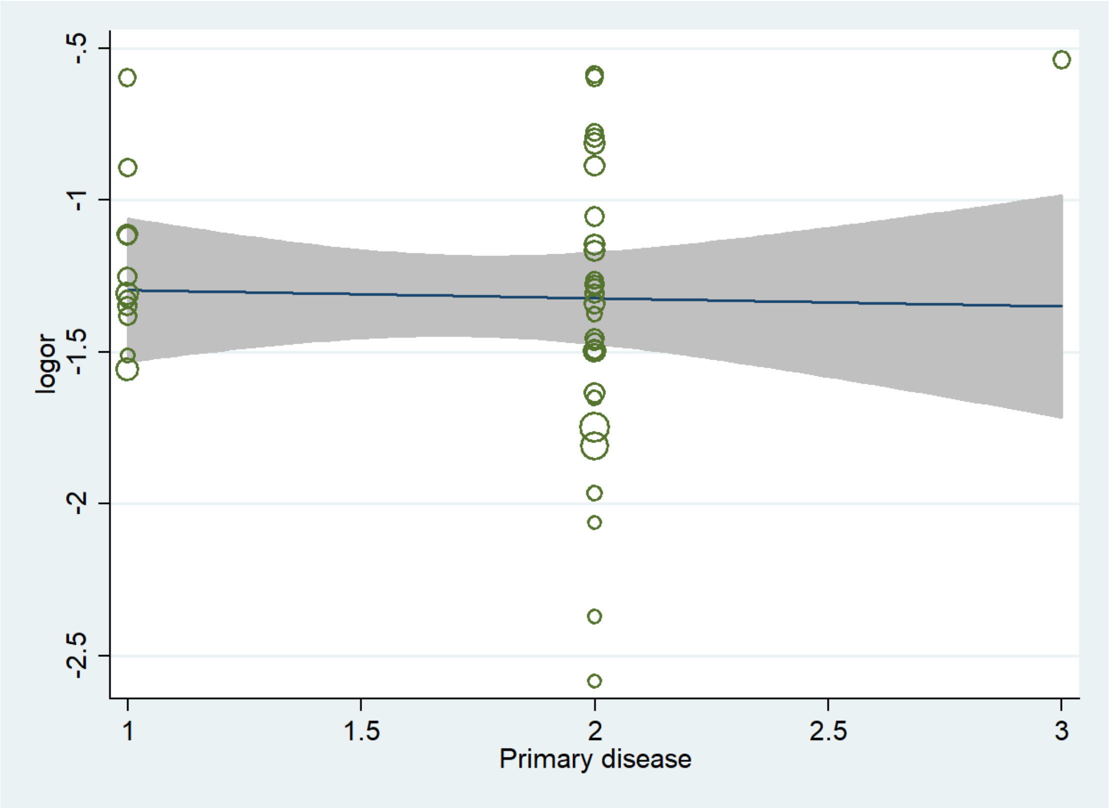


**Figure.S28 Meta-regression of pleurodesis failure via primary disease**

| Study or Subgroup | Kushen Events | Kushen Total | Cisplatin Events | Cisplatin Total | Weight | Odds Ratio M-H, Fixed, 95% CI | Year |
|---|---|---|---|---|---|---|---|
| **1.1.1 Small to large** | | | | | | | |
| Lin, W.2023 | 8 | 26 | 5 | 26 | 2.0% | 1.87 [0.52, 6.73] | 2023 |
| **Subtotal (95% CI)** | | **26** | | **26** | **2.0%** | **1.87 [0.52, 6.73]** | |
| Total events | 8 | | 5 | | | | |
| Heterogeneity: Not applicable | | | | | | | |
| Test for overall effect: Z = 0.95 (P = 0.34) | | | | | | | |
| **1.1.2 Moderate to large** | | | | | | | |
| Lin, S. 2007 | 20 | 33 | 10 | 33 | 2.3% | 3.54 [1.28, 9.80] | 2007 |
| He, L. 2010 | 7 | 24 | 5 | 20 | 2.2% | 1.24 [0.32, 4.72] | 2010 |
| Li, L. 2011 | 6 | 30 | 3 | 30 | 1.4% | 2.25 [0.51, 9.99] | 2011 |
| Chen, F.2012 | 16 | 43 | 9 | 43 | 3.2% | 2.24 [0.86, 5.85] | 2012 |
| Yang, G. 2012 | 14 | 39 | 11 | 39 | 4.0% | 1.43 [0.55, 3.71] | 2012 |
| Zhu, M. 2013 | 9 | 28 | 6 | 28 | 2.3% | 1.74 [0.52, 5.78] | 2013 |
| Guo, L. 2013 | 14 | 31 | 7 | 31 | 2.2% | 2.82 [0.94, 8.48] | 2013 |
| Chen, Y. 2014 | 13 | 30 | 7 | 30 | 2.3% | 2.51 [0.83, 7.64] | 2014 |
| Liu, L. 2017 | 13 | 30 | 7 | 30 | 2.3% | 2.51 [0.83, 7.64] | 2017 |
| Feng, F.2023 | 11 | 34 | 7 | 34 | 2.7% | 1.84 [0.61, 5.54] | 2023 |
| **Subtotal (95% CI)** | | **322** | | **318** | **24.9%** | **2.15 [1.51, 3.05]** | |
| Total events | 123 | | 72 | | | | |
| Heterogeneity: Chi² = 2.88, df = 9 (P = 0.97); I² = 0% | | | | | | | |
| Test for overall effect: Z = 4.29 (P < 0.0001) | | | | | | | |
| **1.1.3 Large** | | | | | | | |
| Huang, X. 2007 | 9 | 20 | 4 | 18 | 1.3% | 2.86 [0.69, 11.82] | 2007 |
| Shi, W. 2017 | 14 | 30 | 8 | 30 | 2.4% | 2.41 [0.82, 7.10] | 2017 |
| Tang, X. 2018 | 15 | 30 | 10 | 30 | 2.9% | 2.00 [0.70, 5.68] | 2018 |
| **Subtotal (95% CI)** | | **80** | | **78** | **6.6%** | **2.32 [1.20, 4.50]** | |
| Total events | 38 | | 22 | | | | |
| Heterogeneity: Chi² = 0.17, df = 2 (P = 0.92); I² = 0% | | | | | | | |
| Test for overall effect: Z = 2.49 (P = 0.01) | | | | | | | |
| **1.1.4 Unclear** | | | | | | | |
| Ning, X. 2001 | 17 | 30 | 8 | 30 | 2.0% | 3.60 [1.22, 10.64] | 2001 |
| Pan, J. 2007 | 15 | 36 | 12 | 34 | 4.1% | 1.31 [0.50, 3.44] | 2007 |
| Zhang, S. 2008 | 8 | 28 | 5 | 23 | 2.2% | 1.44 [0.40, 5.21] | 2008 |
| Deng, M. 2008 | 24 | 40 | 10 | 40 | 2.3% | 4.50 [1.73, 11.70] | 2008 |
| Li, Y. 2008 | 17 | 32 | 8 | 32 | 2.1% | 3.40 [1.18, 9.81] | 2008 |
| Li, Y. 2009 | 17 | 30 | 8 | 30 | 2.0% | 3.60 [1.22, 10.64] | 2009 |
| Ding, L. 2009 | 14 | 31 | 9 | 30 | 2.9% | 1.92 [0.67, 5.51] | 2009 |
| Wang, Y. 2010 | 6 | 24 | 2 | 24 | 0.9% | 3.67 [0.66, 20.42] | 2010 |
| Chen, Y. 2011 | 54 | 84 | 22 | 84 | 4.5% | 5.07 [2.62, 9.82] | 2011 |
| Ran, F. 2011 | 12 | 30 | 10 | 30 | 3.4% | 1.33 [0.46, 3.82] | 2011 |
| Wei, M. 2011 | 13 | 35 | 10 | 35 | 3.6% | 1.48 [0.54, 4.03] | 2011 |
| Han, Z. 2012 | 10 | 28 | 5 | 28 | 1.8% | 2.56 [0.74, 8.81] | 2012 |
| Zhuang, H. 2012 | 7 | 24 | 6 | 22 | 2.5% | 1.10 [0.30, 3.98] | 2012 |
| Han, S. 2013 | 35 | 90 | 13 | 90 | 4.5% | 3.77 [1.83, 7.78] | 2013 |
| Zheng, S. 2013 | 6 | 31 | 3 | 31 | 1.4% | 2.24 [0.51, 9.91] | 2013 |
| Jiang, J. 2014 | 11 | 34 | 7 | 34 | 2.7% | 1.84 [0.61, 5.54] | 2014 |
| Xu, B. 2014 | 12 | 30 | 3 | 30 | 1.0% | 6.00 [1.48, 24.30] | 2014 |
| Xu, L. 2014 | 18 | 32 | 13 | 32 | 3.3% | 1.88 [0.70, 5.07] | 2014 |
| Song, X. 2015 | 47 | 59 | 30 | 59 | 3.5% | 3.79 [1.68, 8.54] | 2015 |
| Liu, D. 2015 | 20 | 46 | 6 | 42 | 2.0% | 4.62 [1.63, 13.09] | 2015 |
| Yan, G. 2016 | 22 | 35 | 12 | 30 | 2.7% | 2.54 [0.93, 6.91] | 2016 |
| Qin,D.2016 | 11 | 32 | 6 | 32 | 2.3% | 2.27 [0.72, 7.16] | 2016 |
| Huang, H. 2017 | 20 | 30 | 11 | 30 | 2.1% | 3.45 [1.19, 9.99] | 2017 |
| Wang, Y. 2019 | 20 | 45 | 9 | 45 | 2.9% | 3.20 [1.25, 8.17] | 2019 |
| Wu, C. 2019 | 8 | 25 | 4 | 25 | 1.6% | 2.47 [0.63, 9.63] | 2019 |
| Peng,H.2020 | 12 | 25 | 5 | 25 | 1.5% | 3.69 [1.05, 12.96] | 2020 |
| Jiang, T 2020 | 24 | 30 | 6 | 30 | 0.7% | 16.00 [4.52, 56.70] | 2020 |
| **Subtotal (95% CI)** | | **996** | | **977** | **66.5%** | **2.98 [2.45, 3.63]** | |
| Total events | 480 | | 243 | | | | |
| Heterogeneity: Chi² = 26.17, df = 26 (P = 0.45); I² = 1% | | | | | | | |
| Test for overall effect: Z = 10.83 (P < 0.00001) | | | | | | | |
| **Total (95% CI)** | | **1424** | | **1399** | **100.0%** | **2.71 [2.30, 3.19]** | |
| Total events | 649 | | 342 | | | | |
| Heterogeneity: Chi² = 32.18, df = 40 (P = 0.81); I² = 0% | | | | | | | |
| Test for overall effect: Z = 11.84 (P < 0.00001) | | | | | | | |
| Test for subgroup differences: Chi² = 3.12, df = 3 (P = 0.37), I² = 3.9% | | | | | | | |

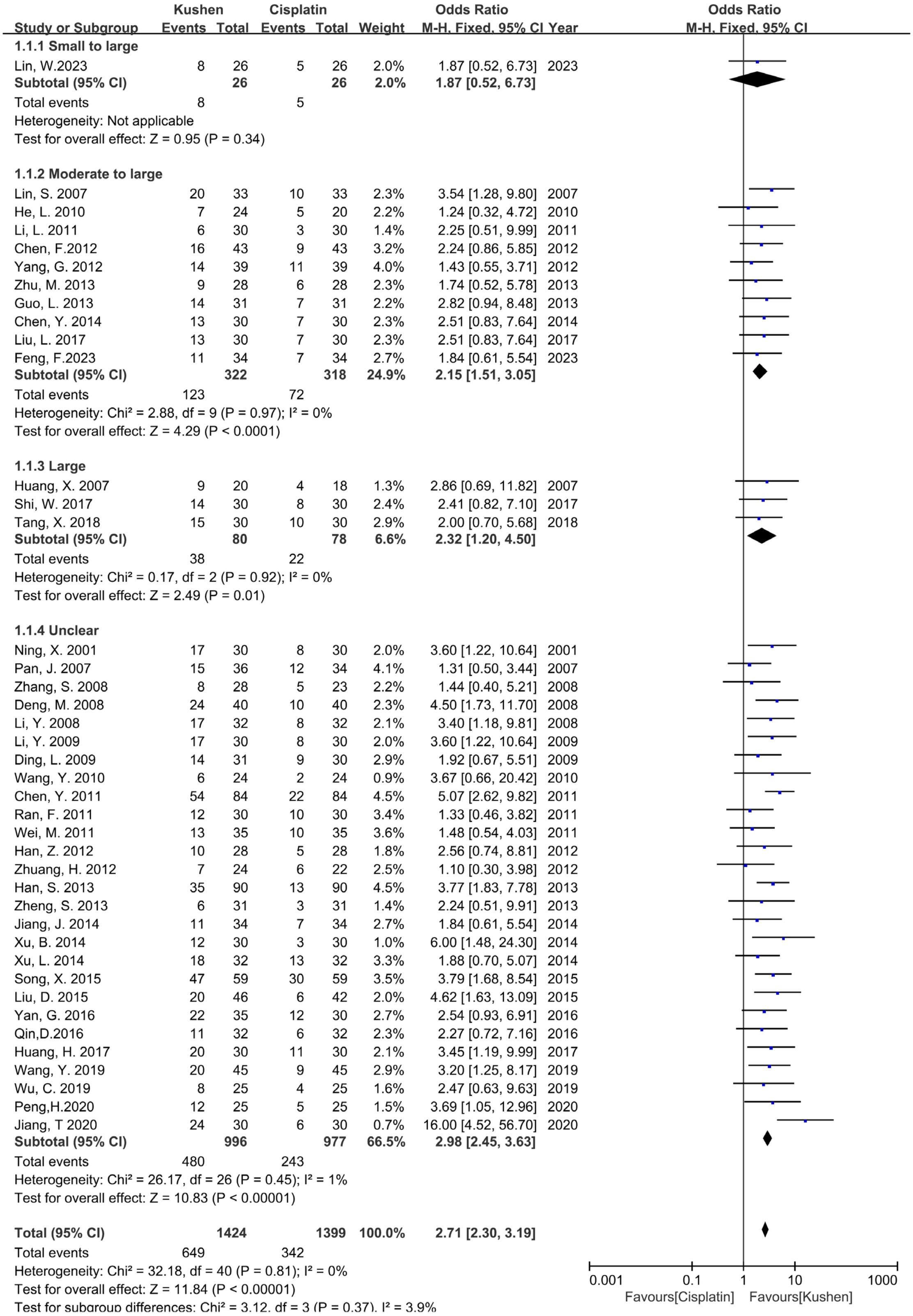


**Figure.S29 Subgroups analysis of complete response via pleural effusion**

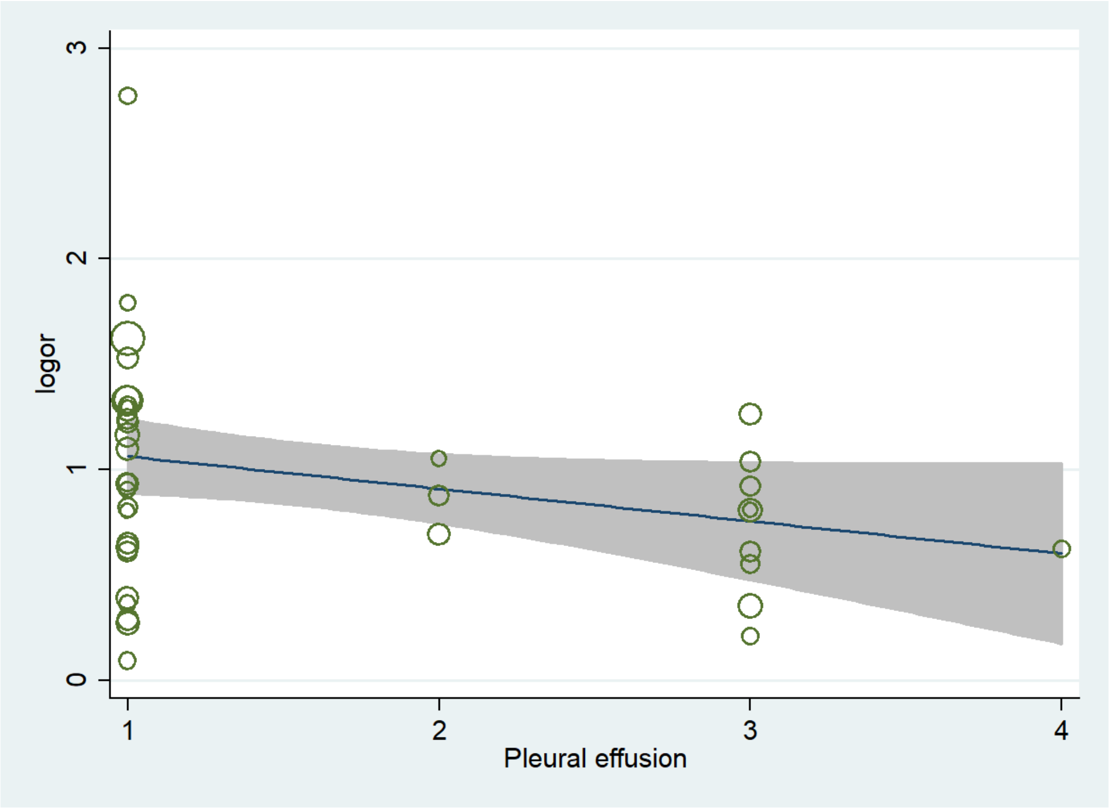


**Figure.S30 Meta-regression of complete response via pleural effusion**

| Study or Subgroup | Kushen Events | Kushen Total | Cisplatin Events | Cisplatin Total | Weight | Odds Ratio M-H, Fixed, 95% CI |
|---|---|---|---|---|---|---|
| **1.2.1 Small to large** | | | | | | |
| Lin, W.2023 | 7 | 26 | 15 | 26 | 2.2% | 0.27 [0.08, 0.87] |
| **Subtotal (95% CI)** | | **26** | | **26** | **2.2%** | **0.27 [0.08, 0.87]** |
| Total events | 7 | | 15 | | | |
| Heterogeneity: Not applicable | | | | | | |
| Test for overall effect: Z = 2.20 (P = 0.03) | | | | | | |
| **1.2.2 Moderate to large** | | | | | | |
| Chen, F.2012 | 7 | 43 | 20 | 43 | 3.4% | 0.22 [0.08, 0.61] |
| Chen, Y. 2014 | 7 | 30 | 14 | 30 | 2.2% | 0.35 [0.11, 1.05] |
| Feng, F.2023 | 7 | 34 | 15 | 34 | 2.4% | 0.33 [0.11, 0.96] |
| Guo, L. 2013 | 7 | 31 | 15 | 31 | 2.4% | 0.31 [0.10, 0.93] |
| He, L. 2010 | 8 | 24 | 11 | 20 | 1.6% | 0.41 [0.12, 1.39] |
| Li, L. 2011 | 7 | 30 | 17 | 30 | 2.6% | 0.23 [0.08, 0.71] |
| Lin, S. 2007 | 4 | 33 | 11 | 33 | 2.0% | 0.28 [0.08, 0.98] |
| Liu, L. 2017 | 6 | 30 | 14 | 30 | 2.3% | 0.29 [0.09, 0.90] |
| Yang, G. 2012 | 6 | 39 | 16 | 39 | 2.7% | 0.26 [0.09, 0.77] |
| Zhu, M. 2013 | 5 | 28 | 9 | 28 | 1.5% | 0.46 [0.13, 1.60] |
| **Subtotal (95% CI)** | | **322** | | **318** | **23.1%** | **0.30 [0.21, 0.43]** |
| Total events | 64 | | 142 | | | |
| Heterogeneity: Chi² = 1.40, df = 9 (P = 1.00); I² = 0% | | | | | | |
| Test for overall effect: Z = 6.64 (P < 0.00001) | | | | | | |
| **1.2.3 Large** | | | | | | |
| Huang, X. 2007 | 3 | 20 | 8 | 18 | 1.5% | 0.22 [0.05, 1.03] |
| Shi, W. 2017 | 6 | 30 | 13 | 30 | 2.1% | 0.33 [0.10, 1.03] |
| Tang, X. 2018 | 4 | 30 | 11 | 30 | 1.9% | 0.27 [0.07, 0.96] |
| **Subtotal (95% CI)** | | **80** | | **78** | **5.5%** | **0.28 [0.13, 0.59]** |
| Total events | 13 | | 32 | | | |
| Heterogeneity: Chi² = 0.17, df = 2 (P = 0.92); I² = 0% | | | | | | |
| Test for overall effect: Z = 3.36 (P = 0.0008) | | | | | | |
| **1.2.4 Unclear** | | | | | | |
| Chen, Y. 2011 | 10 | 84 | 38 | 84 | 6.8% | 0.16 [0.07, 0.36] |
| Deng, M. 2008 | 6 | 40 | 12 | 40 | 2.1% | 0.41 [0.14, 1.24] |
| Ding, L. 2009 | 3 | 31 | 13 | 30 | 2.4% | 0.14 [0.03, 0.56] |
| Han, S. 2013 | 11 | 90 | 40 | 90 | 7.1% | 0.17 [0.08, 0.37] |
| Han, Z. 2012 | 5 | 28 | 13 | 28 | 2.2% | 0.25 [0.07, 0.85] |
| Huang, H. 2017 | 3 | 30 | 11 | 30 | 2.0% | 0.19 [0.05, 0.78] |
| Jiang, J. 2014 | 5 | 34 | 13 | 34 | 2.3% | 0.28 [0.09, 0.90] |
| Jiang, T 2020 | 2 | 30 | 13 | 30 | 2.5% | 0.09 [0.02, 0.47] |
| Li, Y. 2008 | 5 | 32 | 8 | 32 | 1.4% | 0.56 [0.16, 1.93] |
| Li, Y. 2009 | 5 | 30 | 8 | 30 | 1.4% | 0.55 [0.16, 1.93] |
| Liu, D. 2015 | 8 | 46 | 21 | 42 | 3.7% | 0.21 [0.08, 0.56] |
| Ning, X. 2001 | 5 | 30 | 8 | 30 | 1.4% | 0.55 [0.16, 1.93] |
| Pan, J. 2007 | 3 | 36 | 9 | 34 | 1.7% | 0.25 [0.06, 1.03] |
| Peng,H.2020 | 7 | 25 | 15 | 25 | 2.2% | 0.26 [0.08, 0.85] |
| Qin,D.2016 | 2 | 32 | 15 | 32 | 2.9% | 0.08 [0.02, 0.37] |
| Ran, F. 2011 | 4 | 30 | 12 | 30 | 2.1% | 0.23 [0.06, 0.83] |
| Song, X. 2015 | 5 | 59 | 19 | 59 | 3.5% | 0.19 [0.07, 0.57] |
| Wang, Y. 2010 | 2 | 24 | 10 | 24 | 1.9% | 0.13 [0.02, 0.67] |
| Wang, Y. 2019 | 8 | 45 | 20 | 45 | 3.3% | 0.27 [0.10, 0.71] |
| Wei, M. 2011 | 6 | 35 | 11 | 35 | 1.9% | 0.45 [0.15, 1.40] |
| Wu, C. 2019 | 7 | 25 | 15 | 25 | 2.2% | 0.26 [0.08, 0.85] |
| Xu, B. 2014 | 11 | 30 | 17 | 30 | 2.2% | 0.44 [0.16, 1.25] |
| Xu, L. 2014 | 7 | 32 | 15 | 32 | 2.4% | 0.32 [0.11, 0.94] |
| Yan, G. 2016 | 4 | 35 | 11 | 30 | 2.1% | 0.22 [0.06, 0.80] |
| Zhang, S. 2008 | 5 | 28 | 10 | 23 | 1.8% | 0.28 [0.08, 1.01] |
| Zheng, S. 2013 | 6 | 31 | 16 | 31 | 2.6% | 0.23 [0.07, 0.70] |
| Zhuang, H. 2012 | 6 | 24 | 8 | 22 | 1.3% | 0.58 [0.16, 2.07] |
| **Subtotal (95% CI)** | | **996** | | **977** | **69.2%** | **0.25 [0.20, 0.31]** |
| Total events | 151 | | 401 | | | |
| Heterogeneity: Chi² = 17.06, df = 26 (P = 0.91); I² = 0% | | | | | | |
| Test for overall effect: Z = 12.44 (P < 0.00001) | | | | | | |
| **Total (95% CI)** | | **1424** | | **1399** | **100.0%** | **0.26 [0.22, 0.32]** |
| Total events | 235 | | 590 | | | |
| Heterogeneity: Chi² = 19.19, df = 40 (P = 1.00); I² = 0% | | | | | | |
| Test for overall effect: Z = 14.66 (P < 0.00001) | | | | | | |
| Test for subgroup differences: Chi² = 0.72, df = 3 (P = 0.87), I² = 0% | | | | | | |

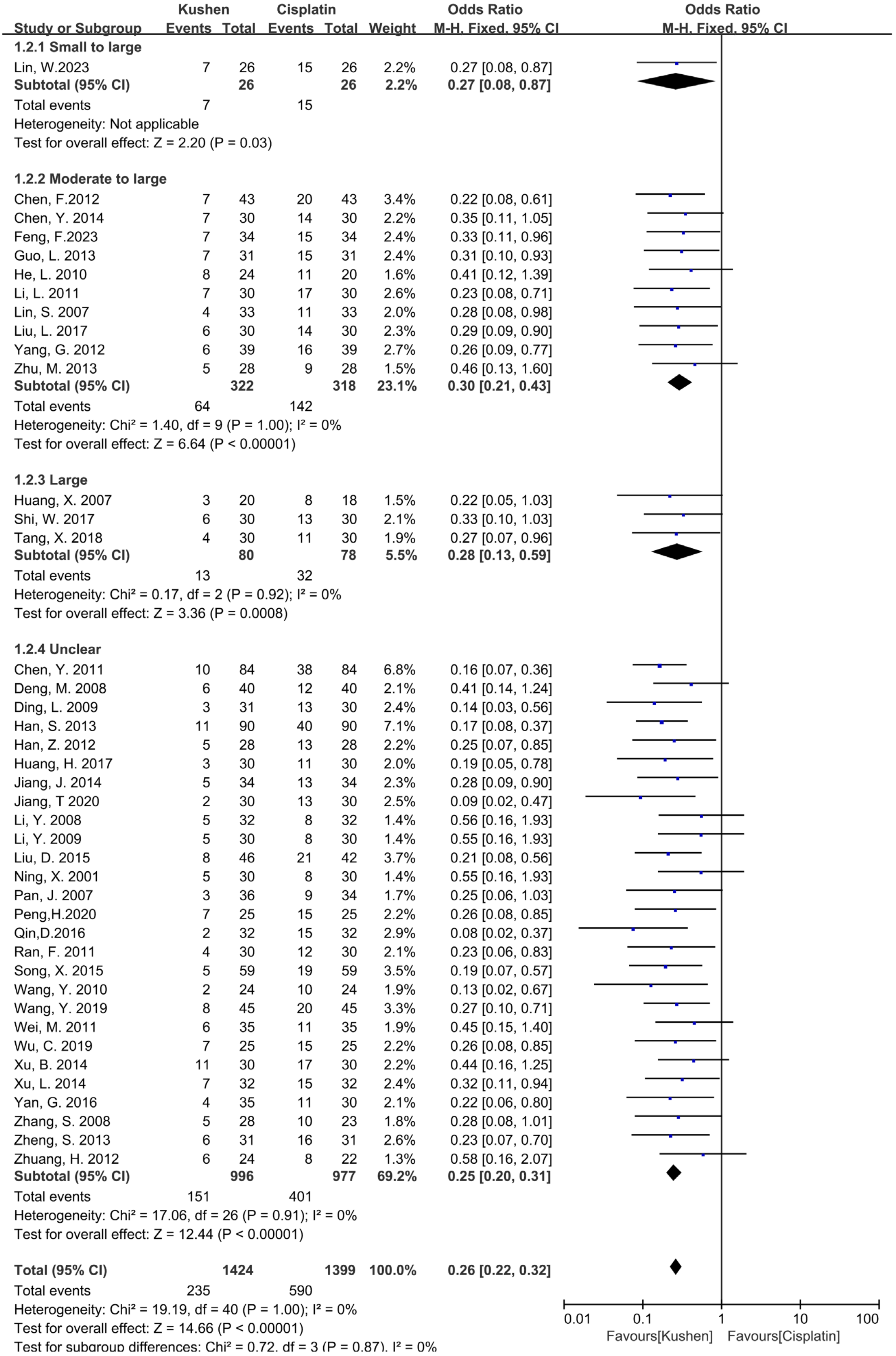


**Figure.S31 Subgroups analysis of pleurodesis failure via pleural effusion**

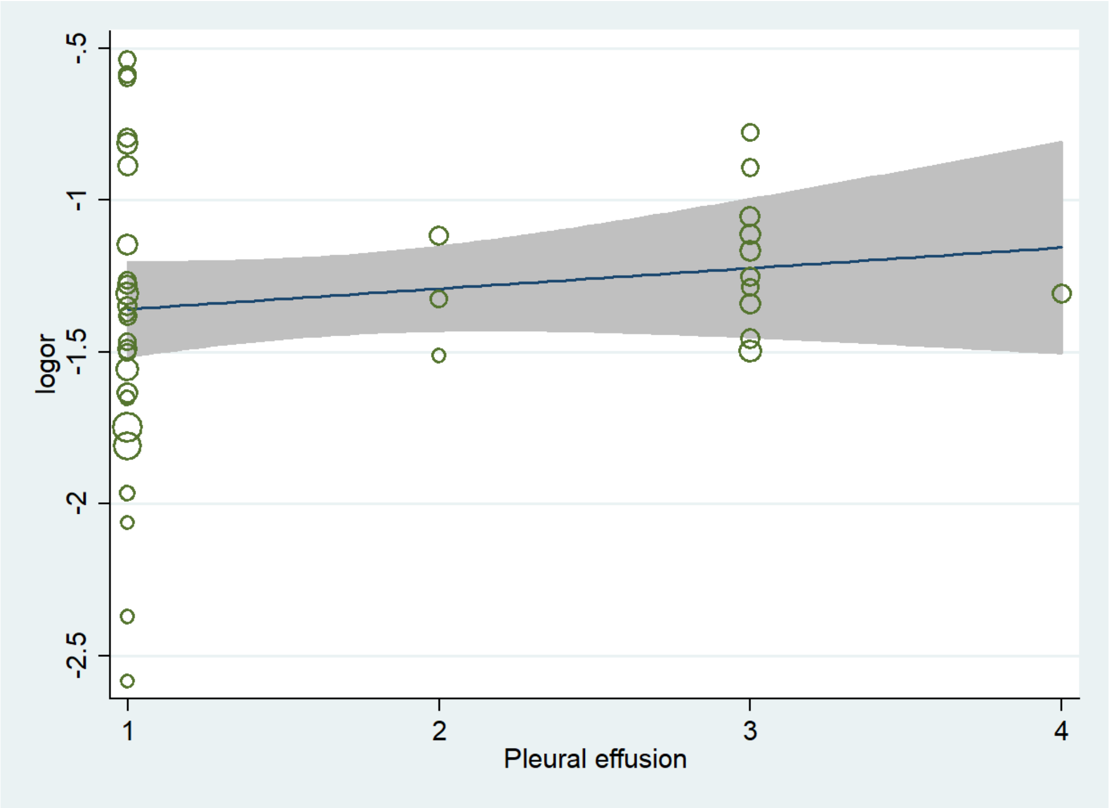


**Figure.S32 Meta-regression of pleurodesis failure via pleural effusion**

| Study or Subgroup | Kushen Events | Kushen Total | Cisplatin Events | Cisplatin Total | Weight | Odds Ratio M-H, Fixed, 95% CI | Year |
|---|---|---|---|---|---|---|---|
| **1.1.1 Karnofsky Performance Status score (≥ 50)** | | | | | | | |
| He, L. 2010 | 7 | 24 | 5 | 20 | 2.2% | 1.24 [0.32, 4.72] | 2010 |
| Han, Z. 2012 | 10 | 28 | 5 | 28 | 1.8% | 2.56 [0.74, 8.81] | 2012 |
| Zheng, S. 2013 | 6 | 31 | 3 | 31 | 1.4% | 2.24 [0.51, 9.91] | 2013 |
| Guo, L. 2013 | 14 | 31 | 7 | 31 | 2.2% | 2.82 [0.94, 8.48] | 2013 |
| Liu, L. 2017 | 13 | 30 | 7 | 30 | 2.3% | 2.51 [0.83, 7.64] | 2017 |
| Shi, W. 2017 | 14 | 30 | 8 | 30 | 2.4% | 2.41 [0.82, 7.10] | 2017 |
| Tang, X. 2018 | 15 | 30 | 10 | 30 | 2.9% | 2.00 [0.70, 5.68] | 2018 |
| **Subtotal (95% CI)** | | **204** | | **200** | **15.2%** | **2.24 [1.44, 3.49]** | |
| Total events | 79 | | 45 | | | | |
| Heterogeneity: $Chi^2$ = 1.07, df = 6 (P = 0.98); $I^2$ = 0% | | | | | | | |
| Test for overall effect: Z = 3.57 (P = 0.0004) | | | | | | | |
| **1.1.2 Karnofsky Performance Status score (≥ 60)** | | | | | | | |
| Pan, J. 2007 | 15 | 36 | 12 | 34 | 4.1% | 1.31 [0.50, 3.44] | 2007 |
| Zhang, S. 2008 | 8 | 28 | 5 | 23 | 2.2% | 1.44 [0.40, 5.21] | 2008 |
| Ding, L. 2009 | 14 | 31 | 9 | 30 | 2.9% | 1.92 [0.67, 5.51] | 2009 |
| Wang, Y. 2010 | 6 | 24 | 2 | 24 | 0.9% | 3.67 [0.66, 20.42] | 2010 |
| Chen, Y. 2011 | 54 | 84 | 22 | 84 | 4.5% | 5.07 [2.62, 9.82] | 2011 |
| Ran, F. 2011 | 12 | 30 | 10 | 30 | 3.4% | 1.33 [0.46, 3.82] | 2011 |
| Yang, G. 2012 | 14 | 39 | 11 | 39 | 4.0% | 1.43 [0.55, 3.71] | 2012 |
| Chen, F.2012 | 16 | 43 | 9 | 43 | 3.2% | 2.24 [0.86, 5.85] | 2012 |
| Han, S. 2013 | 35 | 90 | 13 | 90 | 4.5% | 3.77 [1.83, 7.78] | 2013 |
| Xu, B. 2014 | 12 | 30 | 3 | 30 | 1.0% | 6.00 [1.48, 24.30] | 2014 |
| Liu, D. 2015 | 20 | 46 | 6 | 42 | 2.0% | 4.62 [1.63, 13.09] | 2015 |
| Yan, G. 2016 | 22 | 35 | 12 | 30 | 2.7% | 2.54 [0.93, 6.91] | 2016 |
| Huang, H. 2017 | 20 | 30 | 11 | 30 | 2.1% | 3.45 [1.19, 9.99] | 2017 |
| Lin, W.2023 | 8 | 26 | 5 | 26 | 2.0% | 1.87 [0.52, 6.73] | 2023 |
| Feng, F.2023 | 11 | 34 | 7 | 34 | 2.7% | 1.84 [0.61, 5.54] | 2023 |
| **Subtotal (95% CI)** | | **606** | | **589** | **42.4%** | **2.67 [2.07, 3.44]** | |
| Total events | 267 | | 137 | | | | |
| Heterogeneity: $Chi^2$ = 14.75, df = 14 (P = 0.40); $I^2$ = 5% | | | | | | | |
| Test for overall effect: Z = 7.56 (P < 0.00001) | | | | | | | |
| **1.1.3 Karnofsky Performance Status score (≥ 70)** | | | | | | | |
| Zhu, M. 2013 | 9 | 28 | 6 | 28 | 2.3% | 1.74 [0.52, 5.78] | 2013 |
| Song, X. 2015 | 47 | 59 | 30 | 59 | 3.5% | 3.79 [1.68, 8.54] | 2015 |
| **Subtotal (95% CI)** | | **87** | | **87** | **5.8%** | **2.97 [1.52, 5.79]** | |
| Total events | 56 | | 36 | | | | |
| Heterogeneity: $Chi^2$ = 1.11, df = 1 (P = 0.29); $I^2$ = 10% | | | | | | | |
| Test for overall effect: Z = 3.19 (P = 0.001) | | | | | | | |
| **1.1.4 Unclear** | | | | | | | |
| Ning, X. 2001 | 17 | 30 | 8 | 30 | 2.0% | 3.60 [1.22, 10.64] | 2001 |
| Lin, S. 2007 | 20 | 33 | 10 | 33 | 2.3% | 3.54 [1.28, 9.80] | 2007 |
| Huang, X. 2007 | 9 | 20 | 4 | 18 | 1.3% | 2.86 [0.69, 11.82] | 2007 |
| Deng, M. 2008 | 24 | 40 | 10 | 40 | 2.3% | 4.50 [1.73, 11.70] | 2008 |
| Li, Y. 2008 | 17 | 32 | 8 | 32 | 2.1% | 3.40 [1.18, 9.81] | 2008 |
| Li, Y. 2009 | 17 | 30 | 8 | 30 | 2.0% | 3.60 [1.22, 10.64] | 2009 |
| Wei, M. 2011 | 13 | 35 | 10 | 35 | 3.6% | 1.48 [0.54, 4.03] | 2011 |
| Li, L. 2011 | 6 | 30 | 3 | 30 | 1.4% | 2.25 [0.51, 9.99] | 2011 |
| Zhuang, H. 2012 | 7 | 24 | 6 | 22 | 2.5% | 1.10 [0.30, 3.98] | 2012 |
| Chen, Y. 2014 | 13 | 30 | 7 | 30 | 2.3% | 2.51 [0.83, 7.64] | 2014 |
| Jiang, J. 2014 | 11 | 34 | 7 | 34 | 2.7% | 1.84 [0.61, 5.54] | 2014 |
| Xu, L. 2014 | 18 | 32 | 13 | 32 | 3.3% | 1.88 [0.70, 5.07] | 2014 |
| Qin,D.2016 | 11 | 32 | 6 | 32 | 2.3% | 2.27 [0.72, 7.16] | 2016 |
| Wang, Y. 2019 | 20 | 45 | 9 | 45 | 2.9% | 3.20 [1.25, 8.17] | 2019 |
| Wu, C. 2019 | 8 | 25 | 4 | 25 | 1.6% | 2.47 [0.63, 9.63] | 2019 |
| Peng,H.2020 | 12 | 25 | 5 | 25 | 1.5% | 3.69 [1.05, 12.96] | 2020 |
| Jiang, T 2020 | 24 | 30 | 6 | 30 | 0.7% | 16.00 [4.52, 56.70] | 2020 |
| **Subtotal (95% CI)** | | **527** | | **523** | **36.6%** | **2.91 [2.22, 3.80]** | |
| Total events | 247 | | 124 | | | | |
| Heterogeneity: $Chi^2$ = 14.25, df = 16 (P = 0.58); $I^2$ = 0% | | | | | | | |
| Test for overall effect: Z = 7.81 (P < 0.00001) | | | | | | | |
| **Total (95% CI)** | | **1424** | | **1399** | **100.0%** | **2.71 [2.30, 3.19]** | |
| Total events | 649 | | 342 | | | | |
| Heterogeneity: $Chi^2$ = 32.18, df = 40 (P = 0.81); $I^2$ = 0% | | | | | | | |
| Test for overall effect: Z = 11.84 (P < 0.00001) | | | | | | | |
| Test for subgroup differences: $Chi^2$ = 1.06, df = 3 (P = 0.79), $I^2$ = 0% | | | | | | | |

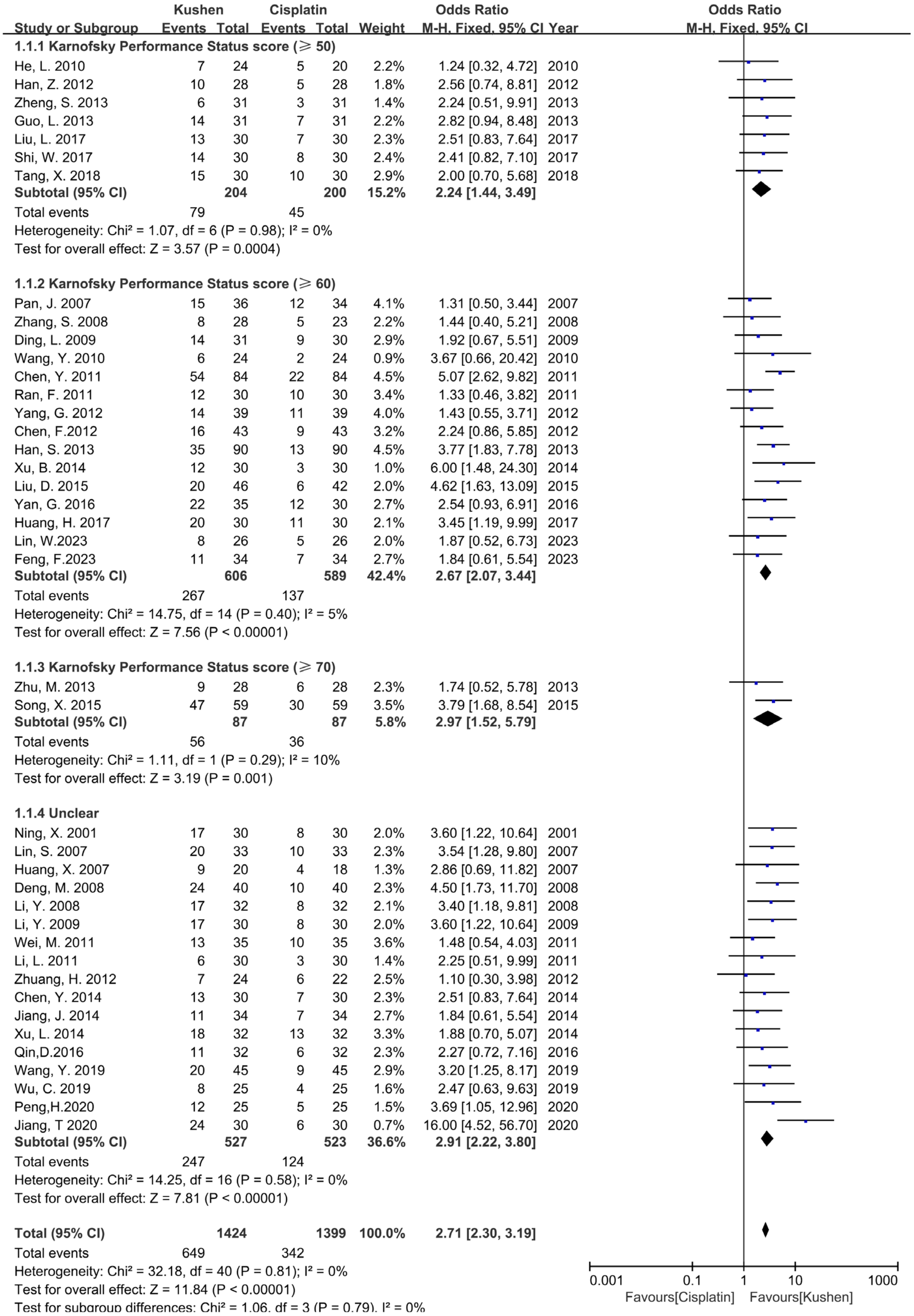


**Figure.S33 Subgroups analysis of complete response via KPS score**

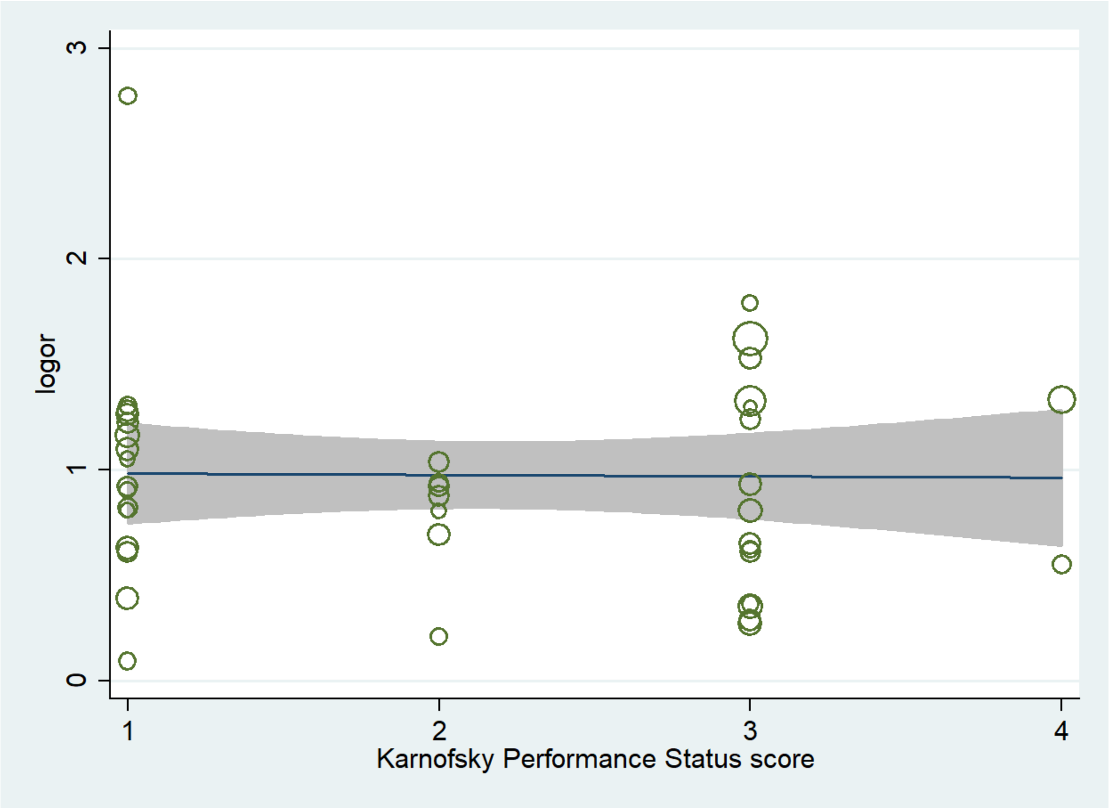


**Figure.S34 Meta-regression of complete response via KPS score**

| Study or Subgroup | Kushen Events | Kushen Total | Cisplatin Events | Cisplatin Total | Weight | Odds Ratio M-H, Fixed, 95% CI | Year |
|---|---|---|---|---|---|---|---|
| **1.2.1 Karnofsky Performance Status score (⩾ 50)** | | | | | | | |
| He, L. 2010 | 8 | 24 | 11 | 20 | 1.6% | 0.41 [0.12, 1.39] | 2010 |
| Han, Z. 2012 | 5 | 28 | 13 | 28 | 2.2% | 0.25 [0.07, 0.85] | 2012 |
| Zheng, S. 2013 | 6 | 31 | 16 | 31 | 2.6% | 0.23 [0.07, 0.70] | 2013 |
| Guo, L. 2013 | 7 | 31 | 15 | 31 | 2.4% | 0.31 [0.10, 0.93] | 2013 |
| Liu, L. 2017 | 6 | 30 | 14 | 30 | 2.3% | 0.29 [0.09, 0.90] | 2017 |
| Shi, W. 2017 | 6 | 30 | 13 | 30 | 2.1% | 0.33 [0.10, 1.03] | 2017 |
| Tang, X. 2018 | 4 | 30 | 11 | 30 | 1.9% | 0.27 [0.07, 0.96] | 2018 |
| **Subtotal (95% CI)** | | **204** | | **200** | **15.1%** | **0.29 [0.19, 0.45]** | |
| Total events | 42 | | 93 | | | | |
| Heterogeneity: Chi² = 0.63, df = 6 (P = 1.00); I² = 0% | | | | | | | |
| Test for overall effect: Z = 5.46 (P < 0.00001) | | | | | | | |
| **1.2.2 Karnofsky Performance Status score (⩾ 60)** | | | | | | | |
| Pan, J. 2007 | 3 | 36 | 9 | 34 | 1.7% | 0.25 [0.06, 1.03] | 2007 |
| Zhang, S. 2008 | 5 | 28 | 10 | 23 | 1.8% | 0.28 [0.08, 1.01] | 2008 |
| Ding, L. 2009 | 3 | 31 | 13 | 30 | 2.4% | 0.14 [0.03, 0.56] | 2009 |
| Wang, Y. 2010 | 2 | 24 | 10 | 24 | 1.9% | 0.13 [0.02, 0.67] | 2010 |
| Chen, Y. 2011 | 10 | 84 | 38 | 84 | 6.8% | 0.16 [0.07, 0.36] | 2011 |
| Ran, F. 2011 | 4 | 30 | 12 | 30 | 2.1% | 0.23 [0.06, 0.83] | 2011 |
| Yang, G. 2012 | 6 | 39 | 16 | 39 | 2.7% | 0.26 [0.09, 0.77] | 2012 |
| Chen, F.2012 | 7 | 43 | 20 | 43 | 3.4% | 0.22 [0.08, 0.61] | 2012 |
| Han, S. 2013 | 11 | 90 | 40 | 90 | 7.1% | 0.17 [0.08, 0.37] | 2013 |
| Xu, B. 2014 | 11 | 30 | 17 | 30 | 2.2% | 0.44 [0.16, 1.25] | 2014 |
| Liu, D. 2015 | 8 | 46 | 21 | 42 | 3.7% | 0.21 [0.08, 0.56] | 2015 |
| Yan, G. 2016 | 4 | 35 | 11 | 30 | 2.1% | 0.22 [0.06, 0.80] | 2016 |
| Huang, H. 2017 | 3 | 30 | 11 | 30 | 2.0% | 0.19 [0.05, 0.78] | 2017 |
| Lin, W.2023 | 7 | 26 | 15 | 26 | 2.2% | 0.27 [0.08, 0.87] | 2023 |
| Feng, F.2023 | 7 | 34 | 15 | 34 | 2.4% | 0.33 [0.11, 0.96] | 2023 |
| **Subtotal (95% CI)** | | **606** | | **589** | **44.7%** | **0.22 [0.17, 0.29]** | |
| Total events | 91 | | 258 | | | | |
| Heterogeneity: Chi² = 4.47, df = 14 (P = 0.99); I² = 0% | | | | | | | |
| Test for overall effect: Z = 10.57 (P < 0.00001) | | | | | | | |
| **1.2.3 Karnofsky Performance Status score (⩾ 70)** | | | | | | | |
| Zhu, M. 2013 | 5 | 28 | 9 | 28 | 1.5% | 0.46 [0.13, 1.60] | 2013 |
| Song, X. 2015 | 5 | 59 | 19 | 59 | 3.5% | 0.19 [0.07, 0.57] | 2015 |
| **Subtotal (95% CI)** | | **87** | | **87** | **5.0%** | **0.27 [0.12, 0.61]** | |
| Total events | 10 | | 28 | | | | |
| Heterogeneity: Chi² = 1.05, df = 1 (P = 0.31); I² = 4% | | | | | | | |
| Test for overall effect: Z = 3.18 (P = 0.001) | | | | | | | |
| **1.2.4 Unclear** | | | | | | | |
| Ning, X. 2001 | 5 | 30 | 8 | 30 | 1.4% | 0.55 [0.16, 1.93] | 2001 |
| Lin, S. 2007 | 4 | 33 | 11 | 33 | 2.0% | 0.28 [0.08, 0.98] | 2007 |
| Huang, X. 2007 | 3 | 20 | 8 | 18 | 1.5% | 0.22 [0.05, 1.03] | 2007 |
| Li, Y. 2008 | 5 | 32 | 8 | 32 | 1.4% | 0.56 [0.16, 1.93] | 2008 |
| Deng, M. 2008 | 6 | 40 | 12 | 40 | 2.1% | 0.41 [0.14, 1.24] | 2008 |
| Li, Y. 2009 | 5 | 30 | 8 | 30 | 1.4% | 0.55 [0.16, 1.93] | 2009 |
| Wei, M. 2011 | 6 | 35 | 11 | 35 | 1.9% | 0.45 [0.15, 1.40] | 2011 |
| Li, L. 2011 | 7 | 30 | 17 | 30 | 2.6% | 0.23 [0.08, 0.71] | 2011 |
| Zhuang, H. 2012 | 6 | 24 | 8 | 22 | 1.3% | 0.58 [0.16, 2.07] | 2012 |
| Jiang, J. 2014 | 5 | 34 | 13 | 34 | 2.3% | 0.28 [0.09, 0.90] | 2014 |
| Chen, Y. 2014 | 7 | 30 | 14 | 30 | 2.2% | 0.35 [0.11, 1.05] | 2014 |
| Xu, L. 2014 | 7 | 32 | 15 | 32 | 2.4% | 0.32 [0.11, 0.94] | 2014 |
| Qin,D.2016 | 2 | 32 | 15 | 32 | 2.9% | 0.08 [0.02, 0.37] | 2016 |
| Wang, Y. 2019 | 8 | 45 | 20 | 45 | 3.3% | 0.27 [0.10, 0.71] | 2019 |
| Wu, C. 2019 | 7 | 25 | 15 | 25 | 2.2% | 0.26 [0.08, 0.85] | 2019 |
| Peng,H.2020 | 7 | 25 | 15 | 25 | 2.2% | 0.26 [0.08, 0.85] | 2020 |
| Jiang, T 2020 | 2 | 30 | 13 | 30 | 2.5% | 0.09 [0.02, 0.47] | 2020 |
| **Subtotal (95% CI)** | | **527** | | **523** | **35.2%** | **0.31 [0.23, 0.41]** | |
| Total events | 92 | | 211 | | | | |
| Heterogeneity: Chi² = 10.07, df = 16 (P = 0.86); I² = 0% | | | | | | | |
| Test for overall effect: Z = 8.05 (P < 0.00001) | | | | | | | |
| **Total (95% CI)** | | **1424** | | **1399** | **100.0%** | **0.26 [0.22, 0.32]** | |
| Total events | 235 | | 590 | | | | |
| Heterogeneity: Chi² = 19.19, df = 40 (P = 1.00); I² = 0% | | | | | | | |
| Test for overall effect: Z = 14.66 (P < 0.00001) | | | | | | | |
| Test for subgroup differences: Chi² = 2.81, df = 3 (P = 0.42), I² = 0% | | | | | | | |

Odds Ratio
M-H, Fixed, 95% CI

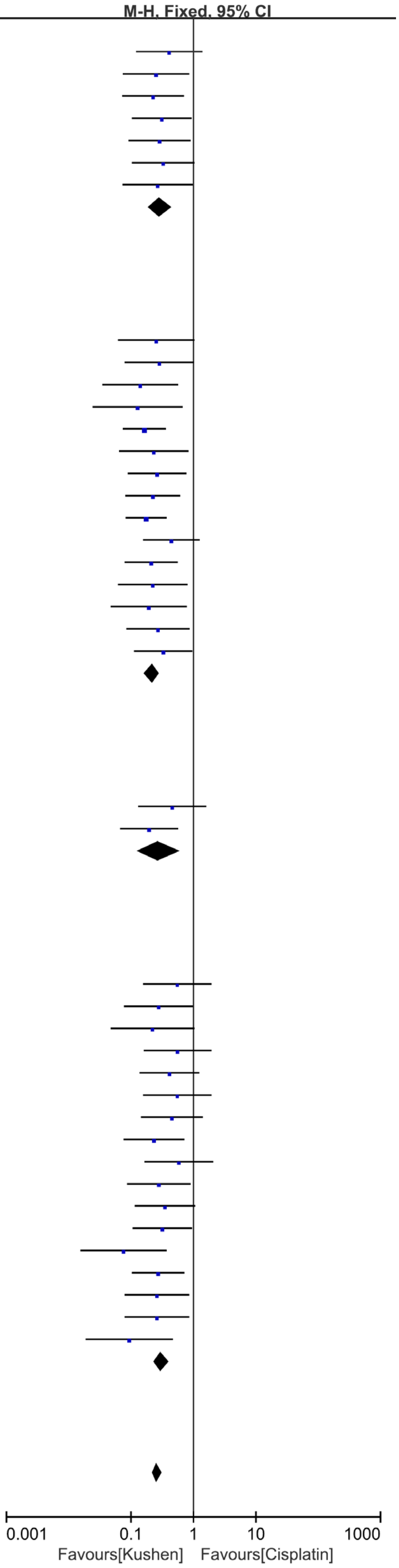


**Figure.S35 Subgroups analysis of pleurodesis failure via KPS score**

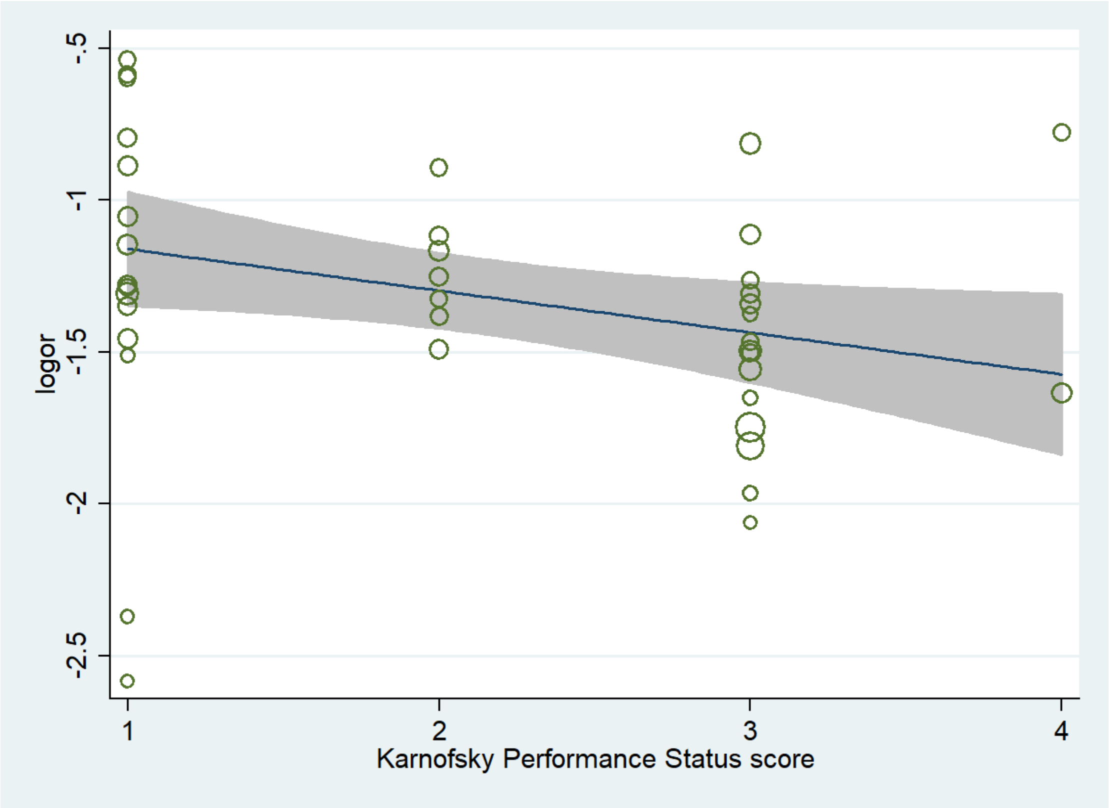


**Figure.S36 Meta-regression of pleurodesis failure via KPS score**

| Study or Subgroup | Kushen Events | Kushen Total | Cisplatin Events | Cisplatin Total | Weight | Odds Ratio M-H, Fixed, 95% CI | Year |
|---|---|---|---|---|---|---|---|
| **1.1.1 Anticipated survival time(Unclear)** | | | | | | | |
| Ning, X. 2001 | 17 | 30 | 8 | 30 | 2.0% | 3.60 [1.22, 10.64] | 2001 |
| Lin, S. 2007 | 20 | 33 | 10 | 33 | 2.3% | 3.54 [1.28, 9.80] | 2007 |
| Pan, J. 2007 | 15 | 36 | 12 | 34 | 4.1% | 1.31 [0.50, 3.44] | 2007 |
| Zhang, S. 2008 | 8 | 28 | 5 | 23 | 2.2% | 1.44 [0.40, 5.21] | 2008 |
| Deng, M. 2008 | 24 | 40 | 10 | 40 | 2.3% | 4.50 [1.73, 11.70] | 2008 |
| Li, Y. 2008 | 17 | 32 | 8 | 32 | 2.1% | 3.40 [1.18, 9.81] | 2008 |
| Chen, Y. 2011 | 54 | 84 | 22 | 84 | 4.5% | 5.07 [2.62, 9.82] | 2011 |
| Wei, M. 2011 | 13 | 35 | 10 | 35 | 3.6% | 1.48 [0.54, 4.03] | 2011 |
| Li, L. 2011 | 6 | 30 | 3 | 30 | 1.4% | 2.25 [0.51, 9.99] | 2011 |
| Chen, F.2012 | 16 | 43 | 9 | 43 | 3.2% | 2.24 [0.86, 5.85] | 2012 |
| Zhuang, H. 2012 | 7 | 24 | 6 | 22 | 2.5% | 1.10 [0.30, 3.98] | 2012 |
| Guo, L. 2013 | 14 | 31 | 7 | 31 | 2.2% | 2.82 [0.94, 8.48] | 2013 |
| Zhu, M. 2013 | 9 | 28 | 6 | 28 | 2.3% | 1.74 [0.52, 5.78] | 2013 |
| Chen, Y. 2014 | 13 | 30 | 7 | 30 | 2.3% | 2.51 [0.83, 7.64] | 2014 |
| Jiang, J. 2014 | 11 | 34 | 7 | 34 | 2.7% | 1.84 [0.61, 5.54] | 2014 |
| Xu, B. 2014 | 12 | 30 | 3 | 30 | 1.0% | 6.00 [1.48, 24.30] | 2014 |
| Xu, L. 2014 | 18 | 32 | 13 | 32 | 3.3% | 1.88 [0.70, 5.07] | 2014 |
| Song, X. 2015 | 47 | 59 | 30 | 59 | 3.5% | 3.79 [1.68, 8.54] | 2015 |
| Qin,D.2016 | 11 | 32 | 6 | 32 | 2.3% | 2.27 [0.72, 7.16] | 2016 |
| Liu, L. 2017 | 13 | 30 | 7 | 30 | 2.3% | 2.51 [0.83, 7.64] | 2017 |
| Shi, W. 2017 | 14 | 30 | 8 | 30 | 2.4% | 2.41 [0.82, 7.10] | 2017 |
| Tang, X. 2018 | 15 | 30 | 10 | 30 | 2.9% | 2.00 [0.70, 5.68] | 2018 |
| Wang, Y. 2019 | 20 | 45 | 9 | 45 | 2.9% | 3.20 [1.25, 8.17] | 2019 |
| Wu, C. 2019 | 8 | 25 | 4 | 25 | 1.6% | 2.47 [0.63, 9.63] | 2019 |
| Peng,H.2020 | 12 | 25 | 5 | 25 | 1.5% | 3.69 [1.05, 12.96] | 2020 |
| Jiang, T 2020 | 24 | 30 | 6 | 30 | 0.7% | 16.00 [4.52, 56.70] | 2020 |
| **Subtotal (95% CI)** | | **906** | | **897** | **64.0%** | **2.83 [2.31, 3.47]** | |
| Total events | 438 | | 231 | | | | |
| Heterogeneity: Chi² = 23.60, df = 25 (P = 0.54); I² = 0% | | | | | | | |
| Test for overall effect: Z = 10.00 (P < 0.00001) | | | | | | | |
| **1.1.2 Anticipated survival time(⩾ 3 months)** | | | | | | | |
| Ding, L. 2009 | 14 | 31 | 9 | 30 | 2.9% | 1.92 [0.67, 5.51] | 2009 |
| Wang, Y. 2010 | 6 | 24 | 2 | 24 | 0.9% | 3.67 [0.66, 20.42] | 2010 |
| He, L. 2010 | 7 | 24 | 5 | 20 | 2.2% | 1.24 [0.32, 4.72] | 2010 |
| Ran, F. 2011 | 12 | 30 | 10 | 30 | 3.4% | 1.33 [0.46, 3.82] | 2011 |
| Yang, G. 2012 | 14 | 39 | 11 | 39 | 4.0% | 1.43 [0.55, 3.71] | 2012 |
| Han, Z. 2012 | 10 | 28 | 5 | 28 | 1.8% | 2.56 [0.74, 8.81] | 2012 |
| Han, S. 2013 | 35 | 90 | 13 | 90 | 4.5% | 3.77 [1.83, 7.78] | 2013 |
| Zheng, S. 2013 | 6 | 31 | 3 | 31 | 1.4% | 2.24 [0.51, 9.91] | 2013 |
| Liu, D. 2015 | 20 | 46 | 6 | 42 | 2.0% | 4.62 [1.63, 13.09] | 2015 |
| Yan, G. 2016 | 22 | 35 | 12 | 30 | 2.7% | 2.54 [0.93, 6.91] | 2016 |
| Huang, H. 2017 | 20 | 30 | 11 | 30 | 2.1% | 3.45 [1.19, 9.99] | 2017 |
| Lin, W.2023 | 8 | 26 | 5 | 26 | 2.0% | 1.87 [0.52, 6.73] | 2023 |
| Feng, F.2023 | 11 | 34 | 7 | 34 | 2.7% | 1.84 [0.61, 5.54] | 2023 |
| **Subtotal (95% CI)** | | **468** | | **454** | **32.7%** | **2.41 [1.79, 3.25]** | |
| Total events | 185 | | 99 | | | | |
| Heterogeneity: Chi² = 7.54, df = 12 (P = 0.82); I² = 0% | | | | | | | |
| Test for overall effect: Z = 5.81 (P < 0.00001) | | | | | | | |
| **1.1.3 Anticipated survival time (⩾ 1 months)** | | | | | | | |
| Huang, X. 2007 | 9 | 20 | 4 | 18 | 1.3% | 2.86 [0.69, 11.82] | 2007 |
| Li, Y. 2009 | 17 | 30 | 8 | 30 | 2.0% | 3.60 [1.22, 10.64] | 2009 |
| **Subtotal (95% CI)** | | **50** | | **48** | **3.3%** | **3.30 [1.40, 7.82]** | |
| Total events | 26 | | 12 | | | | |
| Heterogeneity: Chi² = 0.06, df = 1 (P = 0.80); I² = 0% | | | | | | | |
| Test for overall effect: Z = 2.72 (P = 0.007) | | | | | | | |
| **Total (95% CI)** | | **1424** | | **1399** | **100.0%** | **2.71 [2.30, 3.19]** | |
| Total events | 649 | | 342 | | | | |
| Heterogeneity: Chi² = 32.18, df = 40 (P = 0.81); I² = 0% | | | | | | | |
| Test for overall effect: Z = 11.84 (P < 0.00001) | | | | | | | |
| Test for subgroup differences: Chi² = 0.97, df = 2 (P = 0.62), I² = 0% | | | | | | | |

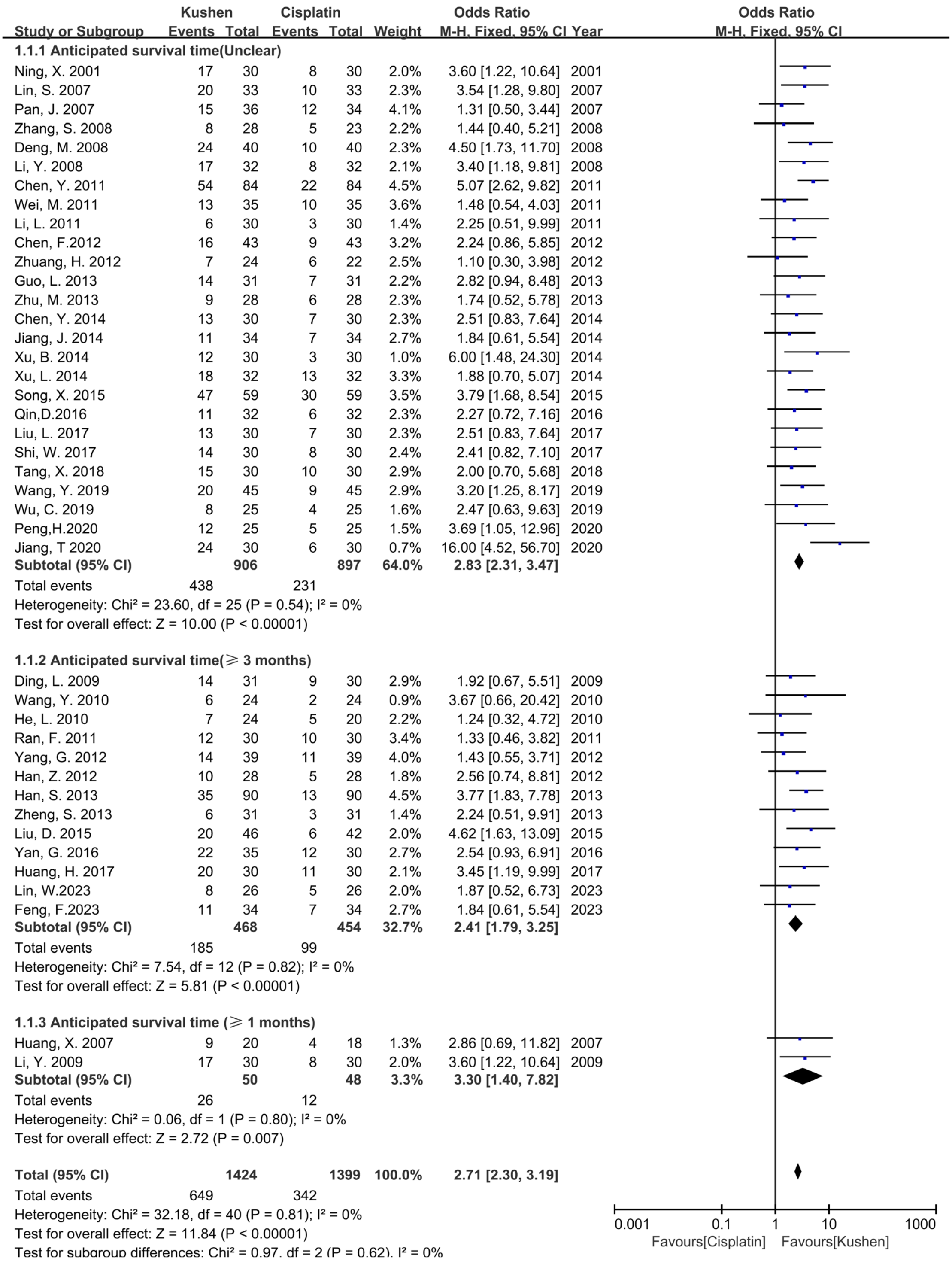


**Figure.S37 Subgroups analysis of complete response via anticipated survival time**

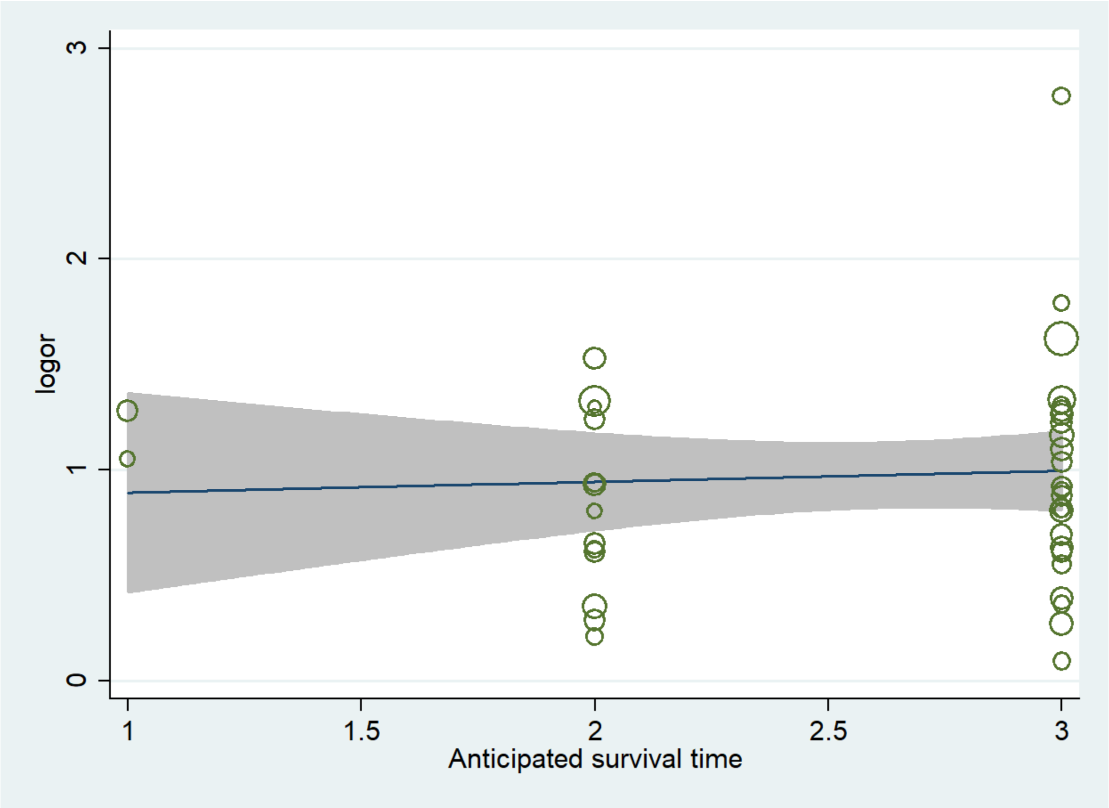


**Figure.S38 Meta-regression of complete response via anticipated survival time**

| Study or Subgroup | Kushen Events | Kushen Total | Cisplatin Events | Cisplatin Total | Weight | Odds Ratio M-H, Fixed, 95% CI | Year |
|---|---|---|---|---|---|---|---|
| **1.2.1 Anticipated survival time(Unclear)** | | | | | | | |
| Ning, X. 2001 | 5 | 30 | 8 | 30 | 1.4% | 0.55 [0.16, 1.93] | 2001 |
| Pan, J. 2007 | 3 | 36 | 9 | 34 | 1.7% | 0.25 [0.06, 1.03] | 2007 |
| Lin, S. 2007 | 4 | 33 | 11 | 33 | 2.0% | 0.28 [0.08, 0.98] | 2007 |
| Li, Y. 2008 | 5 | 32 | 8 | 32 | 1.4% | 0.56 [0.16, 1.93] | 2008 |
| Zhang, S. 2008 | 5 | 28 | 10 | 23 | 1.8% | 0.28 [0.08, 1.01] | 2008 |
| Deng, M. 2008 | 6 | 40 | 12 | 40 | 2.1% | 0.41 [0.14, 1.24] | 2008 |
| Chen, Y. 2011 | 10 | 84 | 38 | 84 | 6.8% | 0.16 [0.07, 0.36] | 2011 |
| Wei, M. 2011 | 6 | 35 | 11 | 35 | 1.9% | 0.45 [0.15, 1.40] | 2011 |
| Li, L. 2011 | 7 | 30 | 17 | 30 | 2.6% | 0.23 [0.08, 0.71] | 2011 |
| Chen, F.2012 | 7 | 43 | 20 | 43 | 3.4% | 0.22 [0.08, 0.61] | 2012 |
| Zhuang, H. 2012 | 6 | 24 | 8 | 22 | 1.3% | 0.58 [0.16, 2.07] | 2012 |
| Zhu, M. 2013 | 5 | 28 | 9 | 28 | 1.5% | 0.46 [0.13, 1.60] | 2013 |
| Guo, L. 2013 | 7 | 31 | 15 | 31 | 2.4% | 0.31 [0.10, 0.93] | 2013 |
| Jiang, J. 2014 | 5 | 34 | 13 | 34 | 2.3% | 0.28 [0.09, 0.90] | 2014 |
| Chen, Y. 2014 | 7 | 30 | 14 | 30 | 2.2% | 0.35 [0.11, 1.05] | 2014 |
| Xu, L. 2014 | 7 | 32 | 15 | 32 | 2.4% | 0.32 [0.11, 0.94] | 2014 |
| Xu, B. 2014 | 11 | 30 | 17 | 30 | 2.2% | 0.44 [0.16, 1.25] | 2014 |
| Song, X. 2015 | 5 | 59 | 19 | 59 | 3.5% | 0.19 [0.07, 0.57] | 2015 |
| Qin,D.2016 | 2 | 32 | 15 | 32 | 2.9% | 0.08 [0.02, 0.37] | 2016 |
| Liu, L. 2017 | 6 | 30 | 14 | 30 | 2.3% | 0.29 [0.09, 0.90] | 2017 |
| Shi, W. 2017 | 6 | 30 | 13 | 30 | 2.1% | 0.33 [0.10, 1.03] | 2017 |
| Tang, X. 2018 | 4 | 30 | 11 | 30 | 1.9% | 0.27 [0.07, 0.96] | 2018 |
| Wang, Y. 2019 | 8 | 45 | 20 | 45 | 3.3% | 0.27 [0.10, 0.71] | 2019 |
| Wu, C. 2019 | 7 | 25 | 15 | 25 | 2.2% | 0.26 [0.08, 0.85] | 2019 |
| Peng,H.2020 | 7 | 25 | 15 | 25 | 2.2% | 0.26 [0.08, 0.85] | 2020 |
| Jiang, T 2020 | 2 | 30 | 13 | 30 | 2.5% | 0.09 [0.02, 0.47] | 2020 |
| **Subtotal (95% CI)** | | **906** | | **897** | **62.0%** | **0.28 [0.23, 0.35]** | |
| Total events | 153 | | 370 | | | | |
| Heterogeneity: Chi² = 13.34, df = 25 (P = 0.97); I² = 0% | | | | | | | |
| Test for overall effect: Z = 11.18 (P < 0.00001) | | | | | | | |
| **1.2.2 Anticipated survival time(⩾ 3 months)** | | | | | | | |
| Ding, L. 2009 | 3 | 31 | 13 | 30 | 2.4% | 0.14 [0.03, 0.56] | 2009 |
| Wang, Y. 2010 | 2 | 24 | 10 | 24 | 1.9% | 0.13 [0.02, 0.67] | 2010 |
| He, L. 2010 | 8 | 24 | 11 | 20 | 1.6% | 0.41 [0.12, 1.39] | 2010 |
| Ran, F. 2011 | 4 | 30 | 12 | 30 | 2.1% | 0.23 [0.06, 0.83] | 2011 |
| Han, Z. 2012 | 5 | 28 | 13 | 28 | 2.2% | 0.25 [0.07, 0.85] | 2012 |
| Yang, G. 2012 | 6 | 39 | 16 | 39 | 2.7% | 0.26 [0.09, 0.77] | 2012 |
| Han, S. 2013 | 11 | 90 | 40 | 90 | 7.1% | 0.17 [0.08, 0.37] | 2013 |
| Zheng, S. 2013 | 6 | 31 | 16 | 31 | 2.6% | 0.23 [0.07, 0.70] | 2013 |
| Liu, D. 2015 | 8 | 46 | 21 | 42 | 3.7% | 0.21 [0.08, 0.56] | 2015 |
| Yan, G. 2016 | 4 | 35 | 11 | 30 | 2.1% | 0.22 [0.06, 0.80] | 2016 |
| Huang, H. 2017 | 3 | 30 | 11 | 30 | 2.0% | 0.19 [0.05, 0.78] | 2017 |
| Lin, W.2023 | 7 | 26 | 15 | 26 | 2.2% | 0.27 [0.08, 0.87] | 2023 |
| Feng, F.2023 | 7 | 34 | 15 | 34 | 2.4% | 0.33 [0.11, 0.96] | 2023 |
| **Subtotal (95% CI)** | | **468** | | **454** | **35.2%** | **0.22 [0.16, 0.31]** | |
| Total events | 74 | | 204 | | | | |
| Heterogeneity: Chi² = 3.01, df = 12 (P = 1.00); I² = 0% | | | | | | | |
| Test for overall effect: Z = 9.35 (P < 0.00001) | | | | | | | |
| **1.2.3 Anticipated survival time (⩾ 1 months)** | | | | | | | |
| Huang, X. 2007 | 3 | 20 | 8 | 18 | 1.5% | 0.22 [0.05, 1.03] | 2007 |
| Li, Y. 2009 | 5 | 30 | 8 | 30 | 1.4% | 0.55 [0.16, 1.93] | 2009 |
| **Subtotal (95% CI)** | | **50** | | **48** | **2.8%** | **0.38 [0.15, 0.99]** | |
| Total events | 8 | | 16 | | | | |
| Heterogeneity: Chi² = 0.81, df = 1 (P = 0.37); I² = 0% | | | | | | | |
| Test for overall effect: Z = 1.98 (P = 0.05) | | | | | | | |
| **Total (95% CI)** | | **1424** | | **1399** | **100.0%** | **0.26 [0.22, 0.32]** | |
| Total events | 235 | | 590 | | | | |
| Heterogeneity: Chi² = 19.19, df = 40 (P = 1.00); I² = 0% | | | | | | | |
| Test for overall effect: Z = 14.66 (P < 0.00001) | | | | | | | |
| Test for subgroup differences: Chi² = 1.96, df = 2 (P = 0.37), I² = 0% | | | | | | | |

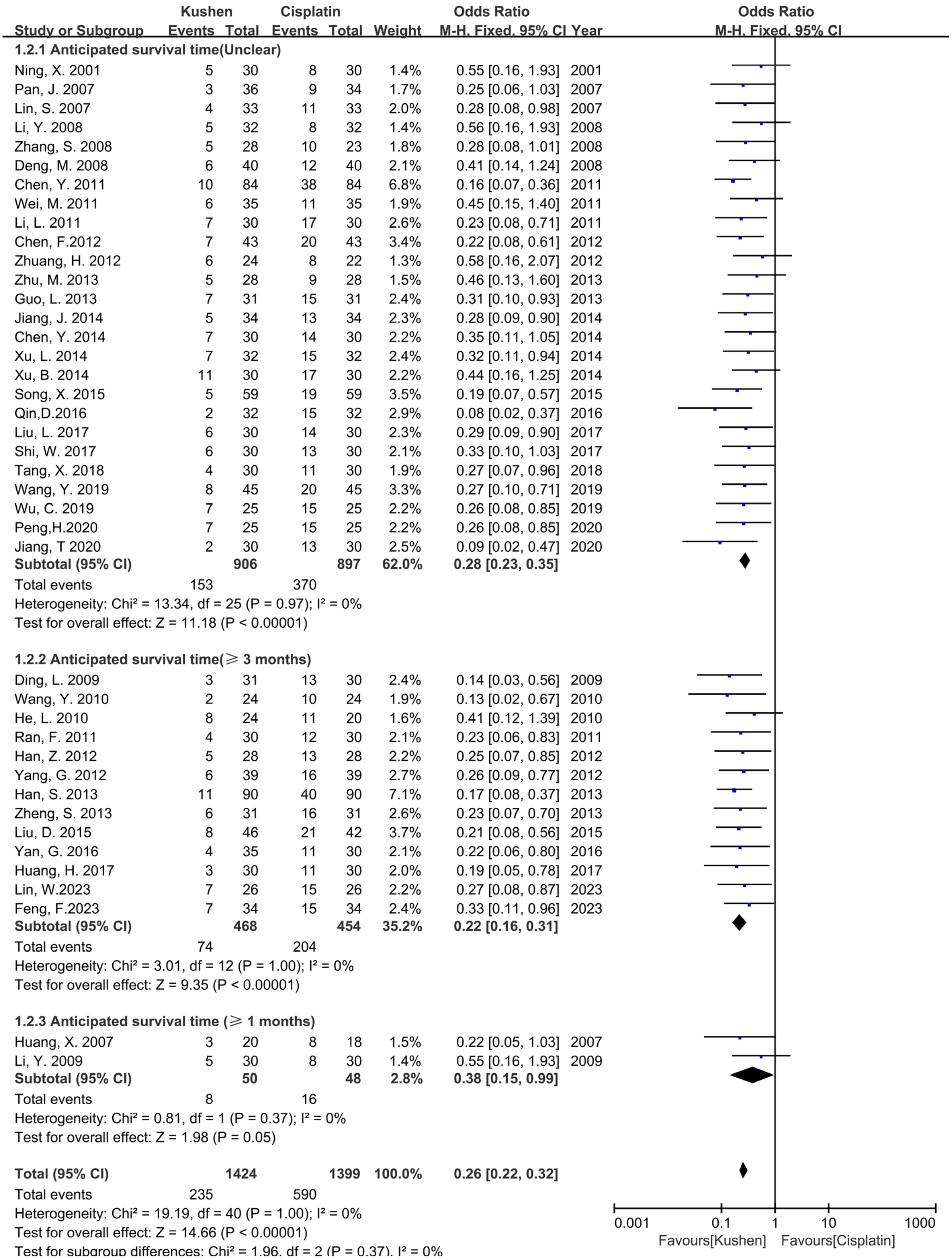


**Figure.S39 Subgroups analysis of pleurodesis failure via anticipated survival time**

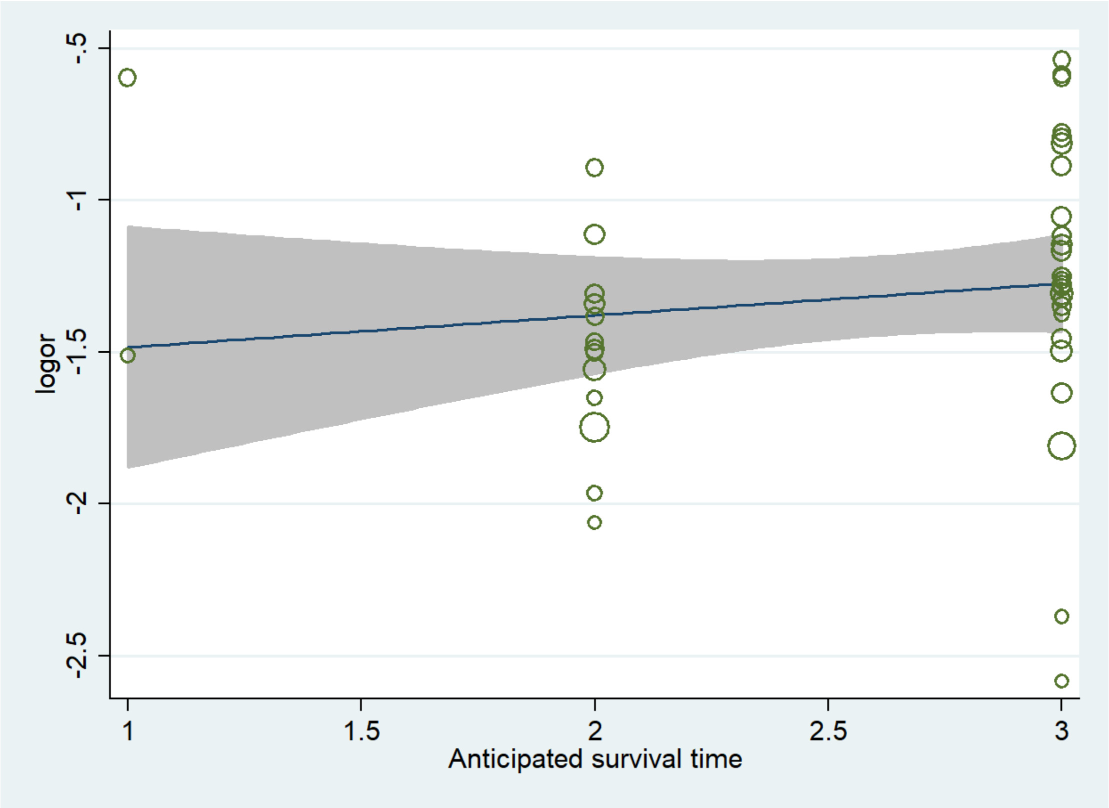


**Figure.S40 Meta-regression of pleurodesis failure via anticipated survival time**

| Study or Subgroup | Kushen Events | Kushen Total | Cisplatin Events | Cisplatin Total | Weight | Odds Ratio M-H, Fixed, 95% CI | Year |
|---|---|---|---|---|---|---|---|
| **1.1.1 Primary treatment** | | | | | | | |
| Zhang, S. 2008 | 8 | 28 | 5 | 23 | 2.2% | 1.44 [0.40, 5.21] | 2008 |
| Han, Z. 2012 | 10 | 28 | 5 | 28 | 1.8% | 2.56 [0.74, 8.81] | 2012 |
| Guo, L. 2013 | 14 | 31 | 7 | 31 | 2.2% | 2.82 [0.94, 8.48] | 2013 |
| Chen, Y. 2014 | 13 | 30 | 7 | 30 | 2.3% | 2.51 [0.83, 7.64] | 2014 |
| Liu, D. 2015 | 20 | 46 | 6 | 42 | 2.0% | 4.62 [1.63, 13.09] | 2015 |
| Liu, L. 2017 | 13 | 30 | 7 | 30 | 2.3% | 2.51 [0.83, 7.64] | 2017 |
| Shi, W. 2017 | 14 | 30 | 8 | 30 | 2.4% | 2.41 [0.82, 7.10] | 2017 |
| Tang, X. 2018 | 15 | 30 | 10 | 30 | 2.9% | 2.00 [0.70, 5.68] | 2018 |
| Lin, W.2023 | 8 | 26 | 5 | 26 | 2.0% | 1.87 [0.52, 6.73] | 2023 |
| **Subtotal (95% CI)** | | **279** | | **270** | **20.1%** | **2.49 [1.71, 3.63]** | |
| Total events | 115 | | 60 | | | | |
| Heterogeneity: Chi² = 2.46, df = 8 (P = 0.96); I² = 0% | | | | | | | |
| Test for overall effect: Z = 4.77 (P < 0.00001) | | | | | | | |
| **1.1.2 Retreatment** | | | | | | | |
| He, L. 2010 | 7 | 24 | 5 | 20 | 2.2% | 1.24 [0.32, 4.72] | 2010 |
| Feng, F.2023 | 11 | 34 | 7 | 34 | 2.7% | 1.84 [0.61, 5.54] | 2023 |
| **Subtotal (95% CI)** | | **58** | | **54** | **4.9%** | **1.57 [0.67, 3.67]** | |
| Total events | 18 | | 12 | | | | |
| Heterogeneity: Chi² = 0.21, df = 1 (P = 0.65); I² = 0% | | | | | | | |
| Test for overall effect: Z = 1.04 (P = 0.30) | | | | | | | |
| **1.1.3 Unclear** | | | | | | | |
| Ning, X. 2001 | 17 | 30 | 8 | 30 | 2.0% | 3.60 [1.22, 10.64] | 2001 |
| Lin, S. 2007 | 20 | 33 | 10 | 33 | 2.3% | 3.54 [1.28, 9.80] | 2007 |
| Pan, J. 2007 | 15 | 36 | 12 | 34 | 4.1% | 1.31 [0.50, 3.44] | 2007 |
| Huang, X. 2007 | 9 | 20 | 4 | 18 | 1.3% | 2.86 [0.69, 11.82] | 2007 |
| Deng, M. 2008 | 24 | 40 | 10 | 40 | 2.3% | 4.50 [1.73, 11.70] | 2008 |
| Li, Y. 2008 | 17 | 32 | 8 | 32 | 2.1% | 3.40 [1.18, 9.81] | 2008 |
| Li, Y. 2009 | 17 | 30 | 8 | 30 | 2.0% | 3.60 [1.22, 10.64] | 2009 |
| Ding, L. 2009 | 14 | 31 | 9 | 30 | 2.9% | 1.92 [0.67, 5.51] | 2009 |
| Wang, Y. 2010 | 6 | 24 | 2 | 24 | 0.9% | 3.67 [0.66, 20.42] | 2010 |
| Chen, Y. 2011 | 54 | 84 | 22 | 84 | 4.5% | 5.07 [2.62, 9.82] | 2011 |
| Ran, F. 2011 | 12 | 30 | 10 | 30 | 3.4% | 1.33 [0.46, 3.82] | 2011 |
| Wei, M. 2011 | 13 | 35 | 10 | 35 | 3.6% | 1.48 [0.54, 4.03] | 2011 |
| Li, L. 2011 | 6 | 30 | 3 | 30 | 1.4% | 2.25 [0.51, 9.99] | 2011 |
| Chen, F.2012 | 16 | 43 | 9 | 43 | 3.2% | 2.24 [0.86, 5.85] | 2012 |
| Yang, G. 2012 | 14 | 39 | 11 | 39 | 4.0% | 1.43 [0.55, 3.71] | 2012 |
| Zhuang, H. 2012 | 7 | 24 | 6 | 22 | 2.5% | 1.10 [0.30, 3.98] | 2012 |
| Han, S. 2013 | 35 | 90 | 13 | 90 | 4.5% | 3.77 [1.83, 7.78] | 2013 |
| Zhu, M. 2013 | 9 | 28 | 6 | 28 | 2.3% | 1.74 [0.52, 5.78] | 2013 |
| Zheng, S. 2013 | 6 | 31 | 3 | 31 | 1.4% | 2.24 [0.51, 9.91] | 2013 |
| Jiang, J. 2014 | 11 | 34 | 7 | 34 | 2.7% | 1.84 [0.61, 5.54] | 2014 |
| Xu, B. 2014 | 12 | 30 | 3 | 30 | 1.0% | 6.00 [1.48, 24.30] | 2014 |
| Xu, L. 2014 | 18 | 32 | 13 | 32 | 3.3% | 1.88 [0.70, 5.07] | 2014 |
| Song, X. 2015 | 47 | 59 | 30 | 59 | 3.5% | 3.79 [1.68, 8.54] | 2015 |
| Yan, G. 2016 | 22 | 35 | 12 | 30 | 2.7% | 2.54 [0.93, 6.91] | 2016 |
| Qin,D.2016 | 11 | 32 | 6 | 32 | 2.3% | 2.27 [0.72, 7.16] | 2016 |
| Huang, H. 2017 | 20 | 30 | 11 | 30 | 2.1% | 3.45 [1.19, 9.99] | 2017 |
| Wang, Y. 2019 | 20 | 45 | 9 | 45 | 2.9% | 3.20 [1.25, 8.17] | 2019 |
| Wu, C. 2019 | 8 | 25 | 4 | 25 | 1.6% | 2.47 [0.63, 9.63] | 2019 |
| Peng,H.2020 | 12 | 25 | 5 | 25 | 1.5% | 3.69 [1.05, 12.96] | 2020 |
| Jiang, T 2020 | 24 | 30 | 6 | 30 | 0.7% | 16.00 [4.52, 56.70] | 2020 |
| **Subtotal (95% CI)** | | **1087** | | **1075** | **75.0%** | **2.84 [2.35, 3.43]** | |
| Total events | 516 | | 270 | | | | |
| Heterogeneity: Chi² = 27.55, df = 29 (P = 0.54); I² = 0% | | | | | | | |
| Test for overall effect: Z = 10.87 (P < 0.00001) | | | | | | | |
| **Total (95% CI)** | | **1424** | | **1399** | **100.0%** | **2.71 [2.30, 3.19]** | |
| Total events | 649 | | 342 | | | | |
| Heterogeneity: Chi² = 32.18, df = 40 (P = 0.81); I² = 0% | | | | | | | |
| Test for overall effect: Z = 11.84 (P < 0.00001) | | | | | | | |
| Test for subgroup differences: Chi² = 2.01, df = 2 (P = 0.37), I² = 0.6% | | | | | | | |

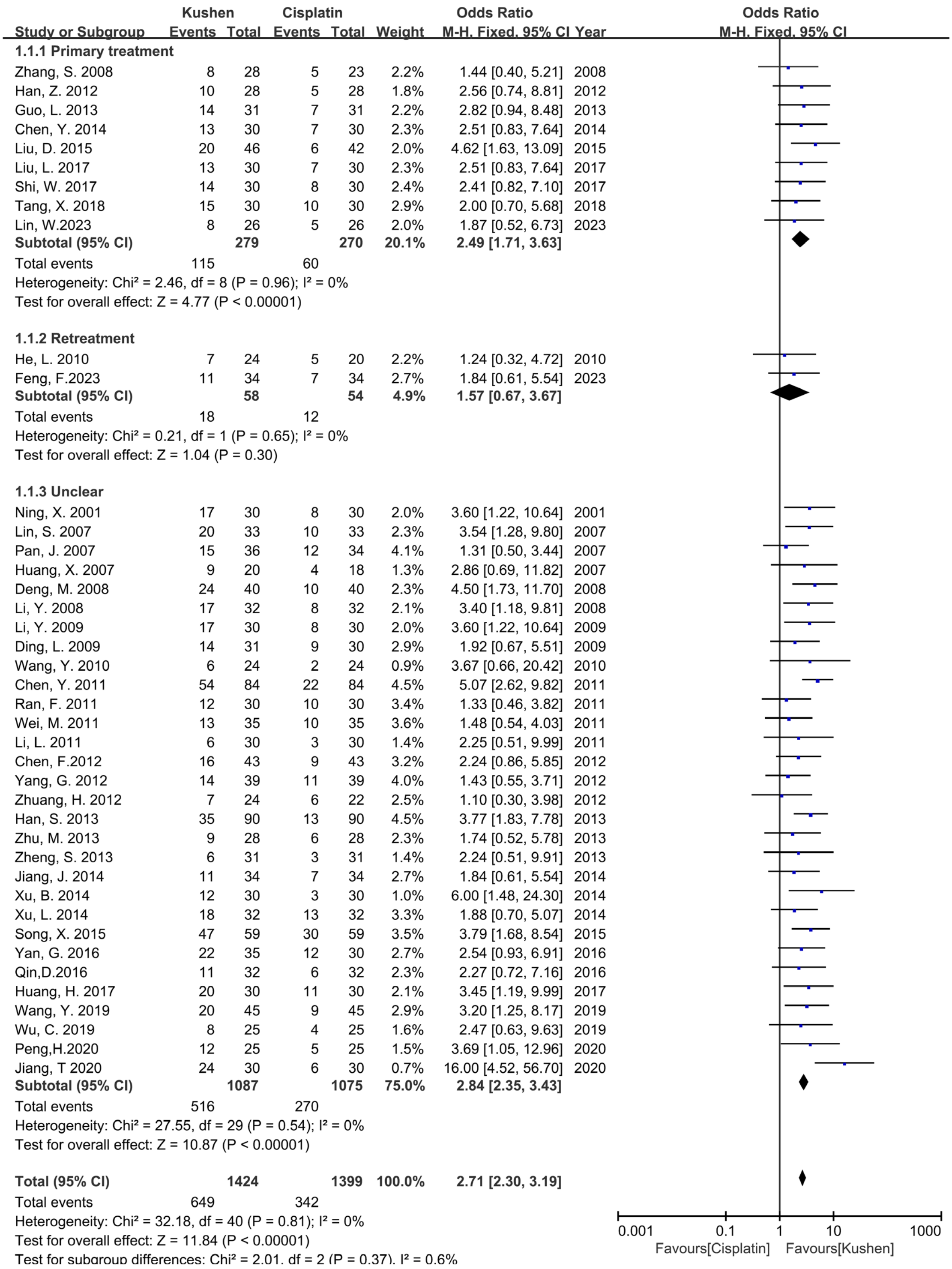


**Figure.S41 Subgroups analysis of complete response via treatment history**

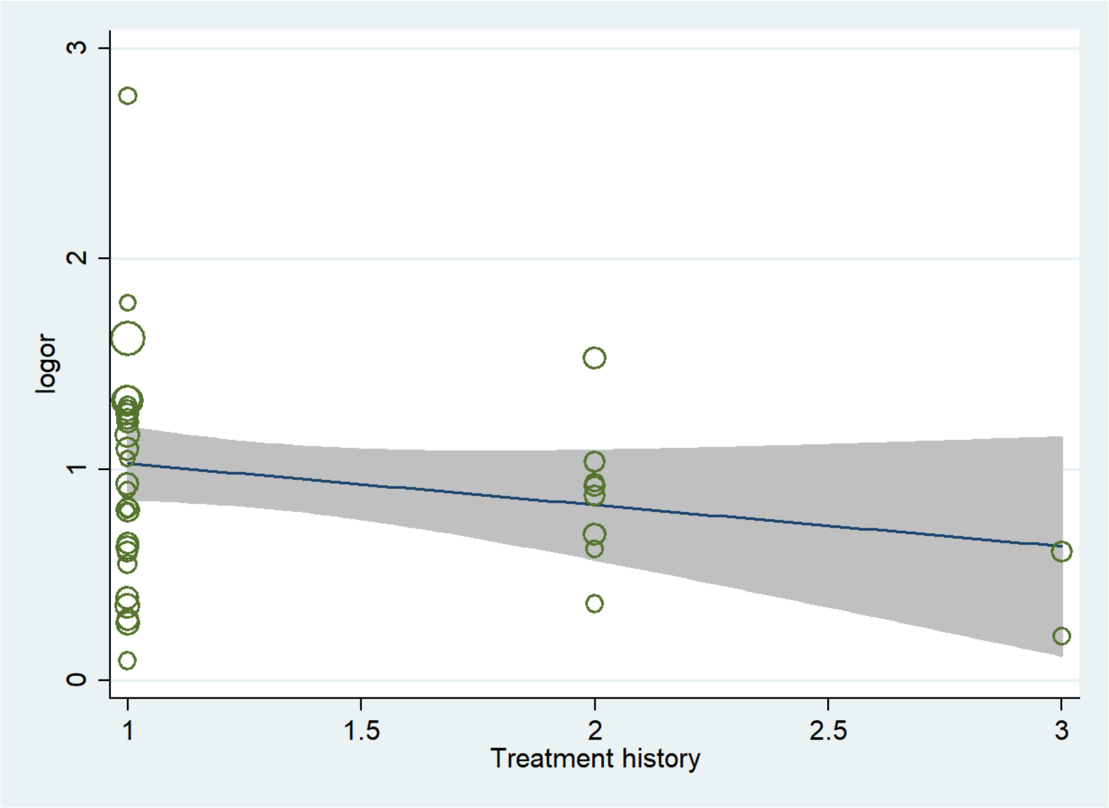


**Figure.S42 Meta-regression of complete response via treatment history**

| Study or Subgroup | Kushen Events | Kushen Total | Cisplatin Events | Cisplatin Total | Weight | Odds Ratio M-H, Fixed, 95% CI | Year |
|---|---|---|---|---|---|---|---|
| **1.2.1 Primary treatment** | | | | | | | |
| Zhang, S. 2008 | 5 | 28 | 10 | 23 | 1.8% | 0.28 [0.08, 1.01] | 2008 |
| Han, Z. 2012 | 5 | 28 | 13 | 28 | 2.2% | 0.25 [0.07, 0.85] | 2012 |
| Guo, L. 2013 | 7 | 31 | 15 | 31 | 2.4% | 0.31 [0.10, 0.93] | 2013 |
| Chen, Y. 2014 | 7 | 30 | 14 | 30 | 2.2% | 0.35 [0.11, 1.05] | 2014 |
| Liu, D. 2015 | 8 | 46 | 21 | 42 | 3.7% | 0.21 [0.08, 0.56] | 2015 |
| Liu, L. 2017 | 6 | 30 | 14 | 30 | 2.3% | 0.29 [0.09, 0.90] | 2017 |
| Shi, W. 2017 | 6 | 30 | 13 | 30 | 2.1% | 0.33 [0.10, 1.03] | 2017 |
| Tang, X. 2018 | 4 | 30 | 11 | 30 | 1.9% | 0.27 [0.07, 0.96] | 2018 |
| Lin, W.2023 | 7 | 26 | 15 | 26 | 2.2% | 0.27 [0.08, 0.87] | 2023 |
| **Subtotal (95% CI)** | | **279** | | **270** | **20.8%** | **0.28 [0.19, 0.41]** | |
| Total events | 55 | | 126 | | | | |
| Heterogeneity: Chi² = 0.62, df = 8 (P = 1.00); I² = 0% | | | | | | | |
| Test for overall effect: Z = 6.56 (P < 0.00001) | | | | | | | |
| **1.2.2 Retreatment** | | | | | | | |
| He, L. 2010 | 8 | 24 | 11 | 20 | 1.6% | 0.41 [0.12, 1.39] | 2010 |
| Feng, F.2023 | 7 | 34 | 15 | 34 | 2.4% | 0.33 [0.11, 0.96] | 2023 |
| **Subtotal (95% CI)** | | **58** | | **54** | **4.0%** | **0.36 [0.16, 0.81]** | |
| Total events | 15 | | 26 | | | | |
| Heterogeneity: Chi² = 0.07, df = 1 (P = 0.79); I² = 0% | | | | | | | |
| Test for overall effect: Z = 2.48 (P = 0.01) | | | | | | | |
| **1.2.3 Unclear** | | | | | | | |
| Ning, X. 2001 | 5 | 30 | 8 | 30 | 1.4% | 0.55 [0.16, 1.93] | 2001 |
| Pan, J. 2007 | 3 | 36 | 9 | 34 | 1.7% | 0.25 [0.06, 1.03] | 2007 |
| Lin, S. 2007 | 4 | 33 | 11 | 33 | 2.0% | 0.28 [0.08, 0.98] | 2007 |
| Huang, X. 2007 | 3 | 20 | 8 | 18 | 1.5% | 0.22 [0.05, 1.03] | 2007 |
| Li, Y. 2008 | 5 | 32 | 8 | 32 | 1.4% | 0.56 [0.16, 1.93] | 2008 |
| Deng, M. 2008 | 6 | 40 | 12 | 40 | 2.1% | 0.41 [0.14, 1.24] | 2008 |
| Ding, L. 2009 | 3 | 31 | 13 | 30 | 2.4% | 0.14 [0.03, 0.56] | 2009 |
| Li, Y. 2009 | 5 | 30 | 8 | 30 | 1.4% | 0.55 [0.16, 1.93] | 2009 |
| Wang, Y. 2010 | 2 | 24 | 10 | 24 | 1.9% | 0.13 [0.02, 0.67] | 2010 |
| Chen, Y. 2011 | 10 | 84 | 38 | 84 | 6.8% | 0.16 [0.07, 0.36] | 2011 |
| Wei, M. 2011 | 6 | 35 | 11 | 35 | 1.9% | 0.45 [0.15, 1.40] | 2011 |
| Li, L. 2011 | 7 | 30 | 17 | 30 | 2.6% | 0.23 [0.08, 0.71] | 2011 |
| Ran, F. 2011 | 4 | 30 | 12 | 30 | 2.1% | 0.23 [0.06, 0.83] | 2011 |
| Chen, F.2012 | 7 | 43 | 20 | 43 | 3.4% | 0.22 [0.08, 0.61] | 2012 |
| Yang, G. 2012 | 6 | 39 | 16 | 39 | 2.7% | 0.26 [0.09, 0.77] | 2012 |
| Zhuang, H. 2012 | 6 | 24 | 8 | 22 | 1.3% | 0.58 [0.16, 2.07] | 2012 |
| Han, S. 2013 | 11 | 90 | 40 | 90 | 7.1% | 0.17 [0.08, 0.37] | 2013 |
| Zhu, M. 2013 | 5 | 28 | 9 | 28 | 1.5% | 0.46 [0.13, 1.60] | 2013 |
| Zheng, S. 2013 | 6 | 31 | 16 | 31 | 2.6% | 0.23 [0.07, 0.70] | 2013 |
| Jiang, J. 2014 | 5 | 34 | 13 | 34 | 2.3% | 0.28 [0.09, 0.90] | 2014 |
| Xu, L. 2014 | 7 | 32 | 15 | 32 | 2.4% | 0.32 [0.11, 0.94] | 2014 |
| Xu, B. 2014 | 11 | 30 | 17 | 30 | 2.2% | 0.44 [0.16, 1.25] | 2014 |
| Song, X. 2015 | 5 | 59 | 19 | 59 | 3.5% | 0.19 [0.07, 0.57] | 2015 |
| Qin,D.2016 | 2 | 32 | 15 | 32 | 2.9% | 0.08 [0.02, 0.37] | 2016 |
| Yan, G. 2016 | 4 | 35 | 11 | 30 | 2.1% | 0.22 [0.06, 0.80] | 2016 |
| Huang, H. 2017 | 3 | 30 | 11 | 30 | 2.0% | 0.19 [0.05, 0.78] | 2017 |
| Wang, Y. 2019 | 8 | 45 | 20 | 45 | 3.3% | 0.27 [0.10, 0.71] | 2019 |
| Wu, C. 2019 | 7 | 25 | 15 | 25 | 2.2% | 0.26 [0.08, 0.85] | 2019 |
| Peng,H.2020 | 7 | 25 | 15 | 25 | 2.2% | 0.26 [0.08, 0.85] | 2020 |
| Jiang, T 2020 | 2 | 30 | 13 | 30 | 2.5% | 0.09 [0.02, 0.47] | 2020 |
| **Subtotal (95% CI)** | | **1087** | | **1075** | **75.2%** | **0.25 [0.21, 0.31]** | |
| Total events | 165 | | 438 | | | | |
| Heterogeneity: Chi² = 17.85, df = 29 (P = 0.95); I² = 0% | | | | | | | |
| Test for overall effect: Z = 12.88 (P < 0.00001) | | | | | | | |
| **Total (95% CI)** | | **1424** | | **1399** | **100.0%** | **0.26 [0.22, 0.32]** | |
| Total events | 235 | | 590 | | | | |
| Heterogeneity: Chi² = 19.19, df = 40 (P = 1.00); I² = 0% | | | | | | | |
| Test for overall effect: Z = 14.66 (P < 0.00001) | | | | | | | |
| Test for subgroup differences: Chi² = 0.77, df = 2 (P = 0.68), I² = 0% | | | | | | | |

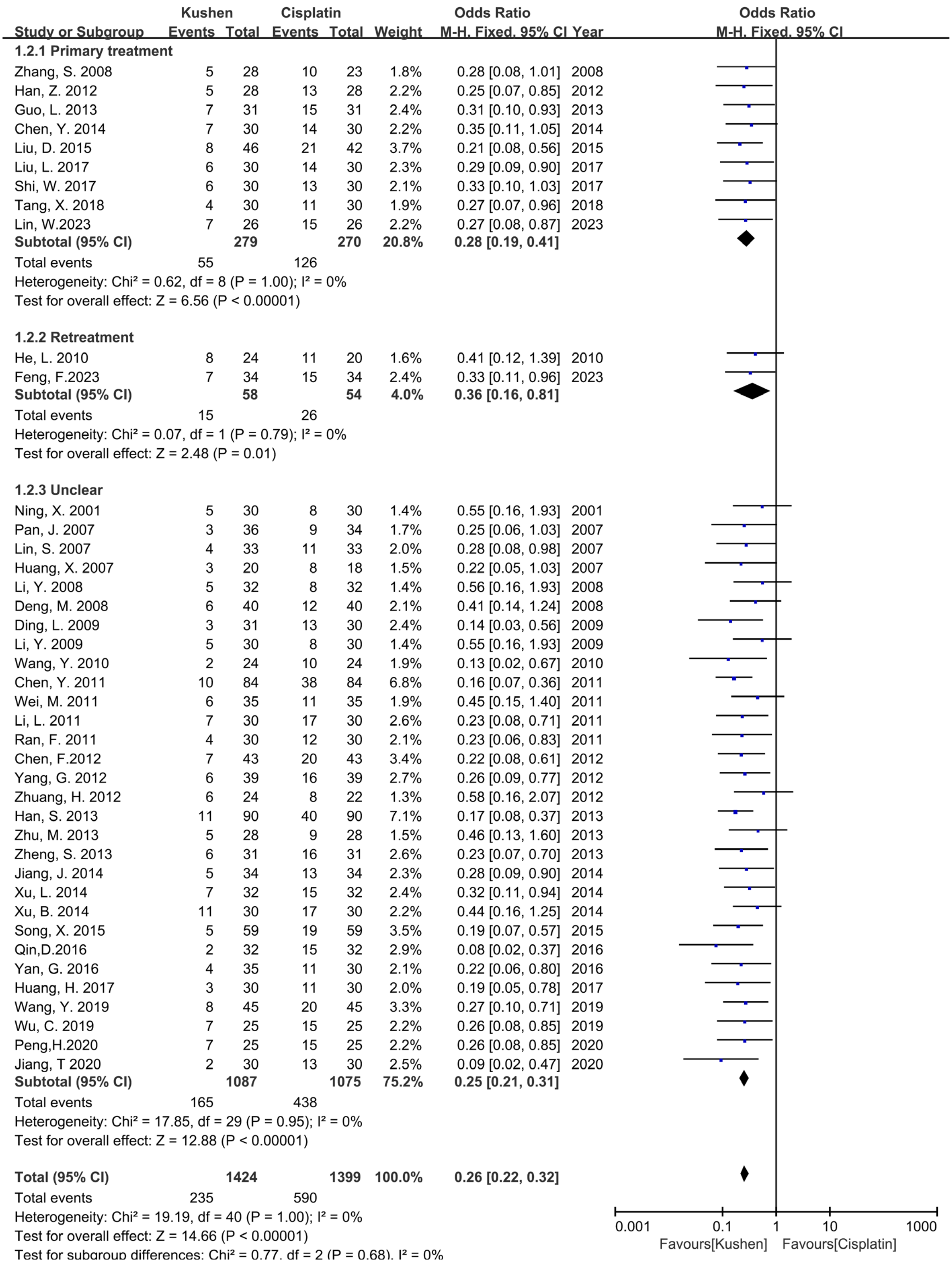


**Figure.S43 Subgroups analysis of pleurodesis failure via treatment history**

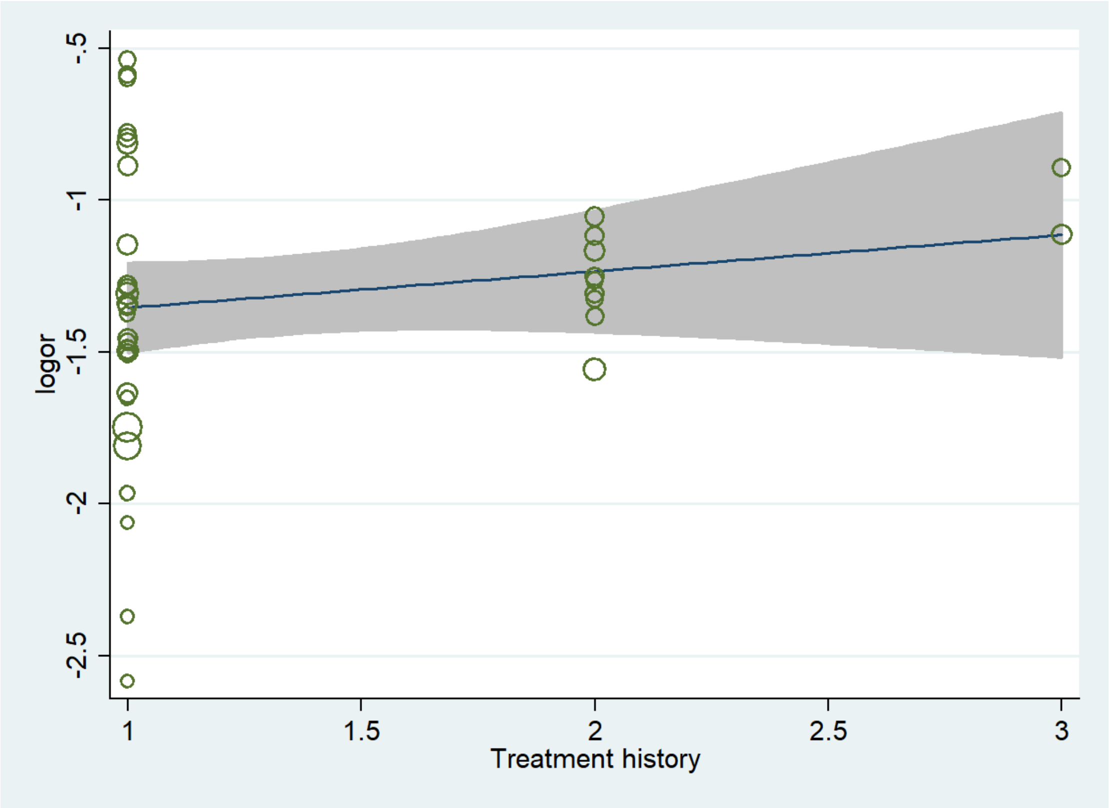


**Figure.S44 Meta-regression of pleurodesis failure via treatment history**

| Study or Subgroup | Kushen Events | Kushen Total | Cisplatin Events | Cisplatin Total | Weight | Odds Ratio M-H, Fixed, 95% CI | Year |
|---|---|---|---|---|---|---|---|
| **1.1.1 Indwelling pleural catheter** | | | | | | | |
| Pan, J. 2007 | 15 | 36 | 12 | 34 | 4.1% | 1.31 [0.50, 3.44] | 2007 |
| Li, Y. 2008 | 17 | 32 | 8 | 32 | 2.1% | 3.40 [1.18, 9.81] | 2008 |
| Zhang, S. 2008 | 8 | 28 | 5 | 23 | 2.2% | 1.44 [0.40, 5.21] | 2008 |
| Ding, L. 2009 | 14 | 31 | 9 | 30 | 2.9% | 1.92 [0.67, 5.51] | 2009 |
| Wang, Y. 2010 | 6 | 24 | 2 | 24 | 0.9% | 3.67 [0.66, 20.42] | 2010 |
| He, L. 2010 | 7 | 24 | 5 | 20 | 2.2% | 1.24 [0.32, 4.72] | 2010 |
| Chen, Y. 2011 | 54 | 84 | 22 | 84 | 4.5% | 5.07 [2.62, 9.82] | 2011 |
| Ran, F. 2011 | 12 | 30 | 10 | 30 | 3.4% | 1.33 [0.46, 3.82] | 2011 |
| Wei, M. 2011 | 13 | 35 | 10 | 35 | 3.6% | 1.48 [0.54, 4.03] | 2011 |
| Li, L. 2011 | 6 | 30 | 3 | 30 | 1.4% | 2.25 [0.51, 9.99] | 2011 |
| Han, Z. 2012 | 10 | 28 | 5 | 28 | 1.8% | 2.56 [0.74, 8.81] | 2012 |
| Chen, F.2012 | 16 | 43 | 9 | 43 | 3.2% | 2.24 [0.86, 5.85] | 2012 |
| Yang, G. 2012 | 14 | 39 | 11 | 39 | 4.0% | 1.43 [0.55, 3.71] | 2012 |
| Guo, L. 2013 | 14 | 31 | 7 | 31 | 2.2% | 2.82 [0.94, 8.48] | 2013 |
| Han, S. 2013 | 35 | 90 | 13 | 90 | 4.5% | 3.77 [1.83, 7.78] | 2013 |
| Zhu, M. 2013 | 9 | 28 | 6 | 28 | 2.3% | 1.74 [0.52, 5.78] | 2013 |
| Zheng, S. 2013 | 6 | 31 | 3 | 31 | 1.4% | 2.24 [0.51, 9.91] | 2013 |
| Chen, Y. 2014 | 13 | 30 | 7 | 30 | 2.3% | 2.51 [0.83, 7.64] | 2014 |
| Jiang, J. 2014 | 11 | 34 | 7 | 34 | 2.7% | 1.84 [0.61, 5.54] | 2014 |
| Song, X. 2015 | 47 | 59 | 30 | 59 | 3.5% | 3.79 [1.68, 8.54] | 2015 |
| Liu, D. 2015 | 20 | 46 | 6 | 42 | 2.0% | 4.62 [1.63, 13.09] | 2015 |
| Yan, G. 2016 | 22 | 35 | 12 | 30 | 2.7% | 2.54 [0.93, 6.91] | 2016 |
| Qin,D.2016 | 11 | 32 | 6 | 32 | 2.3% | 2.27 [0.72, 7.16] | 2016 |
| Huang, H. 2017 | 20 | 30 | 11 | 30 | 2.1% | 3.45 [1.19, 9.99] | 2017 |
| Liu, L. 2017 | 13 | 30 | 7 | 30 | 2.3% | 2.51 [0.83, 7.64] | 2017 |
| Shi, W. 2017 | 14 | 30 | 8 | 30 | 2.4% | 2.41 [0.82, 7.10] | 2017 |
| Tang, X. 2018 | 15 | 30 | 10 | 30 | 2.9% | 2.00 [0.70, 5.68] | 2018 |
| Wang, Y. 2019 | 20 | 45 | 9 | 45 | 2.9% | 3.20 [1.25, 8.17] | 2019 |
| Wu, C. 2019 | 8 | 25 | 4 | 25 | 1.6% | 2.47 [0.63, 9.63] | 2019 |
| Peng,H.2020 | 12 | 25 | 5 | 25 | 1.5% | 3.69 [1.05, 12.96] | 2020 |
| Jiang, T 2020 | 24 | 30 | 6 | 30 | 0.7% | 16.00 [4.52, 56.70] | 2020 |
| Feng, F.2023 | 11 | 34 | 7 | 34 | 2.7% | 1.84 [0.61, 5.54] | 2023 |
| Lin, W.2023 | 8 | 26 | 5 | 26 | 2.0% | 1.87 [0.52, 6.73] | 2023 |
| **Subtotal (95% CI)** | | **1185** | | **1164** | **83.3%** | **2.63 [2.19, 3.16]** | |
| Total events | 525 | | 280 | | | | |
| Heterogeneity: Chi² = 26.51, df = 32 (P = 0.74); I² = 0% | | | | | | | |
| Test for overall effect: Z = 10.43 (P < 0.00001) | | | | | | | |
| **1.1.2 Thoracentesis** | | | | | | | |
| Ning, X. 2001 | 17 | 30 | 8 | 30 | 2.0% | 3.60 [1.22, 10.64] | 2001 |
| Huang, X. 2007 | 9 | 20 | 4 | 18 | 1.3% | 2.86 [0.69, 11.82] | 2007 |
| Lin, S. 2007 | 20 | 33 | 10 | 33 | 2.3% | 3.54 [1.28, 9.80] | 2007 |
| Deng, M. 2008 | 24 | 40 | 10 | 40 | 2.3% | 4.50 [1.73, 11.70] | 2008 |
| Li, Y. 2009 | 17 | 30 | 8 | 30 | 2.0% | 3.60 [1.22, 10.64] | 2009 |
| Zhuang, H. 2012 | 7 | 24 | 6 | 22 | 2.5% | 1.10 [0.30, 3.98] | 2012 |
| Xu, B. 2014 | 12 | 30 | 3 | 30 | 1.0% | 6.00 [1.48, 24.30] | 2014 |
| Xu, L. 2014 | 18 | 32 | 13 | 32 | 3.3% | 1.88 [0.70, 5.07] | 2014 |
| **Subtotal (95% CI)** | | **239** | | **235** | **16.7%** | **3.09 [2.09, 4.56]** | |
| Total events | 124 | | 62 | | | | |
| Heterogeneity: Chi² = 5.14, df = 7 (P = 0.64); I² = 0% | | | | | | | |
| Test for overall effect: Z = 5.66 (P < 0.00001) | | | | | | | |
| **Total (95% CI)** | | **1424** | | **1399** | **100.0%** | **2.71 [2.30, 3.19]** | |
| Total events | 649 | | 342 | | | | |
| Heterogeneity: Chi² = 32.18, df = 40 (P = 0.81); I² = 0% | | | | | | | |
| Test for overall effect: Z = 11.84 (P < 0.00001) | | | | | | | |
| Test for subgroup differences: Chi² = 0.53, df = 1 (P = 0.47), I² = 0% | | | | | | | |

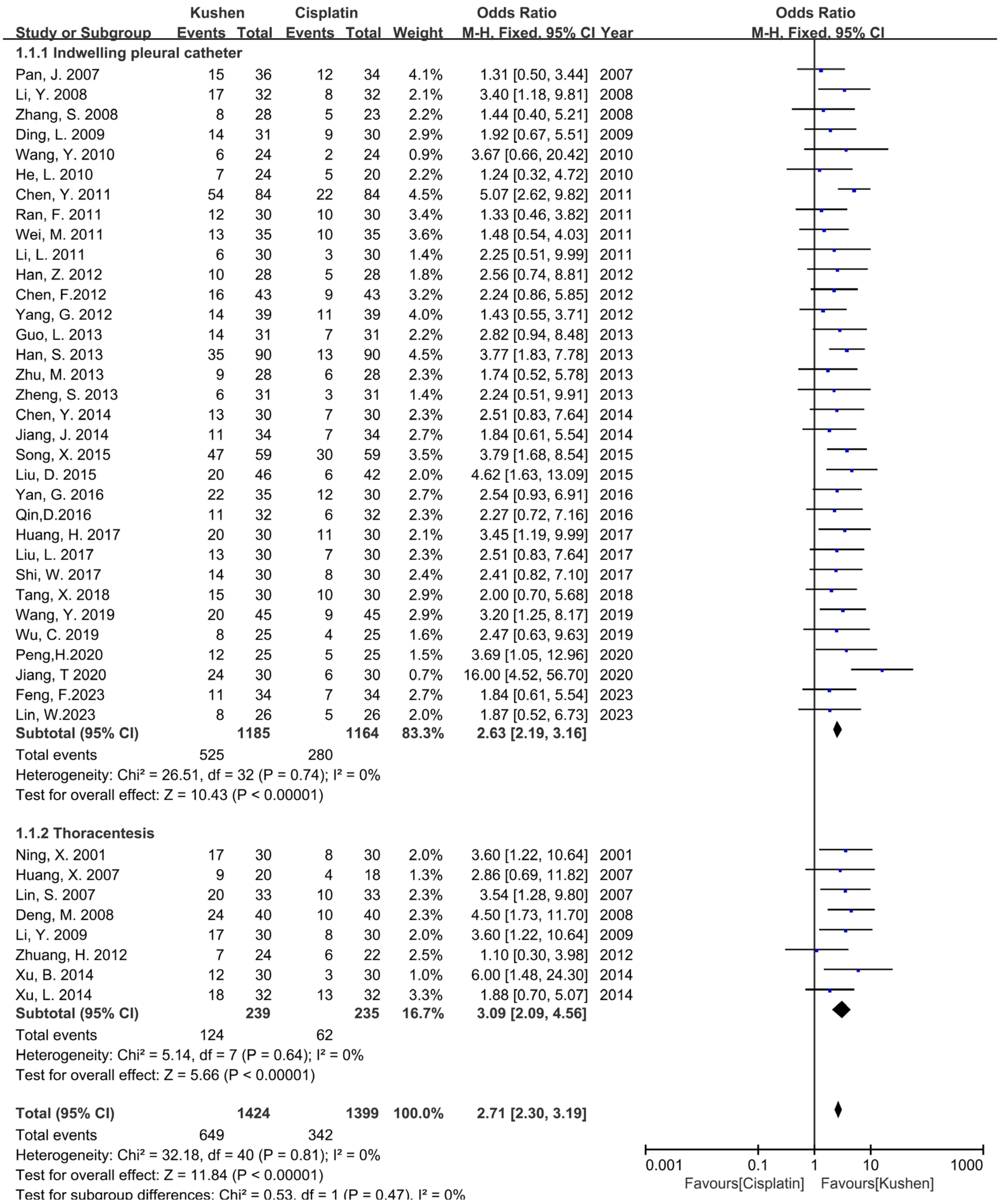


**Figure.S45 Subgroups analysis of complete response via drainage method**

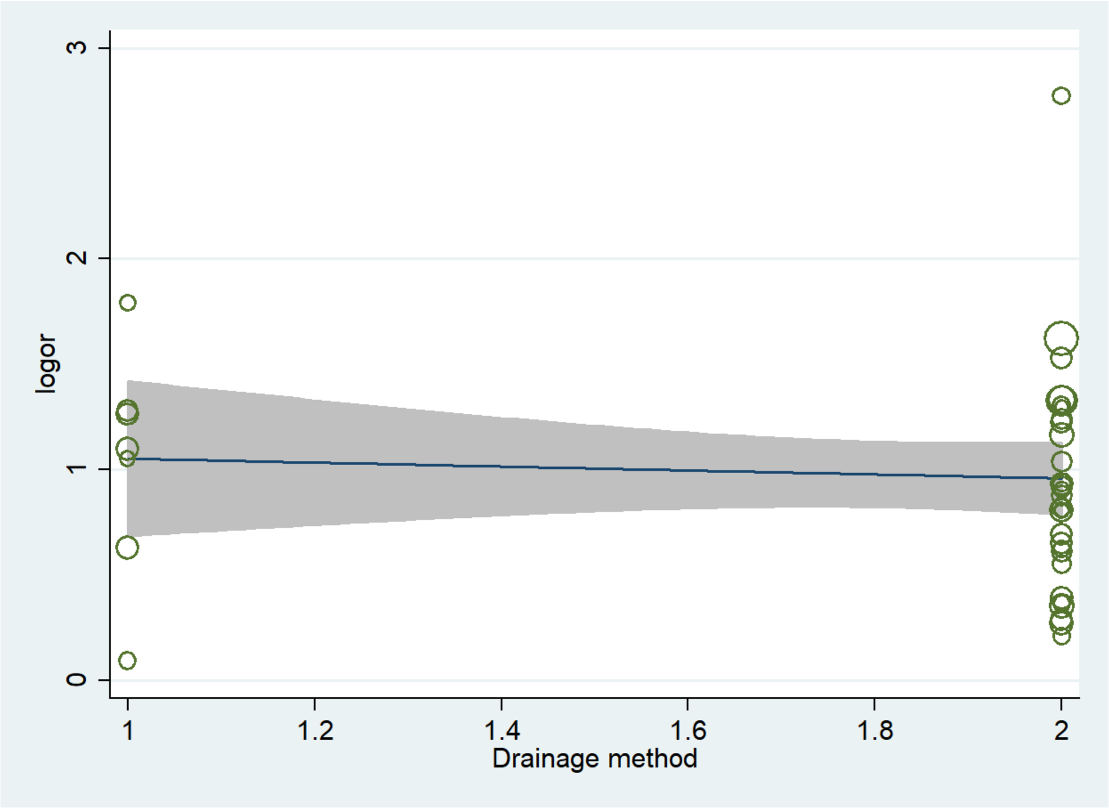


**Figure.S46 Meta-regression of complete response via drainage method**

| Study or Subgroup | Kushen Events | Kushen Total | Cisplatin Events | Cisplatin Total | Weight | Odds Ratio M-H, Fixed, 95% CI | Year |
|---|---|---|---|---|---|---|---|
| **1.2.1 Indwelling pleural catheter** | | | | | | | |
| Pan, J. 2007 | 3 | 36 | 9 | 34 | 1.7% | 0.25 [0.06, 1.03] | 2007 |
| Li, Y. 2008 | 5 | 32 | 8 | 32 | 1.4% | 0.56 [0.16, 1.93] | 2008 |
| Zhang, S. 2008 | 5 | 28 | 10 | 23 | 1.8% | 0.28 [0.08, 1.01] | 2008 |
| Ding, L. 2009 | 3 | 31 | 13 | 30 | 2.4% | 0.14 [0.03, 0.56] | 2009 |
| Wang, Y. 2010 | 2 | 24 | 10 | 24 | 1.9% | 0.13 [0.02, 0.67] | 2010 |
| He, L. 2010 | 8 | 24 | 11 | 20 | 1.6% | 0.41 [0.12, 1.39] | 2010 |
| Chen, Y. 2011 | 10 | 84 | 38 | 84 | 6.8% | 0.16 [0.07, 0.36] | 2011 |
| Wei, M. 2011 | 6 | 35 | 11 | 35 | 1.9% | 0.45 [0.15, 1.40] | 2011 |
| Li, L. 2011 | 7 | 30 | 17 | 30 | 2.6% | 0.23 [0.08, 0.71] | 2011 |
| Ran, F. 2011 | 4 | 30 | 12 | 30 | 2.1% | 0.23 [0.06, 0.83] | 2011 |
| Chen, F.2012 | 7 | 43 | 20 | 43 | 3.4% | 0.22 [0.08, 0.61] | 2012 |
| Yang, G. 2012 | 6 | 39 | 16 | 39 | 2.7% | 0.26 [0.09, 0.77] | 2012 |
| Han, Z. 2012 | 5 | 28 | 13 | 28 | 2.2% | 0.25 [0.07, 0.85] | 2012 |
| Han, S. 2013 | 11 | 90 | 40 | 90 | 7.1% | 0.17 [0.08, 0.37] | 2013 |
| Zhu, M. 2013 | 5 | 28 | 9 | 28 | 1.5% | 0.46 [0.13, 1.60] | 2013 |
| Guo, L. 2013 | 7 | 31 | 15 | 31 | 2.4% | 0.31 [0.10, 0.93] | 2013 |
| Zheng, S. 2013 | 6 | 31 | 16 | 31 | 2.6% | 0.23 [0.07, 0.70] | 2013 |
| Jiang, J. 2014 | 5 | 34 | 13 | 34 | 2.3% | 0.28 [0.09, 0.90] | 2014 |
| Chen, Y. 2014 | 7 | 30 | 14 | 30 | 2.2% | 0.35 [0.11, 1.05] | 2014 |
| Liu, D. 2015 | 8 | 46 | 21 | 42 | 3.7% | 0.21 [0.08, 0.56] | 2015 |
| Song, X. 2015 | 5 | 59 | 19 | 59 | 3.5% | 0.19 [0.07, 0.57] | 2015 |
| Qin,D.2016 | 2 | 32 | 15 | 32 | 2.9% | 0.08 [0.02, 0.37] | 2016 |
| Yan, G. 2016 | 4 | 35 | 11 | 30 | 2.1% | 0.22 [0.06, 0.80] | 2016 |
| Liu, L. 2017 | 6 | 30 | 14 | 30 | 2.3% | 0.29 [0.09, 0.90] | 2017 |
| Huang, H. 2017 | 3 | 30 | 11 | 30 | 2.0% | 0.19 [0.05, 0.78] | 2017 |
| Shi, W. 2017 | 6 | 30 | 13 | 30 | 2.1% | 0.33 [0.10, 1.03] | 2017 |
| Tang, X. 2018 | 4 | 30 | 11 | 30 | 1.9% | 0.27 [0.07, 0.96] | 2018 |
| Wang, Y. 2019 | 8 | 45 | 20 | 45 | 3.3% | 0.27 [0.10, 0.71] | 2019 |
| Wu, C. 2019 | 7 | 25 | 15 | 25 | 2.2% | 0.26 [0.08, 0.85] | 2019 |
| Peng,H.2020 | 7 | 25 | 15 | 25 | 2.2% | 0.26 [0.08, 0.85] | 2020 |
| Jiang, T 2020 | 2 | 30 | 13 | 30 | 2.5% | 0.09 [0.02, 0.47] | 2020 |
| Feng, F.2023 | 7 | 34 | 15 | 34 | 2.4% | 0.33 [0.11, 0.96] | 2023 |
| Lin, W.2023 | 7 | 26 | 15 | 26 | 2.2% | 0.27 [0.08, 0.87] | 2023 |
| **Subtotal (95% CI)** | | **1185** | | **1164** | **86.0%** | **0.24 [0.20, 0.29]** | |
| Total events | 188 | | 503 | | | | |
| Heterogeneity: Chi² = 12.80, df = 32 (P = 1.00); I² = 0% | | | | | | | |
| Test for overall effect: Z = 14.15 (P < 0.00001) | | | | | | | |
| **1.2.2 Thoracentesis** | | | | | | | |
| Ning, X. 2001 | 5 | 30 | 8 | 30 | 1.4% | 0.55 [0.16, 1.93] | 2001 |
| Huang, X. 2007 | 3 | 20 | 8 | 18 | 1.5% | 0.22 [0.05, 1.03] | 2007 |
| Lin, S. 2007 | 4 | 33 | 11 | 33 | 2.0% | 0.28 [0.08, 0.98] | 2007 |
| Deng, M. 2008 | 6 | 40 | 12 | 40 | 2.1% | 0.41 [0.14, 1.24] | 2008 |
| Li, Y. 2009 | 5 | 30 | 8 | 30 | 1.4% | 0.55 [0.16, 1.93] | 2009 |
| Zhuang, H. 2012 | 6 | 24 | 8 | 22 | 1.3% | 0.58 [0.16, 2.07] | 2012 |
| Xu, B. 2014 | 11 | 30 | 17 | 30 | 2.2% | 0.44 [0.16, 1.25] | 2014 |
| Xu, L. 2014 | 7 | 32 | 15 | 32 | 2.4% | 0.32 [0.11, 0.94] | 2014 |
| **Subtotal (95% CI)** | | **239** | | **235** | **14.0%** | **0.40 [0.26, 0.62]** | |
| Total events | 47 | | 87 | | | | |
| Heterogeneity: Chi² = 1.95, df = 7 (P = 0.96); I² = 0% | | | | | | | |
| Test for overall effect: Z = 4.21 (P < 0.0001) | | | | | | | |
| **Total (95% CI)** | | **1424** | | **1399** | **100.0%** | **0.26 [0.22, 0.32]** | |
| Total events | 235 | | 590 | | | | |
| Heterogeneity: Chi² = 19.19, df = 40 (P = 1.00); I² = 0% | | | | | | | |
| Test for overall effect: Z = 14.66 (P < 0.00001) | | | | | | | |
| Test for subgroup differences: Chi² = 4.71, df = 1 (P = 0.03), I² = 78.8% | | | | | | | |

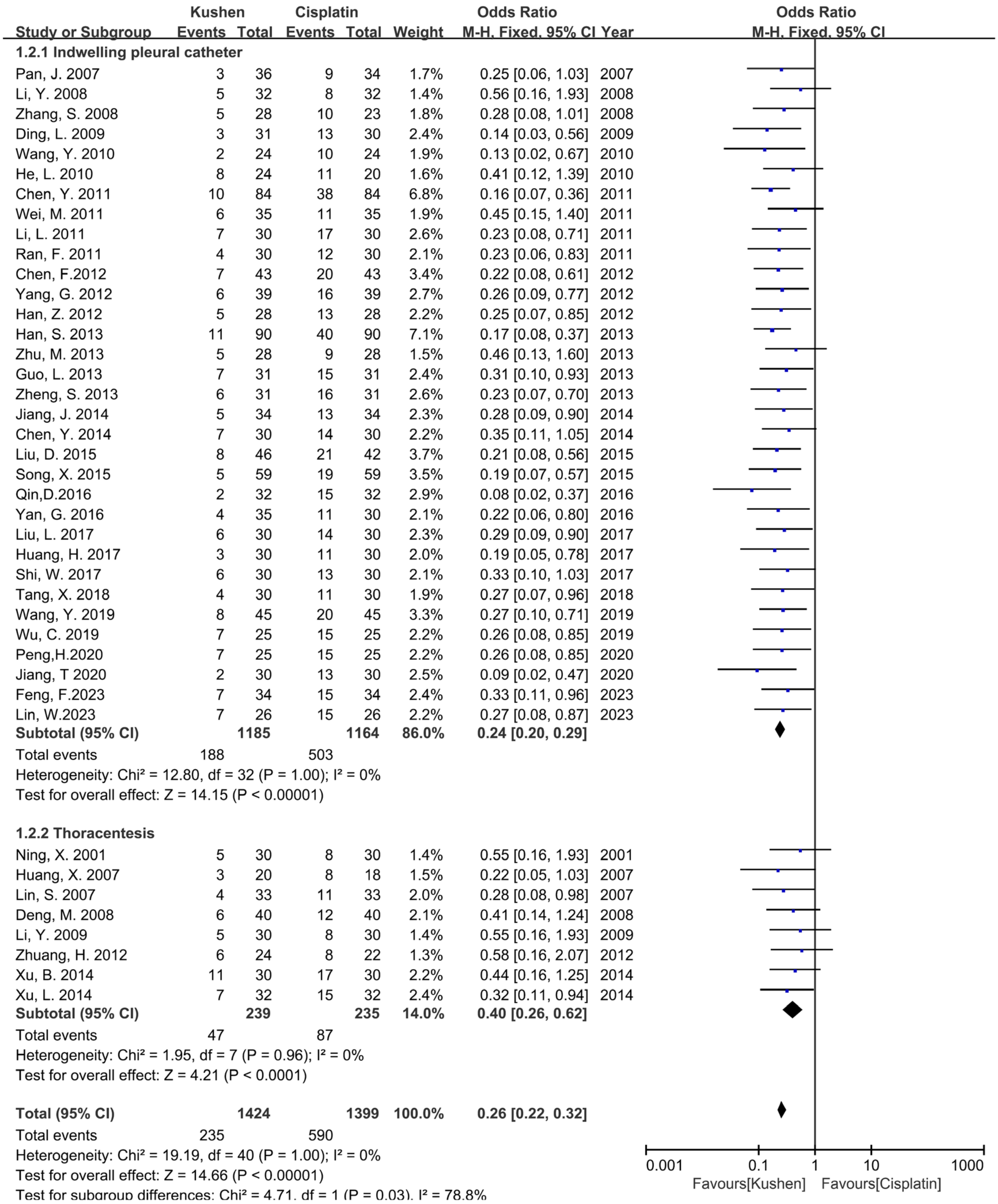


**Figure.S47 Subgroups analysis of pleurodesis failure via drainage method**

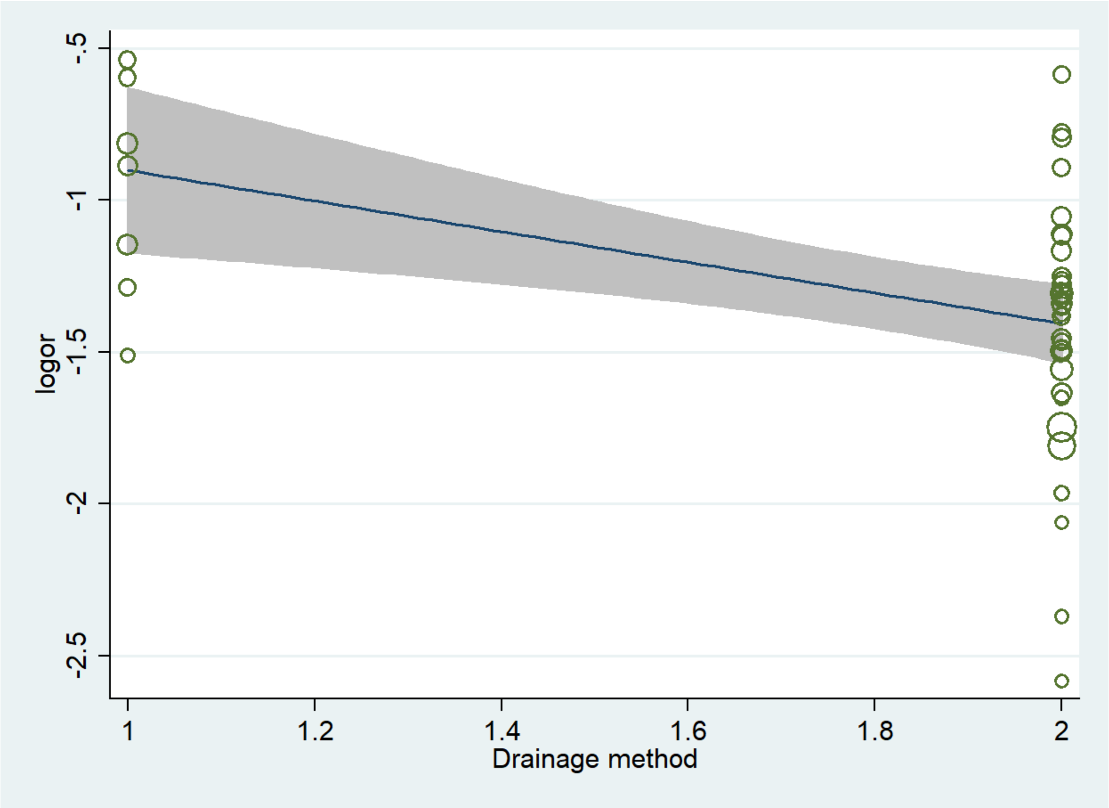


**Figure.S48 Meta-regression of pleurodesis failure via drainage method**

| Study or Subgroup | Kushen Events | Kushen Total | Cisplatin Events | Cisplatin Total | Weight | Odds Ratio M-H, Fixed, 95% CI | Year |
|---|---|---|---|---|---|---|---|
| **1.1.1 Compound Kushen injection(20-30 ml)** | | | | | | | |
| Ning, X. 2001 | 17 | 30 | 8 | 30 | 2.0% | 3.60 [1.22, 10.64] | 2001 |
| Lin, S. 2007 | 20 | 33 | 10 | 33 | 2.3% | 3.54 [1.28, 9.80] | 2007 |
| Pan, J. 2007 | 15 | 36 | 12 | 34 | 4.1% | 1.31 [0.50, 3.44] | 2007 |
| Huang, X. 2007 | 9 | 20 | 4 | 18 | 1.3% | 2.86 [0.69, 11.82] | 2007 |
| Zhang, S. 2008 | 8 | 28 | 5 | 23 | 2.2% | 1.44 [0.40, 5.21] | 2008 |
| Deng, M. 2008 | 24 | 40 | 10 | 40 | 2.3% | 4.50 [1.73, 11.70] | 2008 |
| Li, Y. 2008 | 17 | 32 | 8 | 32 | 2.1% | 3.40 [1.18, 9.81] | 2008 |
| Li, Y. 2009 | 17 | 30 | 8 | 30 | 2.0% | 3.60 [1.22, 10.64] | 2009 |
| Ding, L. 2009 | 14 | 31 | 9 | 30 | 2.9% | 1.92 [0.67, 5.51] | 2009 |
| Wang, Y. 2010 | 6 | 24 | 2 | 24 | 0.9% | 3.67 [0.66, 20.42] | 2010 |
| Ran, F. 2011 | 12 | 30 | 10 | 30 | 3.4% | 1.33 [0.46, 3.82] | 2011 |
| Wei, M. 2011 | 13 | 35 | 10 | 35 | 3.6% | 1.48 [0.54, 4.03] | 2011 |
| Li, L. 2011 | 6 | 30 | 3 | 30 | 1.4% | 2.25 [0.51, 9.99] | 2011 |
| Chen, F.2012 | 16 | 43 | 9 | 43 | 3.2% | 2.24 [0.86, 5.85] | 2012 |
| Yang, G. 2012 | 14 | 39 | 11 | 39 | 4.0% | 1.43 [0.55, 3.71] | 2012 |
| Guo, L. 2013 | 14 | 31 | 7 | 31 | 2.2% | 2.82 [0.94, 8.48] | 2013 |
| Zhu, M. 2013 | 9 | 28 | 6 | 28 | 2.3% | 1.74 [0.52, 5.78] | 2013 |
| Zheng, S. 2013 | 6 | 31 | 3 | 31 | 1.4% | 2.24 [0.51, 9.91] | 2013 |
| Chen, Y. 2014 | 13 | 30 | 7 | 30 | 2.3% | 2.51 [0.83, 7.64] | 2014 |
| Jiang, J. 2014 | 11 | 34 | 7 | 34 | 2.7% | 1.84 [0.61, 5.54] | 2014 |
| Song, X. 2015 | 47 | 59 | 30 | 59 | 3.5% | 3.79 [1.68, 8.54] | 2015 |
| Liu, D. 2015 | 20 | 46 | 6 | 42 | 2.0% | 4.62 [1.63, 13.09] | 2015 |
| Yan, G. 2016 | 22 | 35 | 12 | 30 | 2.7% | 2.54 [0.93, 6.91] | 2016 |
| Qin,D.2016 | 11 | 32 | 6 | 32 | 2.3% | 2.27 [0.72, 7.16] | 2016 |
| Huang, H. 2017 | 20 | 30 | 11 | 30 | 2.1% | 3.45 [1.19, 9.99] | 2017 |
| Liu, L. 2017 | 13 | 30 | 7 | 30 | 2.3% | 2.51 [0.83, 7.64] | 2017 |
| Shi, W. 2017 | 14 | 30 | 8 | 30 | 2.4% | 2.41 [0.82, 7.10] | 2017 |
| Wang, Y. 2019 | 20 | 45 | 9 | 45 | 2.9% | 3.20 [1.25, 8.17] | 2019 |
| Jiang, T 2020 | 24 | 30 | 6 | 30 | 0.7% | 16.00 [4.52, 56.70] | 2020 |
| Lin, W.2023 | 8 | 26 | 5 | 26 | 2.0% | 1.87 [0.52, 6.73] | 2023 |
| Feng, F.2023 | 11 | 34 | 7 | 34 | 2.7% | 1.84 [0.61, 5.54] | 2023 |
| **Subtotal (95% CI)** | | **1032** | | **1013** | **74.2%** | **2.60 [2.15, 3.16]** | |
| Total events | 471 | | 256 | | | | |
| Heterogeneity: Chi² = 22.15, df = 30 (P = 0.85); I² = 0% | | | | | | | |
| Test for overall effect: Z = 9.72 (P < 0.00001) | | | | | | | |
| **1.1.2 Compound Kushen injection(40-60ml)** | | | | | | | |
| He, L. 2010 | 7 | 24 | 5 | 20 | 2.2% | 1.24 [0.32, 4.72] | 2010 |
| Chen, Y. 2011 | 54 | 84 | 22 | 84 | 4.5% | 5.07 [2.62, 9.82] | 2011 |
| Han, S. 2013 | 35 | 90 | 13 | 90 | 4.5% | 3.77 [1.83, 7.78] | 2013 |
| Xu, B. 2014 | 12 | 30 | 3 | 30 | 1.0% | 6.00 [1.48, 24.30] | 2014 |
| Xu, L. 2014 | 18 | 32 | 13 | 32 | 3.3% | 1.88 [0.70, 5.07] | 2014 |
| Tang, X. 2018 | 15 | 30 | 10 | 30 | 2.9% | 2.00 [0.70, 5.68] | 2018 |
| Wu, C. 2019 | 8 | 25 | 4 | 25 | 1.6% | 2.47 [0.63, 9.63] | 2019 |
| Peng,H.2020 | 12 | 25 | 5 | 25 | 1.5% | 3.69 [1.05, 12.96] | 2020 |
| **Subtotal (95% CI)** | | **340** | | **336** | **21.4%** | **3.27 [2.32, 4.59]** | |
| Total events | 161 | | 75 | | | | |
| Heterogeneity: Chi² = 6.84, df = 7 (P = 0.45); I² = 0% | | | | | | | |
| Test for overall effect: Z = 6.80 (P < 0.00001) | | | | | | | |
| **1.1.3 Compound Kushen injection (others)** | | | | | | | |
| Han, Z. 2012 | 10 | 28 | 5 | 28 | 1.8% | 2.56 [0.74, 8.81] | 2012 |
| Zhuang, H. 2012 | 7 | 24 | 6 | 22 | 2.5% | 1.10 [0.30, 3.98] | 2012 |
| **Subtotal (95% CI)** | | **52** | | **50** | **4.4%** | **1.71 [0.71, 4.13]** | |
| Total events | 17 | | 11 | | | | |
| Heterogeneity: Chi² = 0.86, df = 1 (P = 0.35); I² = 0% | | | | | | | |
| Test for overall effect: Z = 1.19 (P = 0.23) | | | | | | | |
| **Total (95% CI)** | | **1424** | | **1399** | **100.0%** | **2.71 [2.30, 3.19]** | |
| Total events | 649 | | 342 | | | | |
| Heterogeneity: Chi² = 32.18, df = 40 (P = 0.81); I² = 0% | | | | | | | |
| Test for overall effect: Z = 11.84 (P < 0.00001) | | | | | | | |
| Test for subgroup differences: Chi² = 2.36, df = 2 (P = 0.31), I² = 15.2% | | | | | | | |

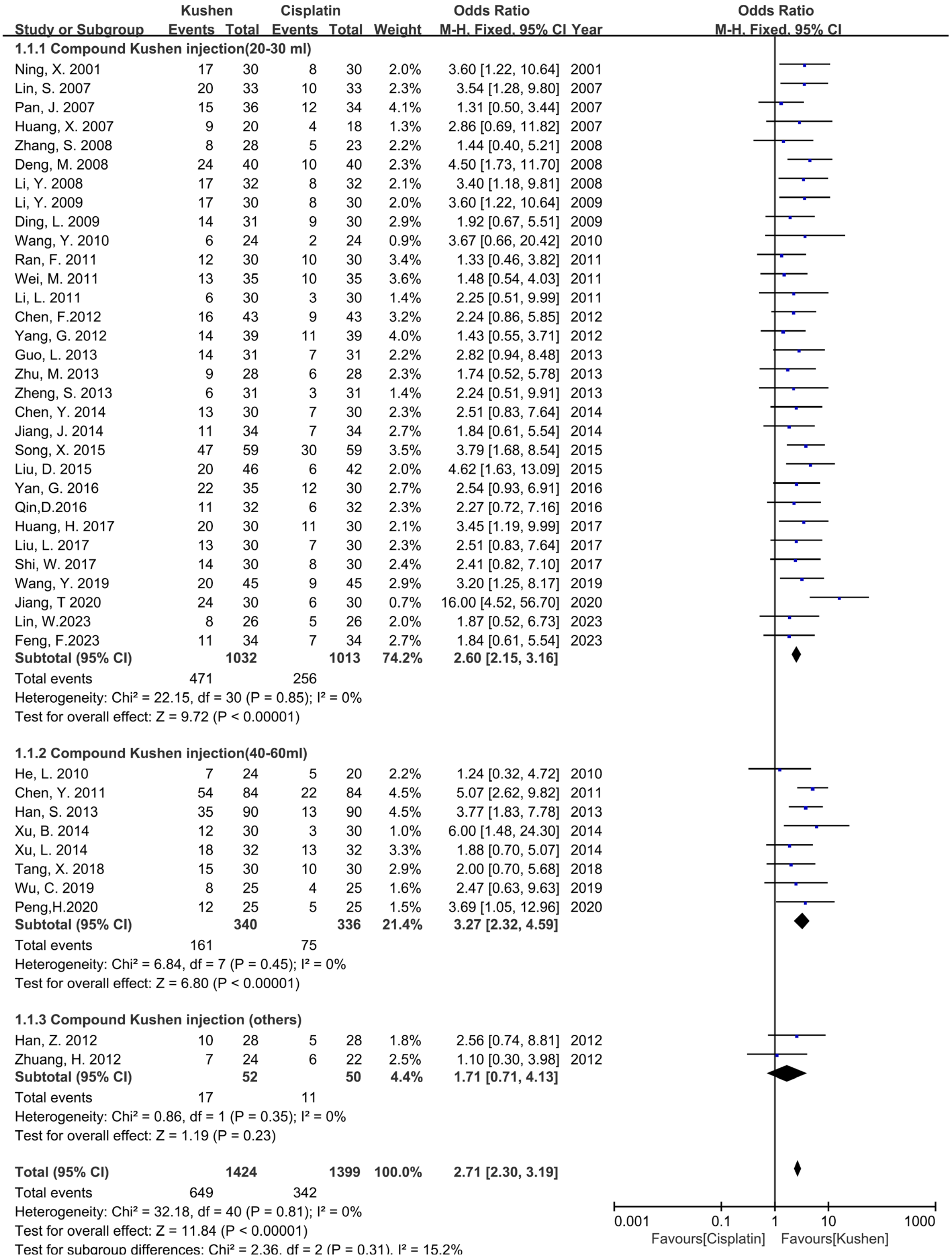


**Figure.S49 Subgroups analysis of complete response via Compound Kushen injection dosage**

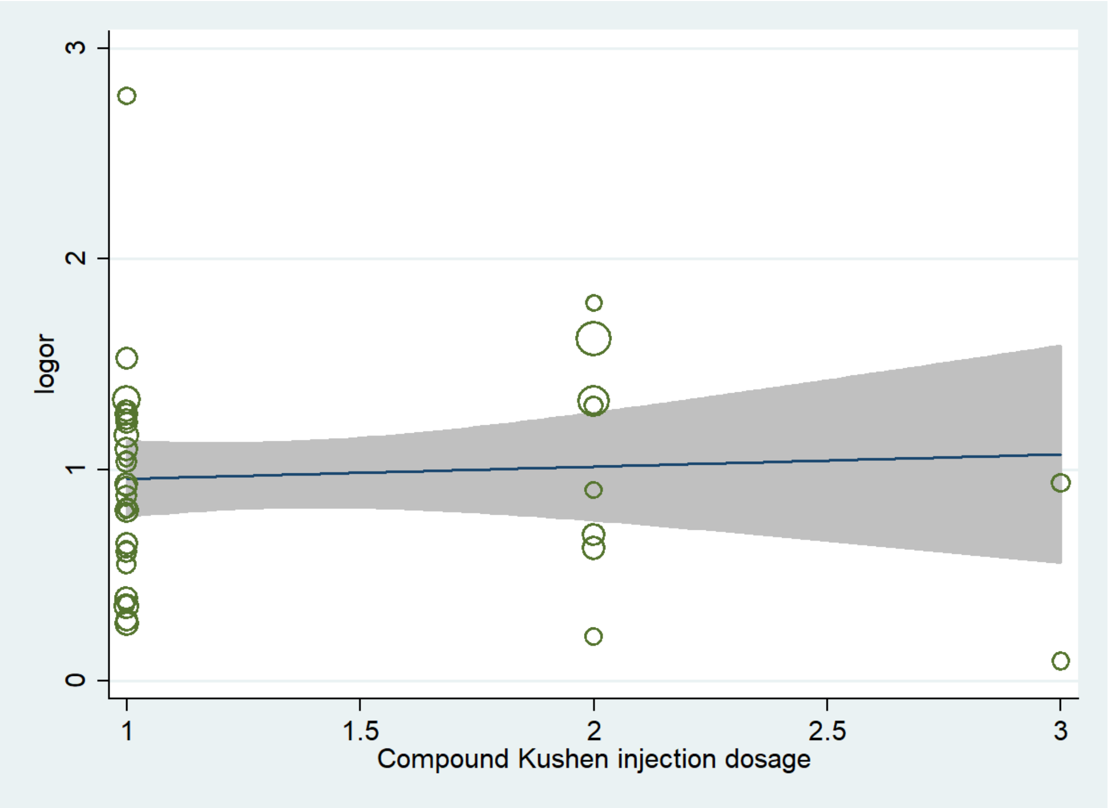


**Figure.S50 Meta-regression of complete response via Compound Kushen injection dosage**

| Study or Subgroup | Kushen Events | Kushen Total | Cisplatin Events | Cisplatin Total | Weight | Odds Ratio M-H, Fixed, 95% CI | Year |
|---|---|---|---|---|---|---|---|
| **1.2.1 Compound Kushen injection(20-30 ml)** | | | | | | | |
| Ning, X. 2001 | 5 | 30 | 8 | 30 | 1.4% | 0.55 [0.16, 1.93] | 2001 |
| Pan, J. 2007 | 3 | 36 | 9 | 34 | 1.7% | 0.25 [0.06, 1.03] | 2007 |
| Lin, S. 2007 | 4 | 33 | 11 | 33 | 2.0% | 0.28 [0.08, 0.98] | 2007 |
| Huang, X. 2007 | 3 | 20 | 8 | 18 | 1.5% | 0.22 [0.05, 1.03] | 2007 |
| Li, Y. 2008 | 5 | 32 | 8 | 32 | 1.4% | 0.56 [0.16, 1.93] | 2008 |
| Zhang, S. 2008 | 5 | 28 | 10 | 23 | 1.8% | 0.28 [0.08, 1.01] | 2008 |
| Deng, M. 2008 | 6 | 40 | 12 | 40 | 2.1% | 0.41 [0.14, 1.24] | 2008 |
| Li, Y. 2009 | 5 | 30 | 8 | 30 | 1.4% | 0.55 [0.16, 1.93] | 2009 |
| Ding, L. 2009 | 3 | 31 | 13 | 30 | 2.4% | 0.14 [0.03, 0.56] | 2009 |
| Wang, Y. 2010 | 2 | 24 | 10 | 24 | 1.9% | 0.13 [0.02, 0.67] | 2010 |
| Wei, M. 2011 | 6 | 35 | 11 | 35 | 1.9% | 0.45 [0.15, 1.40] | 2011 |
| Li, L. 2011 | 7 | 30 | 17 | 30 | 2.6% | 0.23 [0.08, 0.71] | 2011 |
| Ran, F. 2011 | 4 | 30 | 12 | 30 | 2.1% | 0.23 [0.06, 0.83] | 2011 |
| Chen, F.2012 | 7 | 43 | 20 | 43 | 3.4% | 0.22 [0.08, 0.61] | 2012 |
| Yang, G. 2012 | 6 | 39 | 16 | 39 | 2.7% | 0.26 [0.09, 0.77] | 2012 |
| Zhu, M. 2013 | 5 | 28 | 9 | 28 | 1.5% | 0.46 [0.13, 1.60] | 2013 |
| Guo, L. 2013 | 7 | 31 | 15 | 31 | 2.4% | 0.31 [0.10, 0.93] | 2013 |
| Zheng, S. 2013 | 6 | 31 | 16 | 31 | 2.6% | 0.23 [0.07, 0.70] | 2013 |
| Jiang, J. 2014 | 5 | 34 | 13 | 34 | 2.3% | 0.28 [0.09, 0.90] | 2014 |
| Chen, Y. 2014 | 7 | 30 | 14 | 30 | 2.2% | 0.35 [0.11, 1.05] | 2014 |
| Liu, D. 2015 | 8 | 46 | 21 | 42 | 3.7% | 0.21 [0.08, 0.56] | 2015 |
| Song, X. 2015 | 5 | 59 | 19 | 59 | 3.5% | 0.19 [0.07, 0.57] | 2015 |
| Qin,D.2016 | 2 | 32 | 15 | 32 | 2.9% | 0.08 [0.02, 0.37] | 2016 |
| Yan, G. 2016 | 4 | 35 | 11 | 30 | 2.1% | 0.22 [0.06, 0.80] | 2016 |
| Liu, L. 2017 | 6 | 30 | 14 | 30 | 2.3% | 0.29 [0.09, 0.90] | 2017 |
| Huang, H. 2017 | 3 | 30 | 11 | 30 | 2.0% | 0.19 [0.05, 0.78] | 2017 |
| Shi, W. 2017 | 6 | 30 | 13 | 30 | 2.1% | 0.33 [0.10, 1.03] | 2017 |
| Wang, Y. 2019 | 8 | 45 | 20 | 45 | 3.3% | 0.27 [0.10, 0.71] | 2019 |
| Jiang, T 2020 | 2 | 30 | 13 | 30 | 2.5% | 0.09 [0.02, 0.47] | 2020 |
| Lin, W.2023 | 7 | 26 | 15 | 26 | 2.2% | 0.27 [0.08, 0.87] | 2023 |
| Feng, F.2023 | 7 | 34 | 15 | 34 | 2.4% | 0.33 [0.11, 0.96] | 2023 |
| **Subtotal (95% CI)** | | **1032** | | **1013** | **70.1%** | **0.27 [0.22, 0.33]** | |
| Total events | 159 | | 407 | | | | |
| Heterogeneity: Chi² = 13.49, df = 30 (P = 1.00); I² = 0% | | | | | | | |
| Test for overall effect: Z = 12.19 (P < 0.00001) | | | | | | | |
| | | | | | | | |
| **1.2.2 Compound Kushen injection(40-60ml)** | | | | | | | |
| He, L. 2010 | 8 | 24 | 11 | 20 | 1.6% | 0.41 [0.12, 1.39] | 2010 |
| Chen, Y. 2011 | 10 | 84 | 38 | 84 | 6.8% | 0.16 [0.07, 0.36] | 2011 |
| Han, S. 2013 | 11 | 90 | 40 | 90 | 7.1% | 0.17 [0.08, 0.37] | 2013 |
| Xu, L. 2014 | 7 | 32 | 15 | 32 | 2.4% | 0.32 [0.11, 0.94] | 2014 |
| Xu, B. 2014 | 11 | 30 | 17 | 30 | 2.2% | 0.44 [0.16, 1.25] | 2014 |
| Tang, X. 2018 | 4 | 30 | 11 | 30 | 1.9% | 0.27 [0.07, 0.96] | 2018 |
| Wu, C. 2019 | 7 | 25 | 15 | 25 | 2.2% | 0.26 [0.08, 0.85] | 2019 |
| Peng,H.2020 | 7 | 25 | 15 | 25 | 2.2% | 0.26 [0.08, 0.85] | 2020 |
| **Subtotal (95% CI)** | | **340** | | **336** | **26.4%** | **0.24 [0.17, 0.34]** | |
| Total events | 65 | | 162 | | | | |
| Heterogeneity: Chi² = 3.98, df = 7 (P = 0.78); I² = 0% | | | | | | | |
| Test for overall effect: Z = 7.87 (P < 0.00001) | | | | | | | |
| | | | | | | | |
| **1.2.3 Compound Kushen injection (others)** | | | | | | | |
| Han, Z. 2012 | 5 | 28 | 13 | 28 | 2.2% | 0.25 [0.07, 0.85] | 2012 |
| Zhuang, H. 2012 | 6 | 24 | 8 | 22 | 1.3% | 0.58 [0.16, 2.07] | 2012 |
| **Subtotal (95% CI)** | | **52** | | **50** | **3.4%** | **0.37 [0.16, 0.89]** | |
| Total events | 11 | | 21 | | | | |
| Heterogeneity: Chi² = 0.88, df = 1 (P = 0.35); I² = 0% | | | | | | | |
| Test for overall effect: Z = 2.22 (P = 0.03) | | | | | | | |
| | | | | | | | |
| **Total (95% CI)** | | **1424** | | **1399** | **100.0%** | **0.26 [0.22, 0.32]** | |
| Total events | 235 | | 590 | | | | |
| Heterogeneity: Chi² = 19.19, df = 40 (P = 1.00); I² = 0% | | | | | | | |
| Test for overall effect: Z = 14.66 (P < 0.00001) | | | | | | | |
| Test for subgroup differences: Chi² = 0.87, df = 2 (P = 0.65), I² = 0% | | | | | | | |

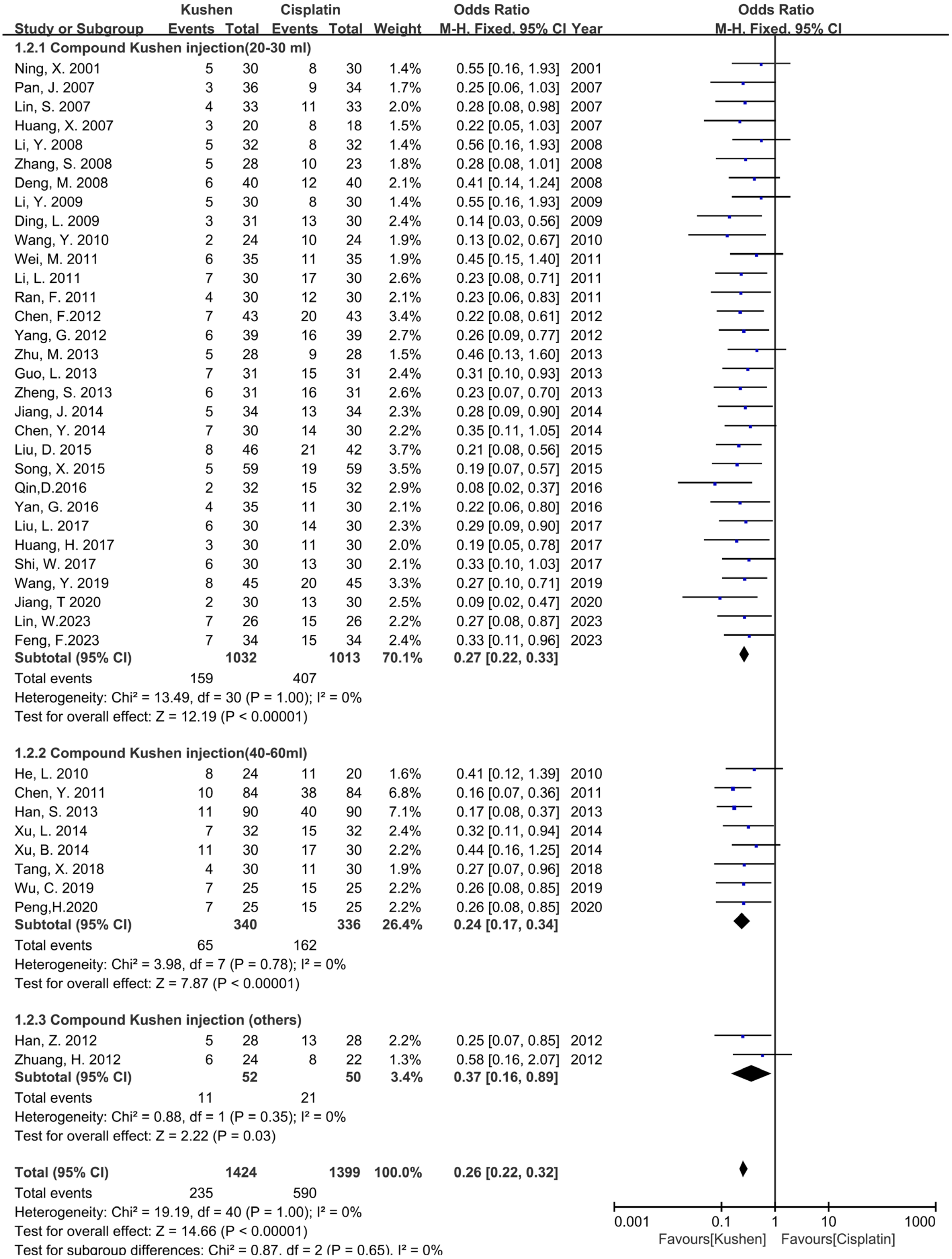


**Figure.S51 Subgroups analysis of pleurodesis failure via Compound Kushen injection dosage**

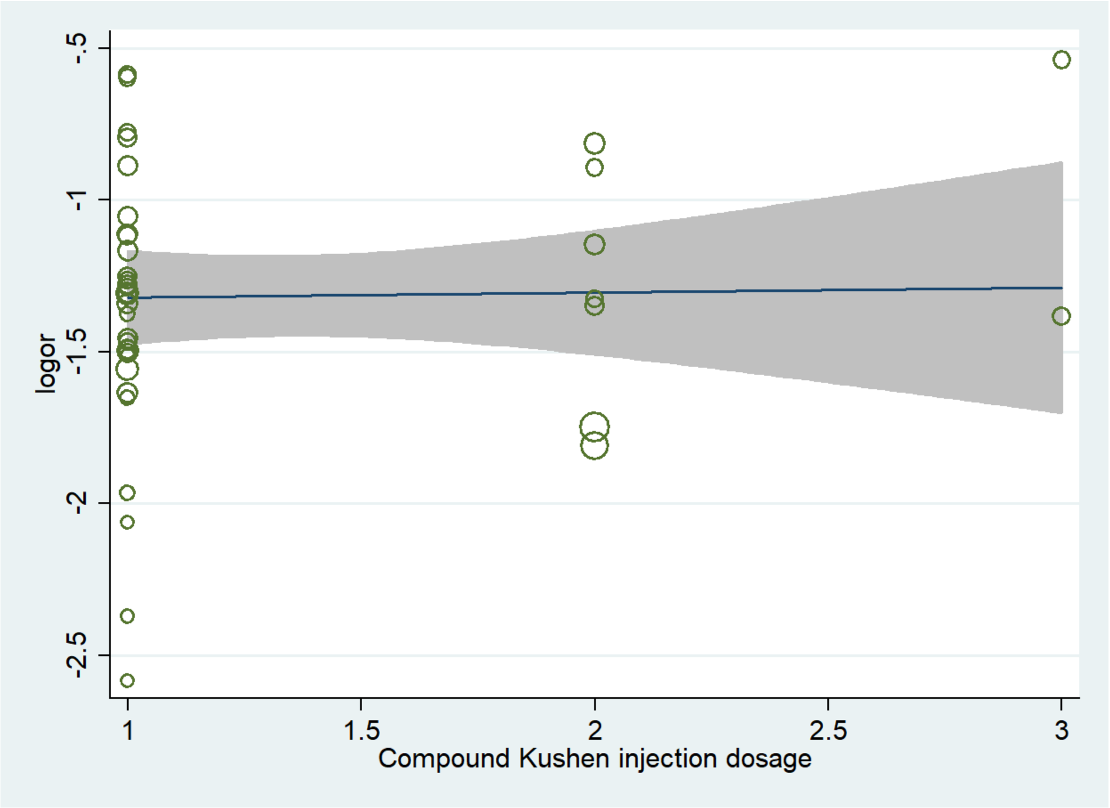


**Figure.S52 Meta-regression of pleurodesis failure via Compound Kushen injection dosage**

| Study or Subgroup | Kushen Events | Kushen Total | Cisplatin Events | Cisplatin Total | Weight | Odds Ratio M-H, Fixed, 95% CI | Year |
|---|---|---|---|---|---|---|---|
| **1.1.1 One time/week** | | | | | | | |
| Ning, X. 2001 | 17 | 30 | 8 | 30 | 2.0% | 3.60 [1.22, 10.64] | 2001 |
| Lin, S. 2007 | 20 | 33 | 10 | 33 | 2.3% | 3.54 [1.28, 9.80] | 2007 |
| Pan, J. 2007 | 15 | 36 | 12 | 34 | 4.1% | 1.31 [0.50, 3.44] | 2007 |
| Huang, X. 2007 | 9 | 20 | 4 | 18 | 1.3% | 2.86 [0.69, 11.82] | 2007 |
| Zhang, S. 2008 | 8 | 28 | 5 | 23 | 2.2% | 1.44 [0.40, 5.21] | 2008 |
| Deng, M. 2008 | 24 | 40 | 10 | 40 | 2.3% | 4.50 [1.73, 11.70] | 2008 |
| Li, Y. 2008 | 17 | 32 | 8 | 32 | 2.1% | 3.40 [1.18, 9.81] | 2008 |
| Li, Y. 2009 | 17 | 30 | 8 | 30 | 2.0% | 3.60 [1.22, 10.64] | 2009 |
| Ding, L. 2009 | 14 | 31 | 9 | 30 | 2.9% | 1.92 [0.67, 5.51] | 2009 |
| He, L. 2010 | 7 | 24 | 5 | 20 | 2.2% | 1.24 [0.32, 4.72] | 2010 |
| Chen, Y. 2011 | 54 | 84 | 22 | 84 | 4.5% | 5.07 [2.62, 9.82] | 2011 |
| Ran, F. 2011 | 12 | 30 | 10 | 30 | 3.4% | 1.33 [0.46, 3.82] | 2011 |
| Wei, M. 2011 | 13 | 35 | 10 | 35 | 3.6% | 1.48 [0.54, 4.03] | 2011 |
| Li, L. 2011 | 6 | 30 | 3 | 30 | 1.4% | 2.25 [0.51, 9.99] | 2011 |
| Han, Z. 2012 | 10 | 28 | 5 | 28 | 1.8% | 2.56 [0.74, 8.81] | 2012 |
| Chen, F.2012 | 16 | 43 | 9 | 43 | 3.2% | 2.24 [0.86, 5.85] | 2012 |
| Yang, G. 2012 | 14 | 39 | 11 | 39 | 4.0% | 1.43 [0.55, 3.71] | 2012 |
| Zhuang, H. 2012 | 7 | 24 | 6 | 22 | 2.5% | 1.10 [0.30, 3.98] | 2012 |
| Guo, L. 2013 | 14 | 31 | 7 | 31 | 2.2% | 2.82 [0.94, 8.48] | 2013 |
| Han, S. 2013 | 35 | 90 | 13 | 90 | 4.5% | 3.77 [1.83, 7.78] | 2013 |
| Zhu, M. 2013 | 9 | 28 | 6 | 28 | 2.3% | 1.74 [0.52, 5.78] | 2013 |
| Xu, B. 2014 | 12 | 30 | 3 | 30 | 1.0% | 6.00 [1.48, 24.30] | 2014 |
| Xu, L. 2014 | 18 | 32 | 13 | 32 | 3.3% | 1.88 [0.70, 5.07] | 2014 |
| Chen, Y. 2014 | 13 | 30 | 7 | 30 | 2.3% | 2.51 [0.83, 7.64] | 2014 |
| Jiang, J. 2014 | 11 | 34 | 7 | 34 | 2.7% | 1.84 [0.61, 5.54] | 2014 |
| Song, X. 2015 | 47 | 59 | 30 | 59 | 3.5% | 3.79 [1.68, 8.54] | 2015 |
| Liu, D. 2015 | 20 | 46 | 6 | 42 | 2.0% | 4.62 [1.63, 13.09] | 2015 |
| Yan, G. 2016 | 22 | 35 | 12 | 30 | 2.7% | 2.54 [0.93, 6.91] | 2016 |
| Qin,D.2016 | 11 | 32 | 6 | 32 | 2.3% | 2.27 [0.72, 7.16] | 2016 |
| Huang, H. 2017 | 20 | 30 | 11 | 30 | 2.1% | 3.45 [1.19, 9.99] | 2017 |
| Liu, L. 2017 | 13 | 30 | 7 | 30 | 2.3% | 2.51 [0.83, 7.64] | 2017 |
| Shi, W. 2017 | 14 | 30 | 8 | 30 | 2.4% | 2.41 [0.82, 7.10] | 2017 |
| Wang, Y. 2019 | 20 | 45 | 9 | 45 | 2.9% | 3.20 [1.25, 8.17] | 2019 |
| Feng, F.2023 | 11 | 34 | 7 | 34 | 2.7% | 1.84 [0.61, 5.54] | 2023 |
| Lin, W.2023 | 8 | 26 | 5 | 26 | 2.0% | 1.87 [0.52, 6.73] | 2023 |
| **Subtotal (95% CI)** | | **1259** | | **1234** | **91.2%** | **2.62 [2.20, 3.11]** | |
| Total events | 578 | | 312 | | | | |
| Heterogeneity: Chi² = 23.67, df = 34 (P = 0.91); I² = 0% | | | | | | | |
| Test for overall effect: Z = 10.83 (P < 0.00001) | | | | | | | |
| **1.1.2 Others(1-2 times/week or 2-3 time/ week）** | | | | | | | |
| Wang, Y. 2010 | 6 | 24 | 2 | 24 | 0.9% | 3.67 [0.66, 20.42] | 2010 |
| Zheng, S. 2013 | 6 | 31 | 3 | 31 | 1.4% | 2.24 [0.51, 9.91] | 2013 |
| Tang, X. 2018 | 15 | 30 | 10 | 30 | 2.9% | 2.00 [0.70, 5.68] | 2018 |
| Wu, C. 2019 | 8 | 25 | 4 | 25 | 1.6% | 2.47 [0.63, 9.63] | 2019 |
| Jiang, T 2020 | 24 | 30 | 6 | 30 | 0.7% | 16.00 [4.52, 56.70] | 2020 |
| Peng,H.2020 | 12 | 25 | 5 | 25 | 1.5% | 3.69 [1.05, 12.96] | 2020 |
| **Subtotal (95% CI)** | | **165** | | **165** | **8.8%** | **3.66 [2.18, 6.14]** | |
| Total events | 71 | | 30 | | | | |
| Heterogeneity: Chi² = 7.25, df = 5 (P = 0.20); I² = 31% | | | | | | | |
| Test for overall effect: Z = 4.90 (P < 0.00001) | | | | | | | |
| **Total (95% CI)** | | **1424** | | **1399** | **100.0%** | **2.71 [2.30, 3.19]** | |
| Total events | 649 | | 342 | | | | |
| Heterogeneity: Chi² = 32.18, df = 40 (P = 0.81); I² = 0% | | | | | | | |
| Test for overall effect: Z = 11.84 (P < 0.00001) | | | | | | | |
| Test for subgroup differences: Chi² = 1.44, df = 1 (P = 0.23), I² = 30.6% | | | | | | | |

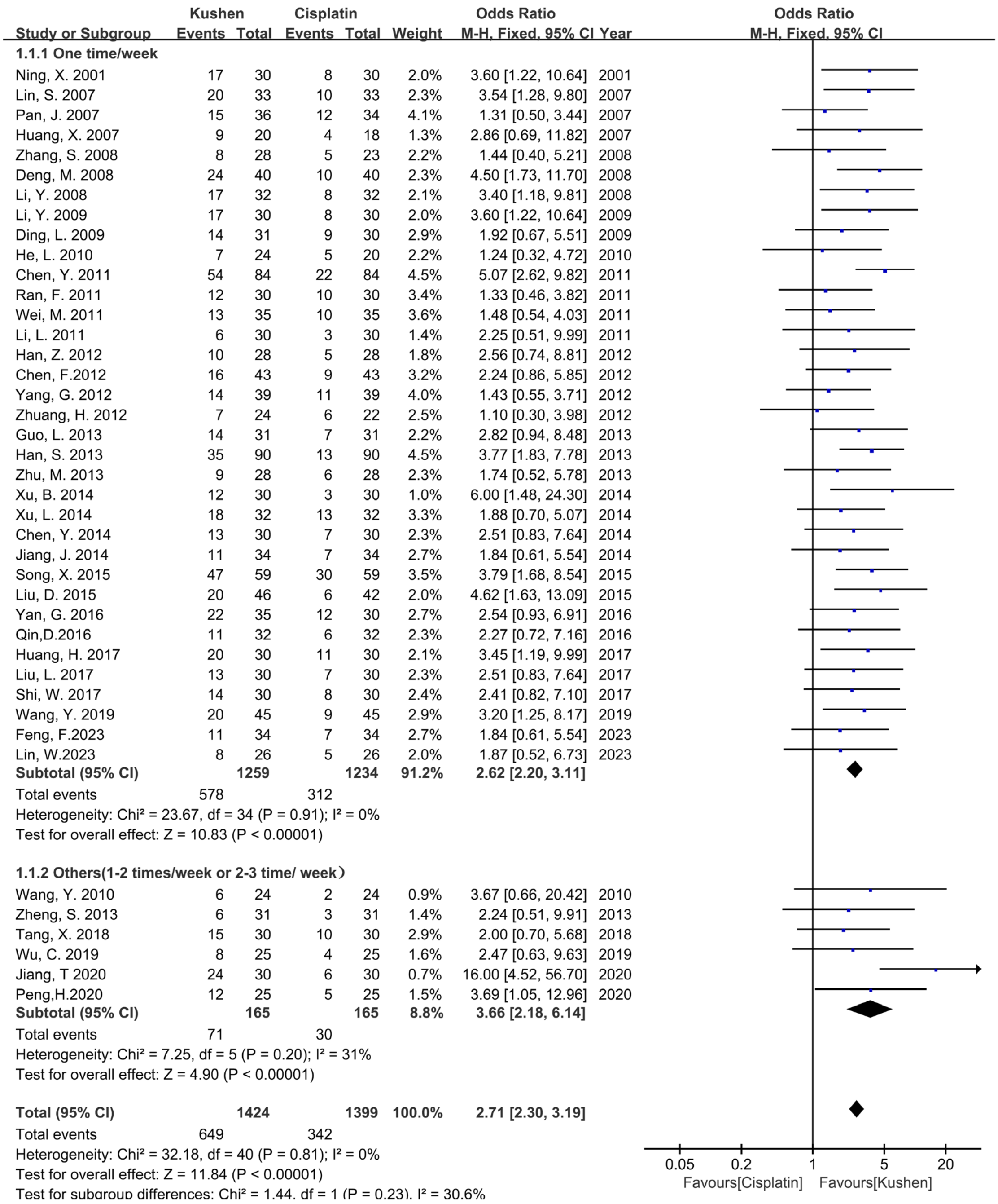


**Figure.S53 Subgroups analysis of complete response via treatment frequency**

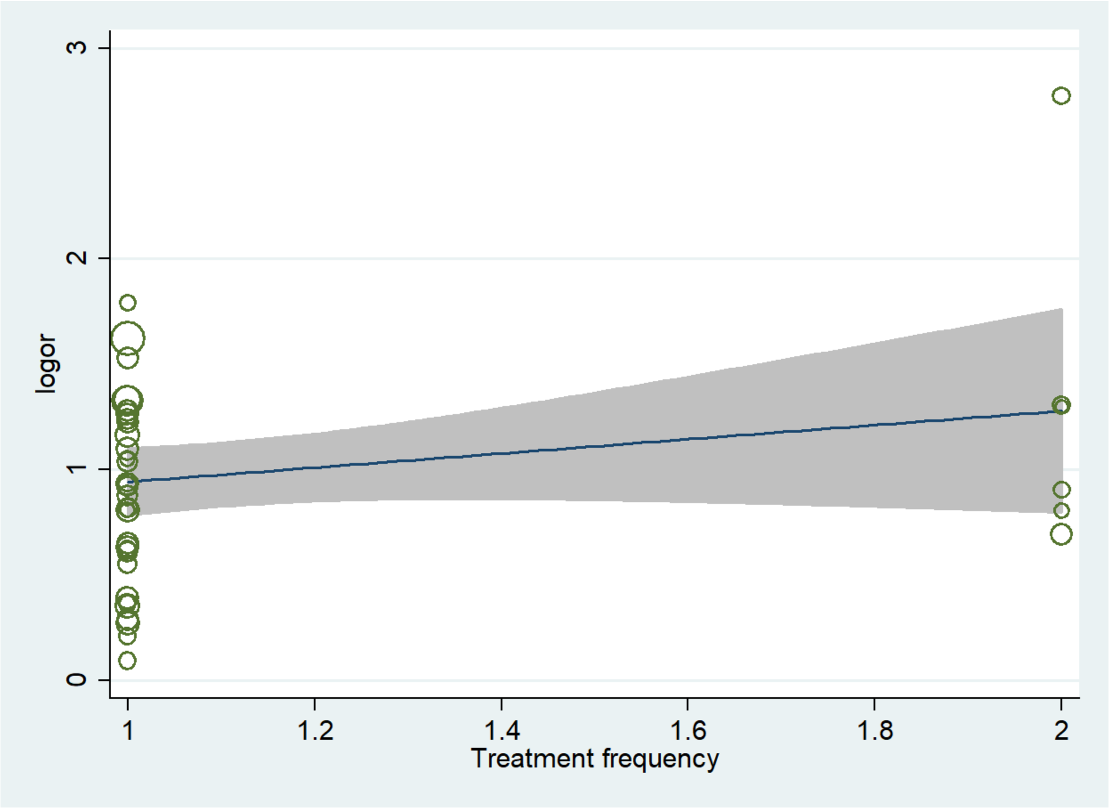


**Figure.S54 Meta-regression of complete response via treatment frequency**

| Study or Subgroup | Kushen Events | Kushen Total | Cisplatin Events | Cisplatin Total | Weight | Odds Ratio M-H, Fixed, 95% CI | Year |
|---|---|---|---|---|---|---|---|
| **1.2.1 One time/week** | | | | | | | |
| Ning, X. 2001 | 5 | 30 | 8 | 30 | 1.4% | 0.55 [0.16, 1.93] | 2001 |
| Pan, J. 2007 | 3 | 36 | 9 | 34 | 1.7% | 0.25 [0.06, 1.03] | 2007 |
| Lin, S. 2007 | 4 | 33 | 11 | 33 | 2.0% | 0.28 [0.08, 0.98] | 2007 |
| Huang, X. 2007 | 3 | 20 | 8 | 18 | 1.5% | 0.22 [0.05, 1.03] | 2007 |
| Li, Y. 2008 | 5 | 32 | 8 | 32 | 1.4% | 0.56 [0.16, 1.93] | 2008 |
| Zhang, S. 2008 | 5 | 28 | 10 | 23 | 1.8% | 0.28 [0.08, 1.01] | 2008 |
| Deng, M. 2008 | 6 | 40 | 12 | 40 | 2.1% | 0.41 [0.14, 1.24] | 2008 |
| Li, Y. 2009 | 5 | 30 | 8 | 30 | 1.4% | 0.55 [0.16, 1.93] | 2009 |
| Ding, L. 2009 | 3 | 31 | 13 | 30 | 2.4% | 0.14 [0.03, 0.56] | 2009 |
| He, L. 2010 | 8 | 24 | 11 | 20 | 1.6% | 0.41 [0.12, 1.39] | 2010 |
| Chen, Y. 2011 | 10 | 84 | 38 | 84 | 6.8% | 0.16 [0.07, 0.36] | 2011 |
| Wei, M. 2011 | 6 | 35 | 11 | 35 | 1.9% | 0.45 [0.15, 1.40] | 2011 |
| Li, L. 2011 | 7 | 30 | 17 | 30 | 2.6% | 0.23 [0.08, 0.71] | 2011 |
| Ran, F. 2011 | 4 | 30 | 12 | 30 | 2.1% | 0.23 [0.06, 0.83] | 2011 |
| Chen, F.2012 | 7 | 43 | 20 | 43 | 3.4% | 0.22 [0.08, 0.61] | 2012 |
| Yang, G. 2012 | 6 | 39 | 16 | 39 | 2.7% | 0.26 [0.09, 0.77] | 2012 |
| Han, Z. 2012 | 5 | 28 | 13 | 28 | 2.2% | 0.25 [0.07, 0.85] | 2012 |
| Zhuang, H. 2012 | 6 | 24 | 8 | 22 | 1.3% | 0.58 [0.16, 2.07] | 2012 |
| Han, S. 2013 | 11 | 90 | 40 | 90 | 7.1% | 0.17 [0.08, 0.37] | 2013 |
| Zhu, M. 2013 | 5 | 28 | 9 | 28 | 1.5% | 0.46 [0.13, 1.60] | 2013 |
| Guo, L. 2013 | 7 | 31 | 15 | 31 | 2.4% | 0.31 [0.10, 0.93] | 2013 |
| Jiang, J. 2014 | 5 | 34 | 13 | 34 | 2.3% | 0.28 [0.09, 0.90] | 2014 |
| Chen, Y. 2014 | 7 | 30 | 14 | 30 | 2.2% | 0.35 [0.11, 1.05] | 2014 |
| Xu, L. 2014 | 7 | 32 | 15 | 32 | 2.4% | 0.32 [0.11, 0.94] | 2014 |
| Xu, B. 2014 | 11 | 30 | 17 | 30 | 2.2% | 0.44 [0.16, 1.25] | 2014 |
| Liu, D. 2015 | 8 | 46 | 21 | 42 | 3.7% | 0.21 [0.08, 0.56] | 2015 |
| Song, X. 2015 | 5 | 59 | 19 | 59 | 3.5% | 0.19 [0.07, 0.57] | 2015 |
| Qin,D.2016 | 2 | 32 | 15 | 32 | 2.9% | 0.08 [0.02, 0.37] | 2016 |
| Yan, G. 2016 | 4 | 35 | 11 | 30 | 2.1% | 0.22 [0.06, 0.80] | 2016 |
| Liu, L. 2017 | 6 | 30 | 14 | 30 | 2.3% | 0.29 [0.09, 0.90] | 2017 |
| Huang, H. 2017 | 3 | 30 | 11 | 30 | 2.0% | 0.19 [0.05, 0.78] | 2017 |
| Shi, W. 2017 | 6 | 30 | 13 | 30 | 2.1% | 0.33 [0.10, 1.03] | 2017 |
| Wang, Y. 2019 | 8 | 45 | 20 | 45 | 3.3% | 0.27 [0.10, 0.71] | 2019 |
| Lin, W.2023 | 7 | 26 | 15 | 26 | 2.2% | 0.27 [0.08, 0.87] | 2023 |
| Feng, F.2023 | 7 | 34 | 15 | 34 | 2.4% | 0.33 [0.11, 0.96] | 2023 |
| **Subtotal (95% CI)** | | **1259** | | **1234** | **86.7%** | **0.27 [0.23, 0.33]** | |
| Total events | 207 | | 510 | | | | |
| Heterogeneity: Chi² = 16.54, df = 34 (P = 0.99); I² = 0% | | | | | | | |
| Test for overall effect: Z = 13.43 (P < 0.00001) | | | | | | | |
| **1.2.2 Others(1-2 times/week or 2-3 time/ week)** | | | | | | | |
| Wang, Y. 2010 | 2 | 24 | 10 | 24 | 1.9% | 0.13 [0.02, 0.67] | 2010 |
| Zheng, S. 2013 | 6 | 31 | 16 | 31 | 2.6% | 0.23 [0.07, 0.70] | 2013 |
| Tang, X. 2018 | 4 | 30 | 11 | 30 | 1.9% | 0.27 [0.07, 0.96] | 2018 |
| Wu, C. 2019 | 7 | 25 | 15 | 25 | 2.2% | 0.26 [0.08, 0.85] | 2019 |
| Jiang, T 2020 | 2 | 30 | 13 | 30 | 2.5% | 0.09 [0.02, 0.47] | 2020 |
| Peng,H.2020 | 7 | 25 | 15 | 25 | 2.2% | 0.26 [0.08, 0.85] | 2020 |
| **Subtotal (95% CI)** | | **165** | | **165** | **13.3%** | **0.20 [0.12, 0.34]** | |
| Total events | 28 | | 80 | | | | |
| Heterogeneity: Chi² = 1.72, df = 5 (P = 0.89); I² = 0% | | | | | | | |
| Test for overall effect: Z = 5.94 (P < 0.00001) | | | | | | | |
| **Total (95% CI)** | | **1424** | | **1399** | **100.0%** | **0.26 [0.22, 0.32]** | |
| Total events | 235 | | 590 | | | | |
| Heterogeneity: Chi² = 19.19, df = 40 (P = 1.00); I² = 0% | | | | | | | |
| Test for overall effect: Z = 14.66 (P < 0.00001) | | | | | | | |
| Test for subgroup differences: Chi² = 1.05, df = 1 (P = 0.31), I² = 4.6% | | | | | | | |

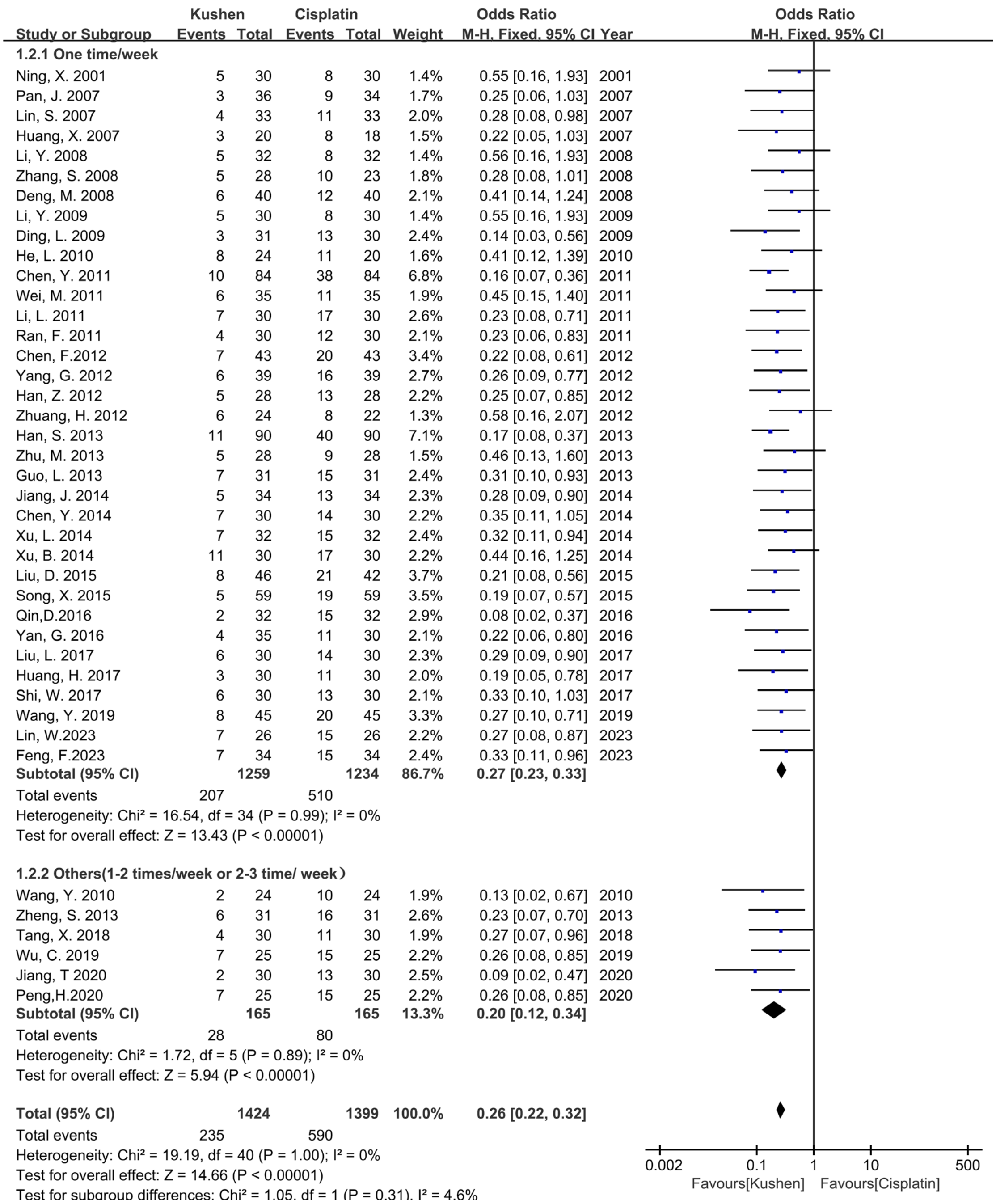


**Figure.S55 Subgroups analysis of pleurodesis failure via treatment frequency**

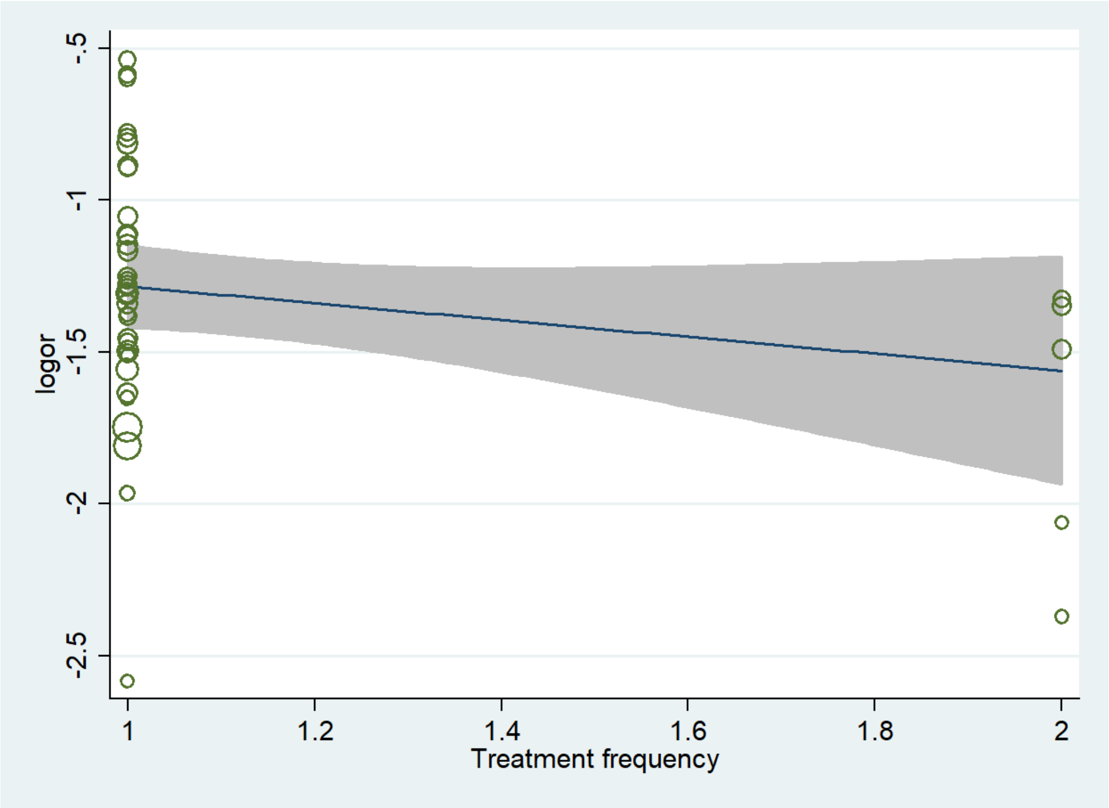


**Figure.S56 Meta-regression of pleurodesis failure via treatment frequency**

| Study or Subgroup | Kushen Events | Kushen Total | Cisplatin Events | Cisplatin Total | Weight | Odds Ratio M-H, Fixed, 95% CI | Year |
|---|---|---|---|---|---|---|---|
| **1.1.1 Two to four times** | | | | | | | |
| Ning, X. 2001 | 17 | 30 | 8 | 30 | 2.0% | 3.60 [1.22, 10.64] | 2001 |
| Pan, J. 2007 | 15 | 36 | 12 | 34 | 4.1% | 1.31 [0.50, 3.44] | 2007 |
| Zhang, S. 2008 | 8 | 28 | 5 | 23 | 2.2% | 1.44 [0.40, 5.21] | 2008 |
| Li, Y. 2008 | 17 | 32 | 8 | 32 | 2.1% | 3.40 [1.18, 9.81] | 2008 |
| Ding, L. 2009 | 14 | 31 | 9 | 30 | 2.9% | 1.92 [0.67, 5.51] | 2009 |
| He, L. 2010 | 7 | 24 | 5 | 20 | 2.2% | 1.24 [0.32, 4.72] | 2010 |
| Ran, F. 2011 | 12 | 30 | 10 | 30 | 3.4% | 1.33 [0.46, 3.82] | 2011 |
| Wei, M. 2011 | 13 | 35 | 10 | 35 | 3.6% | 1.48 [0.54, 4.03] | 2011 |
| Li, L. 2011 | 6 | 30 | 3 | 30 | 1.4% | 2.25 [0.51, 9.99] | 2011 |
| Han, Z. 2012 | 10 | 28 | 5 | 28 | 1.8% | 2.56 [0.74, 8.81] | 2012 |
| Chen, F.2012 | 16 | 43 | 9 | 43 | 3.2% | 2.24 [0.86, 5.85] | 2012 |
| Yang, G. 2012 | 14 | 39 | 11 | 39 | 4.0% | 1.43 [0.55, 3.71] | 2012 |
| Guo, L. 2013 | 14 | 31 | 7 | 31 | 2.2% | 2.82 [0.94, 8.48] | 2013 |
| Han, S. 2013 | 35 | 90 | 13 | 90 | 4.5% | 3.77 [1.83, 7.78] | 2013 |
| Chen, Y. 2014 | 13 | 30 | 7 | 30 | 2.3% | 2.51 [0.83, 7.64] | 2014 |
| Xu, B. 2014 | 12 | 30 | 3 | 30 | 1.0% | 6.00 [1.48, 24.30] | 2014 |
| Song, X. 2015 | 47 | 59 | 30 | 59 | 3.5% | 3.79 [1.68, 8.54] | 2015 |
| Liu, D. 2015 | 20 | 46 | 6 | 42 | 2.0% | 4.62 [1.63, 13.09] | 2015 |
| Yan, G. 2016 | 22 | 35 | 12 | 30 | 2.7% | 2.54 [0.93, 6.91] | 2016 |
| Huang, H. 2017 | 20 | 30 | 11 | 30 | 2.1% | 3.45 [1.19, 9.99] | 2017 |
| Liu, L. 2017 | 13 | 30 | 7 | 30 | 2.3% | 2.51 [0.83, 7.64] | 2017 |
| Shi, W. 2017 | 14 | 30 | 8 | 30 | 2.4% | 2.41 [0.82, 7.10] | 2017 |
| Wang, Y. 2019 | 20 | 45 | 9 | 45 | 2.9% | 3.20 [1.25, 8.17] | 2019 |
| Feng, F.2023 | 11 | 34 | 7 | 34 | 2.7% | 1.84 [0.61, 5.54] | 2023 |
| Lin, W.2023 | 8 | 26 | 5 | 26 | 2.0% | 1.87 [0.52, 6.73] | 2023 |
| **Subtotal (95% CI)** | | **902** | | **881** | **65.7%** | **2.49 [2.02, 3.06]** | |
| Total events | 398 | | 220 | | | | |
| Heterogeneity: Chi² = 14.53, df = 24 (P = 0.93); I² = 0% | | | | | | | |
| Test for overall effect: Z = 8.59 (P < 0.00001) | | | | | | | |
| **1.1.2 Others (>4 times or unclear）** | | | | | | | |
| Huang, X. 2007 | 9 | 20 | 4 | 18 | 1.3% | 2.86 [0.69, 11.82] | 2007 |
| Lin, S. 2007 | 20 | 33 | 10 | 33 | 2.3% | 3.54 [1.28, 9.80] | 2007 |
| Deng, M. 2008 | 24 | 40 | 10 | 40 | 2.3% | 4.50 [1.73, 11.70] | 2008 |
| Li, Y. 2009 | 17 | 30 | 8 | 30 | 2.0% | 3.60 [1.22, 10.64] | 2009 |
| Wang, Y. 2010 | 6 | 24 | 2 | 24 | 0.9% | 3.67 [0.66, 20.42] | 2010 |
| Chen, Y. 2011 | 54 | 84 | 22 | 84 | 4.5% | 5.07 [2.62, 9.82] | 2011 |
| Zhuang, H. 2012 | 7 | 24 | 6 | 22 | 2.5% | 1.10 [0.30, 3.98] | 2012 |
| Zhu, M. 2013 | 9 | 28 | 6 | 28 | 2.3% | 1.74 [0.52, 5.78] | 2013 |
| Zheng, S. 2013 | 6 | 31 | 3 | 31 | 1.4% | 2.24 [0.51, 9.91] | 2013 |
| Jiang, J. 2014 | 11 | 34 | 7 | 34 | 2.7% | 1.84 [0.61, 5.54] | 2014 |
| Xu, L. 2014 | 18 | 32 | 13 | 32 | 3.3% | 1.88 [0.70, 5.07] | 2014 |
| Qin,D.2016 | 11 | 32 | 6 | 32 | 2.3% | 2.27 [0.72, 7.16] | 2016 |
| Tang, X. 2018 | 15 | 30 | 10 | 30 | 2.9% | 2.00 [0.70, 5.68] | 2018 |
| Wu, C. 2019 | 8 | 25 | 4 | 25 | 1.6% | 2.47 [0.63, 9.63] | 2019 |
| Jiang, T 2020 | 24 | 30 | 6 | 30 | 0.7% | 16.00 [4.52, 56.70] | 2020 |
| Peng,H.2020 | 12 | 25 | 5 | 25 | 1.5% | 3.69 [1.05, 12.96] | 2020 |
| **Subtotal (95% CI)** | | **522** | | **518** | **34.3%** | **3.13 [2.39, 4.11]** | |
| Total events | 251 | | 122 | | | | |
| Heterogeneity: Chi² = 15.93, df = 15 (P = 0.39); I² = 6% | | | | | | | |
| Test for overall effect: Z = 8.25 (P < 0.00001) | | | | | | | |
| **Total (95% CI)** | | **1424** | | **1399** | **100.0%** | **2.71 [2.30, 3.19]** | |
| Total events | 649 | | 342 | | | | |
| Heterogeneity: Chi² = 32.18, df = 40 (P = 0.81); I² = 0% | | | | | | | |
| Test for overall effect: Z = 11.84 (P < 0.00001) | | | | | | | |
| Test for subgroup differences: Chi² = 1.76, df = 1 (P = 0.18), I² = 43.3% | | | | | | | |

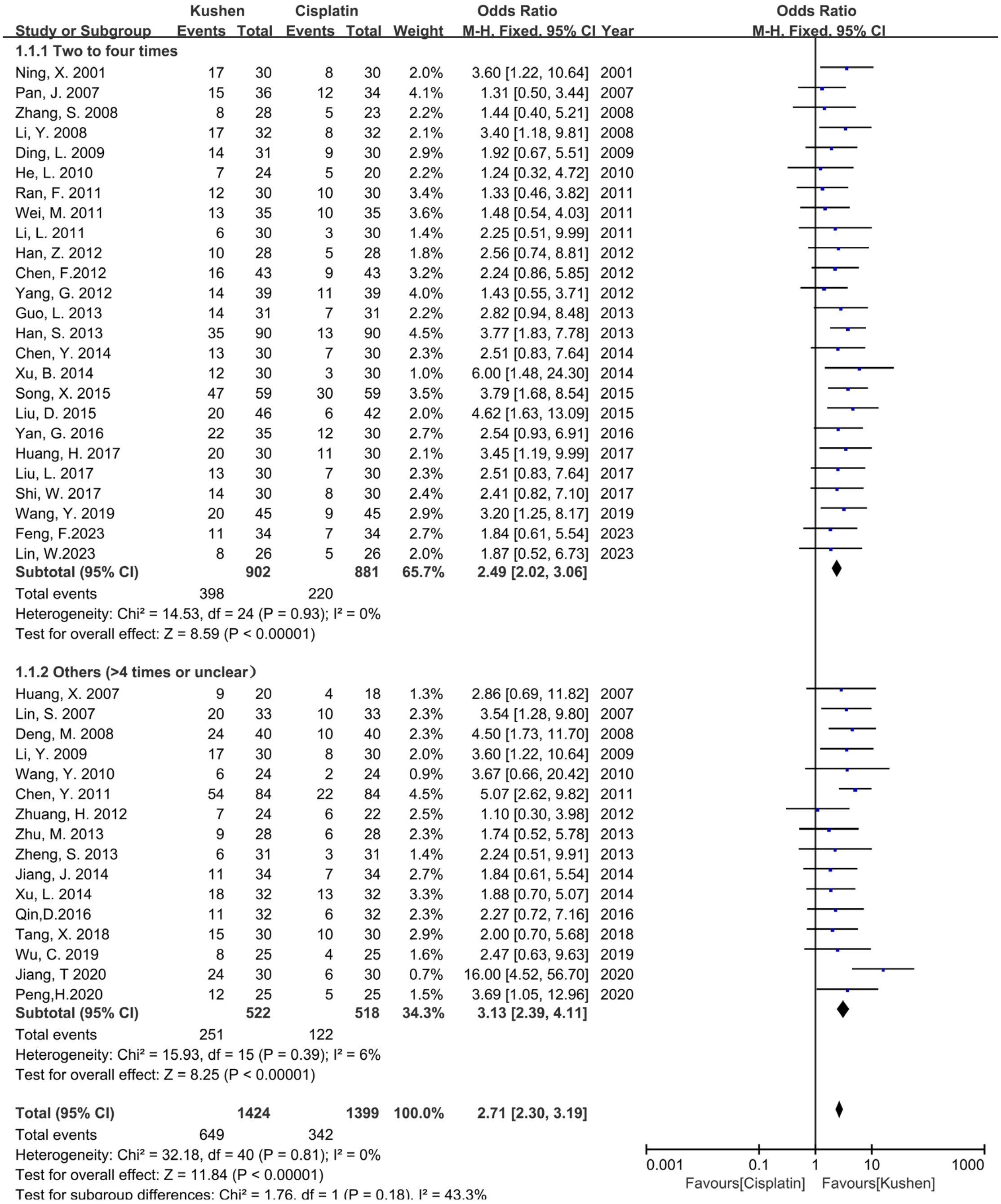


**Figure.S57 Subgroups analysis of complete response via treatment times**

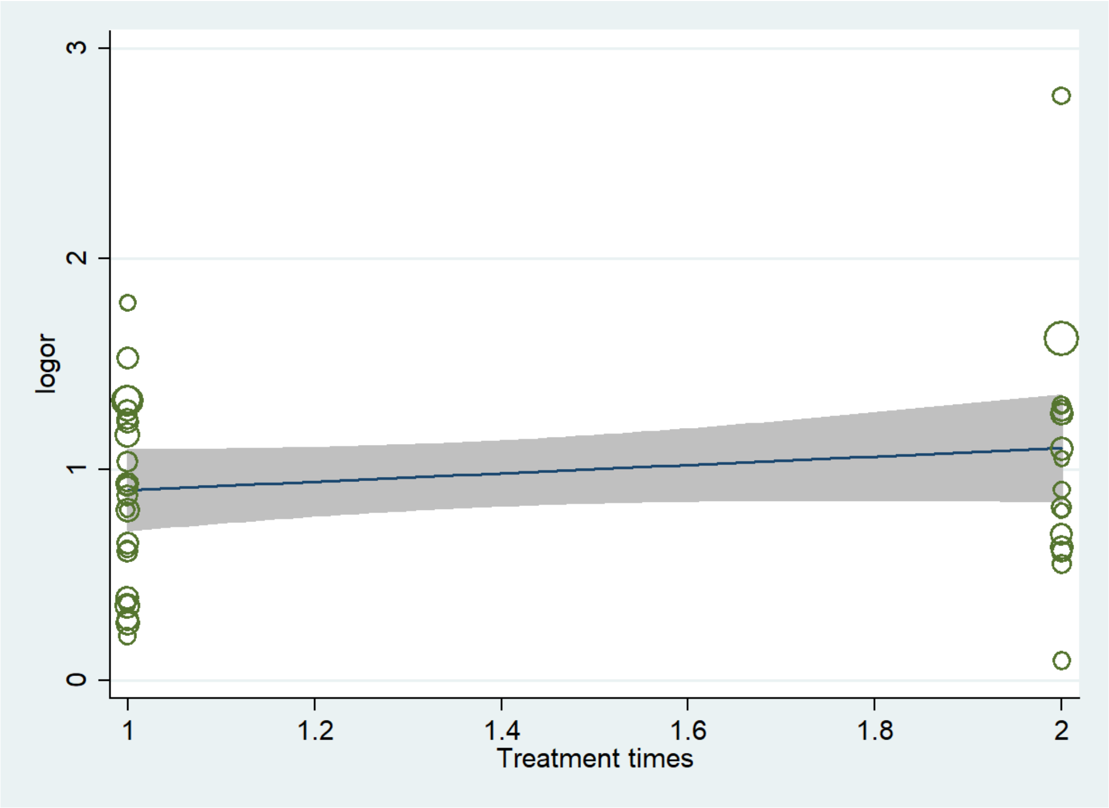


**Figure.S58 Meta-regression of complete response via treatment times**

| Study or Subgroup | Kushen Events | Kushen Total | Cisplatin Events | Cisplatin Total | Weight | Odds Ratio M-H, Fixed, 95% CI | Year |
|---|---|---|---|---|---|---|---|
| **1.2.1 Two to four times** | | | | | | | |
| Ning, X. 2001 | 5 | 30 | 8 | 30 | 1.4% | 0.55 [0.16, 1.93] | 2001 |
| Pan, J. 2007 | 3 | 36 | 9 | 34 | 1.7% | 0.25 [0.06, 1.03] | 2007 |
| Li, Y. 2008 | 5 | 32 | 8 | 32 | 1.4% | 0.56 [0.16, 1.93] | 2008 |
| Zhang, S. 2008 | 5 | 28 | 10 | 23 | 1.8% | 0.28 [0.08, 1.01] | 2008 |
| Ding, L. 2009 | 3 | 31 | 13 | 30 | 2.4% | 0.14 [0.03, 0.56] | 2009 |
| He, L. 2010 | 8 | 24 | 11 | 20 | 1.6% | 0.41 [0.12, 1.39] | 2010 |
| Wei, M. 2011 | 6 | 35 | 11 | 35 | 1.9% | 0.45 [0.15, 1.40] | 2011 |
| Li, L. 2011 | 7 | 30 | 17 | 30 | 2.6% | 0.23 [0.08, 0.71] | 2011 |
| Ran, F. 2011 | 4 | 30 | 12 | 30 | 2.1% | 0.23 [0.06, 0.83] | 2011 |
| Chen, F.2012 | 7 | 43 | 20 | 43 | 3.4% | 0.22 [0.08, 0.61] | 2012 |
| Yang, G. 2012 | 6 | 39 | 16 | 39 | 2.7% | 0.26 [0.09, 0.77] | 2012 |
| Han, Z. 2012 | 5 | 28 | 13 | 28 | 2.2% | 0.25 [0.07, 0.85] | 2012 |
| Han, S. 2013 | 11 | 90 | 40 | 90 | 7.1% | 0.17 [0.08, 0.37] | 2013 |
| Guo, L. 2013 | 7 | 31 | 15 | 31 | 2.4% | 0.31 [0.10, 0.93] | 2013 |
| Chen, Y. 2014 | 7 | 30 | 14 | 30 | 2.2% | 0.35 [0.11, 1.05] | 2014 |
| Xu, B. 2014 | 11 | 30 | 17 | 30 | 2.2% | 0.44 [0.16, 1.25] | 2014 |
| Liu, D. 2015 | 8 | 46 | 21 | 42 | 3.7% | 0.21 [0.08, 0.56] | 2015 |
| Song, X. 2015 | 5 | 59 | 19 | 59 | 3.5% | 0.19 [0.07, 0.57] | 2015 |
| Yan, G. 2016 | 4 | 35 | 11 | 30 | 2.1% | 0.22 [0.06, 0.80] | 2016 |
| Liu, L. 2017 | 6 | 30 | 14 | 30 | 2.3% | 0.29 [0.09, 0.90] | 2017 |
| Huang, H. 2017 | 3 | 30 | 11 | 30 | 2.0% | 0.19 [0.05, 0.78] | 2017 |
| Shi, W. 2017 | 6 | 30 | 13 | 30 | 2.1% | 0.33 [0.10, 1.03] | 2017 |
| Wang, Y. 2019 | 8 | 45 | 20 | 45 | 3.3% | 0.27 [0.10, 0.71] | 2019 |
| Feng, F.2023 | 7 | 34 | 15 | 34 | 2.4% | 0.33 [0.11, 0.96] | 2023 |
| Lin, W.2023 | 7 | 26 | 15 | 26 | 2.2% | 0.27 [0.08, 0.87] | 2023 |
| **Subtotal (95% CI)** | | **902** | | **881** | **62.8%** | **0.27 [0.22, 0.34]** | |
| Total events | 154 | | 373 | | | | |
| Heterogeneity: Chi² = 8.49, df = 24 (P = 1.00); I² = 0% | | | | | | | |
| Test for overall effect: Z = 11.47 (P < 0.00001) | | | | | | | |
| **1.2.2 Others (>4 times or unclear)** | | | | | | | |
| Huang, X. 2007 | 3 | 20 | 8 | 18 | 1.5% | 0.22 [0.05, 1.03] | 2007 |
| Lin, S. 2007 | 4 | 33 | 11 | 33 | 2.0% | 0.28 [0.08, 0.98] | 2007 |
| Deng, M. 2008 | 6 | 40 | 12 | 40 | 2.1% | 0.41 [0.14, 1.24] | 2008 |
| Li, Y. 2009 | 5 | 30 | 8 | 30 | 1.4% | 0.55 [0.16, 1.93] | 2009 |
| Wang, Y. 2010 | 2 | 24 | 10 | 24 | 1.9% | 0.13 [0.02, 0.67] | 2010 |
| Chen, Y. 2011 | 10 | 84 | 38 | 84 | 6.8% | 0.16 [0.07, 0.36] | 2011 |
| Zhuang, H. 2012 | 6 | 24 | 8 | 22 | 1.3% | 0.58 [0.16, 2.07] | 2012 |
| Zheng, S. 2013 | 6 | 31 | 16 | 31 | 2.6% | 0.23 [0.07, 0.70] | 2013 |
| Zhu, M. 2013 | 5 | 28 | 9 | 28 | 1.5% | 0.46 [0.13, 1.60] | 2013 |
| Jiang, J. 2014 | 5 | 34 | 13 | 34 | 2.3% | 0.28 [0.09, 0.90] | 2014 |
| Xu, L. 2014 | 7 | 32 | 15 | 32 | 2.4% | 0.32 [0.11, 0.94] | 2014 |
| Qin,D.2016 | 2 | 32 | 15 | 32 | 2.9% | 0.08 [0.02, 0.37] | 2016 |
| Tang, X. 2018 | 4 | 30 | 11 | 30 | 1.9% | 0.27 [0.07, 0.96] | 2018 |
| Wu, C. 2019 | 7 | 25 | 15 | 25 | 2.2% | 0.26 [0.08, 0.85] | 2019 |
| Peng,H.2020 | 7 | 25 | 15 | 25 | 2.2% | 0.26 [0.08, 0.85] | 2020 |
| Jiang, T 2020 | 2 | 30 | 13 | 30 | 2.5% | 0.09 [0.02, 0.47] | 2020 |
| **Subtotal (95% CI)** | | **522** | | **518** | **37.2%** | **0.25 [0.19, 0.34]** | |
| Total events | 81 | | 217 | | | | |
| Heterogeneity: Chi² = 10.60, df = 15 (P = 0.78); I² = 0% | | | | | | | |
| Test for overall effect: Z = 9.13 (P < 0.00001) | | | | | | | |
| **Total (95% CI)** | | **1424** | | **1399** | **100.0%** | **0.26 [0.22, 0.32]** | |
| Total events | 235 | | 590 | | | | |
| Heterogeneity: Chi² = 19.19, df = 40 (P = 1.00); I² = 0% | | | | | | | |
| Test for overall effect: Z = 14.66 (P < 0.00001) | | | | | | | |
| Test for subgroup differences: Chi² = 0.19, df = 1 (P = 0.66), I² = 0% | | | | | | | |

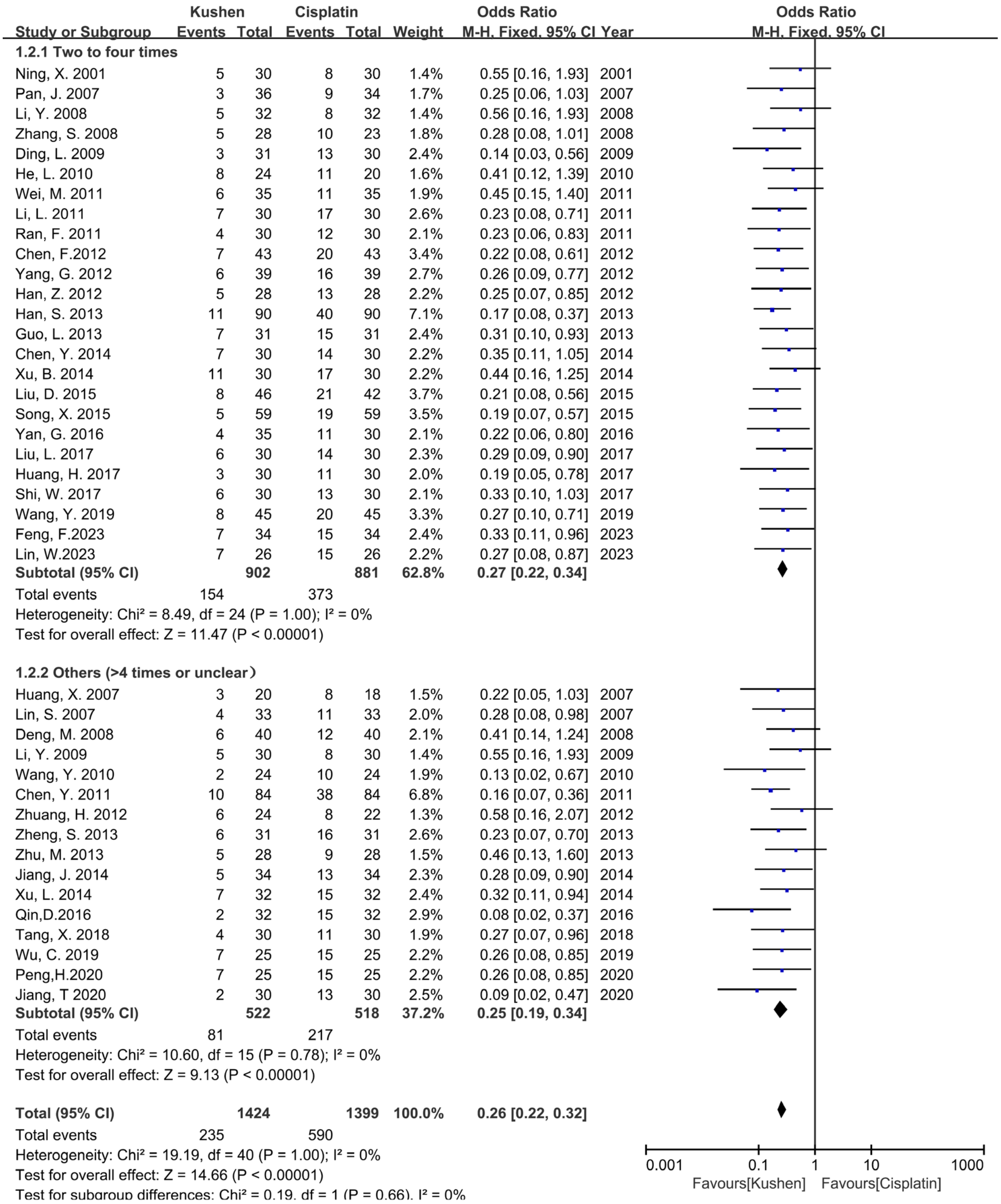


**Figure.S59 Subgroups analysis of pleurodesis failure via treatment times**

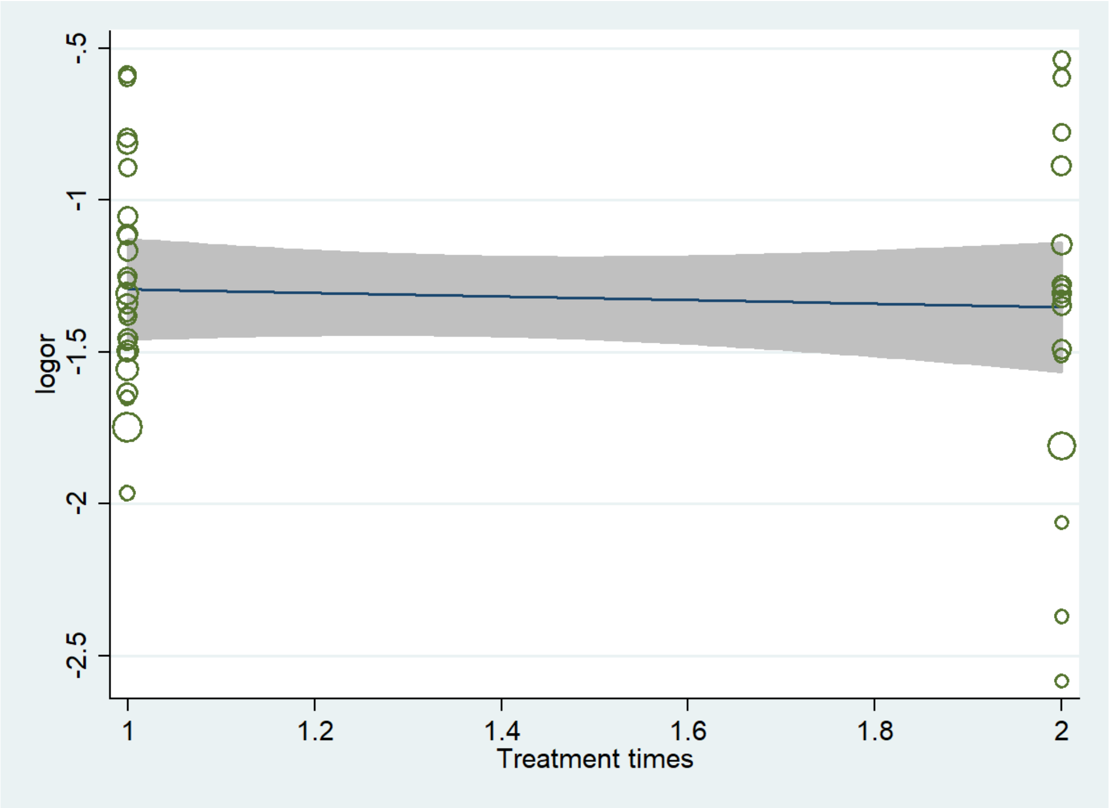


**Figure.S60 Meta-regression of pleurodesis failure via treatment times**

| Study or Subgroup | Kushen Events | Kushen Total | Cisplatin Events | Cisplatin Total | Weight | Odds Ratio M-H, Fixed, 95% CI | Year |
|---|---|---|---|---|---|---|---|
| **1.1.1 Cisplatin (20-30 mg each time)** | | | | | | | |
| Zhang, S. 2008 | 8 | 28 | 5 | 23 | 2.2% | 1.44 [0.40, 5.21] | 2008 |
| Ding, L. 2009 | 14 | 31 | 9 | 30 | 2.9% | 1.92 [0.67, 5.51] | 2009 |
| Zhuang, H. 2012 | 7 | 24 | 6 | 22 | 2.5% | 1.10 [0.30, 3.98] | 2012 |
| Zheng, S. 2013 | 6 | 31 | 3 | 31 | 1.4% | 2.24 [0.51, 9.91] | 2013 |
| Han, S. 2013 | 35 | 90 | 13 | 90 | 4.5% | 3.77 [1.83, 7.78] | 2013 |
| Peng,H.2020 | 12 | 25 | 5 | 25 | 1.5% | 3.69 [1.05, 12.96] | 2020 |
| **Subtotal (95% CI)** | | **229** | | **221** | **15.1%** | **2.47 [1.60, 3.83]** | |
| Total events | 82 | | 41 | | | | |
| Heterogeneity: Chi² = 4.14, df = 5 (P = 0.53); I² = 0% | | | | | | | |
| Test for overall effect: Z = 4.06 (P < 0.0001) | | | | | | | |
| **1.1.2 Cisplatin (40-50 mg each time)** | | | | | | | |
| Pan, J. 2007 | 15 | 36 | 12 | 34 | 4.1% | 1.31 [0.50, 3.44] | 2007 |
| Huang, X. 2007 | 9 | 20 | 4 | 18 | 1.3% | 2.86 [0.69, 11.82] | 2007 |
| Li, Y. 2009 | 17 | 30 | 8 | 30 | 2.0% | 3.60 [1.22, 10.64] | 2009 |
| Wang, Y. 2010 | 6 | 24 | 2 | 24 | 0.9% | 3.67 [0.66, 20.42] | 2010 |
| He, L. 2010 | 7 | 24 | 5 | 20 | 2.2% | 1.24 [0.32, 4.72] | 2010 |
| Wei, M. 2011 | 13 | 35 | 10 | 35 | 3.6% | 1.48 [0.54, 4.03] | 2011 |
| Guo, L. 2013 | 14 | 31 | 7 | 31 | 2.2% | 2.82 [0.94, 8.48] | 2013 |
| Chen, Y. 2014 | 13 | 30 | 7 | 30 | 2.3% | 2.51 [0.83, 7.64] | 2014 |
| Xu, L. 2014 | 18 | 32 | 13 | 32 | 3.3% | 1.88 [0.70, 5.07] | 2014 |
| Song, X. 2015 | 47 | 59 | 30 | 59 | 3.5% | 3.79 [1.68, 8.54] | 2015 |
| Yan, G. 2016 | 22 | 35 | 12 | 30 | 2.7% | 2.54 [0.93, 6.91] | 2016 |
| Huang, H. 2017 | 20 | 30 | 11 | 30 | 2.1% | 3.45 [1.19, 9.99] | 2017 |
| Liu, L. 2017 | 13 | 30 | 7 | 30 | 2.3% | 2.51 [0.83, 7.64] | 2017 |
| Shi, W. 2017 | 14 | 30 | 8 | 30 | 2.4% | 2.41 [0.82, 7.10] | 2017 |
| Tang, X. 2018 | 15 | 30 | 10 | 30 | 2.9% | 2.00 [0.70, 5.68] | 2018 |
| Jiang, T 2020 | 24 | 30 | 6 | 30 | 0.7% | 16.00 [4.52, 56.70] | 2020 |
| Lin, W.2023 | 8 | 26 | 5 | 26 | 2.0% | 1.87 [0.52, 6.73] | 2023 |
| Feng, F.2023 | 11 | 34 | 7 | 34 | 2.7% | 1.84 [0.61, 5.54] | 2023 |
| **Subtotal (95% CI)** | | **566** | | **553** | **43.1%** | **2.55 [1.98, 3.29]** | |
| Total events | 286 | | 164 | | | | |
| Heterogeneity: Chi² = 15.16, df = 17 (P = 0.58); I² = 0% | | | | | | | |
| Test for overall effect: Z = 7.23 (P < 0.00001) | | | | | | | |
| **1.1.3 Cisplatin (60-80 mg each time)** | | | | | | | |
| Ning, X. 2001 | 17 | 30 | 8 | 30 | 2.0% | 3.60 [1.22, 10.64] | 2001 |
| Lin, S. 2007 | 20 | 33 | 10 | 33 | 2.3% | 3.54 [1.28, 9.80] | 2007 |
| Deng, M. 2008 | 24 | 40 | 10 | 40 | 2.3% | 4.50 [1.73, 11.70] | 2008 |
| Li, Y. 2008 | 17 | 32 | 8 | 32 | 2.1% | 3.40 [1.18, 9.81] | 2008 |
| Ran, F. 2011 | 12 | 30 | 10 | 30 | 3.4% | 1.33 [0.46, 3.82] | 2011 |
| Li, L. 2011 | 6 | 30 | 3 | 30 | 1.4% | 2.25 [0.51, 9.99] | 2011 |
| Chen, F.2012 | 16 | 43 | 9 | 43 | 3.2% | 2.24 [0.86, 5.85] | 2012 |
| Zhu, M. 2013 | 9 | 28 | 6 | 28 | 2.3% | 1.74 [0.52, 5.78] | 2013 |
| Xu, B. 2014 | 12 | 30 | 3 | 30 | 1.0% | 6.00 [1.48, 24.30] | 2014 |
| Jiang, J. 2014 | 11 | 34 | 7 | 34 | 2.7% | 1.84 [0.61, 5.54] | 2014 |
| Qin,D.2016 | 11 | 32 | 6 | 32 | 2.3% | 2.27 [0.72, 7.16] | 2016 |
| **Subtotal (95% CI)** | | **362** | | **362** | **25.0%** | **2.71 [1.95, 3.77]** | |
| Total events | 155 | | 80 | | | | |
| Heterogeneity: Chi² = 6.07, df = 10 (P = 0.81); I² = 0% | | | | | | | |
| Test for overall effect: Z = 5.94 (P < 0.00001) | | | | | | | |
| **1.1.4 Cisplatin (others)** | | | | | | | |
| Chen, Y. 2011 | 54 | 84 | 22 | 84 | 4.5% | 5.07 [2.62, 9.82] | 2011 |
| Han, Z. 2012 | 10 | 28 | 5 | 28 | 1.8% | 2.56 [0.74, 8.81] | 2012 |
| Yang, G. 2012 | 14 | 39 | 11 | 39 | 4.0% | 1.43 [0.55, 3.71] | 2012 |
| Liu, D. 2015 | 20 | 46 | 6 | 42 | 2.0% | 4.62 [1.63, 13.09] | 2015 |
| Wang, Y. 2019 | 20 | 45 | 9 | 45 | 2.9% | 3.20 [1.25, 8.17] | 2019 |
| Wu, C. 2019 | 8 | 25 | 4 | 25 | 1.6% | 2.47 [0.63, 9.63] | 2019 |
| **Subtotal (95% CI)** | | **267** | | **263** | **16.8%** | **3.31 [2.25, 4.85]** | |
| Total events | 126 | | 57 | | | | |
| Heterogeneity: Chi² = 5.33, df = 5 (P = 0.38); I² = 6% | | | | | | | |
| Test for overall effect: Z = 6.11 (P < 0.00001) | | | | | | | |
| **Total (95% CI)** | | **1424** | | **1399** | **100.0%** | **2.71 [2.30, 3.19]** | |
| Total events | 649 | | 342 | | | | |
| Heterogeneity: Chi² = 32.18, df = 40 (P = 0.81); I² = 0% | | | | | | | |
| Test for overall effect: Z = 11.84 (P < 0.00001) | | | | | | | |
| Test for subgroup differences: Chi² = 1.42, df = 3 (P = 0.70), I² = 0% | | | | | | | |

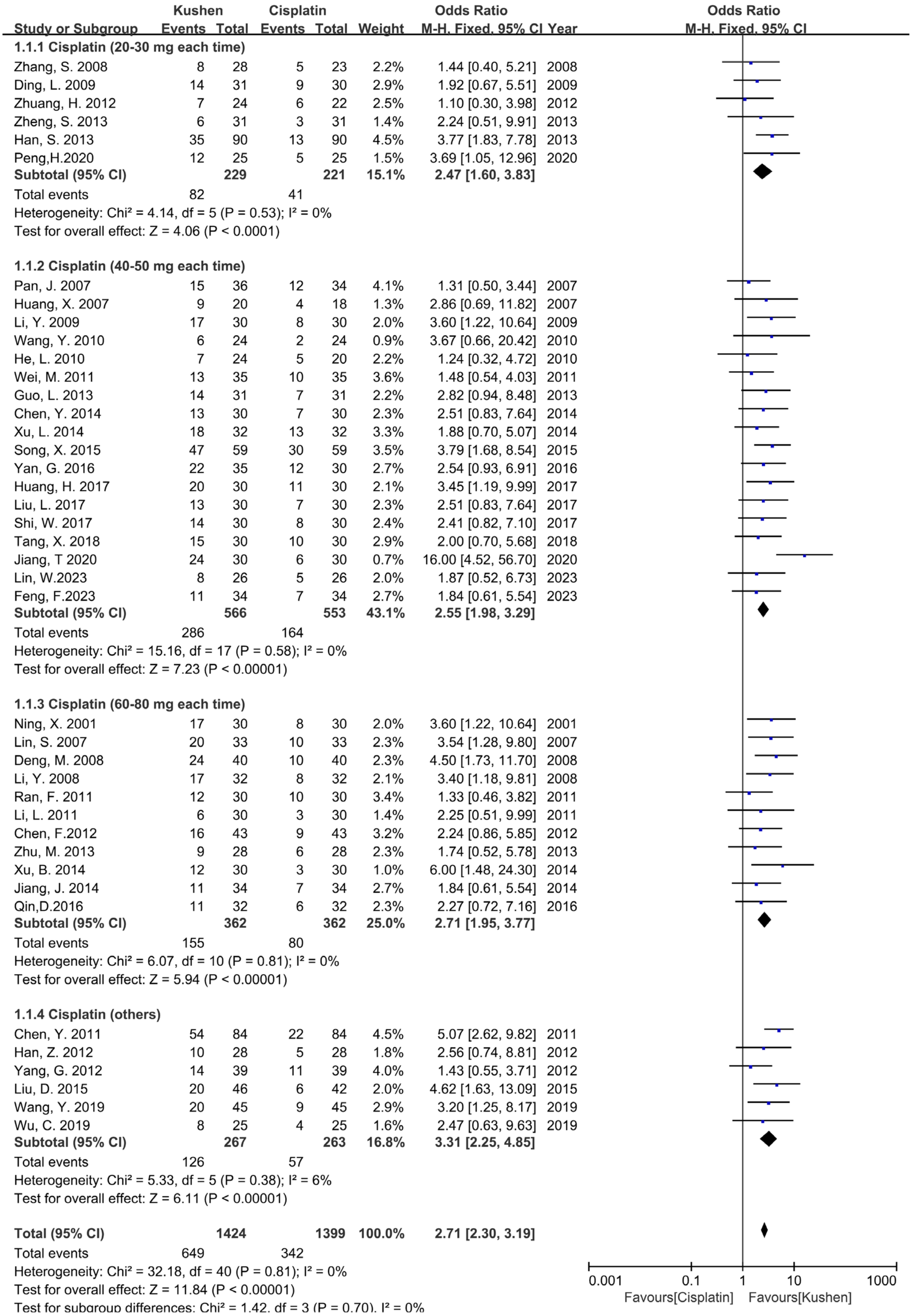


**Figure.S61 Subgroups analysis of complete response via Cisplatin dosage**

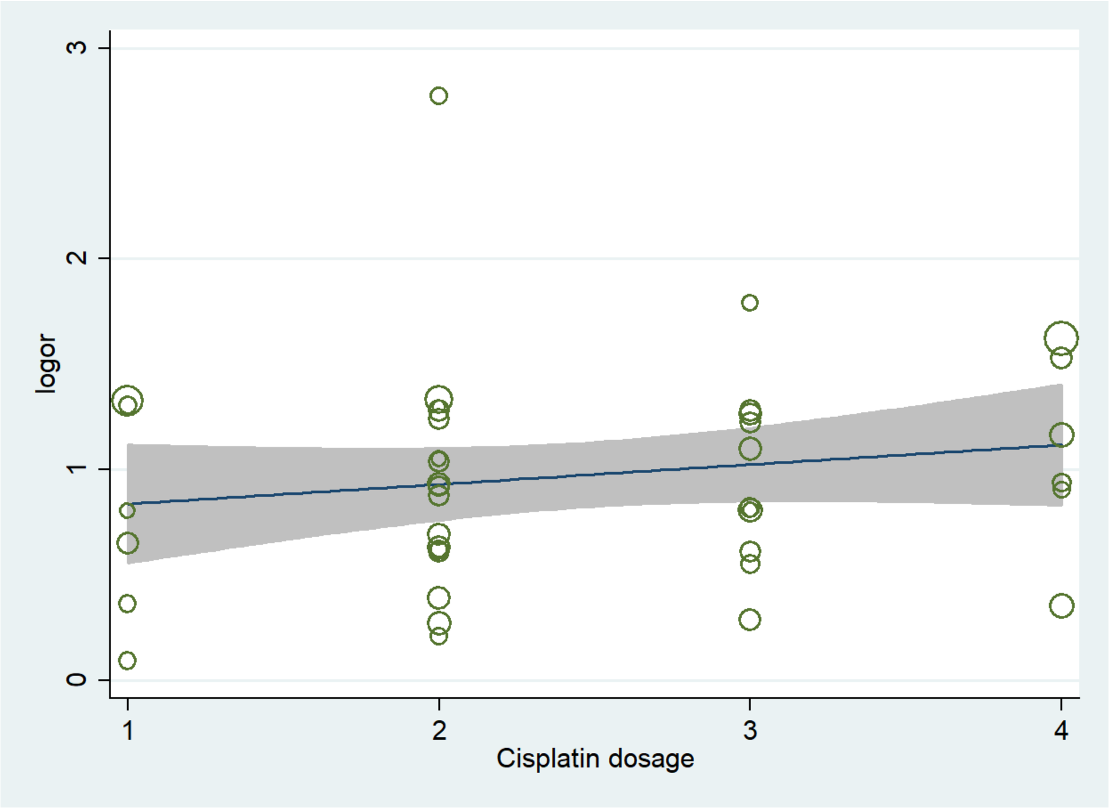


**Figure.S62 Meta-regression of complete response via Cisplatin dosage**

| Study or Subgroup | Kushen Events | Kushen Total | Cisplatin Events | Cisplatin Total | Weight | Odds Ratio M-H, Fixed, 95% CI |
|---|---|---|---|---|---|---|
| **1.2.1 Cisplatin (20-30 mg each time)** | | | | | | |
| Ding, L. 2009 | 3 | 31 | 13 | 30 | 2.4% | 0.14 [0.03, 0.56] |
| Han, S. 2013 | 11 | 90 | 40 | 90 | 7.1% | 0.17 [0.08, 0.37] |
| Peng,H.2020 | 7 | 25 | 15 | 25 | 2.2% | 0.26 [0.08, 0.85] |
| Zhang, S. 2008 | 5 | 28 | 10 | 23 | 1.8% | 0.28 [0.08, 1.01] |
| Zheng, S. 2013 | 6 | 31 | 16 | 31 | 2.6% | 0.23 [0.07, 0.70] |
| Zhuang, H. 2012 | 6 | 24 | 8 | 22 | 1.3% | 0.58 [0.16, 2.07] |
| **Subtotal (95% CI)** | | **229** | | **221** | **17.5%** | **0.23 [0.15, 0.36]** |
| Total events | 38 | | 102 | | | |
| Heterogeneity: Chi² = 3.22, df = 5 (P = 0.67); I² = 0% | | | | | | |
| Test for overall effect: Z = 6.54 (P < 0.00001) | | | | | | |
| **1.2.2 Cisplatin (40-50 mg each time)** | | | | | | |
| Chen, Y. 2014 | 7 | 30 | 14 | 30 | 2.2% | 0.35 [0.11, 1.05] |
| Feng, F.2023 | 7 | 34 | 15 | 34 | 2.4% | 0.33 [0.11, 0.96] |
| Guo, L. 2013 | 7 | 31 | 15 | 31 | 2.4% | 0.31 [0.10, 0.93] |
| He, L. 2010 | 8 | 24 | 11 | 20 | 1.6% | 0.41 [0.12, 1.39] |
| Huang, H. 2017 | 3 | 30 | 11 | 30 | 2.0% | 0.19 [0.05, 0.78] |
| Huang, X. 2007 | 3 | 20 | 8 | 18 | 1.5% | 0.22 [0.05, 1.03] |
| Jiang, T 2020 | 2 | 30 | 13 | 30 | 2.5% | 0.09 [0.02, 0.47] |
| Li, Y. 2009 | 5 | 30 | 8 | 30 | 1.4% | 0.55 [0.16, 1.93] |
| Lin, W.2023 | 7 | 26 | 15 | 26 | 2.2% | 0.27 [0.08, 0.87] |
| Liu, L. 2017 | 6 | 30 | 14 | 30 | 2.3% | 0.29 [0.09, 0.90] |
| Pan, J. 2007 | 3 | 36 | 9 | 34 | 1.7% | 0.25 [0.06, 1.03] |
| Shi, W. 2017 | 6 | 30 | 13 | 30 | 2.1% | 0.33 [0.10, 1.03] |
| Song, X. 2015 | 5 | 59 | 19 | 59 | 3.5% | 0.19 [0.07, 0.57] |
| Tang, X. 2018 | 4 | 30 | 11 | 30 | 1.9% | 0.27 [0.07, 0.96] |
| Wang, Y. 2010 | 2 | 24 | 10 | 24 | 1.9% | 0.13 [0.02, 0.67] |
| Wei, M. 2011 | 6 | 35 | 11 | 35 | 1.9% | 0.45 [0.15, 1.40] |
| Xu, L. 2014 | 7 | 32 | 15 | 32 | 2.4% | 0.32 [0.11, 0.94] |
| Yan, G. 2016 | 4 | 35 | 11 | 30 | 2.1% | 0.22 [0.06, 0.80] |
| **Subtotal (95% CI)** | | **566** | | **553** | **37.9%** | **0.28 [0.21, 0.37]** |
| Total events | 92 | | 223 | | | |
| Heterogeneity: Chi² = 6.19, df = 17 (P = 0.99); I² = 0% | | | | | | |
| Test for overall effect: Z = 8.79 (P < 0.00001) | | | | | | |
| **1.2.3 Cisplatin (60-80 mg each time)** | | | | | | |
| Chen, F.2012 | 7 | 43 | 20 | 43 | 3.4% | 0.22 [0.08, 0.61] |
| Deng, M. 2008 | 6 | 40 | 12 | 40 | 2.1% | 0.41 [0.14, 1.24] |
| Jiang, J. 2014 | 5 | 34 | 13 | 34 | 2.3% | 0.28 [0.09, 0.90] |
| Li, L. 2011 | 7 | 30 | 17 | 30 | 2.6% | 0.23 [0.08, 0.71] |
| Li, Y. 2008 | 5 | 32 | 8 | 32 | 1.4% | 0.56 [0.16, 1.93] |
| Lin, S. 2007 | 4 | 33 | 11 | 33 | 2.0% | 0.28 [0.08, 0.98] |
| Ning, X. 2001 | 5 | 30 | 8 | 30 | 1.4% | 0.55 [0.16, 1.93] |
| Qin,D.2016 | 2 | 32 | 15 | 32 | 2.9% | 0.08 [0.02, 0.37] |
| Ran, F. 2011 | 4 | 30 | 12 | 30 | 2.1% | 0.23 [0.06, 0.83] |
| Xu, B. 2014 | 11 | 30 | 17 | 30 | 2.2% | 0.44 [0.16, 1.25] |
| Zhu, M. 2013 | 5 | 28 | 9 | 28 | 1.5% | 0.46 [0.13, 1.60] |
| **Subtotal (95% CI)** | | **362** | | **362** | **23.7%** | **0.31 [0.22, 0.44]** |
| Total events | 61 | | 142 | | | |
| Heterogeneity: Chi² = 6.70, df = 10 (P = 0.75); I² = 0% | | | | | | |
| Test for overall effect: Z = 6.60 (P < 0.00001) | | | | | | |
| **1.2.4 Cisplatin (others)** | | | | | | |
| Chen, Y. 2011 | 10 | 84 | 38 | 84 | 6.8% | 0.16 [0.07, 0.36] |
| Han, Z. 2012 | 5 | 28 | 13 | 28 | 2.2% | 0.25 [0.07, 0.85] |
| Liu, D. 2015 | 8 | 46 | 21 | 42 | 3.7% | 0.21 [0.08, 0.56] |
| Wang, Y. 2019 | 8 | 45 | 20 | 45 | 3.3% | 0.27 [0.10, 0.71] |
| Wu, C. 2019 | 7 | 25 | 15 | 25 | 2.2% | 0.26 [0.08, 0.85] |
| Yang, G. 2012 | 6 | 39 | 16 | 39 | 2.7% | 0.26 [0.09, 0.77] |
| **Subtotal (95% CI)** | | **267** | | **263** | **20.9%** | **0.22 [0.15, 0.33]** |
| Total events | 44 | | 123 | | | |
| Heterogeneity: Chi² = 0.94, df = 5 (P = 0.97); I² = 0% | | | | | | |
| Test for overall effect: Z = 7.27 (P < 0.00001) | | | | | | |
| **Total (95% CI)** | | **1424** | | **1399** | **100.0%** | **0.26 [0.22, 0.32]** |
| Total events | 235 | | 590 | | | |
| Heterogeneity: Chi² = 19.19, df = 40 (P = 1.00); I² = 0% | | | | | | |
| Test for overall effect: Z = 14.66 (P < 0.00001) | | | | | | |
| Test for subgroup differences: Chi² = 1.95, df = 3 (P = 0.58), I² = 0% | | | | | | |

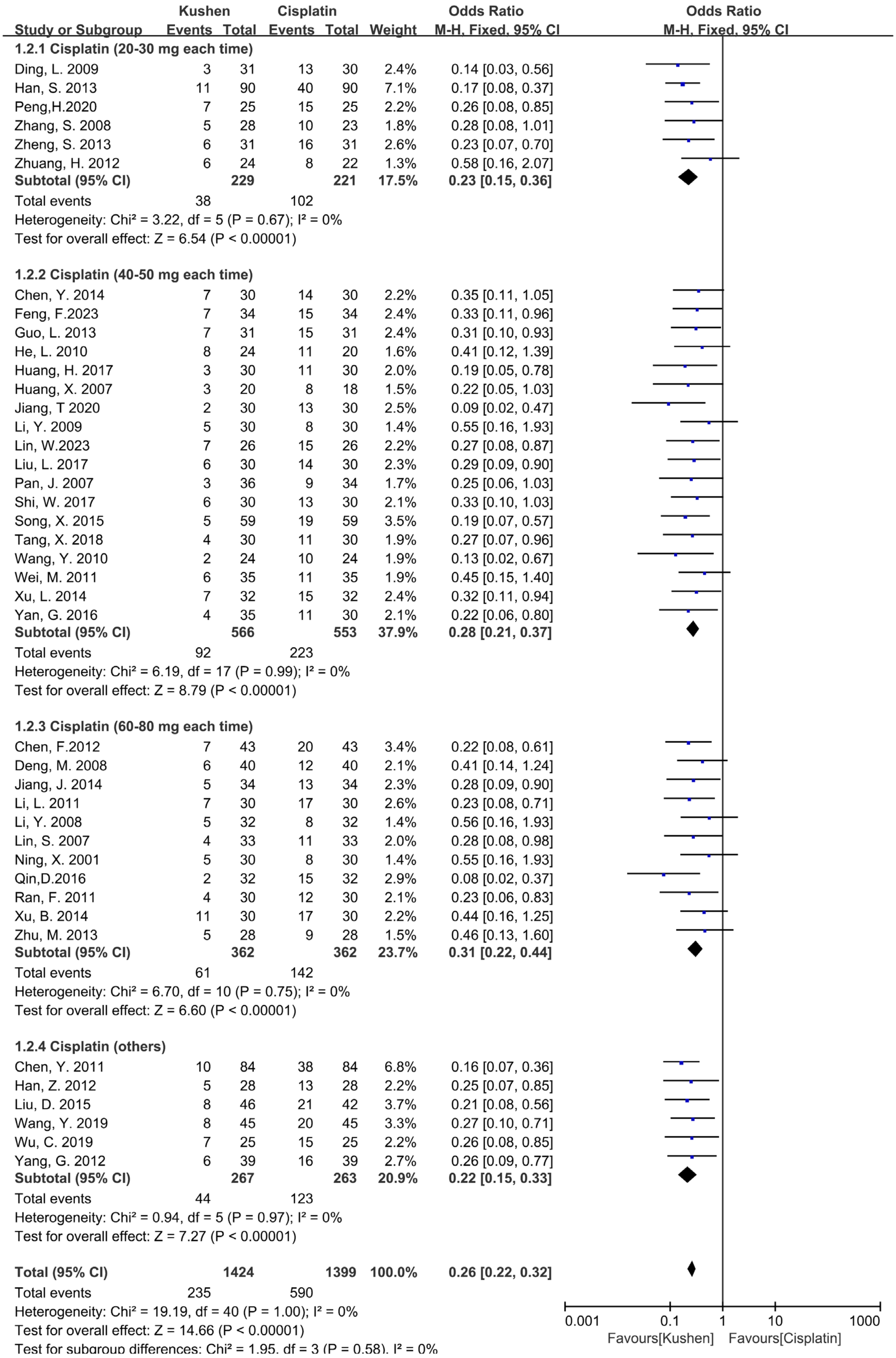


**Figure.S63 Subgroups analysis of pleurodesis failure via Cisplatin dosage**

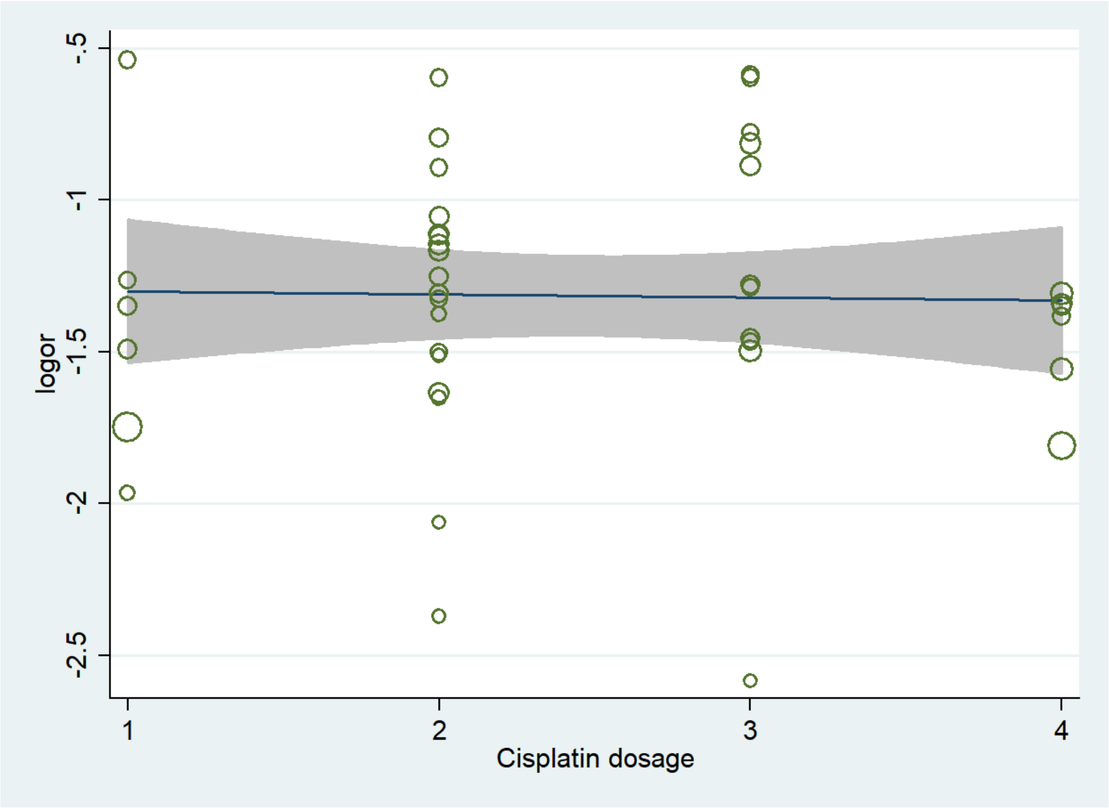


**Figure.S64 Meta-regression of pleurodesis failure via Cisplatin dosage**

| Study or Subgroup | Kushen Events | Kushen Total | Cisplatin Events | Cisplatin Total | Weight | Odds Ratio M-H, Fixed, 95% CI | Year |
|---|---|---|---|---|---|---|---|
| **1.1.1 Equivalent dosage** | | | | | | | |
| Lin, S. 2007 | 20 | 33 | 10 | 33 | 2.3% | 3.54 [1.28, 9.80] | 2007 |
| Pan, J. 2007 | 15 | 36 | 12 | 34 | 4.1% | 1.31 [0.50, 3.44] | 2007 |
| Huang, X. 2007 | 9 | 20 | 4 | 18 | 1.3% | 2.86 [0.69, 11.82] | 2007 |
| Zhang, S. 2008 | 8 | 28 | 5 | 23 | 2.2% | 1.44 [0.40, 5.21] | 2008 |
| Li, Y. 2009 | 17 | 30 | 8 | 30 | 2.0% | 3.60 [1.22, 10.64] | 2009 |
| Ding, L. 2009 | 14 | 31 | 9 | 30 | 2.9% | 1.92 [0.67, 5.51] | 2009 |
| Wang, Y. 2010 | 6 | 24 | 2 | 24 | 0.9% | 3.67 [0.66, 20.42] | 2010 |
| He, L. 2010 | 7 | 24 | 5 | 20 | 2.2% | 1.24 [0.32, 4.72] | 2010 |
| Chen, Y. 2011 | 54 | 84 | 22 | 84 | 4.5% | 5.07 [2.62, 9.82] | 2011 |
| Wei, M. 2011 | 13 | 35 | 10 | 35 | 3.6% | 1.48 [0.54, 4.03] | 2011 |
| Han, Z. 2012 | 10 | 28 | 5 | 28 | 1.8% | 2.56 [0.74, 8.81] | 2012 |
| Chen, F.2012 | 16 | 43 | 9 | 43 | 3.2% | 2.24 [0.86, 5.85] | 2012 |
| Yang, G. 2012 | 14 | 39 | 11 | 39 | 4.0% | 1.43 [0.55, 3.71] | 2012 |
| Zhuang, H. 2012 | 7 | 24 | 6 | 22 | 2.5% | 1.10 [0.30, 3.98] | 2012 |
| Guo, L. 2013 | 14 | 31 | 7 | 31 | 2.2% | 2.82 [0.94, 8.48] | 2013 |
| Han, S. 2013 | 35 | 90 | 13 | 90 | 4.5% | 3.77 [1.83, 7.78] | 2013 |
| Zhu, M. 2013 | 9 | 28 | 6 | 28 | 2.3% | 1.74 [0.52, 5.78] | 2013 |
| Zheng, S. 2013 | 6 | 31 | 3 | 31 | 1.4% | 2.24 [0.51, 9.91] | 2013 |
| Chen, Y. 2014 | 13 | 30 | 7 | 30 | 2.3% | 2.51 [0.83, 7.64] | 2014 |
| Jiang, J. 2014 | 11 | 34 | 7 | 34 | 2.7% | 1.84 [0.61, 5.54] | 2014 |
| Xu, B. 2014 | 12 | 30 | 3 | 30 | 1.0% | 6.00 [1.48, 24.30] | 2014 |
| Xu, L. 2014 | 18 | 32 | 13 | 32 | 3.3% | 1.88 [0.70, 5.07] | 2014 |
| Song, X. 2015 | 47 | 59 | 30 | 59 | 3.5% | 3.79 [1.68, 8.54] | 2015 |
| Liu, D. 2015 | 20 | 46 | 6 | 42 | 2.0% | 4.62 [1.63, 13.09] | 2015 |
| Yan, G. 2016 | 22 | 35 | 12 | 30 | 2.7% | 2.54 [0.93, 6.91] | 2016 |
| Qin,D.2016 | 11 | 32 | 6 | 32 | 2.3% | 2.27 [0.72, 7.16] | 2016 |
| Huang, H. 2017 | 20 | 30 | 11 | 30 | 2.1% | 3.45 [1.19, 9.99] | 2017 |
| Liu, L. 2017 | 13 | 30 | 7 | 30 | 2.3% | 2.51 [0.83, 7.64] | 2017 |
| Shi, W. 2017 | 14 | 30 | 8 | 30 | 2.4% | 2.41 [0.82, 7.10] | 2017 |
| Tang, X. 2018 | 15 | 30 | 10 | 30 | 2.9% | 2.00 [0.70, 5.68] | 2018 |
| Wang, Y. 2019 | 20 | 45 | 9 | 45 | 2.9% | 3.20 [1.25, 8.17] | 2019 |
| Wu, C. 2019 | 8 | 25 | 4 | 25 | 1.6% | 2.47 [0.63, 9.63] | 2019 |
| Peng,H.2020 | 12 | 25 | 5 | 25 | 1.5% | 3.69 [1.05, 12.96] | 2020 |
| Feng, F.2023 | 11 | 34 | 7 | 34 | 2.7% | 1.84 [0.61, 5.54] | 2023 |
| Lin, W.2023 | 8 | 26 | 5 | 26 | 2.0% | 1.87 [0.52, 6.73] | 2023 |
| **Subtotal (95% CI)** | | **1232** | | **1207** | **88.1%** | **2.58 [2.16, 3.08]** | |
| Total events | 549 | | 297 | | | | |
| Heterogeneity: Chi² = 20.96, df = 34 (P = 0.96); I² = 0% | | | | | | | |
| Test for overall effect: Z = 10.45 (P < 0.00001) | | | | | | | |
| **1.1.2 Low vs high-dosage** | | | | | | | |
| Ning, X. 2001 | 17 | 30 | 8 | 30 | 2.0% | 3.60 [1.22, 10.64] | 2001 |
| Deng, M. 2008 | 24 | 40 | 10 | 40 | 2.3% | 4.50 [1.73, 11.70] | 2008 |
| Li, Y. 2008 | 17 | 32 | 8 | 32 | 2.1% | 3.40 [1.18, 9.81] | 2008 |
| Ran, F. 2011 | 12 | 30 | 10 | 30 | 3.4% | 1.33 [0.46, 3.82] | 2011 |
| Li, L. 2011 | 6 | 30 | 3 | 30 | 1.4% | 2.25 [0.51, 9.99] | 2011 |
| Jiang, T 2020 | 24 | 30 | 6 | 30 | 0.7% | 16.00 [4.52, 56.70] | 2020 |
| **Subtotal (95% CI)** | | **192** | | **192** | **11.9%** | **3.64 [2.34, 5.67]** | |
| Total events | 100 | | 45 | | | | |
| Heterogeneity: Chi² = 9.36, df = 5 (P = 0.10); I² = 47% | | | | | | | |
| Test for overall effect: Z = 5.72 (P < 0.00001) | | | | | | | |
| **Total (95% CI)** | | **1424** | | **1399** | **100.0%** | **2.71 [2.30, 3.19]** | |
| Total events | 649 | | 342 | | | | |
| Heterogeneity: Chi² = 32.18, df = 40 (P = 0.81); I² = 0% | | | | | | | |
| Test for overall effect: Z = 11.84 (P < 0.00001) | | | | | | | |
| Test for subgroup differences: Chi² = 2.00, df = 1 (P = 0.16), I² = 50.0% | | | | | | | |

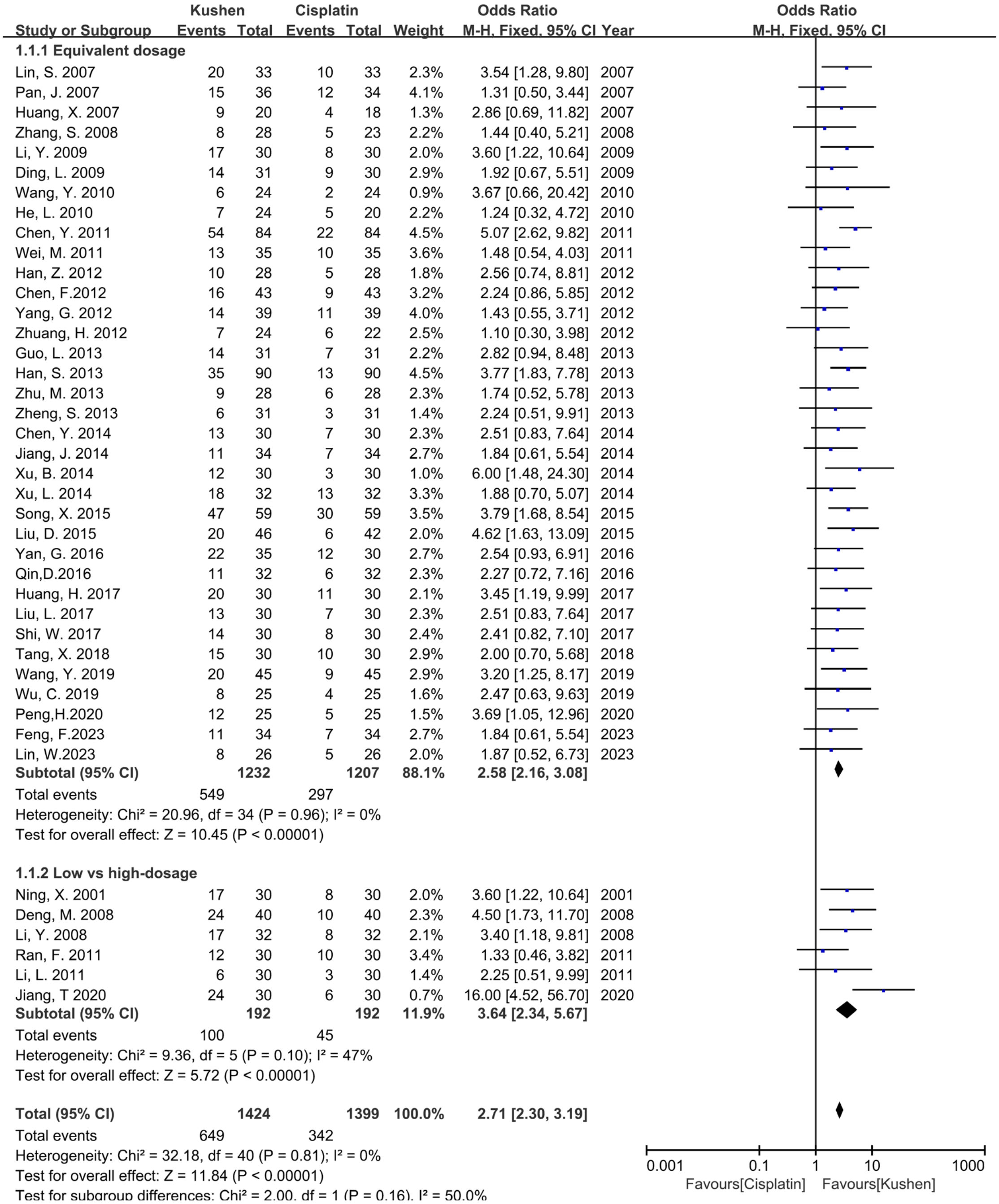


**Figure.S65 Subgroups analysis of complete response via dosage difference of cisplatin**

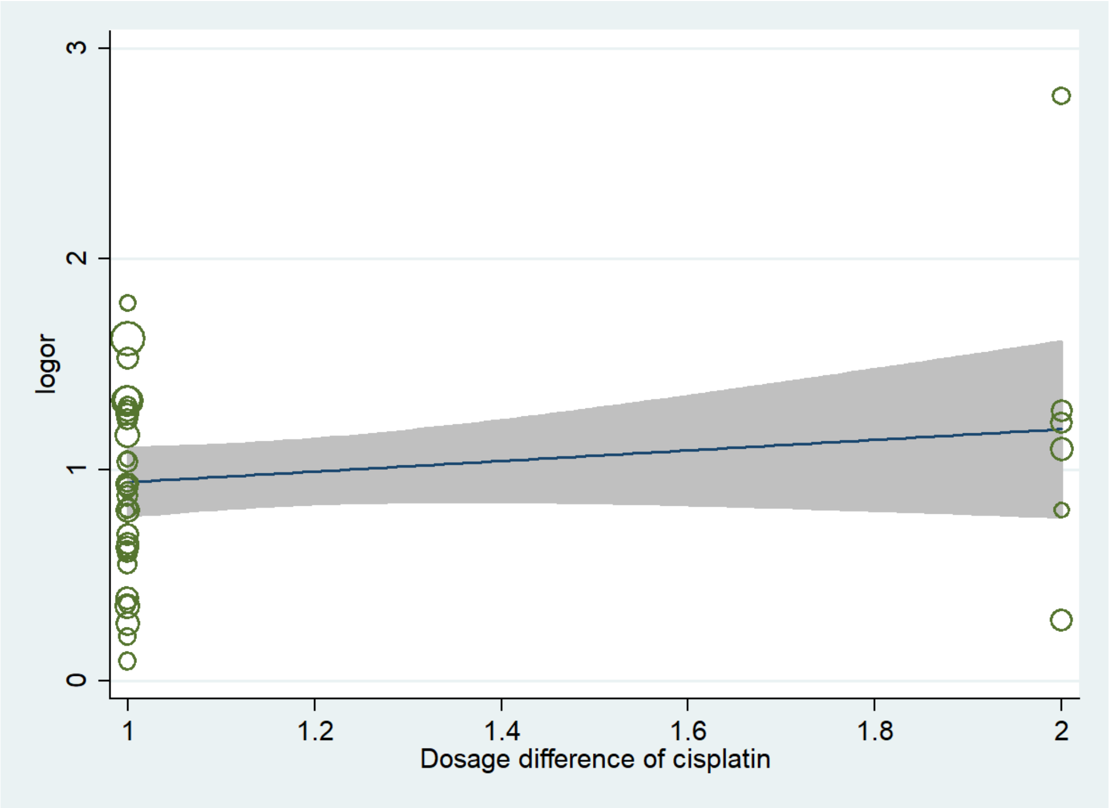


**Figure.S66 Meta-regression of complete response via dosage difference of cisplatin**

| Study or Subgroup | Kushen Events | Kushen Total | Cisplatin Events | Cisplatin Total | Weight | Odds Ratio M-H, Fixed, 95% CI | Year |
|---|---|---|---|---|---|---|---|
| **1.2.1 Equivalent dosage** | | | | | | | |
| Pan, J. 2007 | 3 | 36 | 9 | 34 | 1.7% | 0.25 [0.06, 1.03] | 2007 |
| Lin, S. 2007 | 4 | 33 | 11 | 33 | 2.0% | 0.28 [0.08, 0.98] | 2007 |
| Huang, X. 2007 | 3 | 20 | 8 | 18 | 1.5% | 0.22 [0.05, 1.03] | 2007 |
| Zhang, S. 2008 | 5 | 28 | 10 | 23 | 1.8% | 0.28 [0.08, 1.01] | 2008 |
| Li, Y. 2009 | 5 | 30 | 8 | 30 | 1.4% | 0.55 [0.16, 1.93] | 2009 |
| Ding, L. 2009 | 3 | 31 | 13 | 30 | 2.4% | 0.14 [0.03, 0.56] | 2009 |
| Wang, Y. 2010 | 2 | 24 | 10 | 24 | 1.9% | 0.13 [0.02, 0.67] | 2010 |
| He, L. 2010 | 8 | 24 | 11 | 20 | 1.6% | 0.41 [0.12, 1.39] | 2010 |
| Chen, Y. 2011 | 10 | 84 | 38 | 84 | 6.8% | 0.16 [0.07, 0.36] | 2011 |
| Wei, M. 2011 | 6 | 35 | 11 | 35 | 1.9% | 0.45 [0.15, 1.40] | 2011 |
| Chen, F.2012 | 7 | 43 | 20 | 43 | 3.4% | 0.22 [0.08, 0.61] | 2012 |
| Yang, G. 2012 | 6 | 39 | 16 | 39 | 2.7% | 0.26 [0.09, 0.77] | 2012 |
| Han, Z. 2012 | 5 | 28 | 13 | 28 | 2.2% | 0.25 [0.07, 0.85] | 2012 |
| Zhuang, H. 2012 | 6 | 24 | 8 | 22 | 1.3% | 0.58 [0.16, 2.07] | 2012 |
| Han, S. 2013 | 11 | 90 | 40 | 90 | 7.1% | 0.17 [0.08, 0.37] | 2013 |
| Zhu, M. 2013 | 5 | 28 | 9 | 28 | 1.5% | 0.46 [0.13, 1.60] | 2013 |
| Guo, L. 2013 | 7 | 31 | 15 | 31 | 2.4% | 0.31 [0.10, 0.93] | 2013 |
| Zheng, S. 2013 | 6 | 31 | 16 | 31 | 2.6% | 0.23 [0.07, 0.70] | 2013 |
| Jiang, J. 2014 | 5 | 34 | 13 | 34 | 2.3% | 0.28 [0.09, 0.90] | 2014 |
| Chen, Y. 2014 | 7 | 30 | 14 | 30 | 2.2% | 0.35 [0.11, 1.05] | 2014 |
| Xu, L. 2014 | 7 | 32 | 15 | 32 | 2.4% | 0.32 [0.11, 0.94] | 2014 |
| Xu, B. 2014 | 11 | 30 | 17 | 30 | 2.2% | 0.44 [0.16, 1.25] | 2014 |
| Liu, D. 2015 | 8 | 46 | 21 | 42 | 3.7% | 0.21 [0.08, 0.56] | 2015 |
| Song, X. 2015 | 5 | 59 | 19 | 59 | 3.5% | 0.19 [0.07, 0.57] | 2015 |
| Qin,D.2016 | 2 | 32 | 15 | 32 | 2.9% | 0.08 [0.02, 0.37] | 2016 |
| Yan, G. 2016 | 4 | 35 | 11 | 30 | 2.1% | 0.22 [0.06, 0.80] | 2016 |
| Liu, L. 2017 | 6 | 30 | 14 | 30 | 2.3% | 0.29 [0.09, 0.90] | 2017 |
| Huang, H. 2017 | 3 | 30 | 11 | 30 | 2.0% | 0.19 [0.05, 0.78] | 2017 |
| Shi, W. 2017 | 6 | 30 | 13 | 30 | 2.1% | 0.33 [0.10, 1.03] | 2017 |
| Tang, X. 2018 | 4 | 30 | 11 | 30 | 1.9% | 0.27 [0.07, 0.96] | 2018 |
| Wang, Y. 2019 | 8 | 45 | 20 | 45 | 3.3% | 0.27 [0.10, 0.71] | 2019 |
| Wu, C. 2019 | 7 | 25 | 15 | 25 | 2.2% | 0.26 [0.08, 0.85] | 2019 |
| Peng,H.2020 | 7 | 25 | 15 | 25 | 2.2% | 0.26 [0.08, 0.85] | 2020 |
| Lin, W.2023 | 7 | 26 | 15 | 26 | 2.2% | 0.27 [0.08, 0.87] | 2023 |
| Feng, F.2023 | 7 | 34 | 15 | 34 | 2.4% | 0.33 [0.11, 0.96] | 2023 |
| **Subtotal (95% CI)** | | **1232** | | **1207** | **88.0%** | **0.26 [0.21, 0.31]** | |
| Total events | 206 | | 520 | | | | |
| Heterogeneity: Chi² = 14.19, df = 34 (P = 1.00); I² = 0% | | | | | | | |
| Test for overall effect: Z = 13.91 (P < 0.00001) | | | | | | | |
| **1.2.2 Low vs high-dosage** | | | | | | | |
| Ning, X. 2001 | 5 | 30 | 8 | 30 | 1.4% | 0.55 [0.16, 1.93] | 2001 |
| Deng, M. 2008 | 6 | 40 | 12 | 40 | 2.1% | 0.41 [0.14, 1.24] | 2008 |
| Li, Y. 2008 | 5 | 32 | 8 | 32 | 1.4% | 0.56 [0.16, 1.93] | 2008 |
| Li, L. 2011 | 7 | 30 | 17 | 30 | 2.6% | 0.23 [0.08, 0.71] | 2011 |
| Ran, F. 2011 | 4 | 30 | 12 | 30 | 2.1% | 0.23 [0.06, 0.83] | 2011 |
| Jiang, T 2020 | 2 | 30 | 13 | 30 | 2.5% | 0.09 [0.02, 0.47] | 2020 |
| **Subtotal (95% CI)** | | **192** | | **192** | **12.0%** | **0.31 [0.19, 0.50]** | |
| Total events | 29 | | 70 | | | | |
| Heterogeneity: Chi² = 4.51, df = 5 (P = 0.48); I² = 0% | | | | | | | |
| Test for overall effect: Z = 4.68 (P < 0.00001) | | | | | | | |
| **Total (95% CI)** | | **1424** | | **1399** | **100.0%** | **0.26 [0.22, 0.32]** | |
| Total events | 235 | | 590 | | | | |
| Heterogeneity: Chi² = 19.19, df = 40 (P = 1.00); I² = 0% | | | | | | | |
| Test for overall effect: Z = 14.66 (P < 0.00001) | | | | | | | |
| Test for subgroup differences: Chi² = 0.42, df = 1 (P = 0.52), I² = 0% | | | | | | | |

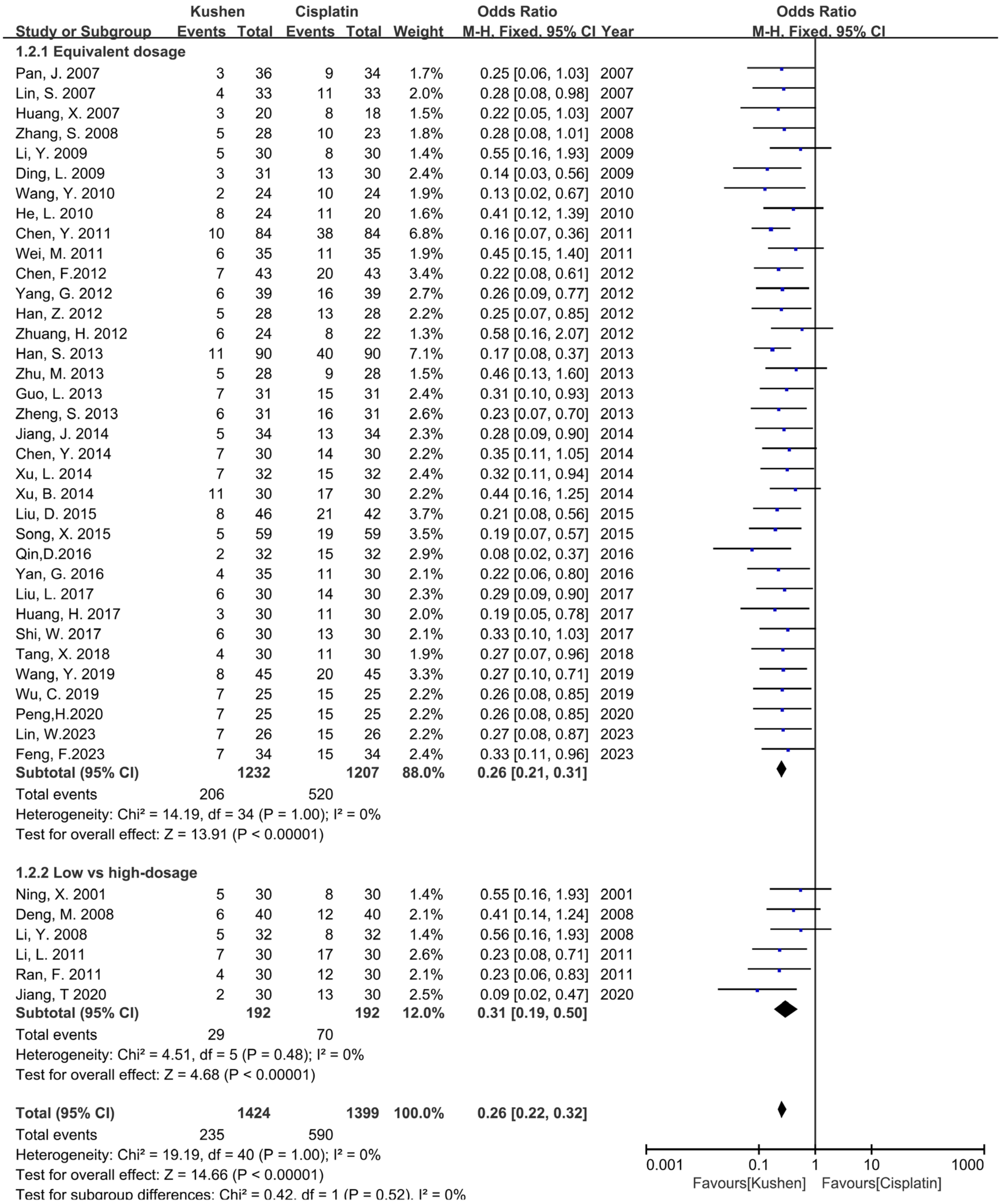


**Figure.S67 Subgroups analysis of pleurodesis failure via dosage difference of cisplatin**

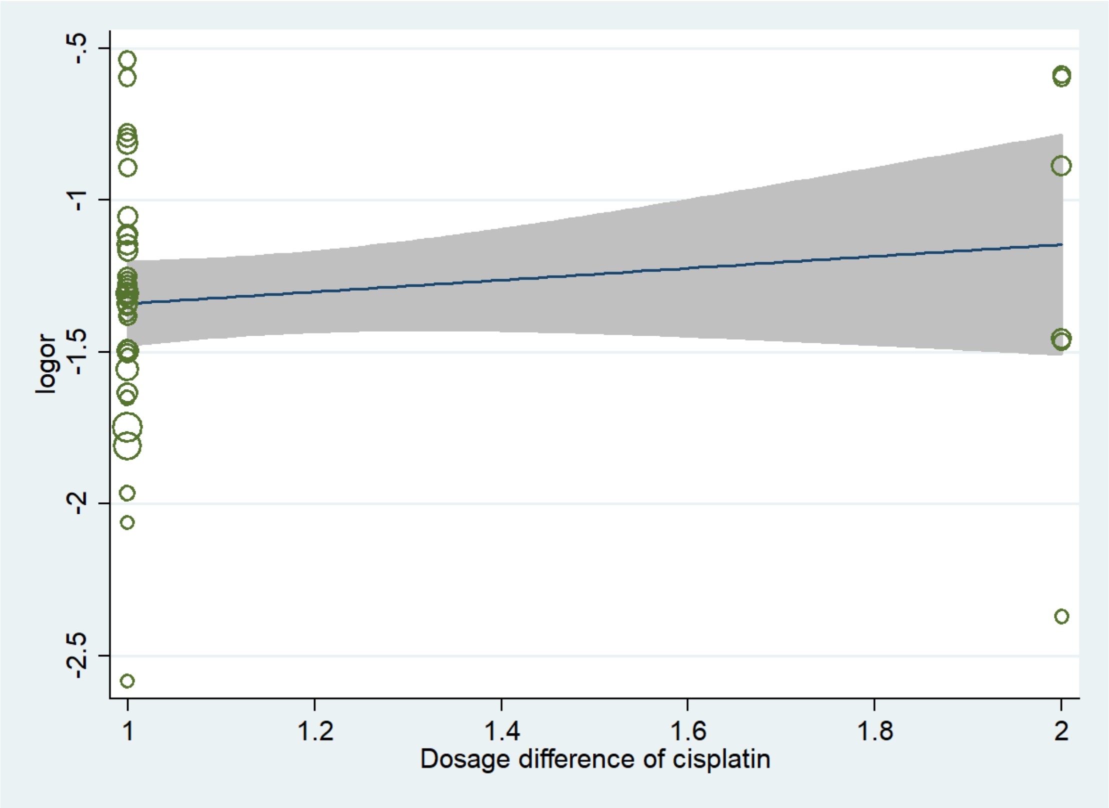


**Figure.S68 Meta-regression of pleurodesis failure via dosage difference of cisplatin**

| Study or Subgroup | Kushen Events | Kushen Total | Cisplatin Events | Cisplatin Total | Weight | Odds Ratio M-H, Fixed, 95% CI | Year |
|---|---|---|---|---|---|---|---|
| **1.1.1 Ostrowskimj** | | | | | | | |
| Ning, X. 2001 | 17 | 30 | 8 | 30 | 2.0% | 3.60 [1.22, 10.64] | 2001 |
| Pan, J. 2007 | 15 | 36 | 12 | 34 | 4.1% | 1.31 [0.50, 3.44] | 2007 |
| Huang, X. 2007 | 9 | 20 | 4 | 18 | 1.3% | 2.86 [0.69, 11.82] | 2007 |
| Zhang, S. 2008 | 8 | 28 | 5 | 23 | 2.2% | 1.44 [0.40, 5.21] | 2008 |
| Deng, M. 2008 | 24 | 40 | 10 | 40 | 2.3% | 4.50 [1.73, 11.70] | 2008 |
| Li, Y. 2008 | 17 | 32 | 8 | 32 | 2.1% | 3.40 [1.18, 9.81] | 2008 |
| Li, Y. 2009 | 17 | 30 | 8 | 30 | 2.0% | 3.60 [1.22, 10.64] | 2009 |
| Ding, L. 2009 | 14 | 31 | 9 | 30 | 2.9% | 1.92 [0.67, 5.51] | 2009 |
| Wang, Y. 2010 | 6 | 24 | 2 | 24 | 0.9% | 3.67 [0.66, 20.42] | 2010 |
| He, L. 2010 | 7 | 24 | 5 | 20 | 2.2% | 1.24 [0.32, 4.72] | 2010 |
| Chen, Y. 2011 | 54 | 84 | 22 | 84 | 4.5% | 5.07 [2.62, 9.82] | 2011 |
| Ran, F. 2011 | 12 | 30 | 10 | 30 | 3.4% | 1.33 [0.46, 3.82] | 2011 |
| Wei, M. 2011 | 13 | 35 | 10 | 35 | 3.6% | 1.48 [0.54, 4.03] | 2011 |
| Li, L. 2011 | 6 | 30 | 3 | 30 | 1.4% | 2.25 [0.51, 9.99] | 2011 |
| Yang, G. 2012 | 14 | 39 | 11 | 39 | 4.0% | 1.43 [0.55, 3.71] | 2012 |
| Guo, L. 2013 | 14 | 31 | 7 | 31 | 2.2% | 2.82 [0.94, 8.48] | 2013 |
| Zheng, S. 2013 | 6 | 31 | 3 | 31 | 1.4% | 2.24 [0.51, 9.91] | 2013 |
| Chen, Y. 2014 | 13 | 30 | 7 | 30 | 2.3% | 2.51 [0.83, 7.64] | 2014 |
| Jiang, J. 2014 | 11 | 34 | 7 | 34 | 2.7% | 1.84 [0.61, 5.54] | 2014 |
| Xu, L. 2014 | 18 | 32 | 13 | 32 | 3.3% | 1.88 [0.70, 5.07] | 2014 |
| Song, X. 2015 | 47 | 59 | 30 | 59 | 3.5% | 3.79 [1.68, 8.54] | 2015 |
| Yan, G. 2016 | 22 | 35 | 12 | 30 | 2.7% | 2.54 [0.93, 6.91] | 2016 |
| Qin,D.2016 | 11 | 32 | 6 | 32 | 2.3% | 2.27 [0.72, 7.16] | 2016 |
| Huang, H. 2017 | 20 | 30 | 11 | 30 | 2.1% | 3.45 [1.19, 9.99] | 2017 |
| Liu, L. 2017 | 13 | 30 | 7 | 30 | 2.3% | 2.51 [0.83, 7.64] | 2017 |
| Shi, W. 2017 | 14 | 30 | 8 | 30 | 2.4% | 2.41 [0.82, 7.10] | 2017 |
| Tang, X. 2018 | 15 | 30 | 10 | 30 | 2.9% | 2.00 [0.70, 5.68] | 2018 |
| Lin, W.2023 | 8 | 26 | 5 | 26 | 2.0% | 1.87 [0.52, 6.73] | 2023 |
| **Subtotal (95% CI)** | | **943** | | **924** | **70.9%** | **2.49 [2.04, 3.04]** | |
| Total events | 445 | | 253 | | | | |
| Heterogeneity: Chi² = 17.22, df = 27 (P = 0.93); I² = 0% | | | | | | | |
| Test for overall effect: Z = 8.99 (P < 0.00001) | | | | | | | |
| **1.1.2 Millar** | | | | | | | |
| Lin, S. 2007 | 20 | 33 | 10 | 33 | 2.3% | 3.54 [1.28, 9.80] | 2007 |
| Zhuang, H. 2012 | 7 | 24 | 6 | 22 | 2.5% | 1.10 [0.30, 3.98] | 2012 |
| Chen, F.2012 | 16 | 43 | 9 | 43 | 3.2% | 2.24 [0.86, 5.85] | 2012 |
| Han, Z. 2012 | 10 | 28 | 5 | 28 | 1.8% | 2.56 [0.74, 8.81] | 2012 |
| Zhu, M. 2013 | 9 | 28 | 6 | 28 | 2.3% | 1.74 [0.52, 5.78] | 2013 |
| Han, S. 2013 | 35 | 90 | 13 | 90 | 4.5% | 3.77 [1.83, 7.78] | 2013 |
| Xu, B. 2014 | 12 | 30 | 3 | 30 | 1.0% | 6.00 [1.48, 24.30] | 2014 |
| Liu, D. 2015 | 20 | 46 | 6 | 42 | 2.0% | 4.62 [1.63, 13.09] | 2015 |
| Wang, Y. 2019 | 20 | 45 | 9 | 45 | 2.9% | 3.20 [1.25, 8.17] | 2019 |
| Wu, C. 2019 | 8 | 25 | 4 | 25 | 1.6% | 2.47 [0.63, 9.63] | 2019 |
| Jiang, T 2020 | 24 | 30 | 6 | 30 | 0.7% | 16.00 [4.52, 56.70] | 2020 |
| Peng,H.2020 | 12 | 25 | 5 | 25 | 1.5% | 3.69 [1.05, 12.96] | 2020 |
| Feng, F.2023 | 11 | 34 | 7 | 34 | 2.7% | 1.84 [0.61, 5.54] | 2023 |
| **Subtotal (95% CI)** | | **481** | | **475** | **29.1%** | **3.23 [2.40, 4.33]** | |
| Total events | 204 | | 89 | | | | |
| Heterogeneity: Chi² = 13.17, df = 12 (P = 0.36); I² = 9% | | | | | | | |
| Test for overall effect: Z = 7.80 (P < 0.00001) | | | | | | | |
| **Total (95% CI)** | | **1424** | | **1399** | **100.0%** | **2.71 [2.30, 3.19]** | |
| Total events | 649 | | 342 | | | | |
| Heterogeneity: Chi² = 32.18, df = 40 (P = 0.81); I² = 0% | | | | | | | |
| Test for overall effect: Z = 11.84 (P < 0.00001) | | | | | | | |
| Test for subgroup differences: Chi² = 2.02, df = 1 (P = 0.16), I² = 50.4% | | | | | | | |

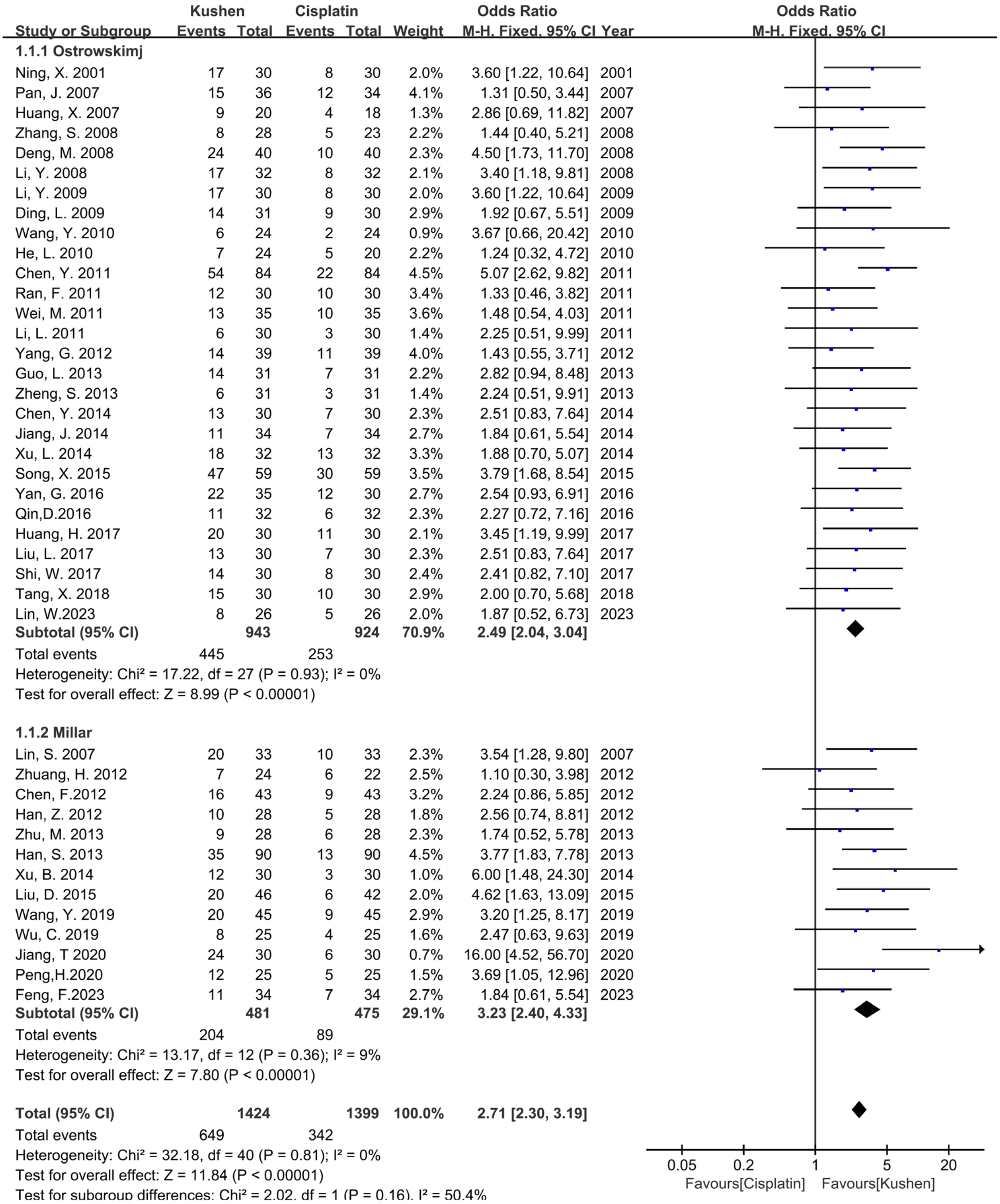


**Figure.S69 Subgroups analysis of complete response via criterion**

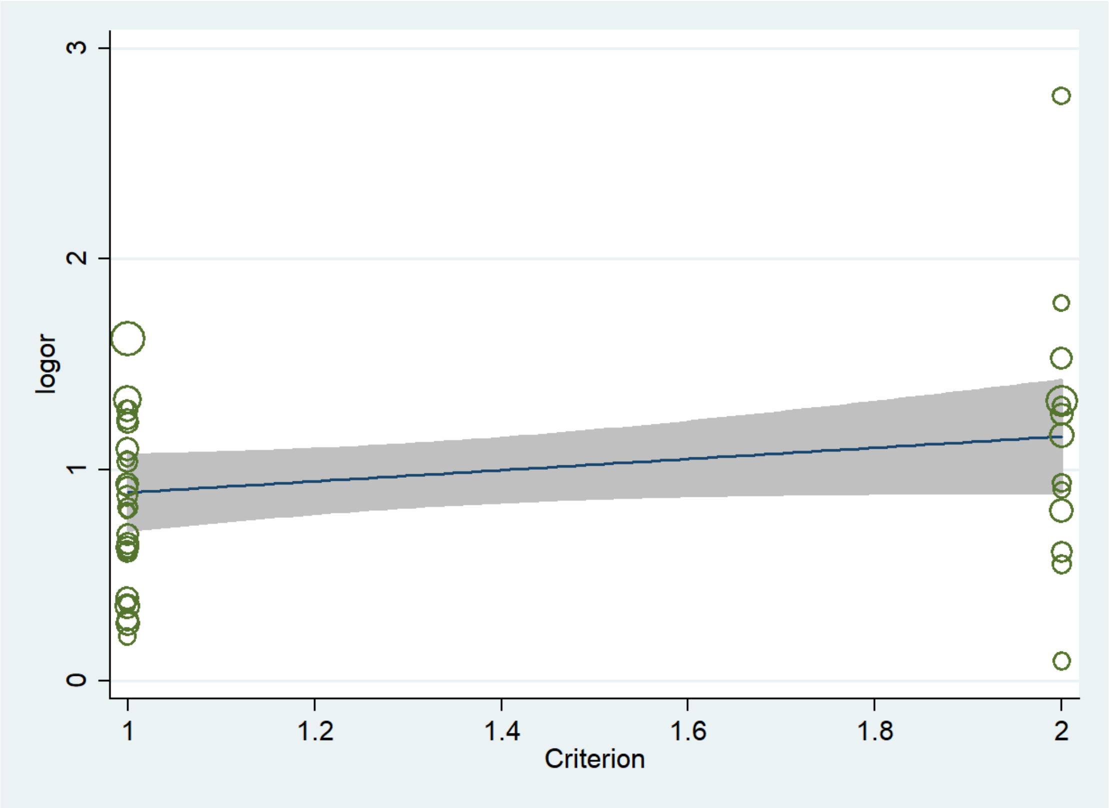


**Figure.S70 Meta-regression of complete response via criterion**

| Study or Subgroup | Kushen Events | Kushen Total | Cisplatin Events | Cisplatin Total | Weight | Odds Ratio M-H, Fixed, 95% CI | Year |
|---|---|---|---|---|---|---|---|
| **1.2.1 Ostrowskimj** | | | | | | | |
| Ning, X. 2001 | 5 | 30 | 8 | 30 | 1.4% | 0.55 [0.16, 1.93] | 2001 |
| Pan, J. 2007 | 3 | 36 | 9 | 34 | 1.7% | 0.25 [0.06, 1.03] | 2007 |
| Huang, X. 2007 | 3 | 20 | 8 | 18 | 1.5% | 0.22 [0.05, 1.03] | 2007 |
| Li, Y. 2008 | 5 | 32 | 8 | 32 | 1.4% | 0.56 [0.16, 1.93] | 2008 |
| Zhang, S. 2008 | 5 | 28 | 10 | 23 | 1.8% | 0.28 [0.08, 1.01] | 2008 |
| Deng, M. 2008 | 6 | 40 | 12 | 40 | 2.1% | 0.41 [0.14, 1.24] | 2008 |
| Li, Y. 2009 | 5 | 30 | 8 | 30 | 1.4% | 0.55 [0.16, 1.93] | 2009 |
| Ding, L. 2009 | 3 | 31 | 13 | 30 | 2.4% | 0.14 [0.03, 0.56] | 2009 |
| Wang, Y. 2010 | 2 | 24 | 10 | 24 | 1.9% | 0.13 [0.02, 0.67] | 2010 |
| He, L. 2010 | 8 | 24 | 11 | 20 | 1.6% | 0.41 [0.12, 1.39] | 2010 |
| Chen, Y. 2011 | 10 | 84 | 38 | 84 | 6.8% | 0.16 [0.07, 0.36] | 2011 |
| Wei, M. 2011 | 6 | 35 | 11 | 35 | 1.9% | 0.45 [0.15, 1.40] | 2011 |
| Li, L. 2011 | 7 | 30 | 17 | 30 | 2.6% | 0.23 [0.08, 0.71] | 2011 |
| Ran, F. 2011 | 4 | 30 | 12 | 30 | 2.1% | 0.23 [0.06, 0.83] | 2011 |
| Yang, G. 2012 | 6 | 39 | 16 | 39 | 2.7% | 0.26 [0.09, 0.77] | 2012 |
| Guo, L. 2013 | 7 | 31 | 15 | 31 | 2.4% | 0.31 [0.10, 0.93] | 2013 |
| Zheng, S. 2013 | 6 | 31 | 16 | 31 | 2.6% | 0.23 [0.07, 0.70] | 2013 |
| Jiang, J. 2014 | 5 | 34 | 13 | 34 | 2.3% | 0.28 [0.09, 0.90] | 2014 |
| Chen, Y. 2014 | 7 | 30 | 14 | 30 | 2.2% | 0.35 [0.11, 1.05] | 2014 |
| Xu, L. 2014 | 7 | 32 | 15 | 32 | 2.4% | 0.32 [0.11, 0.94] | 2014 |
| Song, X. 2015 | 5 | 59 | 19 | 59 | 3.5% | 0.19 [0.07, 0.57] | 2015 |
| Qin,D.2016 | 2 | 32 | 15 | 32 | 2.9% | 0.08 [0.02, 0.37] | 2016 |
| Yan, G. 2016 | 4 | 35 | 11 | 30 | 2.1% | 0.22 [0.06, 0.80] | 2016 |
| Liu, L. 2017 | 6 | 30 | 14 | 30 | 2.3% | 0.29 [0.09, 0.90] | 2017 |
| Huang, H. 2017 | 3 | 30 | 11 | 30 | 2.0% | 0.19 [0.05, 0.78] | 2017 |
| Shi, W. 2017 | 6 | 30 | 13 | 30 | 2.1% | 0.33 [0.10, 1.03] | 2017 |
| Tang, X. 2018 | 4 | 30 | 11 | 30 | 1.9% | 0.27 [0.07, 0.96] | 2018 |
| Lin, W.2023 | 7 | 26 | 15 | 26 | 2.2% | 0.27 [0.08, 0.87] | 2023 |
| **Subtotal (95% CI)** | | **943** | | **924** | **64.1%** | **0.27 [0.21, 0.33]** | |
| Total events | 147 | | 373 | | | | |
| Heterogeneity: Chi² = 12.68, df = 27 (P = 0.99); I² = 0% | | | | | | | |
| Test for overall effect: Z = 11.66 (P < 0.00001) | | | | | | | |
| **1.2.2 Millar** | | | | | | | |
| Lin, S. 2007 | 4 | 33 | 11 | 33 | 2.0% | 0.28 [0.08, 0.98] | 2007 |
| Chen, F.2012 | 7 | 43 | 20 | 43 | 3.4% | 0.22 [0.08, 0.61] | 2012 |
| Han, Z. 2012 | 5 | 28 | 13 | 28 | 2.2% | 0.25 [0.07, 0.85] | 2012 |
| Zhuang, H. 2012 | 6 | 24 | 8 | 22 | 1.3% | 0.58 [0.16, 2.07] | 2012 |
| Han, S. 2013 | 11 | 90 | 40 | 90 | 7.1% | 0.17 [0.08, 0.37] | 2013 |
| Zhu, M. 2013 | 5 | 28 | 9 | 28 | 1.5% | 0.46 [0.13, 1.60] | 2013 |
| Xu, B. 2014 | 11 | 30 | 17 | 30 | 2.2% | 0.44 [0.16, 1.25] | 2014 |
| Liu, D. 2015 | 8 | 46 | 21 | 42 | 3.7% | 0.21 [0.08, 0.56] | 2015 |
| Wang, Y. 2019 | 8 | 45 | 20 | 45 | 3.3% | 0.27 [0.10, 0.71] | 2019 |
| Wu, C. 2019 | 7 | 25 | 15 | 25 | 2.2% | 0.26 [0.08, 0.85] | 2019 |
| Jiang, T 2020 | 2 | 30 | 13 | 30 | 2.5% | 0.09 [0.02, 0.47] | 2020 |
| Peng,H.2020 | 7 | 25 | 15 | 25 | 2.2% | 0.26 [0.08, 0.85] | 2020 |
| Feng, F.2023 | 7 | 34 | 15 | 34 | 2.4% | 0.33 [0.11, 0.96] | 2023 |
| **Subtotal (95% CI)** | | **481** | | **475** | **35.9%** | **0.26 [0.19, 0.35]** | |
| Total events | 88 | | 217 | | | | |
| Heterogeneity: Chi² = 6.48, df = 12 (P = 0.89); I² = 0% | | | | | | | |
| Test for overall effect: Z = 8.88 (P < 0.00001) | | | | | | | |
| **Total (95% CI)** | | **1424** | | **1399** | **100.0%** | **0.26 [0.22, 0.32]** | |
| Total events | 235 | | 590 | | | | |
| Heterogeneity: Chi² = 19.19, df = 40 (P = 1.00); I² = 0% | | | | | | | |
| Test for overall effect: Z = 14.66 (P < 0.00001) | | | | | | | |
| Test for subgroup differences: Chi² = 0.02, df = 1 (P = 0.89), I² = 0% | | | | | | | |

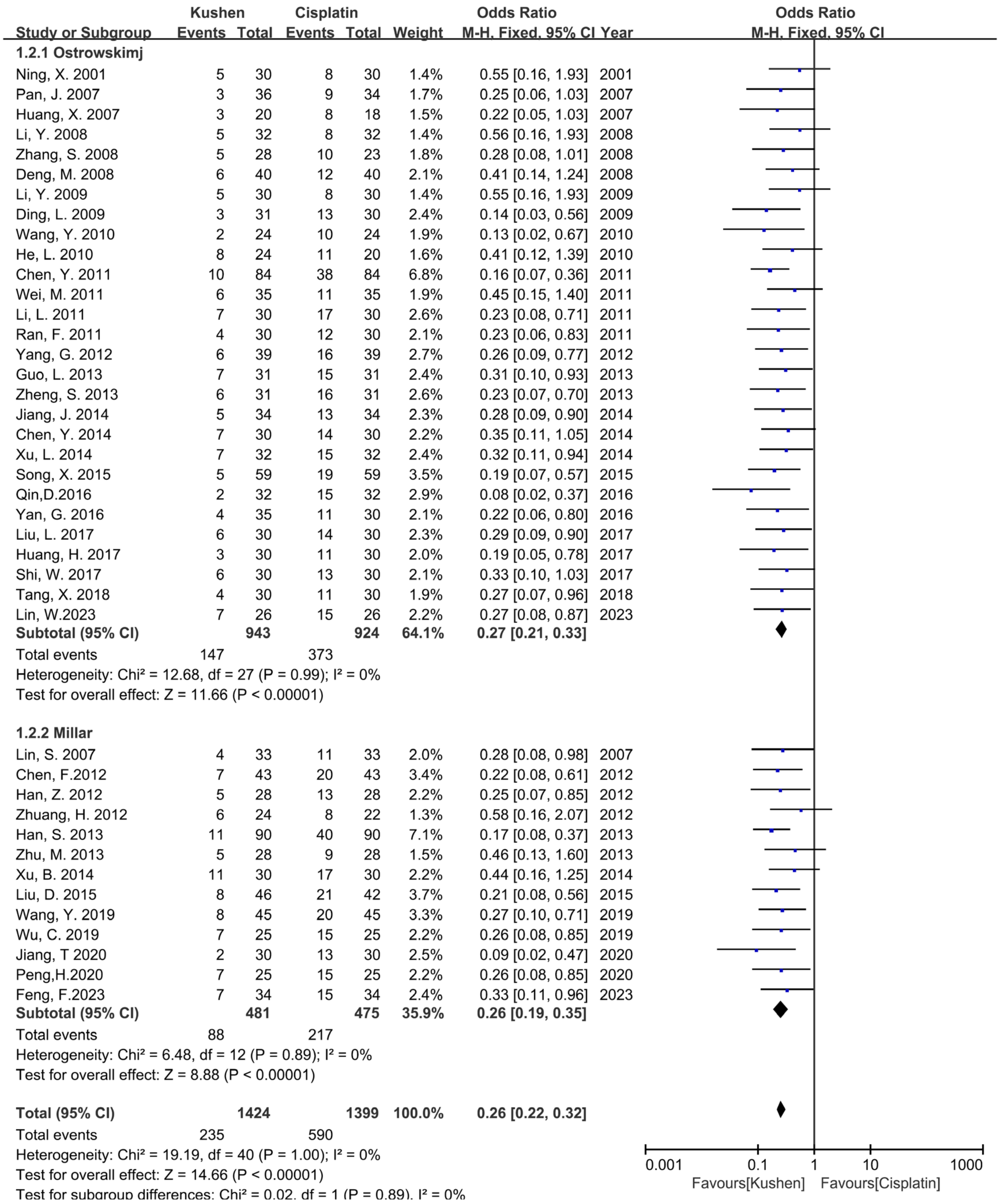


**Figure.S71 Subgroups analysis of pleurodesis failure via criterion**

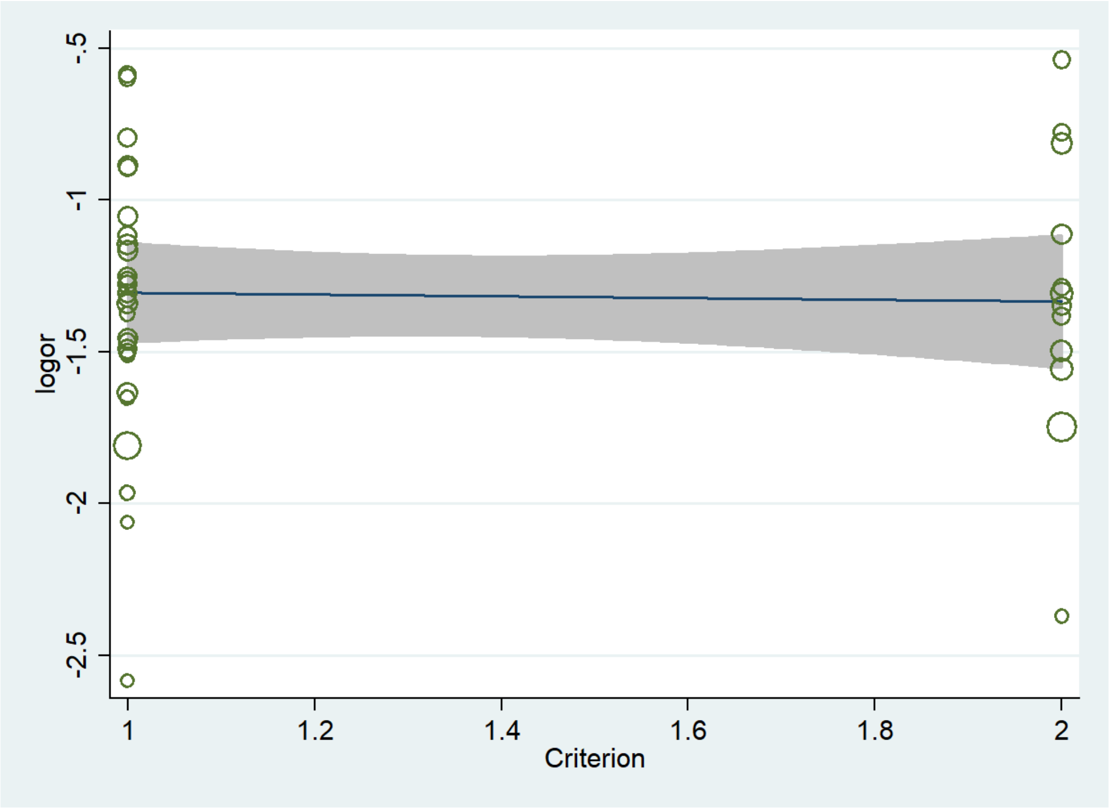


**Figure.S72 Meta-regression of pleurodesis failure via criterion**
